# Advances in dynamic modeling of colorectal cancer signaling-network regions, a path toward targeted therapies

**DOI:** 10.18632/oncotarget.3238

**Published:** 2014-12-31

**Authors:** Lorenzo Tortolina, David J. Duffy, Massimo Maffei, Nicoletta Castagnino, Aimée M. Carmody, Walter Kolch, Boris N. Kholodenko, Cristina De Ambrosi, Annalisa Barla, Elia M. Biganzoli, Alessio Nencioni, Franco Patrone, Alberto Ballestrero, Gabriele Zoppoli, Alessandro Verri, Silvio Parodi

**Affiliations:** ^1^ Department of Internal Medicine and Medical Specializations (DIMI), University of Genoa, Italy; ^2^ Department of Informatics, Bioengineering, Robotics and Systems Engineering (DIBRIS), University of Genoa, Italy; ^3^ Systems Biology Ireland, Conway Institute, University College Dublin, Belfield, Dublin, Ireland; ^4^ Unit of Medical Statistics, Biometry and Bioinformatics “Giulio A. Maccacaro”, Department of Clinical Sciences and Community Health, University of Milan, Italy; ^5^ Istituto a Carattere di Ricerca Clinic - Scientifico (IRCCS), Azienda Ospedaliera Universitaria San Martino, Istituto Nazionale Tumori (IST), Genoa, Italy

**Keywords:** Colorectal cancer, Target therapies, Signaling-network, Dynamic modeling, Onco-protein inhibitors

## Abstract

The interconnected network of pathways downstream of the TGFβ, WNT and EGF-families of receptor ligands play an important role in colorectal cancer pathogenesis.

We studied and implemented dynamic simulations of multiple downstream pathways and described the section of the signaling network considered as a Molecular Interaction Map (MIM). Our simulations used Ordinary Differential Equations (ODEs), which involved 447 reactants and their interactions.

Starting from an initial “physiologic condition”, the model can be adapted to simulate individual pathologic cancer conditions implementing alterations/mutations in relevant onco-proteins. We verified some salient model predictions using the mutated colorectal cancer lines HCT116 and HT29. We measured the amount of MYC and CCND1 mRNAs and AKT and ERK phosphorylated proteins, in response to individual or combination onco-protein inhibitor treatments. Experimental and simulation results were well correlated. Recent independently published results were also predicted by our model.

Even in the presence of an approximate and incomplete signaling network information, a predictive dynamic modeling seems already possible. An important long term road seems to be open and can be pursued further, by incremental steps, toward even larger and better parameterized MIMs. Personalized treatment strategies with rational associations of signaling-proteins inhibitors, could become a realistic goal.

## INTRODUCTION

Colorectal cancer (CRC) can be characterized according to the genomic landscapes of individual CRC patients [1]. Driver mutations can vary to some extent in different CRCs. B. Vogelstein et al. have recently reported [1] a model featuring around 2-5 major driver mutations per individual CRC tumor. Additional somatically inheritable driver alterations were not included. In the COSMIC release of June 2nd 2014 [2], the curators have estimated that an individual cancer can be caused by 5-10 driver mutations (not identical in different tumors), against a background of more than 10,000 passenger mutations per tumor. Notice that in the cancer lines used for our experimental verifications the most frequent driver and gate-keeper mutations / alterations are indeed present (in an order of a decreasing frequency of occurrence in CRC: TP53; APC; KRAS; PTEN; SMAD4; PIK3CA; BRAF; CDH1). From a general perspective, we will encounter most often the most frequent driver mutations/alterations, however from the perspective of the individual tumor of a specific patient, we could have had a Darwinian evolution to cancer, through a constellation of (at least in part) much less frequent driver mutations/alterations. In a modern framework of personalized Systems Oncology, to know more about these individual oncogene constellations is becoming of increasing relevance. An uncommon mutation / somatically inheritable alteration could make a specific patient differently sensitive or resistant to a specific inhibitor.

A more complete analysis requires the assessment not only of DNA-mutations but also alterations in gene copy number, expression of fusion genes, direct or indirect silencing of repressor genes, epigenetic modifications and over-expression of dominant onco-genes [3-5]. At a clinical level, the sub-clonal heterogeneity present in a given tumor [6] as well as in its metastases is also important, according to the principle of Darwinian evolution of an individual tumor over time.

We are convinced that the role of normal and altered signaling-proteins cannot be understood in isolation, as a simple summation of multiple mutations/alterations. These signaling-proteins have to be integrated within pathways and network-regions [7, 8], along which biochemical signals are propagated. To reconstruct our network sub-region we implemented a MIM diagram and MIM notation rules. “A MIM is a diagram convention that is capable of unambiguous representation of networks containing multi-protein complexes, protein modifications, and enzymes that are substrates of other enzymes. This graphical representation makes it possible to view all of the many interactions in which a given molecule may be involved, and it can portray competing interactions, which are common in bio-regulatory networks” [9-12].

Integration of such large and disparate data into functional dynamic networks, capable of suggesting personalized treatment strategies, is best achieved by computational mathematical modeling.

In our work of MIM reconstruction and dynamic mathematic modeling at the biochemical interactions level, we did not consider a different scale of modeling, involving the dynamics of cell differentiation and upward movement in a colon crypt [13]. Notice however that some of the onco-proteins altered/mutated in the pathways covered by the MIM are already altered/mutated in Colon Cancer Stem Cells (CCSC) at the crypt bottom [14, 15], where molecular initial preneoplastic lesions can be passed on to daughter cells as CCSC for long times.

Similar networks are also operative in breast cancer [16], in NSCLC [17] and in many other tumors. Driver-mutations frequencies, and probably some signaling-protein concentrations, can differ significantly in different cancer types. Although these differences must be accounted for, they are not an absolute impediment to adapting the model to other cancer types.

Within current knowledge and technology limits, it is still impossible to reconstruct all pathways involved in the malignant transformation of a given cancer cell. However, some sub-regions of the signaling-network are known to be more frequently mutated/altered in specific cancers. As such, the signaling-proteins of these sub-regions have been intensively investigated and several signaling-protein inhibitors have been developed (initially for preclinical studies, but also directed toward present and future clinical studies).

**Figure 1:**
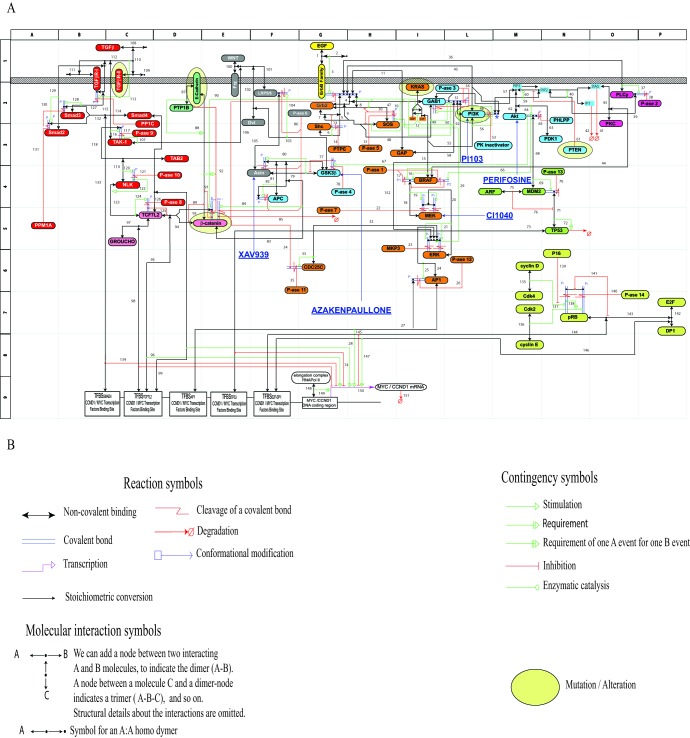
Molecular Interaction Map (MIM) referring to the pathways downstream of the TGFβ-family, WNT-family and EGF-family receptors (Panel A) The cartouches of mutated / altered signaling-proteins in the HCT116 line have been surrounded by an oval in light yellow. A parallel Figure concerning the HT29 line is reported in Supplementary Fig. 1.3. Panel B: Syntactic rules adopted for the MIM construction.

To generate a model of signaling mechanisms, we focused on a functionally relevant sub-region of the cell's signaling network (G0-G1 cell cycle transition). We modeled a network sub-region, downstream of the TGFβ, WNT and EGF pathways (Fig. 1). Driver-gatekeeper-mutations affecting the pathways reconstructed in our MIM are quite frequent in CRC (but not exclusively in CRC). They are included in the Cancer Gene Census [2, 18].

Our simulations used Ordinary Differential Equations (ODEs) involving 447 reactants (basic species, modified species, complexes and inhibitors), 348 reversible reactions and 174 catalytic reactions (for a total of 348•2 + 174 = 870 reactions). For dynamic mathematical simulations, efficient software are available [19-21] for achieving numerical solutions via ODEs of the temporal evolution of large networks of biochemical interactions, a phenomenon which conceptually is at the basis of cell life itself. A version of our Molecular Interaction Map (MIM) and associated modeling was generated in accordance with the mutational background of two CRC lines (HCT116 and HT29) and thus could predict the specific response of each line to inhibitor perturbations.

These cell lines provide a good representation of two major complementary types of CRC, in terms of accuracy of DNA replication. A strong association with Chromosomal Instability (CIN) was described for sporadic cancers, giving rise to aneuploidy. In terms of base level fidelity of DNA replication, heritable cancers were often characterized by the presence of the Microsatellites Instability (MIN) [22]. The two cancer cell lines used in our study represent distinct categories: HT29 represents the sporadic cancers group, being derived from a colon carcinoma without microsatellite instability, but featuring aneuploidy with a DI (DNA Index) ≈ 1.5 (CIN^+^, MIN^−^), while HCT116 is derived from a colon carcinoma with a high microsatellites instability level, which did not present aneuploidy using FCM-DNA ploidy analysis (CIN^−^, MIN^+^) [23].

Pathways and network sub-regions were delimited on an empirical fuzzy logic basis. They are incomplete reconstructions along an ongoing incremental process of network growth and improved refinement.

Moreover, pathways are not discrete linear modules, but intertwined parts of larger robust networks, with built-in functional feedbacks. Even if incomplete, our reconstructed and modeled network sub-region seemed sufficiently coherent in regulating (at least in part) the transcription of two key oncogenes, considered extremely relevant in the G0 – G1 cell cycle transition and S phase entry, MYC (c-myc) and CCND1 (cyclin D1).

In addition to MYC and CCND1 mRNAs, we experimentally validated the behavior of ppERK (Thr202/Tyr204)/total ERK ratio and pAKT (Ser473)/total AKT ratio proteins, which lay upstream of the mRNA response. We submitted our extensively pre-trained model to *a posteriori* experimental verifications, a strategy already used in modeling smaller signaling-network regions. In previous studies, ODEs models of the EGFR/ErbB signaling cascade were developed [24-28]. Dynamic models of signaling pathways such as TGF-β [29-31], IGFR [26, 32], Wnt [33, 34], were also developed. Repository of computational models of biological processes such as Biomodels and Java Web Simulations (JWS) [35, 36] are available on line. Statistical analyses of the degree of correlation and predictivity of these models were not reported.

We developed a MIM / dynamic model which is probably the largest ever reconstructed and modeled. We intertwined the cross-talks of three relevant cancer pathways in CRC (TGFβ, WNT and EGF pathways), instead of reconstructing them separately. Our model can be tailored to the individual mutational background of an individual tumor or cancer cell line, and is already sufficiently capable of predicting the response of CRC cells to different inhibitor treatments.

We are well aware that in order to increase predictive capabilities we have to move in the direction of even larger MIMs + dynamic models of biochemical interactions / catalytic reactions, improving and extending the parameterization of the model, plus looking at larger numbers of molecular and phenotypic outputs.

Our present work suggests that advancement in this direction is technically feasible.

Which “selective” inhibitor drugs have been already tried at a clinical level in CRC? Cetuximab and Panitumumab (anti-EGFR monoclonal antibodies) are currently used in clinical practice for the treatment of metastatic forms of colorectal cancer, in the absence of a downstream KRAS mutation conferring resistance [37-39], either as single agents or in combination with traditional cytotoxic anticancer agents. Bevacizumab, a monoclonal antibody directed against the Vascular Endothelial Growth Factor (VEGF), which promotes neo-angiogenesis, was also clinically employed [40, 41]. Regorafenib, an inhibitor of several kinases (uncertain specificity) has already been studied in a phase III trial [42], where it demonstrated some activity. The survival gains (PFS, OS) reported in these studies [40-42] on metastatic CRC and referred to patients genetically/epigenetically not deeply characterized, were in general less than four months, in comparison with other types of previous chemotherapic treatments or a placebo. Sometimes statistically significant results can depend from the size of the study. They cannot be considered by default clinically relevant.

MEK inhibitors probably more potent than CI1040 (for instance Selumetinib - AZD6244), in association with Afatinib (BIBW2992 - an ERBB2 irreversible inhibitor), can synergize in KRAS-mutant lung and colon cancers [43]. In this last case, clinical studies have just started.

In a phase II study, Everolimus (an mTOR inhibitor) was well tolerated but did not confer meaningful efficacy in heavily pretreated patients with metastatic colorectal cancer [44]. Targeted sequencing in bladder cancers, revealed that TSC1 mutations are correlated with Everolimus sensitivity [45]. A mutated TSC1 acts as an inactive GTPase toward Rheb, which in turn phosphorylates / activates mTORC1. Perhaps, a factor of caution in the usage of Everolimus, is its potent immunosuppressant activity.

The range of new-generation agents directed against specific signaling-proteins, currently evaluated in preclinical studies, is much wider than the number of drugs already approved for clinical use. At the preclinical level, several agents are being investigated whose targets are aberrantly activated in CRC, including PI3K or AKT inhibitors (along the PI3K pathway) and MDM2 inhibitors (in the TP53 pathway). In addition, new agents are emerging, such as Tankyrase inhibitors [46], which, by inhibiting Axin degradation, increase its intracellular concentration, ultimately promoting β-Catenin degradation and Wnt signaling inhibition.

## RESULTS

### Construction of a CRC Molecular Interaction Map (MIM) focused on the G0 - G1 transition

The MIMs reconstructed and used in our simulations are shown in Fig. 1 and Supplementary Fig. 1.3, for HCT116 and HT29 cancer lines, respectively. To our knowledge this network of CRC signaling (at the scale level of biochemical interactions) is probably the largest ever reconstructed and modeled.

The detailed syntactic rules for drawing our MIM are described in [9-12], and briefly summarized in panel B of Fig. 1. Our MIMs are accompanied by an Annotation List (Supplementary Table 1.1) and a Glossary (Supplementary Table 1.2). The 10 pathways involved are reported in a simplified description (Supplementary Table 1.4) indicating the signaling-proteins present in a given pathway, but without the influences or interactions (depicted instead in the MIMs of Fig. 1 and Supplementary Fig. 1.3 *+* Supplementary Table 2.1), and modeled in our mathematical dynamic modeling.

### Model construction

**Figure 2:**
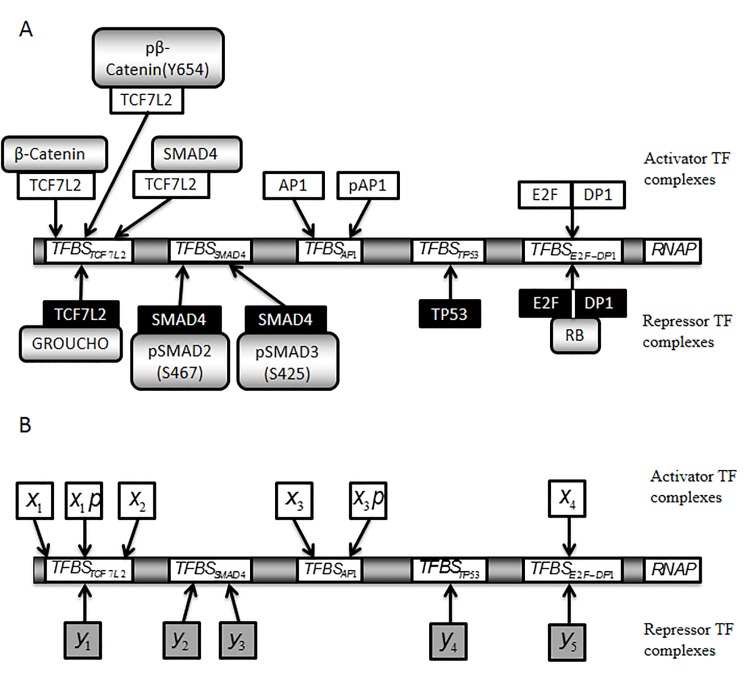
Simplified scheme of a model promoter regulating MYC and CCND1 transcription The model promoter consists of a number of important TFBSs, depicted in arbitrary order. Each TF considered to bind to the promoter is a component of the MIM: TFBS_TCF7L2_, TCF7L2 binding site; TFBS_SMAD4_, SMAD4 binding site; TFBS_AP1_, AP1 binding site; TFBS_TP53_, TP53 binding site; TFBS_E2F-DP1_, E2F-DP1 binding site. A. Schematic representation of the promoter with activators (above) and repressors (below) that are assumed to bind to each TFBS in the model. Arrows indicate the potential for binding at the TFBS. The DNA binding regions of the activators (white) and repressors (black) are also shown. B. Equivalent to A, where names of the activator (white) and repressor (grey) TF forms have been replaced by corresponding model variables. x1, β-Catenin-TCF7L2; x1p, pβ-Catenin(Y654)-TCF7L2; x2, SMAD4-TCF7L2; y1, GROUCHO-TCF7L2; y2, pSMAD2(S467)-SMAD4; y3, pSMAD3(S425)-SMAD4; x3, AP1; x3p, pAP1; y4, TP53; x4, E2F-DP1; y5, E2F-DP1-RB.

The model combines the network MIM (Fig. 1 and Supplementary Fig. 1.3) with a mathematical model of the MIM's underlying molecular interactions (Supplementary 2.1 Reactions List) and a reconstruction of the transcription region controlling the expression of c-MYC and CCND1 mRNAs (Fig. 2 and Derivation of a Transcription Rate Function for MYC and CCND1 in Methods section).

Our model can be easily modified to incorporate different molecular alterations present in different CRC lines or tumors.

It is a work in progress, but our model could already reasonably predict, substantially all the biochemical responses to inhibitor treatments that we have tested.

Supplementary Material 5.1 and 5.2 shows examples of the model complexity, including positive and negative feedbacks, going from a physiologic model to the introduction of different mutations/alterations and inhibitors.

### Experimental verification of the model

Modeling this network sub-region required a series of approximations, especially in the reconstruction of a MIM, and in parameterization of concentrations and reaction rates. It would have been difficult to determine *a priori* if a model, no matter how carefully trained to fit the current literature, was overwhelmed by the accumulated noise of all the inevitable approximations, or whether it already possessed predictive capabilities, which can be further improved in a stepwise fashion. In fact this was a crucial question our work was trying to answer.

At the core of our work, training consisted in a more than three-year long patient continuous patchwork effort, a work of continuous parameter readjustments, aimed at an integrated fitting of our model with the results of about one hundred strictly pertinent molecular reductionist-type experimental papers, published in reputable (high impact) journals.

We feel that our very long and patient empirical “sewing together patchwork”, finalized to a proper tuning of the model during the training phase, was a process somehow describable as a parallel “in silico evolution”, where deficiencies at the level of network components and parameters inputs have been somehow balanced. Let just say here that this intriguing issue could also stimulate more basic “logic” analyses about network properties.

After the training phase of our model, we submitted the model to *a posteriori* experimental verifications, to answer the basic question whether the presently available incomplete information was adequate or inadequate to build a predictive model.

If the level of information was sufficiently adequate, then we are already on a correct road, requiring only possible and achievable gradual incremental improvements, to produce models with increasing clinical utility.

We verified model predictions at both (network-pertinent) oncogene mRNA levels and protein phosphorylation levels, in response to inhibitor treatments, in HCT116 and HT29 cell lines, respectively.

### Accounting for mRNA stability in the model

We investigated directly c-myc and CCND1 mRNAs stability. Complex findings had been reported in the literature, concerning effects of MEK inhibition on CCND1 regulation, for instance as reviewed by Alao [47]. At a protein level, MEK inhibition in HCT116 cells extended the half-life of CCND1 protein by abolishing T286 phosphorylation [48], while in MCF7 cells a longer inhibition of MEK decreased CCND1 protein levels [49]. Our experiments, performed at a mRNA level, illuminated a different dimension. MEK inhibition destabilizes CCND1 mRNA, resulting in reduced protein levels in the longer term. Indeed, we also treated MCF7 cells with CI1040 and found a similar reduction in CCND1 mRNA levels (data not shown), as observed in our CRC cell lines. A CCND1 mRNA inhibition and an extended CCND1 protein half-life, could be part of dual converse-regulatory buffering mechanisms in signaling pathways, as we recently identified a similar regulation of MYCN by GSK3 [49].

In this work we were however primarily interested in c-myc and CCND1 mRNAs levels.

**Figure 3:**
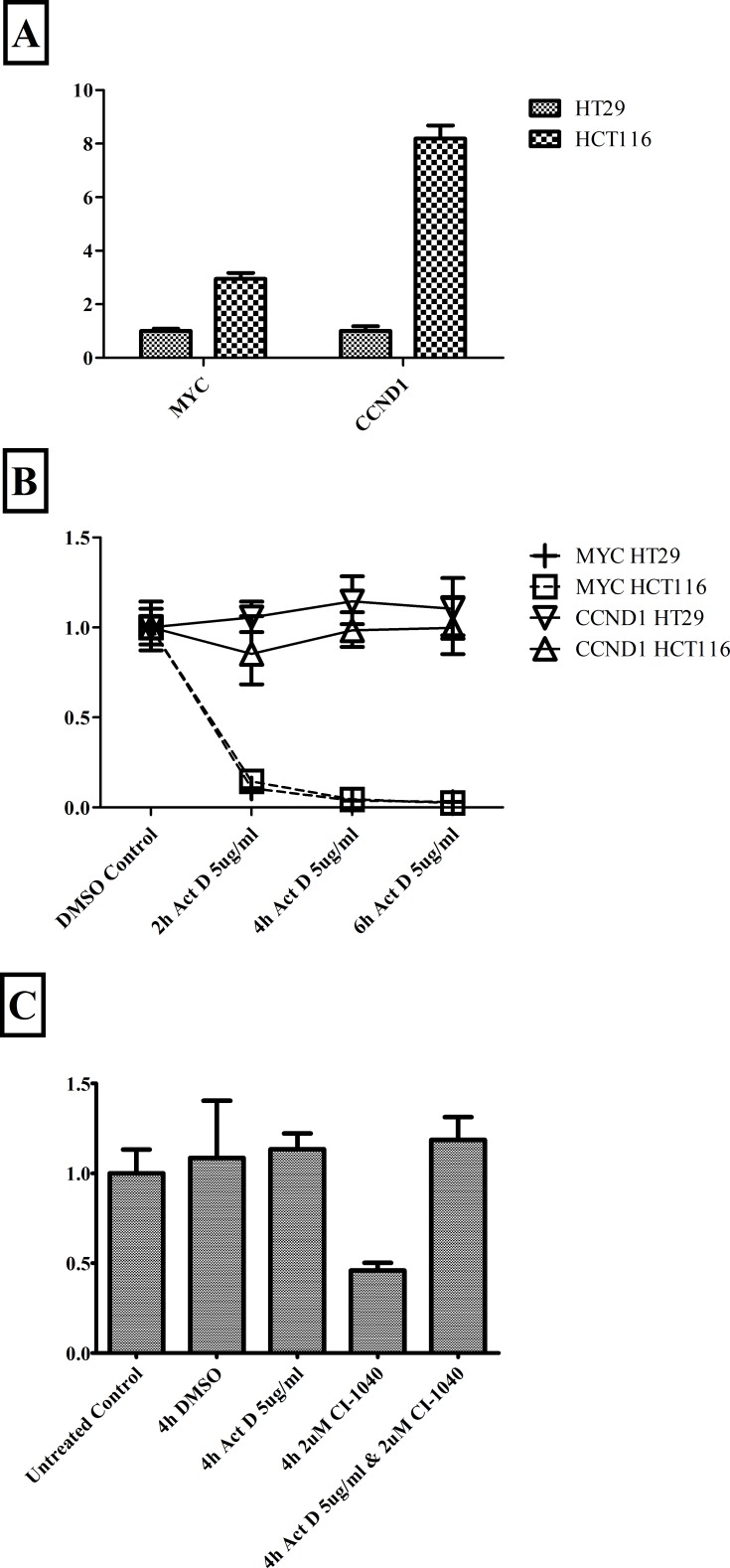
mRNA stability in HCT116 and HT29 cells Panel A: Relative expression levels of MYC and CCND1 mRNA between the cell lines. Panel B: The differing stability of MYC and CCND1 mRNAs after global inhibition of transcription with Act D; mRNA relative expression levels measured after 2h, 4h and 6h of Act D inhibition. We independently compared expression levels in HCT116 and HT29 cells relative to the DMSO control (0h) for that cell line. Notice in Panel B that graphs of MYC HCT116 and MYC HT29 are very similar and overlap. Panel C: Relative expression of CCND1 mRNA in response to the MEK inhibitor CI1040 and the pan-transcriptional inhibitor Act D, in HT29 cells. All treatments performed for 4h. Results in all panels generated by qPCR and expression normalized to endogenous control genes β-actin and PO.

During MIM experimental validation we observed a substantial difference in the response time between MYC and CCND1 mRNA to MEK inhibition (CI1040), particularly for early time points (4h and earlier). We examined the stability of each genes' mRNA using also the pan-transcriptional inhibitor Act D (Fig. 3). We determined the degradation rate of pre-existing mRNAs after blocking *de novo* transcription. While HCT116 cells have higher levels of both MYC and CCND1 mRNAs than HT29 cells (Fig. 3A), mRNAs stability is similar across both cell lines (Fig. 3B). However, MYC and CCND1 mRNAs have a different stability. MYC is highly unstable, with a half-life shorter than 2h, while CCND1 mRNA is substantially stable, being unaffected even after 6h (Fig. 3B). We incorporated into the mathematical model the differing stabilities of the two mRNA species.

Global MYC mRNA is expected to change quickly in response to an upstream inhibitor, due to rapid turnover. Conversely, as an *a priori* expectation, even after a 6h-treatment with an upstream inhibitor, we would expect to see all of the pre-existing CCND1 mRNA still present, obscuring any inhibitor-induced changes to transcription. It is however possible for an upstream inhibitor to modulate an indirect pathway capable of affecting the baseline CCND1 mRNA stability.

This behavior is probably linked to the regulation of a specific miRNA, which is capable to influence the stability of CCND1 degradation. In the literature there are references describing as miRNA-34a transcription is repressed by c-Myc protein [50]. In turn miRNA-34a is capable of targeting CCND1 mRNA for degradation [51], via an Ago complex formation [52].

**Figure 4:**
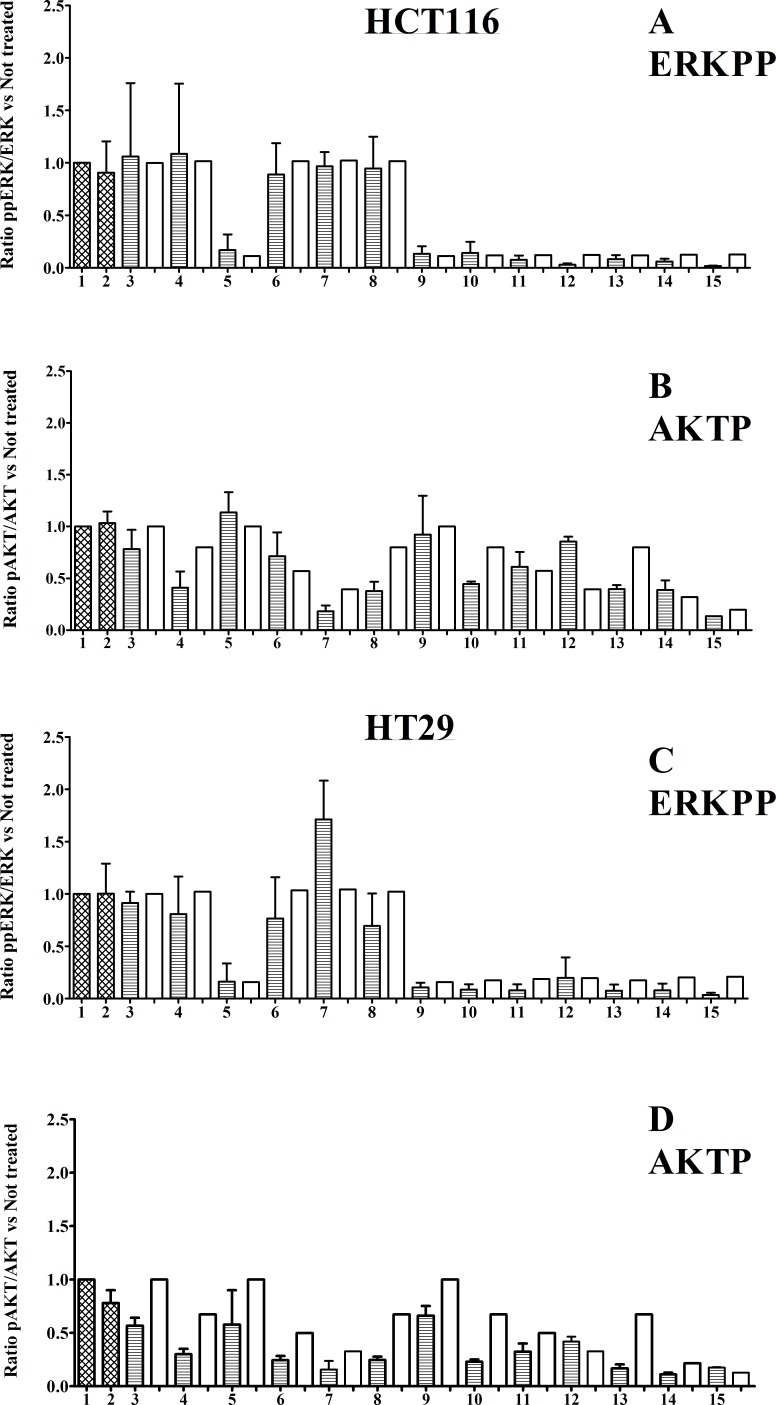
Protein levels Panels A – D histograms, referred to HCT116 and HT29 cancer lines, (30 min treatments). Panel A and C: ratio ppERK (Thr202/Tyr204) /ERK; not treated samples normalized to 1. Panel B and D: ratio pAKT (Ser473) /AKT; not treated samples normalized to 1. Untreated cells are shown in column 1 and vehicle control cells treated with only the inhibitor-solvents (Ethanol 1μl + DMSO 3μl)/(ml of medium) in column 2. The shaded columns represent experimental values, while the adjacent white columns represent simulation values. 3: XAV939; 4: PI103; 5: CI1040; 6: Perifosine 20nM; 7: Perifosine 40nM; 8: XAV939 +PI103; 9: XAV939 + CI1040; 10: PI103 + CI1040; 11: Perifosine 20nM + CI1040; 12: Perifosine 40nM + CI1040; 13: XAV939 + PI103 + CI1040; 14:XAV939 + PI103 + CI1040 + Perifosine 20nM; 15:XAV939 + PI103 + CI1040 + Perifosine 40nM

When CI1040 represses c-myc mRNA transcription by the inhibition of the KRAS pathway, c-myc mRNA and protein levels rapidly decrease, due to the high degradation rates of both molecules (our Fig. 3B and [53, 54] for c-myc mRNA, and [55] for c-Myc protein). A rapidly reduced c-Myc protein level leads to a decreased repression of miRNA-34a expression. As a consequence, more CCND1 mRNA will be degraded. After incubation with CI1040 for 4 h (Fig. 3) and 4-8 h (Fig. 6 and 7), we indeed observed progressively decreasing levels of CCND1 mRNA.

### MEK inhibition changes CCND1 mRNA stability

MEK inhibition altered the stability of pre-existing (pre-CI1040 incubation) CCND1 mRNAs through the transcriptional regulation of an intermediary gene. We revealed this by a combination treatment in HT29 cells (Fig. 3C). Consistent with the earlier experiments, Act D did not alter CCND1 mRNA levels while CI1040 reduced them. However, dual treatment with CI1040 and Act D did not alter CCND1 levels. Act D rescued the CI1040 induced CCND1 degradation. Therefore, the CCND1 mRNA destabilization induced by MEK inhibition must be achieved through the transcriptional regulation of an intermediary gene (miRNA or RNA binding protein).

We implemented in our model this novel modification of CCND1 mRNA stability in the presence of the CI1040 MEK inhibitor (Supplementary Table 2.1, reaction # 868).

### Experimental verification of the model; results related to phospho-proteins experiments

**Figure 5:**
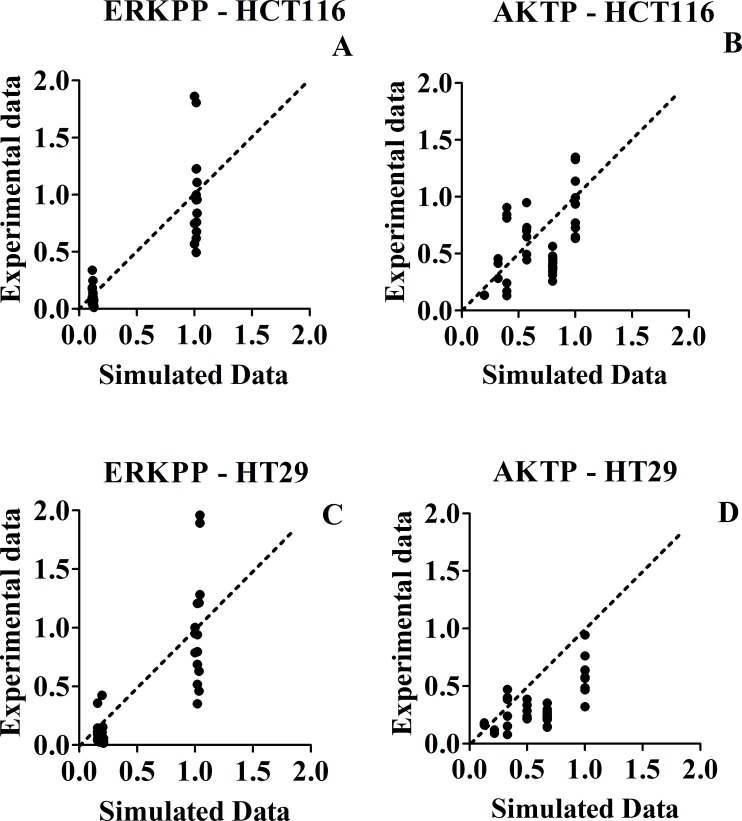
Scatter plots of experimental (Y axis) versus simulated (X axis) values, for phospho-protein levels, in response to different inhibitor treatments Panel A: HCT116 - ERKPP. Panel C: HT29 - ERKPP. Panel B: HCT116 - AKTP. Panel D: HT29 - AKTP. The (0;0) origin of the two axes makes reference to a complete inhibition of phosphorylation, both experimental and simulated.

To compare simulated and experimental data, we performed inhibitor treatments using five different inhibitors and some of their combinations, both in silico and *in vitro*, measuring the levels of pp-ERK (Thr202/Tyr204) and pAKT (Ser473) (Fig. 4 and Fig. 5). Inhibitors were chosen to produce perturbations in each of the main model pathways. Only inhibitors which acted at or downstream of mutated genes were considered. Model predictions and experimental results were statistically correlated for both lines, perhaps slightly more closely for HCT116 cells (see section “Statistical Analyses”).

In both Fig. 5 (ERKPP and AKTP proteins phosphorylation) and Fig. 7 (c-MYC and CCND1 mRNAs levels) we drew a 45° diagonal line between the X axis (simulated data) and the Y axis (experimental data), reflecting a theoretic 1:1 match. Both in the case of strong inhibitions and weak or absent inhibitions, simulated and experimental data tended to go together well.

ERKPP shows only two levels because we tested only one concentration of CI1040 MEK inhibitor (80% of inhibition), AKTP shows different levels because we tested two different concentrations of Perifosine AKT inhibitor (40% and 70% of inhibition) (Fig. 4).

### Experimental verification of the model; mRNA regulation experiments

**Figure 6:**
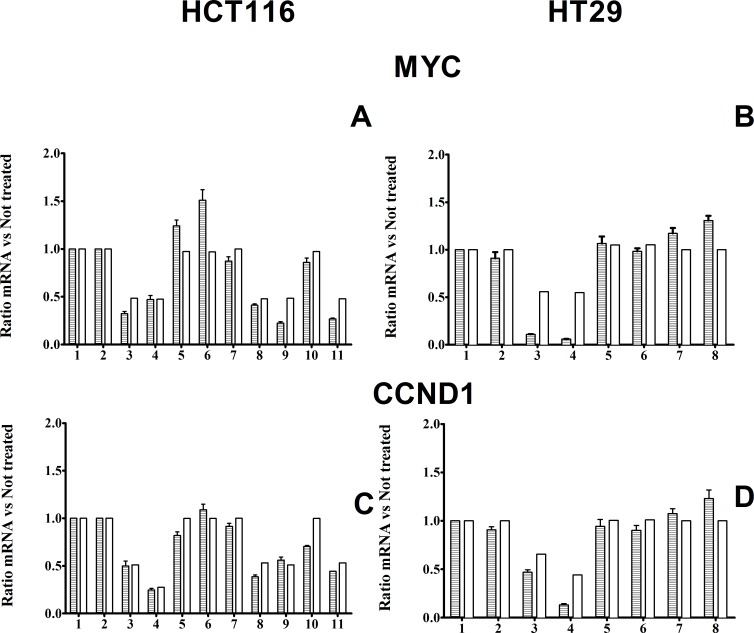
mRNA levels Panel A and B: Histograms of comparison between experimental and simulated MYC mRNA levels in HCT116 and HT29 cells respectively, in response to different inhibitor treatments. Panel C and D: Histograms of comparison between experimental and simulated CCND1 mRNA levels in HCT116 and HT29 cells respectively, in response to different inhibitor treatments. Notice that CCND1 mRNA inhibition is stronger after 8 h incubation with CI1040, than after 4 h incubation. Panel A and C: 1: 4h DMSO control; 2: 8h DMSO control; 3: 4h CI1040; 4: 8h CI1040; 5: 4h PI103; 6: 8h PI103; 7: 4h XAV939; 8: 4h PI103 + CI1040; 9: 4h XAV939 + CI1040; 10: 4h: XAV939 +PI103; 11: 4h PI103 + CI1040 + XAV939 Panel B and D: 1: 4h DMSO control; 2: 8h DMSO control; 3: 4h CI1040; 4: 8h CI1040; 5: 4h PI103; 6: 8h PI103; 7: 4h Azakenpaullone; 8: 8h Azakenpaullone.

**Figure 7:**
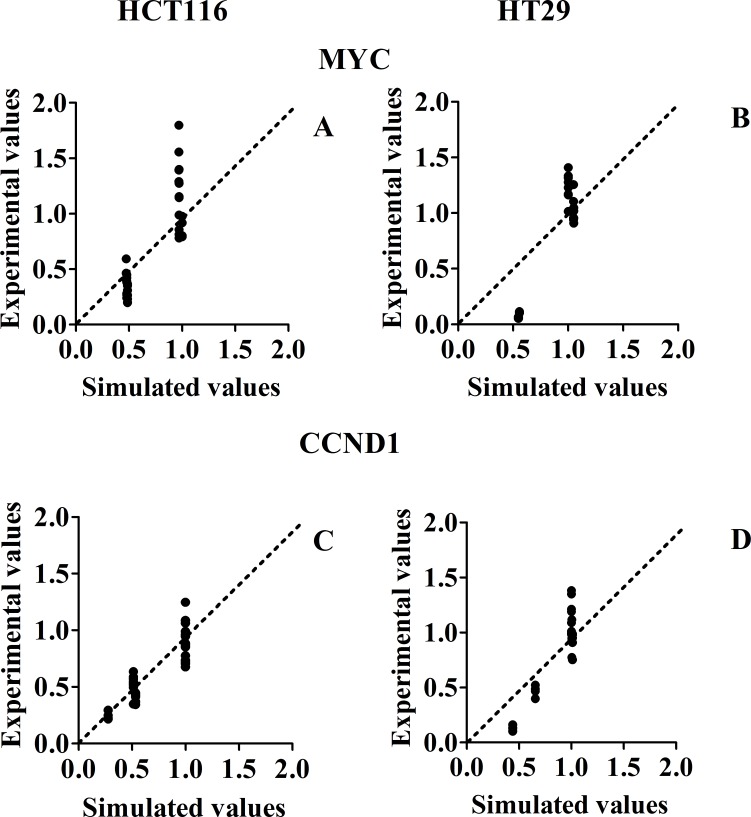
Scatter plots of experimental (Y axis) versus simulated (X axis) values, for mRNA levels, in response to different inhibitor treatments Panel A and B: Scatter plots for MYC mRNAs in HCT116 and HT29 cancer lines, respectively. Panel C and D: Scatter plots for CCND1 mRNAs in HCT116 and HT29 cancer lines, respectively. The (0;0) origin of the two axes makes reference to a complete inhibition of phosphorylation, both experimental and simulated.

To test the predictive power of our model we performed inhibitor treatments (single and combination treatments), both in silico and *in vitro*, measuring MYC and CCND1 mRNA levels and comparing simulated and experimental results (Figs. 6, 7). We selected MYC and CCND1 mRNA levels as these two genes are considered extremely relevant in the G0 – G1 cell cycle transition and S phase entry, being key factors for tumor development, growth and progression. The mRNA level of these oncogenes provides a proxy for the cellular response. The model was able to predict the changes to MYC and CCND1 mRNAs levels. For all inhibitory treatments, the trends toward strong or weak inhibitions were correctly predicted.

With reference to Fig. 7, the only concentration of MEK inhibitor tested (80% of inhibition), seems to have had a more relevant effect on mRNAs transcription than any other inhibitor treatment.

Our model captures only major and explicitly described variations induced by treatments, rather than smaller variations, probably related to additional complexities of the network not represented in our MIM. Considering the usual bench experimental variability, typical of individual data seen in biological replicates of wet experiments, we should expect much more variability in wet experiments (results parallel to the Y axis), than in the virtual predictions of the model (parallel to the X axis), where we make only one prediction for each experimental condition. This is precisely what we observed in Fig. 5 and in Fig. 7.

### Interpretation of our Results as suggested by a Statistical Analysis

Statistical analysis was implemented by means of the R statistical programming framework and the gdata, pspearman and RVAideMemoire libraries [56]. Associations were assessed through non parametric analysis with the Spearman's correlation coefficient.

Supplementary Material 6 provides raw data in four different Tables (two for each cell line): one Table for ERK and AKT proteins, one Table for c-MYC and CCND1 mRNA expression.

The data were then log-transformed to be analyzed with linear regression and the explained variation measure R^2 was provided to assess the extent of experimental variability accounted by the simulation modeling approach (see Table 1 and Table 2).

**Table 1:** Spearman's rho computed for the HTC116 and HT29 cell lines in all experimental conditions (two proteins and two mRNAs end points for each cancer line)

*HTC 116*	Spearman's rho	95% Confidence Interval	p-val [two tailed]
ERKPP	0.56	(0.28,0.73)	0.0002
AKT	0.55	(0.23,0.77)	0.0004
MYC	0.59	(0.34,0.68)	0.0002
CCND1	0.76	(0.58,0.86)	6E-07
*HT29*			
ERKPP	0.66	(0.39,0.80)	9E-06
AKT	0.61	(0.30,0.80)	5E-05
MYC	0.54	(0.03,0.84)	0.007
CCND1	0.53	(0.05,0.82)	0.009

**Table 2:** R^2 coefficient for linear regression. R^2 was computed for two proteins and two mRNAs end points for each cancer line

	R^2 (all individual experimental results were considered)
*HTC 116*	
ERKPP	0.77
AKT	0.35
MYC	0.81
CCND1	0.82
*HT29*	
ERKPP	0.75
AKT	0.49
MYC	0.96
CCND1	0.91

Table 1 reports a non parametric statistical analysis of eight correlations (with 95% confidence intervals) between our predictions through our model simulations and the corresponding experimental results. The Spearman's rho coefficient was computed for the HTC116 and HT29 cell lines for two protein endpoints and two mRNAs.

With reference to Table 2, there is no evidence of non normality in y values (Fig. 4, 5, 6 and 7), and we are not actually performing statistical inference linked to the normality assumption. Therefore, R^2 seems a suitable measure of explained variation in the present context.

R^2 represents the fraction of the Y variable that the X variable is capable to predict [57]. It can also provide a complementary indication of goodness of fit.

The two cancer lines HCT116 and HT29 carry rather distinct mutations and alterations (Section Methods). If we look at Fig. 1 and Supplementary Fig. 1.3, the two endpoints ERKPP and AKTP look as two rather distinct pathways, with an altered behavior dependent from distinct mutations. If we look at Fig. 2 and Fig. 3, it seems evident that the two mRNAs add an additional layer of complex regulation, downstream of activators or repressors bound to the transcription factor binding sites (TFBS).

As a consequence, the different parameters measured can be considered at least partially independent, even if “physically” not completely independent [58]. We found a significant statistical correlation in all cases examined. If we have a partial independence for each case examined, an “always positive outcome” will tend to be much more significant than each individual correlation.

Our statistical analysis has tried not to convey a misleading message of our stage of MIM reconstruction, parameterization, and mathematical modeling, as if it was already close to a practically working instrument.

What we have shown instead, is that the multiple (and inevitable at this stage) deficiencies in input, are definitely not generating a noise obscuring a clear connection between model and experiments.

The very important message (reinforced by statistical analysis) is that an incremental path is now open, justifying more long term future cooperative efforts in the directions of building larger MIMs, supported by more input parameters at the level of molecular concentrations and reaction rates.

In our opinion, our present model is already intriguingly quite suggestive and encouraging, but we work toward future more advanced models, as operative instruments for a rationalization of the treatment of individual cancer patients, using associations of inhibitors of specifically altered pathways in a given specific cancer of a specific patient.

### Our model applied to independently published results

The predictions of our model were tested against preclinical results obtained by independent investigators, in DiFi, LIM1215, HCA-46 and OXCO-2 CRC lines, before and after induction of panErb resistance through a subsequent KRAS mutation. Well after our model finalization (training, + mRNA stability adjustments), and our own experimental verifications, in a very recent paper by Misale et al. [59], these authors demonstrated that, in CRC lines that had become resistant to panErb inhibitors (because of the late appearance of a mutated KRAS), the addition of MEK inhibitors could partially overcome resistance. This re-sensitization was more complete when a MEK inhibitor was given together with a panErb inhibitor. These lines were initially sensitive to the panErb inhibitors cetuximab or panitumab, but resistance emerged through subsequent new KRAS mutations. We examined in our model the simulated behavior before and after the emergence of a resistance to panErb inhibitors. To do so we generated personalized MIM modeling, in the absence or presence of KRAS alterations. We simulated the presence of a panErb inhibitor, a MEK inhibitor, or both. The behavior of P-protein/total protein for EGFR, ERK, AKT (at 30 min - 1 hour) was well compatible with the authors' observations (Supplementary Material 5.2). Moreover, both c-MYC and CCND1 mRNAs (both playing a crucial role for cell replication), were completely normalized (at 4 - 8h), only by the combination of panErb and MEK inhibitors in the presence of a mutated KRAS (Supplementary Material 5.2). Our simulations suggest that this behavior is due to a synergic effect of the two inhibitors, which target two different pathways: MEK inhibitor downstream of the KRAS mutated pathway, and PanErb inhibitor on the PI3K-AKT pathway.

This synergism seems in line with that observed by the authors at a cellular level. The behavior of our model in this new context, being able to satisfactorily reflect these independent and subsequent experimental findings, was quite encouraging.

## DISCUSSION

We started from a signaling-network model without cancer mutations (a “physiologic model”) and generated models including specific mutations/alterations (in our MIM region), concordant with those present in the HCT116 and HT29 colon cancer lines. We demonstrated that this model can be tuned with the individual genetic background of a tumor (using cell lines as a proxy), to predict individual responses to inhibitor treatments. This is an encouraging finding, suggesting that larger similar models using a similar conceptual framework, generated by larger cooperative groups (perhaps consortia-like), could achieve important incremental advances, culminating in a potentially useful new tool for clinical oncology, tailored toward predicting in individual patients the effectiveness of associations-combinations of selective inhibitors of distinctly altered pathways. Tentatively, we would expect that this goal could be achieved in three - five years.

When a signaling-protein is mutated/altered in an appropriate way, it can confer auto-activation, switching on the downstream part of a given pathway independently of the upstream condition. This potential for an irreversible pathway activation, likely drives the selection of an oncogenic mutation during the evolutionary carcinogenetic process (including the mutations/alterations incorporated into our model). A good therapeutic strategy could be to inhibit two or three different pathways simultaneously, but only those pathways altered by driver mutations and implying potential forms of addiction to the mutations/alterations considered [60].

As a first approximation, we should inhibit each altered pathway with drugs acting at or below the pathway-mutation/alteration.

Complementary to the strategy we propose will have to be a more widespread generation of pertinent personalized patient data, for instance through new generation sequencing approaches applied to a routine investigation of the most frequent driver mutations and somatically inheritable alterations, reported for a given tumor type [2].

We have moved in the direction of an important enlargement and progressive improvement of previous modelings of biochemical interactions [24-27, 61-63]. In perspective, for the specific cancer of a given patient, our MIM and its linked mathematical model, aims at a translation of a restricted number of relevant mutation/alteration patient data + selected inhibitor combinations, to clinically actionable information.

In our opinion, the results reported here prove the concept of using mathematical models to better understand the intricacies of biological signaling-networks and treatment responses. Most importantly, our work suggests the feasibility of this approach and the great opportunities offered by continued future incremental advances. Progressively/incrementally amending a model with novel information, should be a working and winning strategy for improving the predictive power of models of this kind. We have in mind not only new externally published information: a strong consortium-like cooperation could become capable of acquiring directly a much larger set of required experimental parameters (both in terms of molecular concentrations and reaction rates).

It was impossible to assess *a priori* the predictive value of our model. More than obviously it could not be a definitive and complete reconstruction of a network region, despite being based on a rather large number of directly pertinent publications (about one hundred). The framework and details of our simulation-engine, and its specific parameters, were all established during the training phase. Only after completion of the training phase and its satisfactory behavior in dealing with the input data, we moved to the verification phase, where we compared the predictions of our simulation-engine with our *ex novo* experimental results. Using a different phrasing, retrofitting procedures were continuous and systematic during the training phase, but strictly confined only to that phase.

As described in the statistical section, both correlation and goodness of fit between simulations and *ex novo* experiments, have been quite encouraging. It seems therefore really worthwhile to move ahead and to build larger improved models. Computer power is continuously increasing and should not be a problem for the implementation of larger models.

Our model was also successfully tested against the results of a novel publication [59], available several months after the conclusion of our model training phase, and also after the conclusion of our wet experiments. By constructing personalized MIMs adapted to panErb sensitive or resistant four CRC lines, once again our model proved to be predictive of the experimental response (last paragraph of Results section and Supplementary Material 5.2). Supplementary Material 5.2 refers specifically to the work of Misale et al [59].

Our MIMs have specific properties in terms of a Graph Theory perspective [64, 65]. We have preliminary evidence that these MIMs, and their dynamic modeling, at this stage of development, can withstand a significant degree of parameter approximation. They can provide an important tool to collating the increasingly complex data on oncogenic signaling and translating it into a program that can be easily interrogated, enhancing human understanding of highly non-linear biological signaling networks. These biochemical-interactions networks, over a given size, are clearly beyond unaided human capabilities of comprehension of what is going on [66].

At the same time this is *not an all or nothing process*, as we do not have to know every detail to reach a more advanced understanding. It seems therefore worth moving forward in this direction, as it may have important effects on a more rational cancer therapy when treating patients with signaling-protein inhibitors. This not only in terms of one-drug therapies, but, in terms of appropriately tailoring drug combinations to individual patient needs: an important step in a progress towards a really innovative type of personalized cancer therapy.

In the near future, more advanced patient specific tumor data, for instance, from deep sequencing of tumor biopsies or liquid biopsies (focused on major driver mutations in a given tumor + some most relevant additional somatically inheritable driver alterations), could be routinely incorporated into a model like ours, to create patient personalized tumor models, capable of supporting clinical treatment decisions. Such models could also potentially inform about the likelihood of some types of acquired drug resistance evolving after an initial sensitivity to a given inhibitor. Simulating different types of perturbation, we could investigate which mutated nodes could circumvent a drug's downstream inhibitory effect.

Our dynamic models could represent an approach complementary to an interesting approach proposed in a recent paper by Crystal and colleagues [67]. They have combined a genetic analysis of acquired-resistance tumors with a pharmacologic screening with targeted agents, to predict the behavior of an association of inhibitors. They used a set of 76 antineoplastic drugs, mostly inhibitors of signaling-proteins (cancer-genes). Their approach was successful, but required an important amount of experimental work to find a working association, in terms of cell growth inhibition. A modeling approach could help to detect priorities and restrict the number of proposed associations, to be finally tested / validated at a cellular level.

Such modeling approaches, which collate the continuously accumulating disparate data from basic biological research into integrated predictive user-friendly models with clinical utility, should fulfill in the cancer field the broad promise of Systems Medicine: the translation of Systems research into Clinical tools [68]. Progress in this direction will be gradually accompanied by a better understanding and acceptance by clinicians.

Extended MIM dynamic models could also be utilized to inform new clinical trials (of both novel compounds and combination therapies) potentially improving the failure rate observed in such trials.

In a recent example Erlotinib (an EGFR inhibitor) and anti-MET onartuzumab would not synergize effectively in the majority of NSCLC patients in a phase III study [69]. This lack of synergism could have been the consequence of multiple mutations/alterations commonly found in the network downstream of EGFR and MET. Perhaps their role could have been understood in advance in the framework of a dynamic modeling. It could be possible to consider the administration of some additional downstream inhibitor. A complementary deeper molecular characterization of each individual cancer could focus combination treatments towards subsets of patients more homogeneous, characterized at a network-pathology level.

In conclusion, we feel that this is a road to a novel and important approach with utility to oncology and medicine more generally. We foresee that second generation descendents of this modeling approach will be progressively chosen more frequently to interpret preclinical and clinical research into onco-protein mutations and to suggest rational combinations of inhibitors. We consider this perspective a better alternative to the more reductionist current approach, of considering substantially a summation of biomarkers, to be matched with a given therapeutic hypothesis.

Signaling-network dynamic simulations are probably an essential tool on the road of a more adequate dealing with the high degree of molecular complexity of cancer and cancer therapy.

## METHODS

### Simulations using ODEs

For dynamic simulations we formulated the reactions scheme as Ordinary Differential Equations (ODEs) according to Supplementary Table 2.1, in terms of the reactions' kinetic laws [70]. The ODE models were developed and simulated with the SimBiology toolbox of the Matlab software (Mathworks) [20].

We performed our simulations for mRNA levels at 4 – 8 h. As changes in protein phosphorylation above transcription (Fig. 1 and Supplementary Fig. 1.3) take place much more rapidly, they were experimentally assessed at 30 min (and 60 min with substantially similar results).

We assigned initially a total concentration (relative to a given basic-protein plus all its complexes and post-translational modifications) entirely to the unbound basic protein (Supplementary Table 2.2). The presence of mutations/alterations and/or inhibitors, according to the cell line and experimental conditions being simulated, were introduced to the model at this early stage. Reactions were then brought to a quasi-stationary equilibrium, which causes redistribution of each basic protein among all its forms and complexes with binding partners (SimBiology numerical approximate solution as a function of time).

### Thermo-statistical derivation of a transcription rate function for MYC and CCND1

This is a quick guide to main equations and assumptions, for more details see also the pertinent Ss.

The first step in building the thermo-statistical model involved identifying key transcription factor binding sites (TFBSs) responsible for MYC and CCND1 activation and repression, as well as the main TFs that bind to them. We have considered only some of the most important and best studied TFs (see reviews on MYC transcription (1, 2 in Supplementary Fig. 4.1), CCND1 transcription (3, 4 in Supplementary Fig. 4.1).

The transcription rate function of a gene describes the relationship between the gene's rate of transcription and the cellular concentrations of upstream transcriptional activator and repressor complexes. A single (as a first-approximation) transcription rate function for the MYC and CCND1 genes was mathematically derived using a statistical thermodynamic framework [71-73]. First, the key transcription factor binding sites (TFBSs) responsible for activation or repression of MYC and CCND1 were identified, as well as the main transcription factors (TFs) that bind to them (Fig. 2 and Supplementary Material 4.2). Each TF identified was included in the MIM. As the two genes were found to have similar key transcriptional regulators, it was assumed that they have equivalent promoter regions and the same transcription rates. We observed however important experimental differences in the degradation rates of the two mRNAs. In our model we accounted also for these differences.

The entire collection of all five TFBSs, all activators and repressors considered in the MIM, RNA Polymerase II (RNAP) and the RNAP binding site was denoted in the model by *Promoter/TF/RNAP* (Supplementary Fig. 4.1). Similarly, each individual TFBS and its associated TFs, when paired with RNAP and the RNAP binding site, formed a theoretical reduced promoter region termed *TFBS/TF/RNAP*. Standard statistical thermodynamic assumptions and procedures were applied in deriving the transcription rate function, with some extensions to the original method to encompass the complexity of the promoter under consideration (Supplementary Material 4.3).

The probability of RNAP binding to the promoter can be written in terms of the regulation factor $F_{reg}$ associated with the promoter, a function of TF concentrations parameterized by TF-DNA dissociation constants (Supplementary Material 4.3):

(a) $$p(RNAP_{bound})\;=\;\frac{1}{1+{\left(\frac{\left[RNAP\right]}{k_{RNAP}}F_{reg}\right)}^{-1}}$$

It was shown that under certain conditions (i.e. independence of TFBSs) the regulation factor of *Promoter/TF/RNAP* can be written as the product of the regulation factors over all its constituent *TFBS/TF/RNAP* parts (Supplementary Material 4.4), i.e.

(b) $$F_{promoter/TF/RNAP}\;=\;F_{TCF7L2}\;*\;F_{SMAD4}\;*\;F_{AP1}\;*\;F_{TP53}\;*\;F_{E2F\;\cdot \;DP1}$$

The mRNA production rate was assumed to be proportional to the probability of RNAP binding to the promoter for transcription initiation [72,73]. Therefore, for each *TFBS/TF/RNAP* a regulation factor was derived (Supplementary Tables 4.7 - 4.12) and a final expression for the transcription rates of MYC and CCND1 was obtained (Supplementary Material 4.6),

(c) $$\frac{d}{dt}mRNA\;=\;k_{synth}\;*\;P(RNAP_{bound})\;-\;k_{\deg}mRNA$$

$P(RNAP_{bound})$ was obtained by Eqn. (a) and thermo-statistical methods (Supplementary Materials 4).

The parameter k_synth_ was assigned the value of 5•10^−5^/sec both for the synthesis of MYC and CCND1. The parameter k_deg_ was assigned values of 2.7•10^−4^/sec or 5.7•10^−6^/sec, for degradation of MYC or CCND1 respectively, in the absence of any inhibitor (Fig. 3B and Supplementary Table 2.1, where we report Eqns 863, 864, 867, 868).

The degradation rate k_deg_ for CCND1 mRNA was modified according to our experimental observation of decreased mRNA stability in the presence of MEK inhibitor CI1040 (Results section: Fig. 3C and Supplementary Table 2.1).

In our model, the velocity of degradation of a reactant was described as the product of the reactant concentration with its degradation rate (Eqs. 747, 748, 770, 775, 782, 864).

Considering the effect of MEK inhibitor on the stability of CCND1 mRNA, observed experimentally in Fig. 3C, CCND1 mRNA degradation rate (k_degCCND1_) was empirically adjusted, in the presence of MEK inhibitor, as:

(d) $$k_{\deg CCND1+MEKinhib}\;=\;K_{\deg CCND1contr}\;\cdot \;[ERKPP_{control}/ERKPP_{MEKinhib}]$$

(*related to* Eqn 868 reported in Supplementary Table 2.1).

MEK inhibitor CI1040 decreases [ERKPP] levels, therefore, in the presence of the inhibitor, [ERKPP_control_/ERKPP_MEK inhib_] > 1. The empirical formula implemented in Eqn 868 increases k_degCCND1_ with an inverse proportionality in respect to the degree of ERK phosphorylation.

### Experimental derivation of MYC and CCND1 mRNA degradation rates

To evaluate MYC and CCND1 mRNA (qPCR) stability in HCT116 and HT29 cell lines, we performed RT-qPCR (see below) after treatment with the pan-transcriptional inhibitor Actinomycin D (Act D) (Sigma-Aldrich) at 5 μg/ml. Our results (see Results section) demonstrate the very different mRNA stability of MYC and CCND1 and highlight the importance of including stability/degradation rates in any MIM/mathematical modeling.

### Mutations/Alterations in our CRC lines virtual implementation

The mutations present in our ATCC CRC lines are described in CCLE database [74]. Four dominant mutations in terms of pathway activation (KRAS (HCT116), β-Catenin (HCT116), BRAF (HT29), PI3K (both lines) have been implemented according to the simplifying rule that the activated protein will remain in a phosphorylated or in an “active” form (de-phosphorylation/“inactivation” was prevented in the biochemical dynamical implementation of the model). According to our experimental results reported in Supplementary Material 3, HT29 cells over-expressed ErbB2 ~ 2x, and this variation was also simulated in our modeling. To model the five alterations involving the loss of function, resulting in global pathway activation (PTEN (HCT116), E-Cadherin (HCT116), TGFβ receptor II (HCT116), APC (HT29), SMAD4 (HT29)), we put zero concentrations, as simulating the absence of a functional protein. In the case of PTEN, we input its concentration at 60% of its physiological value, according to our experimental results in HCT116 cells (Supplementary Material 3). We could also simulate the presence of a single functional allele (data not reported). In the case of hyper-expressed onco-proteins, e.g. ErbB2, its concentration was raised 2x in HT29 cells (Supplementary Material 3).

### Implementation of our virtual inhibitors

As reported in Table *SM2.1*, we applied the following inhibitors: MEKPP inhibitor *CI1040*, PI3K inhibitor *PI103*, AKTP inhibitor *Perifosine*, GSK3β inhibitor *Azakenpaullone*, Tankyrase inhibitor *XAV939* (XAV939 stimulates β-catenin degradation by stabilizing Axin, which is part of the [Axin:GSKβ:APC] degradation complex for β-catenin).

According to the information given by the company selling the inhibitor (see below), and to the concentration of inhibitor used, we calculated different inhibition levels for each inhibitor (Supplementary Material 3.1)

### Cell culture and reagents

The HCT116 and HT29 CRC cell lines were obtained from the American Type Culture Collection (ATCC). For details about cell culture conditions and reagents, Supplementary Material 3.1.

### RT-qPCR experiments

We treated our CRC lines with an individual inhibitor, inhibitor combinations, vehicle only, or no treatment, for the durations indicated below. At the end of treatment, we extracted total RNA from the cells using an RNeasy Kit (Qiagen). Total RNA was used to synthesize cDNA using QuantiTect Reverse Transcription Kit (Qiagen), including a genomic DNA digestion step. We generated biological duplicates for all samples at all time-points, and performed technical replicates for every sample and time-point. We performed reverse transcription qPCR on an ABI 7900HT Real Time qPCR System (Applied Biosystems) with TaqMan reagents (Applied Biosystems) according to the manufacturer's recommended protocol. Gene expression was normalized to the expression of β-actin (Assay I.D: 4326315E), with P0 ribosomal protein (Assay I.D: 4310879E) as a second control. MYC and CCND1 expression levels were detected with assays Hs00905030_m1* and Hs00765553_m1* respectively.

### Western blot experiments

Supplementary Material 3.2 provides methodological details (antibodies used, protein which an antibody binds, supplier and catalog number, concentration they were used at, and incubation time) as well as information about preliminary experiments performed to determine optimal and standard experimental conditions. After these preliminary experiments, all the combinations of different inhibitors were examined at 30 min incubation, after an initial change with complete fresh medium. These results are reported in the Results section and have been correlated with the modeling simulations (Supplementary Material 3.2).

## SUPPLEMENTARY MATERIALS, FIGURES AND TABLES


