# Supplementary material for: Advances in dynamic modeling of colorectal cancer signaling-network regions, a path toward targeted therapies
Source: Oncotarget. 2014 Dec 31;6(7):5041–58. doi: 10.18632/oncotarget.3238 (PMC4467132; doi:10.18632/oncotarget.3238)
Supplement: Supplementary file 1 [file oncotarget-06-5041-s001.pdf]

Supplementary Materials

Supplementary Material 1.1 Annotation List

Supplementary Table 1.2 Glossary

Supplementary Fig. 1.3 – Molecular Interaction Map (MIM) referred to HT29

Supplementary Table 1.4 - Pathways present in our MIMs

Supplementary Table 2.1 - Reaction list

Supplementary Table 2.2 - Species Initial Concentration

Supplementary Material 3.1 Cell cultures and reagents

Supplementary Material 3.2 Western Blots: Methods and raw results

Supplementary Fig. 4.1 Illustration of the transcription factors and transcription factor binding sites considered in the Promoter/TF/RNAP and TFBS/TF/RNAP systems.

Supplementary Material 4.2 Literature background describing the involvement of key transcription factors and transcription factor binding sites in regulation of MYC and CCND1 transcription

Supplementary Material 4.3 Derivation of the transcription rate function for MYC and CCND1

Supplementary Material 4.4 Derivation of the regulation factor $\mathbf{F_{Promoter/TF/RNAP}}$ in terms of the regulation factors of independent TFBS/TF/RNAP subsystems

Supplementary Material 4.5 Example: Detailed derivation of the regulation factor $\mathbf{F_{E2F-DP1}}$ for the TFBS/TF/RNAP subsystem associated with the E2F-DP1 transcription factor binding site

Supplementary Material 4.6 Final expression for the transcription rate of MYC and CCND1

Supplementary Table 4.7 Computation of regulation factor $\mathbf{F_{E2F-DP1}}$

Supplementary Table 4.8 Computation of regulation factor $\mathbf{F_{TCF7L2}}$

Supplementary Table 4.9 Computation of regulation factor $\mathbf{F_{SMAD4}}$

Supplementary Table 4.10 Computation of regulation factor $\mathbf{F_{AP1}}$

Supplementary Table 4.11 Computation of regulation factor $\mathbf{F_{TP53}}$

Supplementary Table 4.12 Summary of regulation factor expressions

Supplementary Material 5.1 Examples of behaviors of the model

Supplementary Material 5.2 Starting from physiologic model: no mutations, KRAS mutation. Inhibitors: no inhibitors, panErb inhibitor, MEK inhibitor, both inhibitors

Supplementary Material 6 Simulated and Experimental Data

Supplementary Table 6.1 ERKPP and AKTP levels in HCT116 cell line

Supplementary Table 6.2 ERKPP and AKTP levels in HT29 cell line

Supplementary Table 6.3 MYC and CCND1 mRNA levels HCT116 cell line

Supplementary Table 6.4 MYC and CCND1 mRNA levels HT29 cell line

Supplementary Material 1.1 - Annotation List referred to the MIM of Fig.1 and Supplementary Fig. *1.3*

Supplementary Material 1.1 *shows an Annotation List of the reference sources of the interactions reconstructed in our MIMs. In order to keep the reference list as short and relevant as possible, recent articles or review articles were often cited that contained references to the original works. Whenever possible these sources of biochemical interactions where also utilized for preliminary extrapolations of kinetic parameters to reconstruct a reasonable global dynamic behavior. Additional information contained in publications were utilized. Corresponding numbers appear in Fig. 1- main text,* Supplementary *Fig.* 1.3 *and in the Annotation List.*

(# followed by an Arabic number indicates an interaction or a contingency depicted in the MIM)

#1-3, coord. 1-2, G: EGF (EGF family proteins) binds the extracellular domain of ErbB family receptors (#1); the EGF:ErbB family receptors complex then forms a dimer (homo or hetero-dimer) (#2) which induces reciprocal multiple tyrosine autophosphorylations (#3) in the intracellular portion of the receptors, and provides specific docking sites for cytoplasmatic proteins [1-3]. <u>In this MIM</u>, we represented only one site of autophosphorylation.

#4-9, coord.2-3, G-H: Following activation of the ErbB family kinases and autophosphorylation, the adaptor protein Grb2 can bind either directly (#4) [4] or through tyrosyl-phosphorylated Shc (#5,#6, #7), to specific docking sites on the receptor [5]. The complex of the ErbB family receptors with the adaptor protein Grb2 and /or ShcP:Grb2 complex (#9), can bind SOS and is then recruited to the plasma membrane [6]. The Grb2:SOS complex can bind the ErbB family receptors activated (#8), directly.

#10, coord. 2-3, H-I: SOS can act on membrane-bound KRAS and is then able to increase KRAS-GTP levels by catalyzing nucleotide exchange on KRAS, resulting in KRAS activation (KRAS-GTP) (#10) [6, 7].

#11-12, coord. 1-3, G-H: Association of GAP with receptors tyrosine kinase phosphorylated (#11) induces GAP protein binding to KRAS-GTP and accelerates the conversion of KRAS-GTP to KRAS-GDP (#12), which terminates signaling [7, 8].

#13-14, coord. 2-3, G: Phosphorylation of Shc (#13) decreases the affinity of the protein for the EGFR activated receptor: Shc dissociated from the receptor does not contribute to RAS activation [9]. Shc:P can be dephosphorylated by cellular protein tyrosine phosphatase (PTPases): in this MIM, it was assumed that Shc:P can be dephosphorylated by PTPε phosphatase (#14) [10].

#15-17, coord. 2-4, H-I: KRAS:GTP binds to BRAF (#15) and recruits BRAF from the cytosol to the cell membrane, where BRAF activation takes place. BRAF activation (#16) is regulated by an interplay of complex and still incompletely understood mechanisms [11-14]. In this MIM, it was assumed that BRAF-P1 can be dephosphorylated by an unknown phosphatase, P-ase 1 (#17).

#18-23, coord. 4-6, G-I: Activated BRAF can phosphorylate and activate MEK (#18, #19), which in turn phosphorylates (#20, #21) and activates extracellular-signal-regulated kinase (ERK). In this MIM, it was assumed that an unknown phosphatase P-ase 7 dephosphorylates MEK-P and MEK-PP (#22); MKP3 phosphatase dephosphorylates ERK-P and ERK-PP (#23). It was reported that inactivation of ERK-PP could be carried out by multiple phosphatases [11-13].

#24-28, coord. 6-9, D-L: ERK-PP translocates to the nucleus and can phosphorylate Elk1 transcription factor (not shown in this MIM) [15], leading to elevated AP-1 activity via c-fos induction. AP1 complexes, depending on their composition, have been shown to activate or repress CCND1, and their activity can be modulated by phosphorylation [16, 17]. In the model, we assume a net-positive effect of AP1 on CCND1 transcription, which has a consensus AP1 ($TFBS_{AP1}$) site in its promoter (#27), [17-20] and on c-MYC transcription (#28) [21-23]. In this MIM, it was assumed that ERK-PP can bind (#24) and phosphorylate a pre-existing AP-1 transcription factor (#25) and not newly synthesized AP-1 components or AP-1 complexes. In this MIM, it was assumed that an unknown phosphatase (P-ase 11) dephosphorylates AP-1-P (#26).

#29-31, coord. 2-6, H-L: Feedback inhibition from ERK-PP to SOS provides an additional mechanism for the inhibition of KRAS signaling: SOS phosphorylation by ERK-PP (#29) causes the dissociation of SOS from its complexes with Grb2 (#30). SOS-P can't bind KRAS [11, 24, 25]. In this MIM, it was assumed that an unknown phosphatase (P-ase 5) dephosphorylate SOS-P (#31).

#32-35, coord. 2-6, F-I: Activated ERK kinase can bind (#32) and phosphorylate CDC25C on several residues (#33), leading to activation of its phosphatase activity [26]. Activated CDC25C can dephosphorylate EGFR receptor (#34). An activating KRAS mutation or a BRAF (V600F) constitutive activation in mutant colon cancer tumors [27] causes a feedback dephosphorylation of EGFR-P. In this MIM, it was assumed that an unknown phosphatase (P-ase 11) dephosphorylates CDC25C-P (#35).

#36-44, coord. 1-4, G-P: Binding of PLCγ to activated ErbB family receptors (#36) results in the phosphorylation of PLCγ (#37), [1]. In this MIM, it was assumed that PLCγ:P can be dephosphorylated by an unknown phosphatase P-ase 2 (#38). Active PLCγ binds and cleaves (#39, #40) the PtdIns(4,5)-P2 to yield the second messengers 1,2-diacylglycerol (DAG) that stays in the plasma membrane and the soluble inositol 1,3,5-trisphosphate (IP3) that induces a Ca2+ release from the endoplasmic reticulum (not shown in this MIM). DAG (#41) and IP3 (#42) are degraded. The increased concentration of diacylglycerol (DAG) can activate, alone and in combination with calcium, various PKC isoforms (#43) [28, 29]. An activated PKC can phosphorylate and activate BRAF (#44) [30].

#45-54, coord. 1-3, G-L: Upon growth factor stimulation, receptors tyrosine kinase phosphorylated can activate PI3K via two major mechanisms: direct binding (#45,#46) [31, 32] or indirect via GAB1 [25]. The association of GAB1 with ErbB family receptors is thought to occur via Grb2 (#47) or ShP:Grb2 (#48), resulting in tyrosine phosphorylation of GAB1 (#49,#50); GAB1-P binds (#51) and activates PI3K and recruits them to the plasma membrane (#52) [33]. In this MIM, we assumed that a PK inactivator protein (#53) inactivates PI3K and unknown phosphatase P-ase 3 dephosphorylates GAB1-P (#54).

#55-56, coord.2-3, I-L: RasGTP can bind (#55) and activate (#56) PI3K [34].

#57-58, coord. 1-3, H-M: GAB1 and GAB1-P can bind PIP3 and lead to a further recruitment of GAB1 (#57) to the membrane [33], and activate PI3K pathway. In addition, the tyrosyl-phosphorylated GAB1 can bind GAP (#58) [33]. This association induces GAP protein binding to KRAS-GTP and accelerates the conversion of KRAS-GTP to KRAS-GDP (#12), and negatively regulates KRAS [7, 8, 33]. Negative feedback regulation of GAB can be achieved by serine phosphorylation by ERKPP (#152) [35, 36].

#59-6 , coord. 2-3, I-N: Activated PI3K binds PIP2 (#59) and catalyzes the formation of PIP3 from PIP2, on the plasma membrane (#60). PTEN directly antagonizes PI3K signaling by dephosphorylating the 3-position of the inositol ring of PIP3 (#61) and thus inactivating downstream signaling [31-33].

#62-66, coord. 2-4, I-N: PIP3 directly binds to Akt (#62) and recruites Akt to the plasma membrane; PDK1 kinase phosphorylates and activates the Akt-PIP3 complex (#63). The phosphorylation of Akt at Thr 308 by PDK1 (#64) results in activation of this protein kinase; additional phosphorylation of Akt at Ser 473 is required for its full activation [37] (not shown in this MIM). PHLPP dephosphorylates Akt-P on Ser473, resulting in inhibition of its kinase activity (#65) [38]. The active phospho-Akt inhibits the activation of BRAF, by hyperphosphorylation on Ser-259 (BRAF-P2)(#66), [39].

#67-76, coord. 2-9, E-N: Activated Akt binds and phosphorylates several cellular proteins, including GSK3β (#67) [37], MDM2 (#68,#69) [40]. In this MIM, we assumed that an unknown P-ase 13 dephosphorylate MDM2 (#70). MDM2-P binds (#71) and promote ubiquitination, nuclear export and proteasomal degradation (#72) [41] of TP53 and blocks its ability to regulate target genes. TP53 can bind transcription binding sites ($TFBS_{TP53}$)(#73) and can inhibit the expression of MYC transcription through a mechanism that involves histone deacetylation (#74) [42]. TP53 also represses CCND1 transcription indirectly, by downregulating a transcriptional coactivator [43].The ARF protein can bind to the MDM2 (#75) protein and modulates down its ubiquitin ligase activity by inhibition of TP53 binding (#76), increasing the levels of the TP53 protein [41].

#77- 86, coord. 2-4, E-M: Activated Akt binds and phosphorylates GSK3β: GSK3β phosphorylated in Ser9 seems to result in inhibition of activity (#77), [37, 44, 45]. (*Some experimental reports [46, 47] have suggested a weak or controversial connection between the PI3K - AKT pathway and GSK3β*). We assumed that unknown phosphatases P-ase 4 dephosphorylates GSK3β (#78).

β-Catenin phosphorylation involves the sequential actions of casein kinase 1 (Ck1, not shown in this MIM) and GSK3β, and takes place in a protein complex, the "destruction complex" assembled by Axin and APC. In the absence of Wnt signalling, GSK3β binds (#79) and phosphorylates the scaffold protein Axin (#80). GSK3β : Axin-P complex binds (#81) and phosphorylates (#82) APC, APC-P binds to GSK3β : Axin-P complex; the "destruction complex" (GSK3β : Axin-P : APC-P complex) promotes the binding (#83) and phosphorylation of cytosolic β-catenin (#84) [48-52]. Phosphorylation in residues 45, 41, 37 and 33 of cytosolic β-catenin (non E-Cadherin bound) by the destruction complex, leads to its degradation

[48-54] by the ubiquitin–proteasome pathway (#85). We assumed that an unknow posphatase P-ase 6 can dephosphorylate Axin, APC and β-catenin in the "destruction complex" (P-ase 6 is also indicated as PP2A in [49, 54, 55]). P-ase 6 also dephoshorylates LRP5/6 (#86).

#87-99, coord. 1-9, C-G: β-catenin normally, associates at the cellular membrane with the adhesion molecule E-cadherin, while any free cytoplasmic β-catenin is phosphorylated and targeted for ubiquination-dependent degradation. E-Cadherin binds PTP1B phosphatase (#87) promoting binding between β-catenin and the cytoplasmatic domain of E-Cadherin (#88) [56, 57]; this binding (#89) inhibits the binding with members of the TCF/LEF family of transcription factor. In the intestinal epithelium, TCF7L2 is the most prominently expressed TCF family member [58, 59]. E-cadherin and TCF7L2 forms mutually exclusive complexes with β-catenin (#94), [59]. An activated ErbB family receptor can phosphorylate β -catenin at Y654 (#90) and can inhibit E-Cadherin binding (#91) and adhesive function [56, 57]. PY654 of β-catenin is target of PTP1B phosphatase (#92) [57]. As a consequence of this phosphorylation β-catenin is not sequestered from E-Cadherin: it is in equilibrium with free β-catenin which can translocate to the nucleus where β-cateninY654 (#93) and β-catenin (#94) can bind the TCF7L2 transcription factor (#95) [60, 61] and can activate the expression of target genes (#96) (in this MIM: CCND1 and MYC genes), [62-66]. In the absence of nuclear β-catenin, TCF7L2 functions instead as transcriptional repressor by binding to co-repressors of the Groucho–TLE family (#97) and binds (#98) and inhibits transcription of target genes [67, 68]. In the absence of TGFβ signaling, Smad4 binds the MYC promoter LEF/TCF-binding elements from the MYC promoter and activates MYC promoter activity (#99), [69]. In this MIM, it was assumed that SMAD4 also activates CCND1 gene transcription. The cadherin cytoplasmic domain binds to β-catenin, which in turn binds with weaker affinity to α-catenin [52]. This binding was omitted in this MIM (an example of simplification).

#100-105, coord. 1-4, E-G: Upon ligand interaction, Wnt, Frizzled receptors and the Wnt co-receptor LRP6 form a ternary signaling complex which recruits Dishevelled proteins to the plasma membrane [70] by direct binding [71]. The scaffold protein Dishevelled promotes the formation of an LRP-associated Wnt 'signalosome' [72] and directly interacts with Axin [73] and recruits it, and also associates GSK3β to the plasma membrane. Formation of signalosomes promotes sequential phosphorylation of LRP6 by GSK3β and casein kinase 1, of cytoplasmic domain of LRP5 or LRP6 at five critical PPSP/TP repeats [74]. The association of the axin complex with the phosphorylated LRP6 leads to (via an as yet unknown mechanism) inhibition of β-catenin phosphorylation. The function of the degradation complex is inhibited by this canonical pathway: β-catenin accumulates, enter the nucleus, and engages in transcriptional activation (#94, #96).

To make it easy, in this MIM, it was assumed that activated Wnt binds Frizzled receptors (#100), then LRP5/6 co-receptors (#101) and the Wnt:Frizzled:LRP5/6 complex binds the Dishevelled protein (#102). It was assumed that activated Wnt:Frizzled:LRP5/6:Dishevelled complex recruits GSK3β (#103) which phosphorylates LRP5 or LRP6 (#104): these sites allow docking of Axin (#105) and its recruitment to the plasma membrane. How Wnt signalling activates β-catenin through the blocking of GSK3β remains one of the main open questions in this pathway: different model have been proposed [48, 49, 74, 75]. In this MIM, it was assumed that LRP5 (or LRP6) can be dephosphorylated by an unknown phosphatase, P-ase 6 (#86).

#106-107, coord. 1-3, C-E: Kinase activity of TAK1 was stimulated in response to Wnt (#106, #107) [76].

#108-114, coord 1-2, B-C: Dimeric TGFβ (#108) binds with high affinity (#110) type II (TGFβR-II) receptors dimers (#109), on the cell surface. Dimeric TGFβ type I (TGFβR-I) (#111) binds dimeric TGFβR-II:dimeric TGFβ complex (#112). In the hetero-tetrameric receptor complex, type II receptors phosphorylate a serine/threonine-rich region (the GS region), located in the kinase domain of TGFβR-I (#113), which then propagates the signal [77-80]. PP1C can dephosphorylate active TGFβR-I (#114) [81].

#115-126, coord. 1-5, C-D: An activated TGFβ complex can bind (#115) and activate TAK1 (#116)[82]. In this MIM, it was assumed that P-ase 9 can dephosphorylate TAK1-P (#117). TAK1-P binds TAB2 scaffold protein (#118) [83] and phosphorylates NLK family of protein kinases and increases their kinase activity (#119-120). In this MIM, it was assumed that P-ase 10 can dephosphorylate NLK-P (#121). NLK-P can bind (#122) and directly phosphorylate TCF7L2 on two serine/threonine residues (#124) and can prevent the β-catenin:TCF7L2 complex from binding to DNA, thereby inhibiting the ability of β-catenin:TCF7L2 to activate transcription (#125). The TAK1-NLK pathway negatively regulates the canonical Wnt signaling pathway [83, 84]. In this MIM, it was assumed that P-ase 8 can dephosphorylate TCF7L2- P (#126).

#127-134, coord. 2-9, A-H: An activated TGFβ complex can bind (#127) and phosphorylate Smad3 and propagate the signal (#128); an activated TGFβ complex (via a region in the type I receptor) can bind Smad2 (#129) and phosphorylate Smad2 (#130) and propagate the signal. Smad4 can bind activated Smad2 and Smad3 (#132) [77-80] forming complexes that can translocate into the nucleus (#133). These SMAD complexes can then bind to a SMAD binding site ($TFBS_{SMAD}$) on MYC, repressing its transcription [85-87]. A similar inhibitory role of the TGF-β pathway on CCDN1 transcription has been

described [88, 89]. SMAD4 (most likely complexed with phosphorylated SMAD2 or SMAD3) binds to the promoter region of CCND1 upon TGF- β treatment, repressing transcription [90]. Dephosphorylation is accompanied with dissociation of the Smad2/3–Smad4 complex and export of its components to the cytoplasm (not shown in this MIM). PPM1A can dephosphorylate active Smad2 and Smad3, in the nucleus (#131) [91].

#135-147, coord. 6-7, M-P: Cdk4 binds exclusively to D-type cyclins (#135), Cdk2 binds to cyclins E (or A) (#136). Cyclin D:Cdk4 phosphorylates pRb at a subset of sites (#137, #138), Cyclin E:Cdk2 phosphorylates pRb at additional sites after the Cyclin D:Cdk4-specific sites have been phosphorylated. p16 competes with Cyclin D1 for binding to Cdk4 (#139) [92]. <u>In this MIM</u>, it was assumed that P-ase 14 can dephosphorylate pRb (#140, #141).

#142-147, coord. 7-8, F-P: Dimerization partner (DP) protein forms stable heterodimers with E2F (#142). Unphosphorylated (or hypophosphorylated) pRb (active form), can bind to DP:E2F complexes (#143) and block E2F-dependent transcription genes (#144, #145). Hyperphosphorylated pRb (inactive form), resulting from combined phosphorylation by Cyclin D:Cdk4 and Cyclin E:Cdk2 (#137, #138), abrogates the binding of pRb to DP:E2F complexes, leads to the release of E2F transcription factors and to the transcriptional activation of E2F-responsive genes (#146, #147). MYC and CCND1 are target genes of E2F [93, 94].

#148-151, coord. 8-9, B-P: <u>In this MIM</u>, MYC and CCND1 genes transcription has been described as importantly stimulated at least by the following: AP-1, AP1-P (#27), TCF7L2:βcatenin, TCF7L2:βcateninY654 (#95), TCF7L2:SMAD4 (#99), E2F:DP1 (#146). They can bind DNA in different sites. Transcription has been described as inhibited by SMADIIP:SMAD4 (#133); SMADIIIP:SMAD4 (#133); GROUCHO:TCF7L2 (#98); TP53 (#73, #74); E2F:DP1:RB (#144). Each transcription factor to activate gene expression, can recruit the transcriptional machinery such as RNA Polymerase II elongation complex (#148) to the promoter region. A completely operational RNA Polymerase II slides over the DNA CCND1 or MYC coding region (#149) and makes a copy of Cyclin D1 or c-myc mRNA (#150). The Cyclin D1 or c-Myc mRNA are degraded (#151), [95, 96].

References related to the MIM Annotation List

1. Jorissen RN, Walker F, Pouliot N, Garrett TP, Ward CW and Burgess AW. Epidermal growth factor receptor: mechanisms of activation and signalling. Exp Cell Res. 2003; 284(1):31-53.
2. Yarden Y and Sliwkowski MX. Untangling the ErbB signalling network. Nat Rev Mol Cell Biol. 2001; 2(2):127-137.
3. Citri A and Yarden Y. EGF-ERBB signalling: towards the systems level. Nat Rev Mol Cell Biol. 2006; 7(7):505-516.
4. Batzer AG, Rotin D, Urena JM, Skolnik EY and Schlessinger J. Hierarchy of binding sites for Grb2 and Shc on the epidermal growth factor receptor. Mol Cell Biol. 1994; 14(8):5192-5201.
5. Wills MK and Jones N. Teaching an old dogma new tricks: twenty years of Shc adaptor signalling. Biochem J. 2012; 447(1):1-16.
6. Markevich NI, Moehren G, Demin OV, Kiyatkin A, Hoek JB and Kholodenko BN. Signal processing at the Ras circuit: what shapes Ras activation patterns? Systems biology. 2004; 1(1):104-113.
7. Malumbres M and Barbacid M. RAS oncogenes: the first 30 years. Nat Rev Cancer. 2003; 3(6):459-465.
8. McCormick F. ras GTPase activating protein: signal transmitter and signal terminator. Cell. 1989; 56(1):5-8.
9. Suenaga A, Hatakeyama M, Kiyatkin AB, Radhakrishnan R, Taiji M and Kholodenko BN. Molecular Dynamics Simulations Reveal that Tyr-317 Phosphorylation Reduces Shc Binding Affinity for Phosphotyrosyl Residues of Epidermal Growth Factor Receptor. Biophysical Journal. 2009; 96(6):2278-2288.
10. Kraut-Cohen J, Muller WJ and Elson A. Protein-tyrosine phosphatase epsilon regulates Shc signaling in a kinase-specific manner - Increasing coherence in tyrosine phosphatase signaling. Journal of Biological Chemistry. 2008; 283(8):4612-4621.
11. Brightman FA and Fell DA. Differential feedback regulation of the MAPK cascade underlies the quantitative differences in EGF and NGF signalling in PC12 cells. Febs Letters. 2000; 482(3):169-174.
12. Udell CM, Rajakulendran T, Sicheri F and Therrien M. Mechanistic principles of RAF kinase signaling. Cellular and Molecular Life Sciences. 2011; 68(4):553-565.
13. Roskoski R. RAF protein-serine/threonine kinases: Structure and regulation. Biochemical and Biophysical Research Communications. 2010; 399(3):313-317.

14. Matallanas D, Birtwistle M, Romano D, Zebisch A, Rauch J, von Kriegsheim A and Kolch W. Raf family kinases: old dogs have learned new tricks. Genes Cancer. 2011; 2(3):232-260.

15. Gille H, Kortenjann M, Thomae O, Moomaw C, Slaughter C, Cobb MH and Shaw PE. ERK phosphorylation potentiates Elk-1-mediated ternary complex formation and transactivation. EMBO J. 1995; 14(5):951-962.

16. Karin M, Liu Z and Zandi E. AP-1 function and regulation. Curr Opin Cell Biol. 1997; 9(2):240-246.

17. Shaulian E and Karin M. AP-1 in cell proliferation and survival. Oncogene. 2001; 20(19):2390-2400.

18. Albanese C, Johnson J, Watanabe G, Eklund N, Vu D, Arnold A and Pestell RG. Transforming p21ras mutants and c-Ets-2 activate the cyclin D1 promoter through distinguishable regions. The Journal of biological chemistry. 1995; 270(40):23589-23597.

19. Bakiri L, Lallemand D, Bossy-Wetzel E and Yaniv M. Cell cycle-dependent variations in c-Jun and JunB phosphorylation: a role in the control of cyclin D1 expression. EMBO J. 2000; 19(9):2056-2068.

20. Zhang HS, Yan B, Li XB, Fan L, Zhang YF, Wu GH, Li M and Fang J. PAX2 protein induces expression of cyclin D1 through activating AP-1 protein and promotes proliferation of colon cancer cells. The Journal of biological chemistry. 2012; 287(53):44164-44172.

21. Kerkhoff E, Houben R, Loffler S, Troppmair J, Lee JE and Rapp UR. Regulation of c-myc expression by Ras/Raf signalling. Oncogene. 1998; 16(2):211-216.

22. Iavarone C, Catania A, Marinissen MJ, Visconti R, Acunzo M, Tarantino C, Carlomagno MS, Bruni CB, Gutkind JS and Chiariello M. The platelet-derived growth factor controls c-myc expression through a JNK- and AP-1-dependent signaling pathway. The Journal of biological chemistry. 2003; 278(50):50024-50030.

23. Weston CR and Davis RJ. The JNK signal transduction pathway. Curr Opin Genet Dev. 2002; 12(1):14-21.

24. Holt KH, Waters SB, Okada S, Yamauchi K, Decker SJ, Saltiel AR, Motto DG, Koretzky GA and Pessin JE. Epidermal growth factor receptor targeting prevents uncoupling of the Grb2-SOS complex. The Journal of biological chemistry. 1996; 271(14):8300-8306.

25. Wolf J, Dronov S, Tobin F and Goryanin I. The impact of the regulatory design on the response of epidermal growth factor receptor-mediated signal transduction towards oncogenic mutations. FEBS J. 2007; 274(21):5505-5517.

26. Wang R, He G, Nelman-Gonzalez M, Ashorn CL, Gallick GE, Stukenberg PT, Kirschner MW and Kuang J. Regulation of Cdc25C by ERK-MAP kinases during the G2/M transition. Cell. 2007; 128(6):1119-1132.

27. Prahallad A, Sun C, Huang S, Di Nicolantonio F, Salazar R, Zecchin D, Beijersbergen RL, Bardelli A and Bernards R. Unresponsiveness of colon cancer to BRAF(V600E) inhibition through feedback activation of EGFR. Nature. 2012; 483(7387):100-103.

28. Fukami K, Inanobe S, Kanemaru K and Nakamura Y. Phospholipase C is a key enzyme regulating intracellular calcium and modulating the phosphoinositide balance. Prog Lipid Res. 2010; 49(4):429-437.

29. Todderud G, Wahl MI, Rhee SG and Carpenter G. Stimulation of phospholipase C-gamma 1 membrane association by epidermal growth factor. Science. 1990; 249(4966):296-298.

30. Kolch W, Heidecker G, Kochs G, Hummel R, Vahidi H, Mischak H, Finkenzeller G, Marme D and Rapp UR. Protein kinase C alpha activates RAF-1 by direct phosphorylation. Nature. 1993; 364(6434):249-252.

31. Bader AG, Kang S, Zhao L and Vogt PK. Oncogenic PI3K deregulates transcription and translation. Nat Rev Cancer. 2005; 5(12):921-929.

32. Chalhoub N and Baker SJ. PTEN and the PI3-kinase pathway in cancer. Annu Rev Pathol. 2009; 4:127-150.

33. Kiyatkin A, Aksamitiene E, Markevich NI, Borisov NM, Hoek JB and Kholodenko BN. Scaffolding protein Grb2-associated binder 1 sustains epidermal growth factor-induced mitogenic and survival signaling by multiple positive feedback loops. The Journal of biological chemistry. 2006; 281(29):19925-19938.

34. Rodriguez-Viciana P, Warne PH, Dhand R, Vanhaesebroeck B, Gout I, Fry MJ, Waterfield MD and Downward J. Phosphatidylinositol-3-OH kinase as a direct target of Ras. Nature. 1994; 370(6490):527-532.

35. Lehr S, Kotzka J, Avci H, Sickmann A, Meyer HE, Herkner A and Muller-Wieland D. Identification of major ERK-related phosphorylation sites in Gab1. Biochemistry. 2004; 43(38):12133-12140.

36. Verma S, Vaughan T and Bunting KD. Gab adapter proteins as therapeutic targets for hematologic disease. Adv Hematol. 2012; 2012:380635.

37. Hemmings BA and Restuccia DF. PI3K-PKB/Akt pathway. Cold Spring Harb Perspect Biol. 2012; 4(9):a011189.

38. Gao TY, Furnari F and Newton A. PHLPP: a novel phosphatase that directly dephosphorylates Akt, promotes apoptosis and suppresses tumor growth. Faseb Journal. 2005; 19(4):A258-A258.

39. Zimmermann S and Moelling K. Phosphorylation and regulation of Raf by Akt (protein kinase B). Science. 1999; 286(5445):1741-1744.

40. Ogawara Y, Kishishita S, Obata T, Isazawa Y, Suzuki T, Tanaka K, Masuyama N and Gotoh Y. Akt enhances Mdm2-mediated ubiquitination and degradation of p53. Journal of Biological Chemistry. 2002; 277(24):21843-21850.

41. Moll UM and Petrenko O. The MDM2-p53 interaction. Molecular Cancer Research. 2003; 1(14):1001-1008.

42. Ho JSL, Ma WL, Mao DYL and Benchimol S. p53-dependent transcriptional repression of c-myc is required for G(1) cell cycle arrest. Molecular and Cellular Biology. 2005; 25(17):7423-7431.

43. Rocha S, Martin AM, Meek DW and Perkins ND. p53 Represses cyclin D1 transcription through down regulation of Bcl-3 and inducing increased association of the p52 NF-kappa B subunit with histone deacetylase 1. Molecular and Cellular Biology. 2003; 23(13):4713-4727.

44. Cross DAE, Alessi DR, Cohen P, Andjelkovich M and Hemmings BA. Inhibition of Glycogen-Synthase Kinase-3 by Insulin-Mediated by Protein-Kinase-B. Nature. 1995; 378(6559):785-789.

45. Chappell WH, Steelman LS, Long JM, Kempf RC, Abrams SL, Franklin RA, Basecke J, Stivala F, Donia M, Fagone P, Malaponte G, Mazzarino MC, Nicoletti F, Libra M, Maksimovic-Ivanic D, Mijatovic S, et al. Ras/Raf/MEK/ERK and PI3K/PTEN/Akt/mTOR Inhibitors: Rationale and Importance to Inhibiting These Pathways in Human Health. Oncotarget. 2011; 2(3):135-164.

46. Ng SS, Mahmoudi T, Danenberg E, Bejaoui I, de Lau W, Korswagen HC, Schutte M and Clevers H. Phosphatidylinositol 3-kinase signaling does not activate the wnt cascade. The Journal of biological chemistry. 2009; 284(51):35308-35313.

47. Voskas D, Ling LS and Woodgett JR. Does GSK-3 provide a shortcut for PI3K activation of Wnt signalling? F1000 Biol Rep. 2010; 2:82.

48. Kikuchi A. Regulation of beta-catenin signaling in the Wnt pathway. Biochem Biophys Res Commun. 2000; 268(2):243-248.

49. Avaiable at www.wnt.stanford.edu.

50. Wu D and Pan W. GSK3: a multifaceted kinase in Wnt signaling. Trends Biochem Sci. 2010; 35(3):161-168.

51. Kishida S, Yamamoto H, Ikeda S, Kishida M, Sakamoto I, Koyama S and Kikuchi A. Axin, a negative regulator of the wnt signaling pathway, directly interacts with adenomatous polyposis coli and regulates the stabilization of beta-catenin. The Journal of biological chemistry. 1998; 273(18):10823-10826.

52. Daugherty RL and Gottardi CJ. Phospho-regulation of Beta-catenin adhesion and signaling functions. Physiology (Bethesda). 2007; 22:303-309.

53. Wu G and He X. Threonine 41 in beta-catenin serves as a key phosphorylation relay residue in beta-catenin degradation. Biochemistry. 2006; 45(16):5319-5323.

54. Su YY, Fu CJ, Ishikawa S, Stella A, Kojima M, Shitoh K, Schreiber EM, Day BW and Liu B. APC Is Essential for Targeting Phosphorylated beta-Catenin to the SCF(beta-TrCP) Ubiquitin Ligase. Molecular Cell. 2008; 32(5):652-661.

55. Zhang W, Yang J, Liu YJ, Chen X, Yu TX, Jia JH and Liu CM. PR55 alpha, a Regulatory Subunit of PP2A, Specifically Regulates PP2A-mediated beta-Catenin Dephosphorylation. Journal of Biological Chemistry. 2009; 284(34):22649-22656.

56. Lilien J and Balsamo J. The regulation of cadherin-mediated adhesion by tyrosine phosphorylation/dephosphorylation of beta-catenin. Current Opinion in Cell Biology. 2005; 17(5):459-465.

57. Wong NACS and Pignatelli M. beta-catenin - A linchpin in colorectal carcinogenesis? American Journal of Pathology. 2002; 160(2):389-401.

58. Korinek V, Barker N, Morin PJ, vanWichen D, deWeger R, Kinzler KW, Vogelstein B and Clevers H. Constitutive transcriptional activation by a beta-catenin-Tcf complex in APC(-/-) colon carcinoma. Science. 1997; 275(5307):1784-1787.

59. van de Wetering M, Sancho E, Verweij C, de Lau W, Oving I, Hurlstone A, van der Horn K, Batlle E, Coudreuse D, Haramis AP, Tion-Pon-Fong M, Moerer P, van den Born M, Soete G, Pals S, Eilers M, et al. The beta-catenin/TCF-4 complex imposes a crypt progenitor phenotype on colorectal cancer cells. Cell. 2002; 111(2):241-250.

60. Orsulic S, Huber O, Aberle H, Arnold S and Kemler R. E-cadherin binding prevents beta-catenin nuclear localization and beta-catenin/LEF-1-mediated transactivation. Journal of Cell Science. 1999; 112(8):1237-1245.

61. Bienz M and Clevers H. Linking colorectal cancer to Wnt signaling. Cell. 2000; 103(2):311-320.

62. He TC, Sparks AB, Rago C, Hermeking H, Zawel L, da Costa LT, Morin PJ, Vogelstein B and Kinzler KW. Identification of c-MYC as a target of the APC pathway. Science. 1998; 281(5382):1509-1512.

63. Tetsu O and McCormick F. beta-catenin regulates expression of cyclin D1 in colon carcinoma cells. Nature. 1999; 398(6726):422-426.

64. Shtutman M, Zhurinsky J, Simcha I, Albanese C, D'Amico M, Pestell R and Ben-Ze'ev A. The cyclin D1 gene is a target of the beta-catenin/LEF-1 pathway. Proceedings of the National Academy of Sciences of the United States of America. 1999; 96(10):5522-5527.

65. Piedra J, Martinez D, Castano J, Miravet S, Dunach M and de Herreros AG. Regulation of beta-catenin structure and activity by tyrosine phosphorylation. Journal of Biological Chemistry. 2001; 276(23):20436-20443.

66. van Veelen W, Le NH, Helvensteijn W, Blonden L, Theeuwes M, Bakker ERM, Franken PF, van Gurp L, Meijlink F, van der Valk MA, Kuipers EJ, Fodde R and Smits R. beta-catenin tyrosine 654 phosphorylation increases Wnt signalling and intestinal tumorigenesis. Gut. 2011; 60(9):1204-1212.

67. Daniels DL and Weis WI. beta-catenin directly displaces Groucho/TLE repressors from Tcf/Lef in Wnt-mediated transcription activation. Nature Structural & Molecular Biology. 2005; 12(4):364-371.

68. Arce L, Pate KT and Waterman ML. Groucho binds two conserved regions of LEF-1 for HDAC-dependent repression. Bmc Cancer. 2009; 9.

69. Lim SK and Hoffmann FM. Smad4 cooperates with lymphoid enhancer-binding factor 1/T cell-specific factor to increase c-myc expression in the absence of TGF-beta signaling. Proceedings of the National Academy of Sciences of the United States of America. 2006; 103(49):18580-18585.

70. Angers S and Moon RT. Proximal events in Wnt signal transduction. Nature Reviews Molecular Cell Biology. 2009; 10(7):468-477.

71. Wong HC, Bourdelas A, Krauss A, Lee HJ, Shao YM, Wu DQ, Mlodzik M, Shi DL and Zheng J. Direct binding of the PDZ domain of Dishevelled to a conserved internal sequence in the C-terminal region of frizzled. Molecular Cell. 2003; 12(5):1251-1260.

72. Bilic J, Huang YL, Davidson G, Zimmermann T, Cruciat CM, Bienz M and Niehrs C. Wnt induces LRP6 signalosomes and promotes dishevelled-dependent LRP6 phosphorylation. Science. 2007; 316(5831):1619-1622.

73. Fiedler M, Mendoza-Topaz C, Rutherford TJ, Mieszczanek J and Bienz M. Dishevelled interacts with the DIX domain polymerization interface of Axin to interfere with its function in down-regulating beta-catenin. Proceedings of the National Academy of Sciences of the United States of America. 2011; 108(5):1937-1942.

74. Taelman VF, Dobrowolski R, Plouhinec JL, Fuentealba LC, Vorwald PP, Gumper I, Sabatini DD and De Robertis EM. Wnt Signaling Requires Sequestration of Glycogen Synthase Kinase 3 inside Multivesicular Endosomes. Cell. 2010; 143(7):1136-1148.

75. Zeng X, Tamai K, Doble B, Li ST, Huang H, Habas R, Okamura H, Woodgett J and He X. A dual-kinase mechanism for Wnt co-receptor phosphorylation and activation. Nature. 2005; 438(7069):873-877.

76. Ishitani T, Ninomiya-Tsuji J, Nagai S, Nishita M, Meneghini M, Barker N, Waterman M, Bowerman B, Clevers H, Shibuya H and Matsumoto K. The TAK1-NLK-MAPK-related pathway antagonizes signalling between beta-catenin and transcription factor TCF. Nature. 1999; 399(6738):798-802.

77. Derynck R and Zhang YE. Smad-dependent and Smad-independent pathways in TGF-beta family signalling. Nature. 2003; 425(6958):577-584.

78. Clarke DC and Liu XD. Decoding the quantitative nature of TGF-beta/Smad signaling. Trends in cell biology. 2008; 18(9):430-442.

79. Massague J, Blain SW and Lo RS. TGFbeta signaling in growth control, cancer, and heritable disorders. Cell. 2000; 103(2):295-309.

80. Massague J. TGF beta signalling in context. Nature Reviews Molecular Cell Biology. 2012; 13(10):616-630.

81. Shi WB, Sun CX, He B, Xiong WC, Shi XM, Yao DC and Cao X. GADD34-PP1c recruited by Smad7 dephosphorylates TGF beta type 1 receptor. Journal of Cell Biology. 2004; 164(2):291-300.

82. Yamaguchi K, Shirakabe T, Shibuya H, Irie K, Oishi I, Ueno N, Taniguchi T, Nishida E and Matsumoto K. Identification of a Member of the Mapkkk Family as a Potential Mediator of Tgf-Beta Signal-Transduction. Science. 1995; 270(5244):2008-2011.

83. Li M, Wang H, Huang T, Wang JY, Ding Y, Li ZF, Zhang JK and Li L. TAB2 Scaffolds TAK1 and NLK in Repressing Canonical Wnt Signaling. Journal of Biological Chemistry. 2010; 285(18):13397-13404.

84. Ishitani T, Ninomiya-Tsuji J and Matsumoto K. Regulation of lymphoid enhancer factor 1/T-Cell factor by mitogen-activated protein kinase-related nemo-like kinase-dependent phosphorylation in Wnt/beta-catenin signaling. Molecular and Cellular Biology. 2003; 23(4):1379-1389.

85. Yagi K, Furuhashi M, Aoki H, Goto D, Kuwano H, Sugamura K, Miyazono K and Kato M. c-myc is a downstream target of the Smad pathway. Journal of Biological Chemistry. 2002; 277(1):854-861.

86. Chen CR, Kang YB, Siegel PM and Massague J. E2F4/5 and p107 as Smad cofactors linking the TGF beta receptor to c-myc repression. Cell. 2002; 110(1):19-32.

87. Massague J, Seoane J and Wotton D. Smad transcription factors. Genes & Development. 2005; 19(23):2783-2810.

88. Ko TC, Sheng HM, Reisman D, Thompson EA and Beauchamp RD. Transforming Growth-Factor-Beta-1 Inhibits Cyclin D1 Expression in Intestinal Epithelial-Cells. Oncogene. 1995; 10(1):177-184.

89. Mithani SK, Balch GC, Shiou SR, Whitehead RH, Datta PK and Beauchamp RD. Smad3 has a critical role in TGF-beta-mediated growth inhibition and apoptosis in colonic epithelial cells. Journal of Surgical Research. 2004; 117(2):296-305.

90. Ding ZH, Wu CJ, Chu GC, Xiao YH, Ho D, Zhang JF, Perry SR, Labrot ES, Wu XQ, Lis R, Hoshida Y, Hiller D, Hu BL, Jiang S, Zheng HW, Stegh AH, et al. SMAD4-dependent barrier constrains prostate cancer growth and metastatic progression. Nature. 2011; 470(7333):269-+.

91. Lin X, Duan XY, Liang YY, Su Y, Wrighton KH, Long JY, Hu M, Davis CM, Wang JR, Brunicardi FC, Shi YG, Chen YG, Meng AM and Feng XH. PPM1A functions as a Smad phosphatase to terminate TGF beta signaling. Cell. 2006; 125(5):915-928.

92. Kohn KW. Molecular interaction map of the mammalian cell cycle control and DNA repair systems. Mol Biol Cell. 1999; 10(8):2703-2734.

93. Bracken AP, Ciro M, Cocito A and Helin K. E2F target genes: unraveling the biology. Trends in Biochemical Sciences. 2004; 29(8):409-417.

94. Chen HZ, Tsai SY and Leone G. Emerging roles of E2Fs in cancer: an exit from cell cycle control. Nature Reviews Cancer. 2009; 9(11):785-797.

95. Swartwout SG and Kinniburgh AJ. C-Myc Rna Degradation in Growing and Differentiating Cells - Possible Alternate Pathways. Molecular and Cellular Biology. 1989; 9(1):288-295.

96. Alao JP. The regulation of cyclin D1 degradation: roles in cancer development and the potential for therapeutic invention. Molecular Cancer. 2007; 6.

Supplementary Table 1.2 - Glossary (referred to the MIM Fig. 1 and Supplementary Fig. 1.3)

In our MIM network we have represented 15 kinase-related cartouches (kinases can act on more than one protein), 7 known phosphatases have been described independently, and 14 additional phosphatases (each kinase activity has to be equilibrated with a corresponding phosphatase activity) have been grouped together (n. 44 in the Glossary), 1 phospholipase (PLCγ), 31 signaling/adaptor-proteins-related cartouches (for TGFβ, WNT and EGF, in each case we have indicated a single molecule as a representative of a family of molecules), 6 small molecules as small rectangular cartouches, 8 white transcription-related cartouches. In the Glossary, we have grouped together three ErbB-family receptors (n. 27 in the Glossary). PIP2 and PIP3 have been grouped together (n. 48 in the Glossary), and GDP and GTP have been grouped together (n. 35 in the Glossary). In conclusion, the Glossary, which includes all the molecules represented in our MIM (colored + white cartouches), only contains 69 numbered items. A total of 85 cartouches are represented in our MIM.

In the signaling network region depicted in our MIM only pathway-involved basic species are represented, not the 447 reactants (complexes) involved in ODEs.

In the Glossary:

Signaling proteins/ adaptor proteins: in **bold**

Kinases/GTPase: <u>underlined</u>

Phosphatases: in ***bold italics***

Small Molecules: in <u>*italics and underlined*</u>

white transcription-related cartouches: in *italics*

phospholipase: <u>**bold** and underlined</u>

1	**APC**	**A**denomatous **p**olyposis **c**oli protein is a tumor suppressor. Promotes rapid degradation of β-Catenin and participates in Wnt signaling as a negative regulator.	MIM's coord. 4,F
2	<u>Akt</u>	The Akt family proteins, also known as protein kinases B (PKB), are serine-threonine protein kinases: they mediate many of the downstream effects of PI3K.	MIM's coord. 2,M
3	**AP1**	The mammalian **A**ctivating **P**rotein-**1** proteins are homodimers and heterodimers composed of basic region-leucine zipper (bZIP) proteins that belong to the Jun (c-Jun, JunB and JunD), Fos (c-Fos, FosB, Fra-1 and Fra-2), Jun dimerization partners (JDP1 and JDP2) and the closely related activating transcription factors (ATF2, LRF1/ ATF3 and B-ATF) subfamiliesis. The AP-1 transcription factors control cell proliferation, survival and death.	MIM's coord. 6, I
4	**ARF**	The gene CDKN2A (cyclin-dependent kinase inhibitor 2A) generates several transcript variants which differ in their first exons. Three alternatively spliced variants encoding distinct proteins have been reported: two of which, p15INK4b and **p16INK4a**, encode structurally related isoformsThe remaining transcript includes an alternate first exon located 20 Kb upstream of the remainder of the gene; this transcript contains an alternate open reading frame (ARF) that specifies a protein which is structurally unrelated to the products of the other variants. This **ARF** product functions as a stabilizer of the tumor suppressor protein p53 as it can interact with, and sequester, the E3 ubiquitin-protein ligase MDM2, a protein responsible for the degradation of p53. Tumor suppressor gene.	MIM's coord. 4, L-M
5	**Axin**	A scaffold protein that binds directly to many proteins involved in the Wnt signaling pathway and facilitates the phosphorylation of β-Catenin and APC, by GSK3β.	MIM's coord. 4,E-F
6	<u>BRAF</u>	The protein isoform (otherwise known as B-Raf) is a MAP kinase kinase kinase (MAP3K), which functions downstream of the RAS family of membrane associated proteins to which it binds directly.	MIM's coord. 4,I
7	**β-catenin**	A regulatory protein that integrates cell surface signals with the actin cytoskeleton and transcription factors involved in cell proliferation.	MIM's coord. 5,D-E
8	***Cdc25c***	The **c**ell **d**ivision **c**ycle 25 homolog **C** is a tyrosine phosphatase and belongs to the Cdc25 phosphatase family. When activated dephosphorylates EGFR and is required to control cyclin-dependent kinase (CdK) dephosphorylation and activation..	MIM's coord. 6,G
9	<u>Cdk2</u>	Monomeric CDK is catalytically inactive and require both cyclin association and phosphorylation for full activity. Cyclin-dependent kinase (CDK) forms heterodimers consisting of a proline-directed serine/threonine kinase and a regulatory cyclin subunit. CDK2 is one the major regulators of cell cycle, contributing to induction and/or progression at S phase.	MIM's coord. 7,M-N

10	Cdk4	Cyclin-dependent kinase (CDK) forms heterodimers consisting of a proline-directed serine/threonine kinase and a regulatory cyclin subunit.CDK4 is one the major regulators of the cell cycle, contributing to induction and/or progression at G1/S transition.	MIM's coord. 6-7,M-N
11	*TFBS$_{TCF7L2}$* *CCND1/MYC transcription factors binding site*	DNA binding sites for transcription factors TCF7L2_BetaCatenin (activating), TCF7L2_BetaCateninY654 (activating), TCF7L2_SMAD4 (activating) and TFC7L2_GROUCHO (inhibitory).	MIM's coord. 9,C
12	*TFBS$_{SMAD}$* *CCND1/MYC transcription factors binding site*	DNA binding sites for transcription factors SMADII-P:SMAD4 (inhibitory) and SMADIII-P:SMAD4 (inhibitory).	MIM's coord. 9,B-C
13	*TFBS$_{AP1}$* *CCND1/MYC transcription factors binding site*	DNA binding sites for transcription factors AP1 (activating) and AP1P (activating).	MIM's coord. 9,D-E
14	*TFBS$_{TP53}$* *CCND1/MYC transcription factors binding site*	DNA binding sites for transcription factor TP53(inhibitory).	MIM's coord. 9,E
15	*TFBS$_{E2F-DP1}$* *CCND1/MYC transcription factors binding site*	DNA binding sites for transcription factors E2F:DP1 (activating) and E2F:DP1:pRB (inhibitory).	MIM's coord. 9,F
16	**Cyclin D**	D-type of cyclins (cyclins D1, D2, and D3) promote cell progression from G1 - S phase by interacting with CDK4 and CDK6.	MIM's coord. 6,M-N
17	*CCND1/MYC DNA coding region*	RNAPol II elongation complex elongates CCND1/MYC mRNA sliding over this region.	MIM's coord. 9,G
18	*CCND1/MYC mRNA*	CCND1 is a key regulator of G1-to-S phase progression of the cell cycle. The cyclin D1 protein levels are largely controlled at the transcriptional level and by ubiquitin-mediated degradation. cyclin D1 is important for the development and progression of several cancers: it is frequently overexpressed in human cancers.<br>MYC plays a role in cell cycle progression, apoptosis and cellular transformation. It functions as a transcription factor that regulates transcription of specific target genes.	MIM's coord. 9,H-I
19	**Cyclin E**	cyclin E is a member of the cyclin family which forms a complex with cyclin-dependent kinase (CDK2). Cyclin E/CDK2 regulates multiple cellular processes by phosphorylating numerous downstream proteins.	MIM's coord. 8,M-N

20	*DAG*	**Dia**cyl**g**lycerol functions as a second messenger signaling lipid. DAG stays inside the membrane.	MIM's coord. 2,N-O
21	**DP1**	Transcription factor Dimerization partner 1. Component of the E2F/DP transcription factor complex. Forms heterodimers with E2F family members, binds DNA cooperatively with E2F family members through the E2 recognition site.	MIM's coord. 8,P
22	**Dvl**	***Dishevelled*** are scaffold proteins which relays Wnt signals from receptors to downstream effectors.	MIM's coord. 2, E-F
23	**E2F**	E2F are a family of transcription factors: they play a crucial role in the control of cell cycle. The protein include a DNA binding domain, a dimerization domain which determines interaction with the differentiation regulated transcription factor proteins (DP), a transactivation domain enriched in acidic amino acids, and a tumor suppressor protein association domain which is embedded within the transactivation domain. E2F1-E2F3, have an additional cyclin binding domain.	MIM's coord. 7,P
24	**E-Cadherin**	**E**pithelial-Cadherin (Calcium dependent adhesion molecules) is a subclass of type-1 trans- membrane proteins. It plays important roles in cell adhesion.	MIM's coord.1-2,D
25	**EGF**	**E**pidermal **G**rowth **F**actor; belongs to EGF family factors.	MIM's coord. 1,G
26	*Elongation complex RNA Pol II*	A fully activated RNAPol II complex elongates mRNA.	MIM's coord. 8,G
27	ErbB family receptors	A family of receptor tyrosine kinases (RTKs): ErbB1(v-erb-b1 avian erythroblastic leukemia viral oncogene homolog 1)/EGFR1(**E**pidermal **g**rowth **f**actor **r**eceptor 1) is a well known dominant proto-onco-protein; ErbB2/Her2 (**H**uman **E**pidermal growth factor **R**eceptor 2) /neu (neuro/glioblastoma derived oncogene homolog 2) lacks the capacity to interact with a ligand; ErbB3 is kinase-defective; ErbB4 shares features with ErbB1(not included in the model).	MIM's coord.1-2,G
28	ERK	**E**xtracellular signal-**R**egulated **K**inase or MAPK1 (mitogen-activated protein kinase 1). The activation of this kinase requires its phosphorylation by upstream kinases. Upon activation, this kinase translocates to the nucleus of the stimulated cells, where it phosphorylates nuclear targets.	MIM's coord. 6,I
29	**Frz**	The **Friz**zled proteins are seven-pass transmembrane domain cell surface receptors for Wnt ligands, that belong phylogenetically to the large family of G protein-coupled receptors (GPCRs). The mammalian genome harbors 10 frizzled genes.	MIM's coord. 1-2, E

30	**GAB1**	Belongs to the family of **G**rb2-**a**ssciated **b**inder (GAB) adaptor proteins.	MIM's coord. 2,I
31	<u>GAP</u>	A generic **G**TPase-**a**ctivating **p**rotein (RAS-GAPs) that drastically promotes the weak intrinsic ability of KRAS to hydrolyze GTP and become inactive.	MIM's coord. 3,H-I
32	**Grb2**	**G**rowth factor **r**eceptor-**b**inding protein **2** is an adaptor protein: it plays key roles in signaling downstream of ErbB family receptors.	MIM's coord. 2,G
33	**GROUCHO**	The protein is the prototype for a large family of co-repressors. As transcriptional co-repressors, Groucho/TLE proteins do not bind to DNA directly, but rather are recruited by DNA-bound repressor proteins.	MIM's coord. 5,C
34	<u>GSK3β</u>	The **G**lycogen **s**ynthase **k**inase-3 (GSK-3) protein family was originally characterized as a serine/threonine kinase that phosphorylates and inactivates glycogen synthase; it is now implicated in the regulation of several physiological responses in mammalian cells through phosphorylation of many substrates.	MIM's coord. 4,G
35	<u>*GTP, GDP*</u>	**G**uanosine-5'-**trip**hosphate is essential to signal transduction, especially with G-proteins, in second-messenger mechanisms where it is converted to guanosine **dip**hosphate) through the action of GTPases	MIM's coord. 2,I
36	<u>*IP_3*</u>	**I**nositol **t**risphosphate together with diacylglycerol (DAG), is a secondary messenger molecule used in signal transduction and lipid signaling in biological cells. IP3 is soluble and diffuses through the cell.	MIM's coord. 2,N
37	**KRAS**	Small G protein which transmits signals via different effector proteins. RAS-family proteins are well known dominant proto-onco-proteins.	MIM's coord. 2,I
38	**LRP5/6**	***L**DL **r**eceptor related **p**rotein **5** and **6** are co-receptor with Frz in Wnt pathway.*	MIM's coord. 2,F
39	**MDM2**	MDM2 (**m**ouse **d**ouble-**m**inute 2) belongs to the family of E3 ubiquitin ligases that contain a RING domain and serves as the major E3 ubiquitin ligase for p53 degradationan. MDM2 has potentially oncogenic activity.	MIM's coord. 4,M
40	<u>MEK</u>	MEK kinase or MAP3K (mitogen-activated protein kinase kinase kinase) is a serine/threonine kinase.	MIM's coord. 5,I

41	***MKP3***	MKP3 is a member of MKPs (**M**AP **K**inase **p**hosphatases), a class of phosphatases with dual-specificity activity toward threonine and tyrosine residues that dephosphorylates and inactivates the MAP kinases.	MIM's coord. 5,H-I
42	NLK	**N**emo **l**ike **k**inase is an atypical mitogen-activated protein kinase (MAPK), that belongs to the proline-directed serine/threonine protein kinase superfamily. It phosphorylates several transcription factors: suppresses the transcriptional activity of β-catenin/T-cell factor complex through phosphorylation of TCF/LEF.	MIM's coord. 4, C
43	**P16**	The gene CDKN2A (cyclin-dependent kinase inhibitor 2A) generates several transcript variants which differ in their first exons. Three alternatively spliced variants encoding distinct proteins have been reported: two of which, p15INK4b and **p16INK4a**, encode structurally related isoforms known to function as inhibitors of CDK4 kinase.	MIM's coord. 6, N
44	***P-ase 1-14***	Unknown phosphatases. The effect of a P-kinase has to be balanced by a phosphatase, to avoid shifting of phosphorylated proteins toward a complete and unbalanced phosphorylation.	MIM's coord. 2-6, C-P
45	PDK1	3-**p**hosphoinositide-**d**ependent **p**rotein **k**inase (PDPK) is required for full activation of Akt.	MIM's coord. 3,N
46	***PHLPP***	The protein phosphatase PHLPP (**PH** domain **l**eucine-rich repeat **p**rotein **p**hosphatase), especially dephosphorylates Akt.	MIM's coord. 2,N
47	PI3K	**P**hospho**i**nositide 3-**k**inases are a family of intracellular lipid kinases that phosphorylate the 3′-hydroxyl group of phosphatidylinositols and phosphoinositides. PI3Ks are classified into classes I, II, and III, based on structure and substrate specificity. Class I PI3Ks phosphorylate phosphatidylinositol-4,5-bisphosphate (PIP_2) to generate phosphatidylinositol-3,4,5-trisphosphate (PIP_3); Class IA PI3Ks are activated by growth factor receptor tyrosine kinases (RTKs) and are heterodimers (regulatory subunit + catalytic subunit).	MIM's coord. 2,L
48	*PIP_2, PIP_3*	Phosphatidylinositol-3,4-bisphosphate (PtdIns-4,5-P_2);<br>Phosphatidylinositol 3,4,5-trisphosphate (PtdIns-3,4,5-P_3).<br>They are defined as second messengers. The concentrations of PIP**3** and PIP**2** are regulated at the inner cellular membrane by PI3K and PTEN.	MIM's coord. 2,M-N
49	***PK Inactivator***	The complex inactivation of PI3K was synthesized as a PK Inactivator function.	MIM's coord. 3,L-M
50	PKC	**P**rotein **k**inase **C** is a family of serine- and threonine-specific protein kinases that can be activated by calcium and the second messenger diacylglycerol. PKC family members phosphorylates a wide variety of protein targets and are<br>known to be involved in diverse cellular signaling pathways.	MIM's coord. 2-3,O

51	**PLCγ**	**P**hospho**l**ipase **C** **g**amma binds directly the ErbB family receptors and is activated by their kinase activity: it is implicated in the formation of inositol 1,4,5-triphosphate and generation of a Ca^{2+} response.	MIM's coord. 2,O
52	***PP1C***	**P**rotein **P**hosphatase **1C** works as a phosphatase of TGFβR-I	MIM's coord. 2-3,C
53	***PPM1A***	**P**rotein **P**hosphatase **1A** is a member of the PPM family of monomeric, metal ion-dependent protein serine/threonine phosphatase; it acts as a phosphatase for TGF-β-activated Smad2 and Smad3.	MIM's coord. 5, A
54	**pRb**	The retinoblastoma protein is a member of the pRB family. The protein is a negative regulator of the cell cycle. The active, hypo-phosphorylated form of the protein binds transcription factor E2F1. Tumor suppressor gene.	MIM's coord. 7, N-O
55	***PTEN***	**P**hosphatase and **ten**sin homologue, is a lipid phosphatase and is a ubiquitous regulator of the cellular PI3K signaling pathway. The gene is located on chromosome 10, it can be mutated by deletion, but it can also be hypo-expressed.	MIM's coord. 3,N
56	***PTP1B***	**P**rotein **T**yrosine **P**hospatase **1B** is activated as a hetero-dimer with Cadherin and works as a phosphatase of Y654 of β-catenin.	MIM's coord. 2,D
57	***PTPε***	**P**rotein **t**yrosine **p**hosphatase **ε** belongs to a structurally diverse superfamily containing several dozens of membrane-bound or unbound enzymes.	MIM's coord. 3,G
58	**Shc**	**S**rc **h**omology and **c**ollagen-containing protein is an adaptor protein: it plays key roles in signaling downstream of Erb family receptors.	MIM's coord. 3,G
59	**Smad2**	Belong to a subclass of Smads called receptor regulated Smads (R-Smad) and are transcription factors; these serve principally as substrates for the TGFβ receptors. The name Smad (mothers against decapentaplegic homolog) was coined in reference to identification of Smad1 and its sequence similarity to the Sma (the orthologs in C. elegans) and Mad (the orthologs in Drosophila) proteins.	MIM's coord. 2-3,A-B
60	**Smad3**	Belong to a subclass of Smads called receptor regulated Smads (R-Smad) and are transcription factors; these serve principally as substrates for the TGFβ receptors. The name Smad (mothers against decapentaplegic homolog) was coined in reference to identification of Smad1 and its sequence similarity to the Sma (the orthologs in C. elegans) and Mad (the orthologs in Drosophila) proteins.	MIM's coord. 2-3,A-B

61	**Smad4**	Belongs to a subclass of Smads called co-mediator-Smad (co-Smad) and is a transcription factors.	MIM's coord. 2,C
62	**SOS**	**S**on **o**f **S**evenless drosophila homolog protein; is a guanine nucleotide exchange factor (GEF) that facilitates dissociation and exchange of bound nucleotides from KRAS. It typically favors the transition from KRAS-GDP to KRAS-GTP.	MIM's coord. 2-3,H
63	**TAB2**	**TA**K1-**b**inding protein **2** is a scaffold protein required in several TAK-1 functions.	MIM's coord. 3,D
64	<u>TAK-1</u>	TGF-β activated kinase 1, also known as MEKK7, is a mitogen- activated protein-kinase-kinase kinase (MAP3K). It was originally identified as a kinase involved in TGF-β signaling.	MIM's coord. 3,C
65	**TCF7L2**	TCF7L2 (**t**ranscription **f**actor **7**-**l**ike **2** (T-cell specific, HMG-box)) also know[illegible] as TCF4 is a protein acting as a transcription factor. TCF7L2 influencin[illegible] the transcription of several genes thereby exerting a large variety of function[illegible] within the cell.	MIM's coord. 5,C-D
66	**TGFβ**	The **T**ransforming **G**rowth **F**actor **Beta** is a member of the TGFβ superfamily of more 30 factors of multifunctional cytokine that regulates biological responses as cell growth, cell cycle progression, differentiation, adhesion, migration and death of target cells, in a developmental context-dependent and cell type-specific manner. Dysfunction of TGFβ signaling has been implicated in cancer progression: Because TGFβ signaling generally has a negative effect on cell growth, inactivation of this pathway contributes to tumorigenesis.	MIM's coord. 1,B-C
67	<u>TGFβR-I/ TGFβR-II</u>	The effects of TGFβ are mediated through type I and type II receptors, which are transmembrane proteins possessing cytoplasmic serine/threonine kinase domains for signal propagation.	MIM's coord. 1-2,B-C
68	**TP53**	TP53 is a transcription factor whose protein levels and post-translationa[illegible] modification state alter in response to cellular stress. Acts as a tumor suppresso[illegible] in many tumor types; induces growth arrest or apoptosis depending on th[illegible] physiological circumstances and cell type.	MIM's coord. 5, N
69	**Wnt**	***Wingless/Int-1*** (Wnt) is a large family of 19 human highly conserved secreted glycoproteins that play fundamental roles in controlling cell proliferation, cell-fate determination, and differentiation during embryonic development and adult homeostasis.	MIM's coord. 1, E

Oncoprotein inhibitors			
	**AZAKENPAULLONE**	1-Azakenpaullone is a selective inhibitor of glycogen synthase kinase 3β (GSK3β) with 100-fold less cross-reactivity against CDKs.	
	**CI-1040**	CI-1040 (PD184352) is an ATP non-competitive MEK1/2 inhibitor. CI-1040 is an highly specific, small-molecule inhibitor of one of the key components of the MAPK pathway (MEK1/MEK2), and thereby effectively blocks the phosphorylation of ERK and continued signal transduction through this pathway.	
	**PERIFOSINE**	Perifosine is an alkyl-phosphocholine compound which greatly hampers translocation of Akt to the cell membrane, where Thr308- and Ser473-directed kinases normally activate Akt. Perifosine is undergoing its Phase III clinical trials for the treatment of refractory multiple myeloma, in combination with a placebo, and is in Phase II trials for several other cancers.	
	**PI103**	PI-103 is a potent, ATP-competitive PI3K inhibitor of DNA-PK, p110α, mTORC1, PI3KC2β, p110δ, mTORC2, p110β, and p110γ.	
	**XAV939**	XAV939 is a trifluoromethylphenylpyrimidine derivative. XAV939 can prolong the half-life of axin and promote β-catenin degradation through inhibiting tankyrase. TNKS1 and TNKS2 modify axin substrate through the addition of several ADP-ribose units, referred to as poly-ADP-ribosylation (PARsylation).	

http://www.ncbi.nlm.nih.gov/gene/

http://www.genecards.org/

http://www.uniprot.org

Fact Sheet of the inhibitor drugs

References in the Annotation List

Supplementary Figure 1.3 – Molecular Interaction Map (MIM) referred to *HT29*

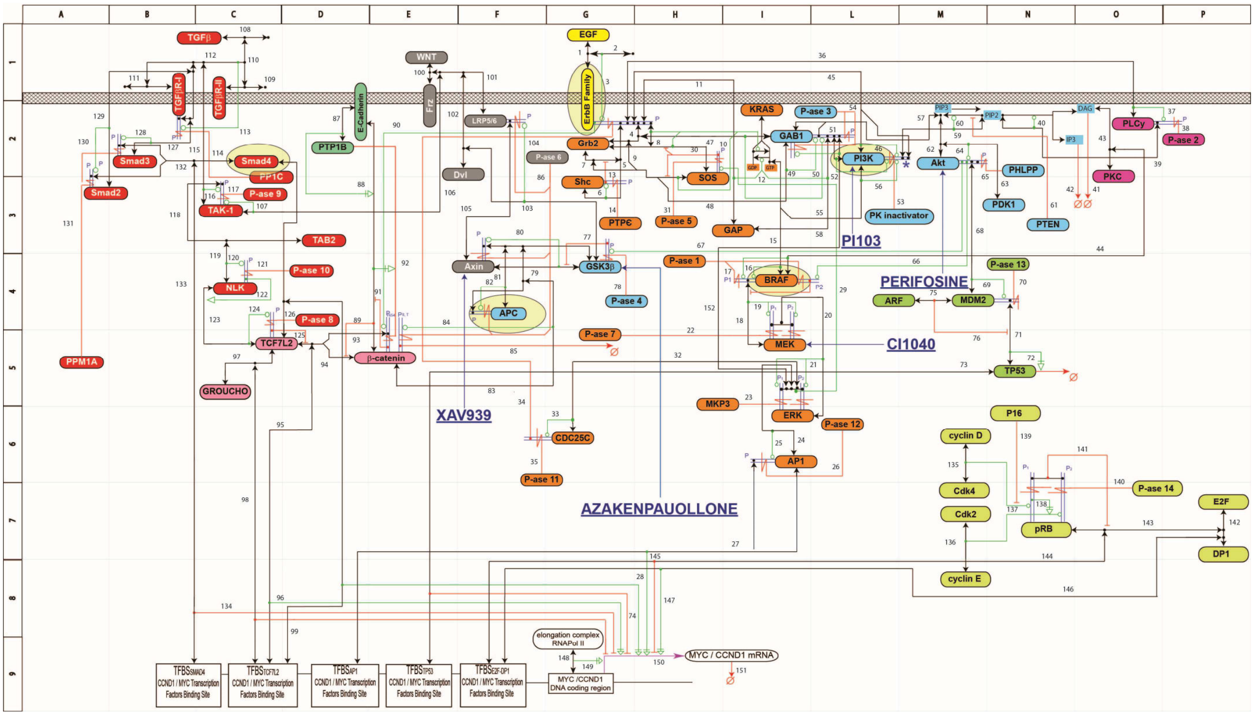

Supplementary Fig. (1.3): **Molecular Interaction Map (MIM) referred to *HT29*.** Molecular Interaction Map (MIM) referring to the pathways downstream of the TGFβ-family, WNT-family and EGF-family proteins, pathways which are relevant in colorectal cancer. The cartouches of mutated / altered signaling-proteins in the HT29 cell line have been surrounded by an oval.

**Supplementary Table 1.4** - Pathways present in our MIMs	
**1**	ErbB-family receptors – PI3K – PTEN – Akt – GSK3β – APC – β-catenin – TCF7L2 — $TFBS_{TCF7L2}$ (TCF7L2 binding site), transcription agonist
**2**	ErbB-family receptors – Grb2 – Shc – SOS– GAP– KRAS – BRAF – MEK – ERK- AP1 – AP1 binding site – $TFBS_{AP1}$, transcription agonist
**3**	ErbB-family receptors-E-Cadherin (Cadherin/Catenin adhesive complex);
**4**	ErbB-family receptors – PLCγ – PIP2 – PKC – BRAF – MEK – ERK - AP1– $TFBS_{AP1}$ (AP1 binding sites), transcription agonist, (the terminal parts of pathway 2 and 4 are the same)
**5**	WNT – Frz/LRP5/6 – Dvl – AXIN – APC – GSK3β – β-catenin – TCF7L2 — $TFBS_{TCF7L2}$
**6**	TGFβ-receptors – SMAD2/3 – SMAD4 – $TFBS_{SMAD4}$ (SMAD4 binding site), transcription antagonist
**7**	TGFβ-receptors – TAK-1 – TAB2 – NLK – TCF7L2 –$TFBS_{TCF7L2}$, converging with 8
**8**	WNT – Frz/LRP5/6 – TAK-1 – TAB2 – NLK – TCF7L2 — $TFBS_{TCF7L2}$, converging with 7
**9**	Akt – MDM2 – TP53 – $TFBS_{TP53}$ (TP53 binding site)
**10**	Cyclin (D/E) / CDK (2/4) – pRB – E2F:DP – $TFBS_{E2F:DP1}$ (E2F:DP1 binding site).

Supplementary Table 1.4: Simplified description of the pathways involved in our MIMs

Supplementary Table 2.1 - Reaction list

Supplementary Table 2.1 shows a list of 348 protein-protein-interaction reactions (348 association reactions + 348 dissociation reactions) and 174 catalytic reactions, rate-constants included, which represent the complete set of our dynamic simulations. 348 + 348 + 174 gives a total of 870 reactions.

We derived our list of reactions from the literature. The references associated with the Supplementary Material 1.1 - Annotation List give information about all the interactions described in our MIM. These references were utilized for preliminary extrapolations of kinetic parameters, in a patient patchwork of readjustments, to reconstruct a reasonable global dynamic behavior. The references associated with the Annotation List are the main direct / indirect source of the concentrations and reactions data involved in training the modeling of our MIM. Additional references are listed below.

	**Chemical Reactions**	**Kinetic costants**	**Reaction Rates**	**Kinetic costant values**	**Units**	**Extrapolations from Annotation List References (*S1.1*) + Notes / Ref. listed below**
**1**	R + EGF <-> Rl	c1f	c1f*R*EGF - c1r*Rl	0.01	$nM^{-1}\cdot s^{-1}$	1 - 3 (*S1.1*) + [1-8]
**2**	R + EGF <-> Rl	c1r		1	s^{-1}	1 - 3 (*S1.1*) + [1-8]
**3**	Rl + Rl <-> Rl2	c2f	c2f*Rl*Rl - c2r*Rl2	0.01	$nM^{-1}\cdot s^{-1}$	1 – 3 (*S1.1*) + [1-8]
**4**	Rl + Rl <-> Rl2	c2r		0.1	s^{-1}	1 - 3 (*S1.1*) + [1-8]
**5**	Rl2 -> Rl2P	c3f	c3f*Rl2	1	s^{-1}	1 - 3 (*S1.1*) + [1-8]
**6**	RP + PLCy <-> RP_PL	c5f	c5f*RP*PLCy - c5r*RP_PL	0.06	$nM^{-1}\cdot s^{-1}$	1 - 3 (*S1.1*) + [1-8]
**7**	RP + PLCy <-> RP_PL	c5r		0.2	s^{-1}	1 - 3 (*S1.1*) + [1-8]
**8**	RP_PL -> RP_PLP	c6f	c6f*RP_PL	1	s^{-1}	1 -3 (*S1.1*) + [1-8]
**9**	RP_PLP <-> RP + PLCyP	c7f		0.3	s^{-1}	1 - 3 (*S1.1*) + [1-8]
**10**	RP_PLP <-> RP + PLCyP	c7r	c7f*RP_PLP - c7r*RP*PLCyP	0.006	$nM^{-1}\cdot s^{-1}$	1 - 3 (*S1.1*) + [1-8]
**11**	RP + Grb <-> RP_G	c10f	c10f*RP*Grb - c10r*RP_G	0.0015	$nM^{-1}\cdot s^{-1}$	1 - 5 (*S1.1*) + [1-9]
**12**	RP + Grb <-> RP_G	c10r		0.2	s^{-1}	1 - 5 (*S1.1*) + [1-9]
**13**	RP_G + SOS <-> RP_G_S	c11f	c11f*RP_G*SOS - c11r*RP_G_S	0.01	$nM^{-1}\cdot s^{-1}$	1 - 6 (*S1.1*) + [1-9]
**14**	RP_G + SOS <-> RP_G_S	c11r		0.06	s^{-1}	1 - 6 (*S1.1*) + [1-9]
**15**	RP_G_S <-> RP + G_S	c12f	c12f*RP_G_S - c12r*RP*G_S	0.15	s^{-1}	1 - 6 (*S1.1*) + [1-9]

**16**	RP_G_S <-> RP + G_S	c12r		0.0028	$nM^{-1}\cdot s^{-1}$	1 - 6 (*S1.1*) + [1-9]
**17**	G_S <-> Grb + SOS	c13f	c13f*G_S - c13r*Grb*SOS	0.0015	s^{-1}	1 - 6 (*S1.1*) + [1-9]
**18**	G_S <-> Grb + SOS	c13r		0.0001	$nM^{-1}\cdot s^{-1}$	1 - 6 (*S1.1*) + [1-9]
**19**	RP + Shc <-> RP_Sh	c14f	c14f*RP*Shc - c14r*RP_Sh	0.09	$nM^{-1}\cdot s^{-1}$	1 - 5 (*S1.1*) + [1-9]
**20**	RP + Shc <-> RP_Sh	c14r		0.6	s^{-1}	1 - 5 (*S1.1*) + [1-9]
**21**	RP_Sh -> RP_ShP	c15	c15*RP_Sh	6	s^{-1}	1 – 5 (*S1.1*) + [1-9]
**22**	RP_ShP <-> ShP + RP	c16f		0.3	s^{-1}	1 - 5 (*S1.1*) + [1-9]
**23**	RP_ShP <-> ShP + RP	c16r	c16f*RP_ShP - c16r*ShP*RP	0.0009	$nM^{-1}\cdot s^{-1}$	1 - 5 (*S1.1*) + [1-9]
**24**	RP_ShP + Grb <-> RP_ShP_G	c17f	c17f*RP_ShP*Grb - c17r*RP_ShP_G	0.003	$nM^{-1}\cdot s^{-1}$	1 - 5 (*S1.1*) + [1-9]
**25**	RP_ShP + Grb <-> RP_ShP_G	c17r		0.1	s^{-1}	1 - 5 (*S1.1*) + [1-9]
**26**	RP_ShP_G <-> RP + ShP_G	c18f	c18f*RP_ShP_G - c18r*RP*ShP_G	0.3	s^{-1}	1 - 5 (*S1.1*) + [1-9]
**27**	RP_ShP_G <-> RP + ShP_G	c18r		0.0009	$nM^{-1}\cdot s^{-1}$	1 - 5 (*S1.1*) + [1-9]
**28**	RP_ShP_G + SOS <-> RP_ShP_G_S	c19f	c19f*RP_ShP_G*SOS - c19r*RP_ShP_G_S	0.01	$nM^{-1}\cdot s^{-1}$	1 - 6 (*S1.1*) + [1-9]
**29**	RP_ShP_G + SOS <-> RP_ShP_G_S	c19r		0.0214	s^{-1}	1 - 6 (*S1.1*) + [1-9]
**30**	RP_ShP_G_S <-> ShP_G_S + RP	c20f	c20f*RP_ShP_G_S - c20r*ShP_G_S*RP	0.12	s^{-1}	1 - 6 (*S1.1*) + [1-9]
**31**	RP_ShP_G_S <-> ShP_G_S + RP	c20r		0.00024	$nM^{-1}\cdot s^{-1}$	1 - 6, 9 (*S1.1*) + [1-9]
**32**	ShP_G + SOS <-> ShP_G_S	c21f	c21f*ShP_G*SOS - c21r*ShP_G_S	0.03	$nM^{-1}\cdot s^{-1}$	4 - 6, 9, 24 (*S1.1*) + [1-9]
**33**	ShP_G + SOS <-> ShP_G_S	c21r		0.064	s^{-1}	4 - 6, 9, 24 (*S1.1*) + [1-9]
**34**	ShP + Grb <-> ShP_G	c22f	c22f*ShP*Grb - c22r*ShP_G	0.003	$nM^{-1}\cdot s^{-1}$	4 - 6, 9, 24 (*S1.1*) + [1-9]
**35**	ShP + Grb <-> ShP_G	c22r		0.1	s^{-1}	4 - 6, 9, 24 (*S1.1*) + [1-9]
**36**	ShP_G_S <-> ShP + G_S	c24f	c24f*ShP_G_S - c24r*ShP*G_S	0.1	s^{-1}	4 - 6, 9, 24 (*S1.1*) + [1-9]
**37**	ShP_G_S <-> ShP + G_S	c24r		0.021	$nM^{-1}\cdot s^{-1}$	4 - 6, 9, 24 (*S1.1*) + [1-9]
**38**	RP_ShP + G_S <-> RP_ShP_G_S	c25f	c25f*RP_ShP*G_S - c25r*RP_ShP_G_S	0.009	$nM^{-1}\cdot s^{-1}$	1 - 6, 9, 24 (*S1.1*) + [1-9]
**39**	RP_ShP + G_S <-> RP_ShP_G_S	c25r		0.0429	s^{-1}	1 - 6, 9, 24 (*S1.1*) + [1-9]
**40**	Ras_GDP <-> Ras + GDP	c26f	c26f*Ras_GDP - c26r*Ras*GDP	0.0000054	s^{-1}	1 - 8 (*S1.1*) + [1-11]
**41**	Ras_GDP <-> Ras + GDP	c26r		0.00027	$nM^{-1}\cdot s^{-1}$	1 - 8 (*S1.1*) + [1-11]
**42**	Ras + GTP <-> Ras_GTP	c27f	c27f*Ras*GTP - c27r*Ras_GTP	0.078	$nM^{-1}\cdot s^{-1}$	1 - 8 (*S1.1*) + [1-11]
**43**	Ras + GTP <-> Ras_GTP	c27r		0.00078	s^{-1}	1- 8 (*S1.1*) + [1-11]
**44**	Ras_GTP -> Ras_GDP	c28	c28*Ras_GTP	0.00001	s^{-1}	1 - 8 (*S1.1*) + [1-11]
**45**	RP_ShP_G_S + Ras_GDP <-> RP_ShP_G_S_Ras_GDP	c29f	c29f*RP_ShP_G_S*Ras_GDP - c29r*RP_ShP_G_S_Ras_GDP	0.00475	$nM^{-1}\cdot s^{-1}$	1 - 8 (*S1.1*) + [1-11]

**46**	RP_ShP_G_S + Ras_GDP <-> RP_ShP_G_S_Ras_GDP	c29r		0.76	s^{-1}	1 - 8 (*S1.1*) + [1-11]
**47**	RP_ShP_G_S_Ras_GDP <-> RP_ShP_G_S_Ras + GDP	c30f	c30f*RP_ShP_G_S_Ras_GDP - c30r*RP_ShP_G_S_Ras*GDP	46.5	s^{-1}	1 - 8 (*S1.1*) + [1-11]
**48**	RP_ShP_G_S_Ras_GDP <-> RP_ShP_G_S_Ras + GDP	c30r		0.093	$nM^{-1}\cdot s^{-1}$	1 - 8 (*S1.1*) + [1-11]
**49**	RP_ShP_G_S_Ras + GTP <-> RP_ShP_G_S_Ras_GTP	c31f	c31f*RP_ShP_G_S_Ras*GTP - c31r*RP_ShP_G_S_Ras_GTP	0.003	$nM^{-1}\cdot s^{-1}$	1 - 8 (*S1.1*) + [1-11]
**50**	RP_ShP_G_S_Ras + GTP <-> RP_ShP_G_S_Ras_GTP	c31r		2.4	s^{-1}	1 - 8 (*S1.1*) + [1-11]
**51**	RP_ShP_G_S_Ras_GTP <-> RP_ShP_G_S + Ras_GTP	c32f	c32f*RP_ShP_G_S_Ras_GTP - c32r*RP_ShP_G_S*Ras_GTP	806.4	s^{-1}	1 - 8 (*S1.1*) + [1-11]
**52**	RP_ShP_G_S_Ras_GTP <-> RP_ShP_G_S + Ras_GTP	c32r		1.575	$nM^{-1}\cdot s^{-1}$	1 - 8 (*S1.1*) + [1-11]
**53**	RP_ShP_G_S + Ras <-> RP_ShP_G_S_Ras	c33f	c33f*RP_ShP_G_S*Ras - c33r*RP_ShP_G_S_Ras	0.15625	$nM^{-1}\cdot s^{-1}$	1 - 8 (*S1.1*) + [1-11]
**54**	RP_ShP_G_S + Ras <-> RP_ShP_G_S_Ras	c33r		0.001	s^{-1}	1 - 8 (*S1.1*) + [1-11]
**55**	RP_G_S + Ras_GDP <-> RP_G_S_Ras_GDP	c34f	c34f*RP_G_S*Ras_GDP - c34r*RP_G_S_Ras_GDP	0.0075	$nM^{-1}\cdot s^{-1}$	1 - 8 (*S1.1*) + [1-11]
**56**	RP_G_S + Ras_GDP <-> RP_G_S_Ras_GDP	c34r		1.2	s^{-1}	1 - 8 (*S1.1*) + [1-11]
**57**	RP_G_S_Ras_GDP <-> RP_G_S_Ras + GDP	c35f	c35f*RP_G_S_Ras_GDP - c35r*RP_G_S_Ras*GDP	50	s^{-1}	1 - 8 (*S1.1*) + [1-11]
**58**	RP_G_S_Ras_GDP <-> RP_G_S_Ras + GDP	c35r		0.1	$nM^{-1}\cdot s^{-1}$	1 - 8 (*S1.1*) + [1-11]
**59**	RP_G_S_Ras + GTP <-> RP_G_S_Ras_GTP	c36f	c36f*RP_G_S_Ras*GTP - c36r*RP_G_S_Ras_GTP	0.1	$nM^{-1}\cdot s^{-1}$	1 - 8 (*S1.1*) + [1-11]
**60**	RP_G_S_Ras + GTP <-> RP_G_S_Ras_GTP	c36r		80	s^{-1}	1 - 8 (*S1.1*) + [1-11]
**61**	RP_G_S_Ras_GTP <-> RP_G_S + Ras_GTP	c37f	c37f*RP_G_S_Ras_GTP - c37r*RP_G_S*Ras_GTP	640	s^{-1}	1 - 8 (*S1.1*) + [1-11]
**62**	RP_G_S_Ras_GTP <-> RP_G_S + Ras_GTP	c37r		1.25	$nM^{-1}\cdot s^{-1}$	1 - 8 (*S1.1*) + [1-11]
**63**	RP_G_S + Ras <-> RP_G_S_Ras	c38f	c38f*RP_G_S*Ras - c38r*RP_G_S_Ras	0.25	$nM^{-1}\cdot s^{-1}$	1 - 8 (*S1.1*) + [1-11]
**64**	RP_G_S + Ras <-> RP_G_S_Ras	c38r		0.0016	s^{-1}	1 - 8 (*S1.1*) + [1-11]
**65**	Raf + Ras_GTP <-> Raf_Ras_GTP	c39f	c39f*Raf*Ras_GTP - c39r*Raf_Ras_GTP	0.01	$nM^{-1}\cdot s^{-1}$	6 - 8, 11 - 14 (*S1.1*) + [1-11]
**66**	Raf + Ras_GTP <-> Raf_Ras_GTP	c39r		0.0053	s^{-1}	6 - 8, 11 - 14 (*S1.1*) + [1-11]
**67**	Raf_Ras_GTP -> [Raf) + Ras_GTP	c40f	c40f*Raf_Ras_GTP	1	s^{-1}	6 - 8, 11 - 14 (*S1.1*) + [1-11]
**68**	[Raf) + Pase1 <-> [Raf_Pase1)	c41f	c41f*[Raf*)*Pase1 - c41r*[Raf*_Pase1)	0.0717	$nM^{-1}\cdot s^{-1}$	11 – 14 (*S1.1*) + [1,4-9]
**69**	[Raf) + Pase1 <-> [Raf_Pase1)	c41r		0.2	s^{-1}	11 – 14 (*S1.1*) + [1,4-9]
**70**	[Raf_Pase1) -> Raf + Pase1	c42	c42*[Raf*_Pase1)	1	s^{-1}	11 – 14 (*S1.1*) + [1,4-9]
**71**	MEK + [Raf) <-> [MEK_Raf)	c43f		0.01	$nM^{-1}\cdot s^{-1}$	11 – 14 (*S1.1*) + [1,4-9]

**72**	MEK + [Raf) <-> [MEK_Raf)	c43r	c43f*MEK*[Raf*) - c43r*[MEK_Raf*)	0.1	s^{-1}	11 – 14 (*S1.1*) + [1,4-9]
**73**	[MEK_Raf) -> [Raf) + MEKP	c44	c44*[MEK_Raf*)	1	s^{-1}	11 – 14 (*S1.1*) + [1,4-9]
**74**	MEKP + [Raf) <-> [MEKP_Raf)	c45f	c45f*MEKP*[Raf*) - c45r*[MEKP_Raf*)	0.01	$nM^{-1}\cdot s^{-1}$	11 – 14 (*S1.1*) + [1,4-9]
**75**	MEKP + [Raf) <-> [MEKP_Raf)	c45r		0.1	s^{-1}	11 – 14 (*S1.1*) + [1,4-9]
**76**	[MEKP_Raf) -> MEKPP + [Raf)	c46	c46*[MEKP_Raf*)	1	s^{-1}	11 – 14 (*S1.1*) + [1,4-9]
**77**	MEKPP + Pase2 <-> MEKPP_Pase2	c47f	c47f*MEKPP*Pase2 - c47r*MEKPP_Pase2	0.01	$nM^{-1}\cdot s^{-1}$	11 – 14 (*S1.1*) + [1,4-9]
**78**	MEKPP + Pase2 <-> MEKPP_Pase2	c47r		0.1	s^{-1}	11 – 14 (*S1.1*) + [1,4-9]
**79**	MEKPP_Pase2 -> MEKP + Pase2	c48	c48*MEKPP_Pase2	0.1	s^{-1}	11 – 14 (*S1.1*) + [1,4-9]
**80**	MEKP + Pase2 <-> MEKP_Pase2	c49f	c49f*MEKP*Pase2 - c49r*MEKP_Pase2	0.01	$nM^{-1}\cdot s^{-1}$	11 – 14 (*S1.1*) + [1,4-9]
**81**	MEKP + Pase2 <-> MEKP_Pase2	c49r		0.1	s^{-1}	11 – 14 (*S1.1*) + [1,4-9]
**82**	MEKP_Pase2 -> MEK + Pase2	c50	c50*MEKP_Pase2	0.1	s^{-1}	11 – 14 (*S1.1*) + [1,4-9]
**83**	ERK + MEKPP <-> ERK_MEKPP	c51f	c51f*ERK*MEKPP - c51r*ERK_MEKPP	0.01	$nM^{-1}\cdot s^{-1}$	11 – 14 (*S1.1*) + [1,4-9]
**84**	ERK + MEKPP <-> ERK_MEKPP	c51r		0.0033	s^{-1}	11 – 14 (*S1.1*) + [1,4-9]
**85**	ERK_MEKPP -> ERKP + MEKPP	c52	c52*ERK_MEKPP	16	s^{-1}	11 – 14 (*S1.1*) + [1,4-9]
**86**	ERKP + MEKPP <-> ERKP_MEKPP	c53f	c53f*ERKP*MEKPP - c53r*ERKP_MEKPP	0.01	$nM^{-1}\cdot s^{-1}$	11 – 14 (*S1.1*) + [1,4-9]
**87**	ERKP + MEKPP <-> ERKP_MEKPP	c53r		0.0033	s^{-1}	11 – 14 (*S1.1*) + [1,4-9]
**88**	ERKP_MEKPP -> ERKPP + MEKPP	c54	c54*ERKP_MEKPP	5.7	s^{-1}	11 – 14 (*S1.1*) + [1,4-9]
**89**	ERKPP + Pase3 <-> ERKPP_Pase3	c55f	c55f*ERKPP*Pase3 - c55r*ERKPP_Pase3	0.0145	$nM^{-1}\cdot s^{-1}$	11 – 14 (*S1.1*) + [1,4-9]
**90**	ERKPP + Pase3 <-> ERKPP_Pase3	c55r		0.6	s^{-1}	11 – 14 (*S1.1*) + [1,4-9]
**91**	ERKPP_Pase3 -> ERKP + Pase3	c56	c56*ERKPP_Pase3	0.27	s^{-1}	11 – 14 (*S1.1*) + [1,4-9]
**92**	ERKP + Pase3 <-> ERKP_Pase3	c57f	c57f*ERKP*Pase3 - c57r*ERKP_Pase3	0.05	$nM^{-1}\cdot s^{-1}$	11 – 14 (*S1.1*) + [1,4-9]
**93**	ERKP + Pase3 <-> ERKP_Pase3	c57r		0.5	s^{-1}	11 – 14 (*S1.1*) + [1,4-9]
**94**	ERKP_Pase3 -> ERK + Pase3	c58	c58*ERKP_Pase3	0.3	s^{-1}	11 – 14 (*S1.1*) + [1,4-9]
**95**	GAP + RP <-> RP_GAP	c59f	c59f*GAP*RP - c59r*RP_GAP	0.083	$nM^{-1}\cdot s^{-1}$	6 - 8 (*S1.1*) + [1,5,11]
**96**	GAP + RP <-> RP_GAP	c59r		0.15	s^{-1}	6 - 8 (*S1.1*) + [1,5,11]
**97**	Ras_GTP + RP_GAP <-> RP_GAP_Ras_GTP	c60f	c60f*Ras_GTP*RP_GAP - c60r*RP_GAP_Ras_GTP	0.01	$nM^{-1}\cdot s^{-1}$	6 - 8 (*S1.1*) + [1,5,11]
**98**	Ras_GTP + RP_GAP <-> RP_GAP_Ras_GTP	c60r		0.03	s^{-1}	6 - 8 (*S1.1*) + [1,5,11]
**99**	RP_GAP_Ras_GTP -> RP_GAP + Ras_GDP	c61	c61*RP_GAP_Ras_GTP	1.494	s^{-1}	6 - 8 (*S1.1*) + [1,5,11]
**100**	RP_ShP_G_S + ERKPP <-> RP_ShP_G_S_ERKPP	c70f	c70f*RP_ShP_G_S*ERKPP - c70r*RP_ShP_G_S_ERKPP	0.01	$nM^{-1}\cdot s^{-1}$	11, 24, 25 (*S1.1*) + [1,5,12-13]

**101**	RP_ShP_G_S + ERKPP <-> RP_ShP_G_S_ERKPP	c70r		0.033	s^{-1}	11, 24, 25 (*S1.1*) + [1,5,12-13]
**102**	RP_ShP_G_S_ERKPP -> RP_ShP_G + ERKPP + SOSP	c71	c71*RP_ShP_G_S_ERKPP	1	s^{-1}	11, 24, 25 (*S1.1*) + [1,5,12-13]
**103**	RP_G_S + ERKPP <-> RP_G_S_ERKPP	c72f	c72f*RP_G_S*ERKPP - c72r*RP_G_S_ERKPP	0.01	$nM^{-1}\cdot s^{-1}$	11, 24, 25 (*S1.1*) + [1,5,12-13]
**104**	RP_G_S + ERKPP <-> RP_G_S_ERKPP	c72r		0.033	s^{-1}	11,24, 25 (*S1.1*) + [1,5,12-13]
**105**	RP_G_S_ERKPP -> RP_G + SOSP + ERKPP	c73	c73*RP_G_S_ERKPP	1	s^{-1}	11, 24, 25 (*S1.1*) + [1,5,12-13]
**106**	SOSP + Pase5 <-> SOSP_Pase5	c74f	c74f*SOSP*Pase5 - c74r*SOSP_Pase5	0.01	$nM^{-1}\cdot s^{-1}$	11, 24 - 25 (*S1.1*) + [1,5,13]
**107**	SOSP + Pase5 <-> SOSP_Pase5	c74r		0.1	s^{-1}	11, 24 - 25 (*S1.1*) + [12-13]
**108**	SOSP_Pase5 -> SOS + Pase5	c75	c75*SOSP_Pase5	1	s^{-1}	11, 24 - 25 (*S1.1*) + [12-13]
**109**	RP_G + GAB <-> RP_G_GAB	c76f	c76f*RP_G*GAB - c76r*RP_G_GAB	0.01	$nM^{-1}\cdot s^{-1}$	33 (*S1.1*) + [1,9]
**110**	RP_G + GAB <-> RP_G_GAB	c76r		1	s^{-1}	33 (*S1.1*) + [1,9]
**111**	RP_ShP_G + GAB <-> RP_ShP_G_GAB	c77f	c77f*RP_ShP_G*GAB - c77r*RP_ShP_G_GAB	0.01	$nM^{-1}\cdot s^{-1}$	33 (*S1.1*) + [1,9]
**112**	RP_ShP_G + GAB <-> RP_ShP_G_GAB	c77r		1	s^{-1}	33 (*S1.1*) + [1,9]
**113**	RP_G_GAB -> RP_G_GABP	c78	c78*RP_G_GAB	0.05	s^{-1}	33 (*S1.1*) + [1,9]
**114**	RP_ShP_G_GAB -> RP_ShP_G_GABP	c79	c79*RP_ShP_G_GAB	0.05	s^{-1}	33 (*S1.1*) + [1,9]
**115**	RP_G_GABP + PI3K <-> RP_G_GABP_PK	c80f	c80f*RP_G_GABP*PI3K - c80r*RP_G_GABP_PK	0.01	$nM^{-1}\cdot s^{-1}$	31 - 33 (*S1.1*) + [1,9]
**116**	RP_G_GABP + PI3K <-> RP_G_GABP_PK	c80r		1	s^{-1}	31 - 33 (*S1.1*) + [12-13]
**117**	RP_G_GABP_PK -> RP_G_GABP_PKP	c82	c82*RP_G_GABP_PK	1	s^{-1}	31 - 33 (*S1.1*) + [12-13]
**118**	RP_ShP_G_GABP + PI3K <-> RP_ShP_G_GABP_PK	c81f	c81f*RP_ShP_G_GABP*PI3K - c81r*RP_ShP_G_GABP_PK	0.01	$nM^{-1}\cdot s^{-1}$	31 - 33 (*S1.1*) + [12-13]
**119**	RP_ShP_G_GABP + PI3K <-> RP_ShP_G_GABP_PK	c81r		1	s^{-1}	31 - 33 (*S1.1*) + [12-13]
**120**	RP_ShP_G_GABP_PK -> RP_ShP_G_GABP_PKP	c83	c83*RP_ShP_G_GABP_PK	1	s^{-1}	31 - 33 (*S1.1*) + [12-13]
**121**	RP_ShP_G_GABP + GAP <-> RP_ShP_G_GABP_GAP	c84f	c84f*RP_ShP_G_GABP*GAP - c84r*RP_ShP_G_GABP_GAP	0.083	$nM^{-1}\cdot s^{-1}$	33 (*S1.1*) + [12-13]
**122**	RP_ShP_G_GABP + GAP <-> RP_ShP_G_GABP_GAP	c84r		0.15	s^{-1}	33 (*S1.1*) + [1,5,11]
**123**	RP_G_GABP + GAP <-> RP_G_GABP_GAP	c85f	c85f*RP_G_GABP*GAP - c85r*RP_G_GABP_GAP	0.083	$nM^{-1}\cdot s^{-1}$	33 (*S1.1*) + [1,5,11]
**124**	RP_G_GABP + GAP <-> RP_G_GABP_GAP	c85r		0.15	s^{-1}	33 (*S1.1*) + [1,5,11]

**125**	RP_G_GABP_GAP + Ras_GTP <-> RP_G_GABP_GAP_Ras_GTP	c86f	c86f*RP_G_GABP_GAP*Ras_GTP - c86r*RP_G_GABP_GAP_Ras_GTP	0.6225	$nM^{-1}\cdot s^{-1}$	7, 8, 33 (*S1.1*) + [1,5,11]
**126**	RP_G_GABP_GAP + Ras_GTP <-> RP_G_GABP_GAP_Ras_GTP	c86r		0.3	s^{-1}	7, 8, 33 (*S1.1*) + [1,5,11]
**127**	RP_G_GABP_GAP_Ras_GTP -> RP_G_GABP_GAP + Ras_GDP	c87	c87*RP_G_GABP_GAP_Ras_GTP	1.494	s^{-1}	7, 8, 33 (*S1.1*) + [1,5,11]
**128**	RP_ShP_G_GABP_GAP + Ras_GTP <-> RP_ShP_G_GABP_GAP_Ras_GTP	c88f	c88f*RP_ShP_G_GABP_GAP*Ras_GTP - c88r*RP_ShP_G_GABP_GAP_Ras_GTP	0.6225	$nM^{-1}\cdot s^{-1}$	7, 8, 33 (*S1.1*) + [1,5,11]
**129**	RP_ShP_G_GABP_GAP + Ras_GTP <-> RP_ShP_G_GABP_GAP_Ras_GTP	c88r		0.3	s^{-1}	7, 8, 33 (*S1.1*) + [1,5,11]
**130**	RP_ShP_G_GABP_GAP_Ras_GTP -> RP_ShP_G_GABP_GAP + Ras_GDP	c89	c89*RP_ShP_G_GABP_GAP_Ras_GTP	1.494	s^{-1}	7, 8, 33 (*S1.1*) + [1,5,11]
**131**	RP + PI3K <-> RP_PK	c90f	c90f*RP*PI3K - c90r*RP_PK	0.01	$nM^{-1}\cdot s^{-1}$	1 – 3, 31, 32 (*S1.1*) + [12-13]
**132**	RP + PI3K <-> RP_PK	c90r		1	s^{-1}	1 – 3, 31, 32 (*S1.1*) + [12-13]
**133**	RP_PK -> RP_PKP	c91	c91*RP_PK	1	s^{-1}	1 – 3, 31, 32 (*S1.1*) + [12-13]
**134**	RP + PKP <-> RP_PKP	c92r	c92f*RP*PKP - c92r*RP_PKP	0.1	s^{-1}	1 – 3, 31, 32 (*S1.1*) + [12-13]
**135**	RP + PKP <-> RP_PKP	c92f		0.01	$nM^{-1}\cdot s^{-1}$	1 – 3, 31, 32 (*S1.1*) + [12-13]
**136**	PIP3 + PTEN <-> PIP3_PTEN	c93f	c93f*PIP3*PTEN - c93r*PIP3_PTEN	0.01	$nM^{-1}\cdot s^{-1}$	31, 32 (*S1.1*) + [1-9]
**137**	PIP3 + PTEN <-> PIP3_PTEN	c93r		0.01	s^{-1}	31, 32 (*S1.1*)) + [1-9]
**138**	PIP3_PTEN -> PIP2 + PTEN	c94	c94*PIP3_PTEN	20	s^{-1}	31, 32 (*S1.1*) + [1-9]
**139**	PKP + PIP2 <-> PKP_PIP2	c95f	c95f*PKP*PIP2 - c95r*PKP_PIP2	0.01	$nM^{-1}\cdot s^{-1}$	31, 32 (*S1.1*) + [1-9]
**140**	PKP + PIP2 <-> PKP_PIP2	c95r		0.035	s^{-1}	31, 32 (*S1.1*) + [1-9]
**141**	PKP_PIP2 -> PKP + PIP3	c96	c96*PKP_PIP2	2.5	s^{-1}	31, 32 (*S1.1*) + [1-9]
**142**	RP_ShP_G_GABP + PhoA <-> RP_ShP_G_GABP_PhoA	c98f	c98f*RP_ShP_G_GABP*PhoA - c98r*RP_ShP_G_GABP_PhoA	0.001	$nM^{-1}\cdot s^{-1}$	33 (*S1.1*) + [1-9]
**143**	RP_ShP_G_GABP + PhoA <-> RP_ShP_G_GABP_PhoA	c98r		0.1	s^{-1}	33 (*S1.1*) + [1-9]
**144**	RP_ShP_G_GABP_PhoA -> RP_ShP_G_GAB + PhoA	c100	c100*RP_ShP_G_GABP_PhoA	0.03	s^{-1}	33 (*S1.1*) + [1-9]
**145**	RP_G_GABP + PhoA <-> RP_G_GABP_PhoA	c99f	c99f*RP_G_GABP*PhoA - c99r*RP_G_GABP_PhoA	0.001	$nM^{-1}\cdot s^{-1}$	33 (*S1.1*) + [1-9]
**146**	RP_G_GABP + PhoA <-> RP_G_GABP_PhoA	c99r		0.1	s^{-1}	33 (*S1.1*) + [1-9]
**147**	RP_G_GABP_PhoA -> RP_G_GAB + PhoA	c101	c101*RP_G_GABP_PhoA	0.03	s^{-1}	33 (*S1.1*) + [1-9]

**148**	RP_G_GABP_PKP + PIP2 <-> RP_G_GABP_PKP_PIP2	c102f	c102f*RP_G_GABP_PKP*PIP2 - c102r*RP_G_GABP_PKP_PIP2	6.25	$nM^{-1}\cdot s^{-1}$	33 (*S1.1*) + [1-9]
**149**	RP_G_GABP_PKP + PIP2 <-> RP_G_GABP_PKP_PIP2	c102r		3.5	s^{-1}	33 (*S1.1*) + [1-9]
**150**	RP_ShP_G_GABP_PKP + PIP2 <-> RP_ShP_G_GABP_PKP_PIP2	c103f	c103f*RP_ShP_G_GABP_PKP*PIP2 - c103r*RP_ShP_G_GABP_PKP_PIP2	6.25	$nM^{-1}\cdot s^{-1}$	33 (*S1.1*) + [1-9]
**151**	RP_ShP_G_GABP_PKP + PIP2 <-> RP_ShP_G_GABP_PKP_PIP2	c103r		3.5	s^{-1}	33 (*S1.1*) + [1-9]
**152**	RP_G_GABP_PKP_PIP2 -> RP_G_GABP_PKP + PIP3	c104	c104*RP_G_GABP_PKP_PIP2	25	s^{-1}	33 (*S1.1*) + [1-9]
**153**	RP_ShP_G_GABP_PKP_PIP2 -> RP_ShP_G_GABP_PKP + PIP3	c105	c105*RP_ShP_G_GABP_PKP_PIP2	25	s^{-1}	33 (*S1.1*) + [1-9]
**154**	RP_ShP_G + GABP <-> RP_ShP_G_GABP	c110r	c110f*RP_ShP_G*GABP - c110r*RP_ShP_G_GABP	1	s^{-1}	33 (*S1.1*) + [1-9]
**155**	RP_ShP_G + GABP <-> RP_ShP_G_GABP	c110f		0.01	$nM^{-1}\cdot s^{-1}$	33 (*S1.1*) + [1-9]
**156**	RP_G + GABP <-> RP_G_GABP	c111r	c111f*RP_G*GABP - c111r*RP_G_GABP	1	s^{-1}	33 (*S1.1*) + [1-9]
**157**	RP_G + GABP <-> RP_G_GABP	c111f		0.01	$nM^{-1}\cdot s^{-1}$	33 (*S1.1*) + [1-9]
**158**	GABP + PhoA <-> GABP_PhoA	c112f	c112f*GABP*PhoA - c112r*GABP_PhoA	0.001	$nM^{-1}\cdot s^{-1}$	33 (*S1.1*) + [1-9]
**159**	GABP + PhoA <-> GABP_PhoA	c112r		0.1	s^{-1}	33 (*S1.1*) + [1-9]
**160**	GABP_PhoA -> GAB + PhoA	c113	c113*GABP_PhoA	0.03	s^{-1}	33 (*S1.1*) + [1-9]
**161**	AKT + PIP3 <-> AKT_PIP3	c114f	c114f*AKT*PIP3 - c114r*AKT_PIP3	0.01	$nM^{-1}\cdot s^{-1}$	31, 32, 37 (*S1.1*) + [1-9]
**162**	AKT + PIP3 <-> AKT_PIP3	c114r		10	s^{-1}	31, 32, 37 (*S1.1*) + [1-9]
**163**	GAB + PIP3 <-> GAB_PIP3	c115f	c115f*GAB*PIP3 - c115r*GAB_PIP3	0.01	$nM^{-1}\cdot s^{-1}$	33 (*S1.1*) + [1-9]
**164**	GAB + PIP3 <-> GAB_PIP3	c115r		3	s^{-1}	33 (*S1.1*) + [1-9]
**165**	GABP + PIP3 <-> GABP_PIP3	c116f	c116f*GABP*PIP3 - c116r*GABP_PIP3	0.01	$nM^{-1}\cdot s^{-1}$	33 (*S1.1*) + [1-9]
**166**	GABP + PIP3 <-> GABP_PIP3	c116r		3	s^{-1}	33 (*S1.1*) + [1-9]
**167**	RP_G + GAB_PIP3 <-> RP_G_GAB_PIP3	c117f	c117f*RP_G*GAB_PIP3 - c117r*RP_G_GAB_PIP3	2.5	$nM^{-1}\cdot s^{-1}$	33 (*S1.1*) + [1-9]
**168**	RP_G + GAB_PIP3 <-> RP_G_GAB_PIP3	c117r		1	s^{-1}	33 (*S1.1*) + [1-9]
**169**	RP_ShP_G + GAB_PIP3 <-> RP_ShP_G_GAB_PIP3	c119r	c119f*RP_ShP_G*GAB_PIP3 - c119r*RP_ShP_G_GAB_PIP3	1	s^{-1}	33 (*S1.1*) + [1-9]
**170**	RP_ShP_G + GAB_PIP3 <-> RP_ShP_G_GAB_PIP3	c119f		2.5	$nM^{-1}\cdot s^{-1}$	33 (*S1.1*) + [1-9]
**171**	RP_ShP_G_GAB_PIP3 -> RP_ShP_G_GABP + PIP3	c120	c120*RP_ShP_G_GAB_PIP3	0.01	s^{-1}	33 (*S1.1*) + [1-9]
**172**	AKT_PIP3 + PDK1 <-> AKT_PIP3_PDK1	c124f	c124f*AKT_PIP3*PDK1 - c124r*AKT_PIP3_PDK1	0.01	$nM^{-1}\cdot s^{-1}$	31, 32, 37 (*S1.1*) + [1-9]
**173**	AKT_PIP3 + PDK1 <-> AKT_PIP3_PDK1	c124r		0.1	s^{-1}	31, 32, 37 (*S1.1*) + [1-9]
**174**	AKT_PIP3_PDK1 -> AKTP + PIP3 + PDK1	c123	c123*AKT_PIP3_PDK1	5	s^{-1}	31, 32, 37 (*S1.1*) + [1-9]

**175**	AKTP + TAKT <-> AKTP_TAKT	c129f	c129f*AKTP*TAKT - c129r*AKTP_TAKT	0.01	$nM^{-1}\cdot s^{-1}$	31, 32, 37 (*S1.1*) + [1-9]
**176**	AKTP + TAKT <-> AKTP_TAKT	c129r		1	s^{-1}	31, 32, 37 (*S1.1*) + [1-9]
**177**	AKTP_TAKT -> AKT + TAKT	c130	c130*AKTP_TAKT	1	s^{-1}	31, 32, 37 (*S1.1*) + [1-9]
**178**	PKP + PhoB <-> PKP_PhoB	c135f	c135f*PKP*PhoB - c135r*PKP_PhoB	0.01	$nM^{-1}\cdot s^{-1}$	31, 32, 37 (*S1.1*) + [1-9]
**179**	PKP + PhoB <-> PKP_PhoB	c135r		1	s^{-1}	31, 32, 37 (*S1.1*) + [1-9]
**180**	PKP_PhoB -> PI3K + PhoB	c136	c136*PKP_PhoB	0.5	s^{-1}	31, 32, 37 (*S1.1*) + [1-9]
**181**	RP_G_GABP_PKP + PhoB <-> RP_G_GABP_PKP_PhoB	c137f	c137f*RP_G_GABP_PKP*PhoB - c137r*RP_G_GABP_PKP_PhoB	0.001	$nM^{-1}\cdot s^{-1}$	33 (*S1.1*) + [1-9]
**182**	RP_G_GABP_PKP + PhoB <-> RP_G_GABP_PKP_PhoB	c137r		0.038	s^{-1}	33 (*S1.1*) + [1-9]
**183**	RP_G_GABP_PKP_PhoB -> RP_G_GABP_PK + PhoB	c138	c138*RP_G_GABP_PKP_PhoB	0.595	s^{-1}	33 (*S1.1*) + [1-9]
**184**	RP_ShP_G_GABP_PKP + PhoB <-> RP_ShP_G_GABP_PKP_PhoB	c139f	c139f*RP_ShP_G_GABP_PKP*PhoB - c139r*RP_ShP_G_GABP_PKP_PhoB	0.001	$nM^{-1}\cdot s^{-1}$	33 (*S1.1*) + [1-9]
**185**	RP_ShP_G_GABP_PKP + PhoB <-> RP_ShP_G_GABP_PKP_PhoB	c139r		0.038	s^{-1}	33 (*S1.1*) + [1-9]
**186**	RP_ShP_G_GABP_PKP_PhoB -> RP_ShP_G_GABP_PK + PhoB	c140	c140*RP_ShP_G_GABP_PKP_PhoB	0.595	s^{-1}	33 (*S1.1*) + [1-9]
**187**	RP_ShP_G_GAB + PIP3 <-> RP_ShP_G_GAB_PIP3	c143f	c143f*RP_ShP_G_GAB*PIP3 - c143r*RP_ShP_G_GAB_PIP3	2.5	$nM^{-1}\cdot s^{-1}$	33 (*S1.1*) + [1-9]
**188**	RP_ShP_G_GAB + PIP3 <-> RP_ShP_G_GAB_PIP3	c143r		1	s^{-1}	33 (*S1.1*) + [1-9]
**189**	RP_PKP + PIP2 <-> RP_PKP_PIP2	c146f	c146f*RP_PKP*PIP2 - c146r*RP_PKP_PIP2	6.25	$nM^{-1}\cdot s^{-1}$	31, 32 (*S1.1*) + [1-9]
**190**	RP_PKP + PIP2 <-> RP_PKP_PIP2	c146r		3.5	s^{-1}	31, 32 (*S1.1*) + [1-9]
**191**	RP_PKP_PIP2 -> RP_PKP + PIP3	c147f	c147f*RP_PKP_PIP2	2.5	s^{-1}	31, 32 (*S1.1*) + [1-9]
**192**	RP_ShP_G_GABP_PKP <-> RP_ShP_G_GABP + PKP	c148f	c148f*RP_ShP_G_GABP_PKP - c148r*RP_ShP_G_GABP*PKP	5	s^{-1}	33 (*S1.1*) + [1-9]
**193**	RP_ShP_G_GABP_PKP <-> RP_ShP_G_GABP + PKP	c148r		0.05	$nM^{-1}\cdot s^{-1}$	33 (*S1.1*) + [1-9]
**194**	RP_G_GABP_PKP <-> RP_G_GABP + PKP	c149f	c149f*RP_G_GABP_PKP - c149r*RP_G_GABP*PKP	5	s^{-1}	33 (*S1.1*) + [1-9]
**195**	RP_G_GABP_PKP <-> RP_G_GABP + PKP	c149r		0.05	$nM^{-1}\cdot s^{-1}$	33 (*S1.1*) + [1-9]
**196**	RP_G_GAB_PIP3 -> RP_G_GABP + PIP3	c150	c150*RP_G_GAB_PIP3	0.01	s^{-1}	33 (*S1.1*) + [1-9]
**197**	GSKP + PhoC <-> GSKP_PhoC	c151f	c151f*GSKP*PhoC - c151r*GSKP_PhoC	0.01	$nM^{-1}\cdot s^{-1}$	46 - 50 (*S1.1*) + [12-13]
**198**	GSKP + PhoC <-> GSKP_PhoC	c151r		5	s^{-1}	46 - 50 (*S1.1*) + [12-13]
**199**	GSKP_PhoC -> GSK + PhoC	c152	c152*GSKP_PhoC	0.1	s^{-1}	46 - 50 (*S1.1*) + [12-13]
**200**	RP_G_GAB + PIP3 <-> RP_G_GAB_PIP3	c156f	c156f*RP_G_GAB*PIP3 - c156r*RP_G_GAB_PIP3	1	$nM^{-1}\cdot s^{-1}$	31, 33 (*S1.1*) + [1-9]

**201**	RP_G_GAB + PIP3 <-> RP_G_GAB_PIP3	c156r		2.5	s^{-1}	31, 33 (*S1.1*) + [1-9]
**202**	[Raf) + AKTP <-> [Raf_ AKTP)	c157f	c157f*[Raf*)*AKTP - c157r*[Raf*_ AKTP)	0.01	$nM^{-1}\cdot s^{-1}$	38 (*S1.1*) + [11,13]
**203**	[Raf) + AKTP <-> [Raf_ AKTP)	c157r		0.033	s^{-1}	38 (*S1.1*) + [11,13]
**204**	[Raf_ AKTP) -> [Raf) + AKTP	c158	c158*[Raf*_ AKTP)	5.7	s^{-1}	38 (*S1.1*) + [11,13]
**205**	[Raf) + Pase1 <-> [Raf_Pase1)	c159f	c159f*[Raf**)*Pase1 - c159r*[Raf**_Pase1)	0.0717	$nM^{-1}\cdot s^{-1}$	12 – 14 (1*S1.1*) + [11,13]
**206**	[Raf) + Pase1 <-> [Raf_Pase1)	c159r		0.2	s^{-1}	12 – 14 (1*S1.1*) + [11,13]
**207**	[Raf_Pase1) -> [Raf) + Pase1	c160	c160*[Raf**_Pase1)	1	s^{-1}	12 – 14 (1*S1.1*) + [11,13]
**208**	AKTP + GSK <-> [AKTP_ GSK)	c170f	c170f*AKTP*GSK - c170r*[AKTP_ GSK)	0.01	$nM^{-1}\cdot s^{-1}$	31 - 32, 44 - 49 (1*S1.1*) + [12-13]
**209**	AKTP + GSK <-> [AKTP_ GSK)	c170r		0.1	s^{-1}	31 - 32, 44 - 49 (1*S1.1*) + [12-13]
**210**	[AKTP_ GSK) -> AKTP + GSKP	c171	c171*[AKTP_ GSK)	1	s^{-1}	31 - 32, 44 - 49 (1*S1.1*) + [12-13]
**211**	R + R <-> R_R	c181f		0.0001	$nM^{-1}\cdot s^{-1}$	1 - 3 (*S1.1*) + [1-9,12-16]
**212**	R + R <-> R_R	c181r	c181f*R*R - c181r*R_R	0.1	s^{-1}	1 - 3 (*S1.1*) + [1-9,12-16]
**213**	EGF + R_R <-> Rl_R	c182f	c182f*EGF*R_R - c182r*Rl_R	0.0001	$nM^{-1}\cdot s^{-1}$	1 - 3 (*S1.1*) + [1-9,12-16]
**214**	EGF + R_R <-> Rl_R	c182r		1	s^{-1}	1 - 3 (*S1.1*) + [1-9,12-16]
**215**	Rl + R <-> Rl_R	c183f	c183f*Rl*R - c183r*Rl_R	0.001	$nM^{-1}\cdot s^{-1}$	1 - 3 (*S1.1*) + [1-9,12-16]
**216**	Rl + R <-> Rl_R	c183r		0.1	s^{-1}	1 - 3 (*S1.1*) + [1-9,12-16]
**217**	Rl_R -> RlP_RP	c184f	c184f*Rl_R	1	s^{-1}	1 - 3 (*S1.1*) + [1-9,12-16]
**218**	RlP_RP <-> EGF + RP_RP	c185f	c185f*RlP_RP - c185r*EGF*RP_RP	0.1	s^{-1}	1 - 3 (*S1.1*) + [1-9,12-16]
**219**	RlP_RP <-> EGF + RP_RP	c185r		0.01	$nM^{-1}\cdot s^{-1}$	1 - 3 (*S1.1*) + [1-9,12-16]
**220**	RP_RP <-> RP + RP	c186f	c186f*RP_RP - c187r*RP*RP	0.1	s^{-1}	1 - 3 (*S1.1*) + [1-9,12-16]
**221**	RP_RP <-> RP + RP	c187r	c187f*RP_R	0.01	$nM^{-1}\cdot s^{-1}$	1 - 3 (*S1.1*) + [1-9,12-16]
**222**	RP_R -> RP_RP	c187f		1	s^{-1}	1 - 3 (*S1.1*) + [1-9,12-16]
**223**	RP + R <-> RP_R	c189f	c189f*RP*R - c189r*RP_R	0.001	$nM^{-1}\cdot s^{-1}$	1 - 3 (*S1.1*) + [1-9,12-16]
**224**	RP + R <-> RP_R	c189r		0.1	s^{-1}	1 - 3 (*S1.1*) + [1-9,12-16]
**225**	CDC25CP + RP <-> CDC25CP_RP	c190f	c190f*CDC25CP*RP - c190r*CDC25CP_RP	0.01	$nM^{-1}\cdot s^{-1}$	26, 27 (*S1.1*) + [12-13]
**226**	CDC25CP + RP <-> CDC25CP_RP	c190r		0.1	s^{-1}	26, 27 (*S1.1*) + [12-13]
**227**	CDC25CP_RP -> CDC25CP + R	c191f	c191f*CDC25CP_RP	1	s^{-1}	26, 27 (*S1.1*) + [12-13]
**228**	CDC25CP + RP_RP <-> CDC25CP_RP_RP	c192f		0.01	$nM^{-1}\cdot s^{-1}$	26, 27 (*S1.1*) + [12-13]
**229**	CDC25CP + RP_RP <-> CDC25CP_RP_RP	c192r	c192f*CDC25CP*RP_RP - c192r*CDC25CP_RP_RP	0.1	s^{-1}	26, 27 (*S1.1*) + [12-13]

**230**	CDC25CP_RP_RP -> CDC25CP + R_R	c193f	c193f*CDC25CP_RP_RP	0.5	s^{-1}	26, 27 (*S1.1*) + [12-13]
**231**	R + ERB <-> R_ERB	c194f	c194f*R*ERB - c194r*R_ERB	0.001	$nM^{-1}\cdot s^{-1}$	1 - 3 (*S1.1*) + [1,6,13-16]
**232**	R + ERB <-> R_ERB	c194r		0.1	s^{-1}	1 - 3 (*S1.1*) + [1,6,13-16]
**233**	R_ERB + EGF <-> Rl_ERB	c195f	c195f*R_ERB*EGF - c195r*Rl_ERB	0.01	$nM^{-1}\cdot s^{-1}$	1 - 3 (*S1.1*) + [1,6,13-16]
**234**	R_ERB + EGF <-> Rl_ERB	c195r		0.1	s^{-1}	1 - 3 (*S1.1*) + [1,6,13-16]
**235**	Rl_ERB -> RlP_ERBP	c196f	c196f*Rl_ERB	1	s^{-1}	1 - 3 (*S1.1*) + [1,6,13-16]
**236**	RlP_ERBP <-> EGF + RP_ERBP	c197f	c197f*RlP_ERBP - c197r*EGF*RP_ERBP	0.1	s^{-1}	1 - 3 (*S1.1*) + [1,6,13-16]
**237**	RlP_ERBP <-> EGF + RP_ERBP	c197r		0.01	$nM^{-1}\cdot s^{-1}$	1 - 3 (*S1.1*) + [1,6,13-16]
**238**	RP_ERBP <-> RP + ERBP	c198f	c198f*RP_ERBP - c198r*RP*ERBP	0.1	s^{-1}	1 - 3 (*S1.1*) + [1,6,13-16]
**239**	RP_ERBP <-> RP + ERBP	c198r		0.01	$nM^{-1}\cdot s^{-1}$	1 - 3 (*S1.1*) + [1,6,13-16]
**240**	Rl + ERB <-> Rl_ERB	c199f	c199f*Rl*ERB - c199r*Rl_ERB	0.01	$nM^{-1}\cdot s^{-1}$	1 - 3 (*S1.1*) + [1,6,13-16]
**241**	Rl + ERB <-> Rl_ERB	c199r		0.1	s^{-1}	1 - 3 (*S1.1*) + [1,6,13-16]
**242**	R + ERBP <-> R_ERBP	c200f	c200f*R*ERBP - c200r*R_ERBP	0.001	$nM^{-1}\cdot s^{-1}$	1 - 3 (*S1.1*) + [1,6,13-16]
**243**	R + ERBP <-> R_ERBP	c200r		0.1	s^{-1}	1 - 3 (*S1.1*) + [1,6,13-16]
**244**	R_ERBP -> RP_ERBP	c201f	c201f*R_ERBP	1	s^{-1}	1 - 3 (*S1.1*) + [1,6,13-16]
**245**	RlP_ERBP <-> RlP + ERBP	c202f	c202f*RlP_ERBP - c202r*RlP*ERBP	0.1	s^{-1}	1 - 3 (*S1.1*) + [1,6,13-16]
**246**	RlP_ERBP <-> RlP + ERBP	c202r		0.01	$nM^{-1}\cdot s^{-1}$	1 - 3 (*S1.1*) + [1,6,13-16]
**247**	RP + ERB <-> RP_ERB	c203f	c203f*RP*ERB - c203r*RP_ERB	0.01	$nM^{-1}\cdot s^{-1}$	1 - 3 (*S1.1*) + [1,6,13-16]
**248**	RP + ERB <-> RP_ERB	c203r		0.1	s^{-1}	1 - 3 (*S1.1*) + [1,6,13-16]
**249**	RP_ERB -> RP_ERBP	c204f	c204f*RP_ERB	1	s^{-1}	1 - 3 (*S1.1*) + [1,6,13-16]
**250**	ERBP + ERB <-> ERBP_ERB	c205f	c205f*ERBP*ERB - c205r*ERBP_ERB	0.01	$nM^{-1}\cdot s^{-1}$	1 - 3 (*S1.1*) + [1,6,13-16]
**251**	ERBP + ERB <-> ERBP_ERB	c205r		0.1	s^{-1}	1 - 3 (*S1.1*) + [1,6,13-16]
**252**	ERBP_ERBP <-> ERBP + ERBP	c207f	c207f*ERBP_ERBP - c207r*ERBP*ERBP	0.1	s^{-1}	1 - 3 (*S1.1*) + [1,6,13-16]
**253**	ERBP_ERBP <-> ERBP + ERBP	c207r		0.01	$nM^{-1}\cdot s^{-1}$	1 - 3 (*S1.1*) + [1,6,13-16]
**254**	ERB + ERB <-> ERB_ERB	c208f	c208f*ERB*ERB - c209r*ERB_ERB	0.01	$nM^{-1}\cdot s^{-1}$	1 - 3 (*S1.1*) + [1,6,13-16]
**255**	ERB + ERB <-> ERB_ERB	c209r		0.1	s^{-1}	1 - 3 (*S1.1*) + [1,6,13-16]
**256**	ERBP_ERB -> ERBP_ERBP	c206f	c206f*ERBP_ERB	1	s^{-1}	1 - 3 (*S1.1*) + [1,6,13-16]
**257**	CDC25CP + ERBP <-> CDC25CP_ERBP	c207f_n	c207f_n*CDC25CP*ERBP - c208r*CDC25CP_ERBP	0.01	$nM^{-1}\cdot s^{-1}$	26, 27 (*S1.1*) + [12-13,16]
**258**	CDC25CP + ERBP <-> CDC25CP_ERBP	c208r	c208f_n*CDC25CP_ERBP	0.1	s^{-1}	26, 27 (*S1.1*) + [12-13,16]
**259**	CDC25CP_ERBP -> CDC25CP + ERB	c208f_n		0.5	s^{-1}	26, 27 (*S1.1*) + [12-13,16]
**260**	ShP + PTP1E <-> PTP1E_ShP	c210f	c210f*ShP*PTP1E - c210r*PTP1E_ShP	0.01	$nM^{-1}\cdot s^{-1}$	10 (*S1.1*) + [1]

**261**	ShP + PTP1E <-> PTP1E_ShP	c210r		0.1	s^{-1}	10 (*S1.1*) + [1]
**262**	PTP1E_ShP -> PTP1E + Shc	c209f	c209f*PTP1E_ShP	0.005	s^{-1}	10 (*S1.1*) + [1]
**263**	PLCyP + PaseX <-> PLCyP_PaseX	c211f	c211f*PLCyP*PaseX - c211r*PLCyP_PaseX	0.01	$nM^{-1}\cdot s^{-1}$	1 - 3 (*S1.1*) + [1]
**264**	PLCyP + PaseX <-> PLCyP_PaseX	c211r		0.1	s^{-1}	1 - 3 (*S1.1*) + [1]
**265**	PLCyP_PaseX -> PLCy + PaseX	c212f	c212f*PLCyP_PaseX	0.01	s^{-1}	1 - 3 (*S1.1*) + [1]
**266**	Rl2P <-> RlP + RlP	c213f	c213f*Rl2P - c213r*RlP*RlP	0.1	s^{-1}	1 - 3 (*S1.1*) + [1,6,13-16]
**267**	Rl2P <-> RlP + RlP	c213r		0.01	$nM^{-1}\cdot s^{-1}$	1 - 3 (*S1.1*) + [1,6,13-16]
**268**	RlP <-> EGF + RP	c214f	c214f*RlP - c214r*EGF*RP	0.1	s^{-1}	1 - 3 (*S1.1*) + [1,6,13-16]
**269**	RlP <-> EGF + RP	c214r		0.01	$nM^{-1}\cdot s^{-1}$	1 - 3 (*S1.1*) + [1,6,13-16]
**270**	CDC25CP + ERBP_ERBP <-> CDC25CP_ERBP_ERBP	c215f	c215f*CDC25CP*ERBP_ERBP - c215r*CDC25CP_ERBP_ERBP	0.01	$nM^{-1}\cdot s^{-1}$	26, 27 (*S1.1*) + [12-13,16]
**271**	CDC25CP + ERBP_ERBP <-> CDC25CP_ERBP_ERBP	c215r		0.1	s^{-1}	26, 27 (*S1.1*) + [12-13,16]
**272**	CDC25CP_ERBP_ERBP -> CDC25CP + ERB_ERB	c216f	c216f*CDC25CP_ERBP_ERBP	0.5	s^{-1}	26, 27 (*S1.1*) + [12-13,16]
**273**	ERBP + PLCy <-> ERBP_PL	c_a_185f	c_a_185f*ERBP*PLCy - c_a_185r*ERBP_PL	0.06	$nM^{-1}\cdot s^{-1}$	2, 3 (*S1.1*) + [1-9,16]
**274**	ERBP + PLCy <-> ERBP_PL	c_a_185r		0.2	s^{-1}	2, 3 (*S1.1*) + [1-9,16]
**275**	ERBP_PL -> ERBP_PLP	c_a_186f	c_a_186f*ERBP_PL	1	s^{-1}	2, 3 (*S1.1*) + [1-9,16]
**276**	ERBP_PLP <-> ERBP + PLCyP	c_a_187f	c_a_187f*ERBP_PLP - c_a_187r*ERBP*PLCyP	0.3	s^{-1}	2, 3 (*S1.1*) + [1-9,16]
**277**	ERBP_PLP <-> ERBP + PLCyP	c_a_187r		0.006	$nM^{-1}\cdot s^{-1}$	2, 3 (*S1.1*) + [1-9,16]
**278**	ERBP + Grb <-> ERBP_G	c_a_190f	c_a_190f*ERBP*Grb - c_a_190r*ERBP_G	0.0015	$nM^{-1}\cdot s^{-1}$	2, 3 (*S1.1*) + [1-9,16]
**279**	ERBP + Grb <-> ERBP_G	c_a_190r		0.2	s^{-1}	2, 3 (*S1.1*) + [1-9,16]
**280**	ERBP_G + SOS <-> ERBP_G_S	c_a_191f	c_a_191f*ERBP_G*SOS - c_a_191r*ERBP_G_S	0.01	$nM^{-1}\cdot s^{-1}$	2, 3 (*S1.1*) + [1-9,16]
**281**	ERBP_G + SOS <-> ERBP_G_S	c_a_191r		0.06	s^{-1}	2, 3 (*S1.1*) + [1-9,16]
**282**	ERBP_G_S <-> ERBP + G_S	c_a_192f	c_a_192f*ERBP_G_S - c_a_192r*ERBP*G_S	0.15	s^{-1}	2, 3 (*S1.1*) + [1-9,16]
**283**	ERBP_G_S <-> ERBP + G_S	c_a_192r		0.0028	$nM^{-1}\cdot s^{-1}$	2, 3 (*S1.1*) + [1-9,16]
**284**	ERBP + Shc <-> ERBP_Sh	c_a_194f	c_a_194f*ERBP*Shc - c_a_194r*ERBP_Sh	0.09	$nM^{-1}\cdot s^{-1}$	2, 3 (*S1.1*) + [1-9,16]
**285**	ERBP + Shc <-> ERBP_Sh	c_a_194r		0.6	s^{-1}	2, 3 (*S1.1*) + [1-9,16]
**286**	ERBP_Sh -> ERBP_ShP	c_a_195	c_a_195*ERBP_Sh	6	s^{-1}	2, 3 (*S1.1*) + [1-9,16]
**287**	ERBP_ShP <-> ShP + ERBP	c_a_196f	c_a_196f*ERBP_ShP - c_a_196r*ShP*ERBP	0.3	s^{-1}	2, 3 (*S1.1*) + [1-9,16]
**288**	ERBP_ShP <-> ShP + ERBP	c_a_196r		0.0009	$nM^{-1}\cdot s^{-1}$	2, 3 (*S1.1*) + [1-9,16]
**289**	ERBP_ShP + Grb <-> ERBP_ShP_G	c_a_197f	c_a_197f*ERBP_ShP*Grb - c_a_197r*ERBP_ShP_G	0.003	$nM^{-1}\cdot s^{-1}$	2, 3 (*S1.1*) + [1-9,16]
**290**	ERBP_ShP + Grb <-> ERBP_ShP_G	c_a_197r		0.1	s^{-1}	2, 3 (*S1.1*) + [1-9,16]

**291**	ERBP_ShP_G <-> ERBP + ShP_G	c_a_198f	c_a_198f*ERBP_ShP_G - c_a_198r*ERBP*ShP_G	0.3	s^{-1}	2, 3 (*S1.1*) + [1-9,16]
**292**	ERBP_ShP_G <-> ERBP + ShP_G	c_a_198r		0.0009	$nM^{-1}\cdot s^{-1}$	2, 3 (*S1.1*) + [1-9,16]
**293**	ERBP_ShP_G + SOS <-> ERBP_ShP_G_S	c_a_199f	c_a_199f*ERBP_ShP_G*SOS - c_a_199r*ERBP_ShP_G_S	0.01	$nM^{-1}\cdot s^{-1}$	2, 3 (*S1.1*) + [1-9,16]
**294**	ERBP_ShP_G + SOS <-> ERBP_ShP_G_S	c_a_199r		0.0214	s^{-1}	2, 3 (*S1.1*) + [1-9,16]
**295**	ERBP_ShP_G_S <-> ShP_G_S + ERBP	c_a_200f	c_a_200f*ERBP_ShP_G_S - c_a_200r*ShP_G_S*ERBP	0.12	s^{-1}	2, 3 (*S1.1*) + [1-9,16]
**296**	ERBP_ShP_G_S <-> ShP_G_S + ERBP	c_a_200r		0.00024	$nM^{-1}\cdot s^{-1}$	2, 3 (*S1.1*) + [1-9,16]
**297**	ERBP_ShP + G_S <-> ERBP_ShP_G_S	c_a_205f	c_a_205f*ERBP_ShP*G_S - c_a_205r*ERBP_ShP_G_S	0.009	$nM^{-1}\cdot s^{-1}$	2, 3 (*S1.1*) + [1-9,16]
**298**	ERBP_ShP + G_S <-> ERBP_ShP_G_S	c_a_205r		0.0429	s^{-1}	2, 3 (*S1.1*) + [1-9,16]
**299**	ERBP_ShP_G_S + Ras_GDP <-> ERBP_ShP_G_S_Ras_GDP	c_a_209f	c_a_209f*ERBP_ShP_G_S*Ras_GDP - c_a_209r*ERBP_ShP_G_S_Ras_GDP	0.00475	$nM^{-1}\cdot s^{-1}$	2, 3 (*S1.1*) + [1-9,16]
**300**	ERBP_ShP_G_S + Ras_GDP <-> ERBP_ShP_G_S_Ras_GDP	c_a_209r		0.76	s^{-1}	2, 3 (*S1.1*) + [1-9,16]
**301**	ERBP_ShP_G_S_Ras_GDP <-> ERBP_ShP_G_S_Ras + GDP	c_a_210f	c_a_210f*ERBP_ShP_G_S_Ras_GDP - c_a_210r*ERBP_ShP_G_S_Ras*GDP	46.5	s^{-1}	2, 3 (*S1.1*) + [1-9,16]
**302**	ERBP_ShP_G_S_Ras_GDP <-> ERBP_ShP_G_S_Ras + GDP	c_a_210r		0.093	$nM^{-1}\cdot s^{-1}$	2, 3 (*S1.1*) + [1-9,16]
**303**	ERBP_ShP_G_S_Ras + GTP <-> ERBP_ShP_G_S_Ras_GTP	c_a_211f	c_a_211f*ERBP_ShP_G_S_Ras*GTP - c_a_211r*ERBP_ShP_G_S_Ras_GTP	0.003	$nM^{-1}\cdot s^{-1}$	2, 3 (*S1.1*) + [1-9,16]
**304**	ERBP_ShP_G_S_Ras + GTP <-> ERBP_ShP_G_S_Ras_GTP	c_a_211r		2.4	s^{-1}	2, 3 (*S1.1*) + [1-9,16]
**305**	ERBP_ShP_G_S_Ras_GTP <-> ERBP_ShP_G_S + Ras_GTP	c_a_212f	c_a_212f*ERBP_ShP_G_S_Ras_GTP - c_a_212r*ERBP_ShP_G_S*Ras_GTP	806.4	s^{-1}	2, 3 (*S1.1*) + [1-9,16]
**306**	ERBP_ShP_G_S_Ras_GTP <-> ERBP_ShP_G_S + Ras_GTP	c_a_212r		1.575	$nM^{-1}\cdot s^{-1}$	2, 3 (*S1.1*) + [1-9,16]
**307**	ERBP_ShP_G_S + Ras <-> ERBP_ShP_G_S_Ras	c_a_213f	c_a_213f*ERBP_ShP_G_S*Ras - c_a_213r*ERBP_ShP_G_S_Ras	0.15625	$nM^{-1}\cdot s^{-1}$	2, 3 (*S1.1*) + [1-9,16]
**308**	ERBP_ShP_G_S + Ras <-> ERBP_ShP_G_S_Ras	c_a_213r		0.001	s^{-1}	2, 3 (*S1.1*) + [1-9,16]
**309**	ERBP_G_S + Ras_GDP <-> ERBP_G_S_Ras_GDP	c_a_214f	c_a_214f*ERBP_G_S*Ras_GDP - c_a_214r*ERBP_G_S_Ras_GDP	0.0075	$nM^{-1}\cdot s^{-1}$	2, 3 (*S1.1*) + [1-9,16]
**310**	ERBP_G_S + Ras_GDP <-> ERBP_G_S_Ras_GDP	c_a_214r		1.2	s^{-1}	2, 3 (*S1.1*) + [1-9,16]
**311**	ERBP_G_S_Ras_GDP <-> ERBP_G_S_Ras + GDP	c_a_215f	c_a_215f*ERBP_G_S_Ras_GDP - c_a_215r*ERBP_G_S_Ras*GDP	50	s^{-1}	2, 3 (*S1.1*) + [1-9,16]
**312**	ERBP_G_S_Ras_GDP <-> ERBP_G_S_Ras + GDP	c_a_215r		0.1	$nM^{-1}\cdot s^{-1}$	2, 3 (*S1.1*) + [1-9,16]

**313**	ERBP_G_S_Ras + GTP <-> ERBP_G_S_Ras_GTP	c_a_216f	c_a_216f*ERBP_G_S_Ras*GTP - c_a_216r*ERBP_G_S_Ras_GTP	0.1	$nM^{-1}\cdot s^{-1}$	2, 3 (*S1.1*) + [1-9,16]
**314**	ERBP_G_S_Ras + GTP <-> ERBP_G_S_Ras_GTP	c_a_216r		80	s^{-1}	2, 3 (*S1.1*) + [1-9,16]
**315**	ERBP_G_S_Ras_GTP <-> ERBP_G_S + Ras_GTP	c_a_217f	c_a_217f*ERBP_G_S_Ras_GTP - c_a_217r*ERBP_G_S*Ras_GTP	640	s^{-1}	2, 3 (*S1.1*) + [1-9,16]
**316**	ERBP_G_S_Ras_GTP <-> ERBP_G_S + Ras_GTP	c_a_217r		1.25	$nM^{-1}\cdot s^{-1}$	2, 3 (*S1.1*) + [1-9,16]
**317**	ERBP_G_S + Ras <-> ERBP_G_S_Ras	c_a_218f	c_a_218f*ERBP_G_S*Ras - c_a_218r*ERBP_G_S_Ras	0.25	$nM^{-1}\cdot s^{-1}$	2, 3 (*S1.1*) + [1-9,16]
**318**	ERBP_G_S + Ras <-> ERBP_G_S_Ras	c_a_218r		0.0016	s^{-1}	2, 3 (*S1.1*) + [1-10,16]
**319**	GAP + ERBP <-> ERBP_GAP	c_a_239f	c_a_239f*GAP*ERBP - c_a_239r*ERBP_GAP	0.083	$nM^{-1}\cdot s^{-1}$	2, 3 (*S1.1*) + [1-10,16]
**320**	GAP + ERBP <-> ERBP_GAP	c_a_239r		0.15	s^{-1}	2, 3 (*S1.1*) + [1-10,16]
**321**	Ras_GTP + ERBP_GAP <-> ERBP_GAP_Ras_GTP	c_a_240f	c_a_240f*Ras_GTP*ERBP_GAP - c_a_240r*ERBP_GAP_Ras_GTP	0.01	$nM^{-1}\cdot s^{-1}$	2, 3 (*S1.1*) + [1-10,16]
**322**	Ras_GTP + ERBP_GAP <-> ERBP_GAP_Ras_GTP	c_a_240r		0.03	s^{-1}	2, 3 (*S1.1*) + [1-10,16]
**323**	ERBP_GAP_Ras_GTP -> ERBP_GAP + Ras_GDP	c_a_241	c_a_241*ERBP_GAP_Ras_GTP	1.494	s^{-1}	2, 3 (*S1.1*) + [1-10,16]
**324**	ERBP_ShP_G_S + ERKPP <-> ERBP_ShP_G_S_ERKPP	c_a_250f	c_a_250f*ERBP_ShP_G_S*ERKPP - c_a_250r*ERBP_ShP_G_S_ERKPP	0.01	$nM^{-1}\cdot s^{-1}$	11, 24 - 25 (*S1.1*) + [1,16]
**325**	ERBP_ShP_G_S + ERKPP <-> ERBP_ShP_G_S_ERKPP	c_a_250r		0.033	s^{-1}	11, 24 - 25 (*S1.1*) + [1,16]
**326**	ERBP_ShP_G_S_ERKPP -> ERBP_ShP_G + ERKPP + SOSP	c_a_251	c_a_251*ERBP_ShP_G_S_ERKPP	1	s^{-1}	11, 24 - 25 (*S1.1*) + [1,16]
**327**	ERBP_G_S + ERKPP <-> ERBP_G_S_ERKPP	c_a_252f	c_a_252f*ERBP_G_S*ERKPP - c_a_252r*ERBP_G_S_ERKPP	0.01	$nM^{-1}\cdot s^{-1}$	11, 24 - 25 (*S1.1*) + [1,16]
**328**	ERBP_G_S + ERKPP <-> ERBP_G_S_ERKPP	c_a_252r		0.033	s^{-1}	11, 24 - 25 (*S1.1*) + [1,16]
**329**	ERBP_G_S_ERKPP -> ERBP_G + SOSP + ERKPP	c_a_253	c_a_253*ERBP_G_S_ERKPP	1	s^{-1}	11, 24 - 25 (*S1.1*) + [1,16]
**330**	ERBP_G + GAB <-> ERBP_G_GAB	c_a_256f	c_a_256f*ERBP_G*GAB - c_a_256r*ERBP_G_GAB	0.01	$nM^{-1}\cdot s^{-1}$	33 (*S1.1*) + [1,16]
**331**	ERBP_G + GAB <-> ERBP_G_GAB	c_a_256r		1	s^{-1}	33 (*S1.1*) + [1,16]
**332**	ERBP_ShP_G + GAB <-> ERBP_ShP_G_GAB	c_a_257f	c_a_257f*ERBP_ShP_G*GAB - c_a_257r*ERBP_ShP_G_GAB	0.01	$nM^{-1}\cdot s^{-1}$	33 (*S1.1*) + [1,16]
**333**	ERBP_ShP_G + GAB <-> ERBP_ShP_G_GAB	c_a_257r		1	s^{-1}	33 (*S1.1*) + [1,16]
**334**	ERBP_G_GAB -> ERBP_G_GABP	c_a_258	c_a_258*ERBP_G_GAB	0.05	s^{-1}	33 (*S1.1*) + [1,16]

**335**	ERBP_ShP_G_GAB -> ERBP_ShP_G_GABP	c_a_259	c_a_259*ERBP_ShP_G_GAB	0.05	s^{-1}	33 (*S1.1*) + [1,16]
**336**	ERBP_G_GABP + PI3K <-> ERBP_G_GABP_PK	c_a_260f	c_a_260f*ERBP_G_GABP*PI3K - c_a_260r*ERBP_G_GABP_PK	0.01	$nM^{-1}\cdot s^{-1}$	31 - 33 (*S1.1*) + [12-13,16]
**337**	ERBP_G_GABP + PI3K <-> ERBP_G_GABP_PK	c_a_260r		1	s^{-1}	31 - 33 (*S1.1*) + [12-13,16]
**338**	ERBP_G_GABP_PK -> ERBP_G_GABP_PKP	c_a_262	c_a_262*ERBP_G_GABP_PK	1	s^{-1}	31 - 33 (*S1.1*) + [12-13,16]
**339**	ERBP_ShP_G_GABP + PI3K <-> ERBP_ShP_G_GABP_PK	c_a_261f	c_a_261f*ERBP_ShP_G_GABP*PI3K - c_a_261r*ERBP_ShP_G_GABP_PK	0.01	$nM^{-1}\cdot s^{-1}$	31 - 33 (*S1.1*) + [12-13,16]
**340**	ERBP_ShP_G_GABP + PI3K <-> ERBP_ShP_G_GABP_PK	c_a_261r		1	s^{-1}	31 - 33 (*S1.1*) + [12-13,16]
**341**	ERBP_ShP_G_GABP_PK -> ERBP_ShP_G_GABP_PKP	c_a_263	c_a_263*ERBP_ShP_G_GABP_PK	1	s^{-1}	31 - 33 (*S1.1*) + [12-13,16]
**342**	ERBP_ShP_G_GABP + GAP <-> ERBP_ShP_G_GABP_GAP	c_a_264f	c_a_264f*ERBP_ShP_G_GABP*GAP - c_a_264r*ERBP_ShP_G_GABP_GAP	0.083	$nM^{-1}\cdot s^{-1}$	33 (*S1.1*) + [1,16]
**343**	ERBP_ShP_G_GABP + GAP <-> ERBP_ShP_G_GABP_GAP	c_a_264r		0.15	s^{-1}	33 (*S1.1*) + [1,16]
**344**	ERBP_G_GABP + GAP <-> ERBP_G_GABP_GAP	c_a_265f	c_a_265f*ERBP_G_GABP*GAP - c_a_265r*ERBP_G_GABP_GAP	0.083	$nM^{-1}\cdot s^{-1}$	33 (*S1.1*) + [1,16]
**345**	ERBP_G_GABP + GAP <-> ERBP_G_GABP_GAP	c_a_265r		0.15	s^{-1}	33 (*S1.1*) + [1,16]
**346**	ERBP_G_GABP_GAP + Ras_GTP <-> ERBP_G_GABP_GAP_Ras_GTP	c_a_266f	c_a_266f*ERBP_G_GABP_GAP*Ras_GTP - c_a_266r*ERBP_G_GABP_GAP_Ras_GTP	0.6225	$nM^{-1}\cdot s^{-1}$	7, 8, 33 (*S1.1*) + [1,16]
**347**	ERBP_G_GABP_GAP + Ras_GTP <-> ERBP_G_GABP_GAP_Ras_GTP	c_a_266r		0.3	s^{-1}	7, 8, 33 (*S1.1*) + [1,16]
**348**	ERBP_G_GABP_GAP_Ras_GTP -> ERBP_G_GABP_GAP + Ras_GDP	c_a_267	c_a_267*ERBP_G_GABP_GAP_Ras_GTP	1.494	s^{-1}	7, 8, 33 (*S1.1*) + [1,16]
**349**	ERBP_ShP_G_GABP_GAP + Ras_GTP <-> ERBP_ShP_G_GABP_GAP_Ras_GTP	c_a_268f	c_a_268f*ERBP_ShP_G_GABP_GAP*Ras_GTP - c_a_268r*ERBP_ShP_G_GABP_GAP_Ras_GTP	0.6225	$nM^{-1}\cdot s^{-1}$	7, 8, 33 (*S1.1*) + [1,16]
**350**	ERBP_ShP_G_GABP_GAP + Ras_GTP <-> ERBP_ShP_G_GABP_GAP_Ras_GTP	c_a_268r		0.3	s^{-1}	7, 8, 33 (*S1.1*) + [1,16]
**351**	ERBP_ShP_G_GABP_GAP_Ras_GTP -> ERBP_ShP_G_GABP_GAP + Ras_GDP	c_a_269	c_a_269*ERBP_ShP_G_GABP_GAP_Ras_GTP	1.494	s^{-1}	7, 8, 33 (*S1.1*) + [1,16]
**352**	ERBP + PI3K <-> ERBP_PK	c_a_270f	c_a_270f*ERBP*PI3K - c_a_270r*ERBP_PK	0.01	$nM^{-1}\cdot s^{-1}$	1 - 3, 31, 32 (*S1.1*) + [1,16]
**353**	ERBP + PI3K <-> ERBP_PK	c_a_270r		1	s^{-1}	1 - 3, 31, 32 (*S1.1*) + [1,16]
**354**	ERBP_PK -> ERBP_PKP	c_a_271f	c_a_271f*ERBP_PK	1	s^{-1}	1 - 3, 31, 32 (*S1.1*) + [1,16]
**355**	ERBP + PKP <-> ERBP_PKP	c_a_272r	c_a_272r*ERBP*PKP - c_a_272f*ERBP_PKP	0.01	$nM^{-1}\cdot s^{-1}$	1 - 3, 31, 32 (*S1.1*) + [1,16]
**356**	ERBP + PKP <-> ERBP_PKP	c_a_272f		0.1	s^{-1}	1 - 3, 31, 32 (*S1.1*) + [1,16]

**357**	ERBP_ShP_G_GABP + PhoA <-> ERBP_ShP_G_GABP_PhoA	c_a_278f	c_a_278f*ERBP_ShP_G_GABP*PhoA - c_a_278r*ERBP_ShP_G_GABP_PhoA	0.001	$nM^{-1}\cdot s^{-1}$	33 (*S1.1*) + [1,16]
**358**	ERBP_ShP_G_GABP + PhoA <-> ERBP_ShP_G_GABP_PhoA	c_a_278r		0.1	s^{-1}	33 (*S1.1*) + [1,16]
**359**	ERBP_ShP_G_GABP_PhoA -> ERBP_ShP_G_GAB + PhoA	c_a_280	c_a_280*ERBP_ShP_G_GABP_PhoA	0.03	s^{-1}	33 (*S1.1*) + [1,16]
**360**	ERBP_G_GABP + PhoA <-> ERBP_G_GABP_PhoA	c_a_279f	c_a_279f*ERBP_G_GABP*PhoA - c_a_279r*ERBP_G_GABP_PhoA	0.001	$nM^{-1}\cdot s^{-1}$	33 (*S1.1*) + [1,16]
**361**	ERBP_G_GABP + PhoA <-> ERBP_G_GABP_PhoA	c_a_279r		0.1	s^{-1}	33 (*S1.1*) + [1,16]
**362**	ERBP_G_GABP_PhoA -> ERBP_G_GAB + PhoA	c_a_281	c_a_281*ERBP_G_GABP_PhoA	0.03	s^{-1}	33 (*S1.1*) + [1,16]
**363**	ERBP_G_GABP_PKP + PIP2 <-> ERBP_G_GABP_PKP_PIP2	c_a_282f	c_a_282f*ERBP_G_GABP_PKP*PIP2 - c_a_282r*ERBP_G_GABP_PKP_PIP2	6.25	$nM^{-1}\cdot s^{-1}$	1 - 3, 31 - 33 (*S1.1*) + [1,16]
**364**	ERBP_G_GABP_PKP + PIP2 <-> ERBP_G_GABP_PKP_PIP2	c_a_282r		3.5	s^{-1}	1 - 3, 31 - 33 (*S1.1*) + [1,16]
**365**	ERBP_ShP_G_GABP_PKP + PIP2 <-> ERBP_ShP_G_GABP_PKP_PIP2	c_a_283f	c_a_283f*ERBP_ShP_G_GABP_PKP*PIP2 - c_a_283r*ERBP_ShP_G_GABP_PKP_PIP2	6.25	$nM^{-1}\cdot s^{-1}$	1 - 3, 31 - 33 (*S1.1*) + [1,16]
**366**	ERBP_ShP_G_GABP_PKP + PIP2 <-> ERBP_ShP_G_GABP_PKP_PIP2	c_a_283r		3.5	s^{-1}	1 - 3, 31 - 33 (*S1.1*) + [1,16]
**367**	ERBP_G_GABP_PKP_PIP2 -> ERBP_G_GABP_PKP + PIP3	c_a_284	c_a_284*ERBP_G_GABP_PKP_PIP2	25	s^{-1}	1 - 3, 31 - 33 (*S1.1*) + [1,16]
**368**	ERBP_ShP_G_GABP_PKP_PIP2 -> ERBP_ShP_G_GABP_PKP + PIP3	c_a_285	c_a_285*ERBP_ShP_G_GABP_PKP_PIP2	25	s^{-1}	1 - 3, 31 - 33 (*S1.1*) + [1,16]
**369**	ERBP_G_GABP_PKP + PIP3 <-> ERBP_G_GABP_PKP_PIP3	c_a_288f	c_a_288f*ERBP_G_GABP_PKP*PIP3 - c_a_288r*ERBP_G_GABP_PKP_PIP3	25	$nM^{-1}\cdot s^{-1}$	1 - 3, 31 - 33 (*S1.1*) + [1,16]
**370**	ERBP_G_GABP_PKP + PIP3 <-> ERBP_G_GABP_PKP_PIP3	c_a_288r		3	s^{-1}	1 - 3, 31 - 33 (*S1.1*) + [1,16]
**371**	ERBP_ShP_G_GABP_PKP + PIP3 <-> ERBP_ShP_G_GABP_PKP_PIP3	c_a_289f	c_a_289f*ERBP_ShP_G_GABP_PKP*PIP3 - c_a_289r*ERBP_ShP_G_GABP_PKP_PIP3	25	$nM^{-1}\cdot s^{-1}$	1 - 3, 31 - 33 (*S1.1*) + [1,16]
**372**	ERBP_ShP_G_GABP_PKP + PIP3 <-> ERBP_ShP_G_GABP_PKP_PIP3	c_a_289r		3	s^{-1}	1 - 3, 31 - 33 (*S1.1*) + [1,16]
**373**	ERBP_ShP_G + GABP <-> ERBP_ShP_G_GABP	c_a_290r	c_a_290r*ERBP_ShP_G*GABP - c_a_290f*ERBP_ShP_G_GABP	0.01	$nM^{-1}\cdot s^{-1}$	1 - 3, 31 - 33 (*S1.1*) + [1,16]
**374**	ERBP_ShP_G + GABP <-> ERBP_ShP_G_GABP	c_a_290f		1	s^{-1}	1 - 3, 31 - 33 (*S1.1*) + [1,16]
**375**	ERBP_G + GABP <-> ERBP_G_GABP	c_a_291r	c_a_291r*ERBP_G*GABP - c_a_291f*ERBP_G_GABP	0.01	$nM^{-1}\cdot s^{-1}$	1 - 3, 33 (*S1.1*) + [1,16]
**376**	ERBP_G + GABP <-> ERBP_G_GABP	c_a_291f		1	s^{-1}	1 - 3, 33 (*S1.1*) + [1,16]
**377**	ERBP_G + GAB_PIP3 <-> ERBP_G_GAB_PIP3	c_a_297f	c_a_297f*ERBP_G*GAB_PIP3 - c_a_297r*ERBP_G_GAB_PIP3	2.5	$nM^{-1}\cdot s^{-1}$	1 - 3, 33 (*S1.1*) + [1,16]

**378**	ERBP_G + GAB_PIP3 <-> ERBP_G_GAB_PIP3	c_a_297r		1	s^{-1}	1 - 3, 33 (*S1.1*) + [1,16]
**379**	ERBP_ShP_G + GAB_PIP3 <-> ERBP_ShP_G_GAB_PIP3	c_a_299r	c_a_299r*ERBP_ShP_G*GAB_PIP3 - c_a_299f*ERBP_ShP_G_GAB_PIP3	2.5	$nM^{-1}\cdot s^{-1}$	1 - 3, 33 (*S1.1*) + [1,16]
**380**	ERBP_ShP_G + GAB_PIP3 <-> ERBP_ShP_G_GAB_PIP3	c_a_299f		1	s^{-1}	1 - 3, 33 (*S1.1*) + [1,16]
**381**	ERBP_ShP_G_GAB_PIP3 -> ERBP_ShP_G_GABP + PIP3	c_a_300	c_a_300*ERBP_ShP_G_GAB_PIP3	0.01	s^{-1}	1 - 3, 33 (*S1.1*) + [1,16]
**382**	ERBP_G_GABP_PKP + PhoB <-> ERBP_G_GABP_PKP_PhoB	c_a_317f	c_a_317f*ERBP_G_GABP_PKP*PhoB - c_a_317r*ERBP_G_GABP_PKP_PhoB	0.001	$nM^{-1}\cdot s^{-1}$	1 - 3, 33 (*S1.1*) + [1,16]
**383**	ERBP_G_GABP_PKP + PhoB <-> ERBP_G_GABP_PKP_PhoB	c_a_317r		0.038	s^{-1}	1 - 3, 33 (*S1.1*) + [1,16]
**384**	ERBP_G_GABP_PKP_PhoB -> ERBP_G_GABP_PK + PhoB	c_a_318	c_a_318*ERBP_G_GABP_PKP_PhoB	0.595	s^{-1}	1 - 3, 33 (*S1.1*) + [1,16]
**385**	ERBP_ShP_G_GABP_PKP + PhoB <-> ERBP_ShP_G_GABP_PKP_PhoB	c_a_319f	c_a_319f*ERBP_ShP_G_GABP_PKP*PhoB - c_a_319r*ERBP_ShP_G_GABP_PKP_PhoB	0.001	$nM^{-1}\cdot s^{-1}$	1 - 3, 33 (*S1.1*) + [1,16]
**386**	ERBP_ShP_G_GABP_PKP + PhoB <-> ERBP_ShP_G_GABP_PKP_PhoB	c_a_319r		0.038	s^{-1}	1 - 3, 33 (*S1.1*) + [1,16]
**387**	ERBP_ShP_G_GABP_PKP_PhoB -> ERBP_ShP_G_GABP_PK + PhoB	c_a_320	c_a_320*ERBP_ShP_G_GABP_PKP_PhoB	0.595	s^{-1}	1 - 3, 33 (*S1.1*) + [1,16]
**388**	ERBP_ShP_G_GAB + PIP3 <-> ERBP_ShP_G_GAB_PIP3	c_a_323f	c_a_323f*ERBP_ShP_G_GAB*PIP3 - c_a_323r*ERBP_ShP_G_GAB_PIP3	2.5	$nM^{-1}\cdot s^{-1}$	1 - 3, 33 (*S1.1*) + [1,16]
**389**	ERBP_ShP_G_GAB + PIP3 <-> ERBP_ShP_G_GAB_PIP3	c_a_323r		1	s^{-1}	1 - 3, 33 (*S1.1*) + [1,16]
**390**	ERBP_PKP + PIP2 <-> ERBP_PKP_PIP2	c_a_326f	c_a_326f*ERBP_PKP*PIP2 - c_a_326r*ERBP_PKP_PIP2	6.25	$nM^{-1}\cdot s^{-1}$	1 – 3, 31, 32 (*S1.1*) + [1,16]
**391**	ERBP_PKP + PIP2 <-> ERBP_PKP_PIP2	c_a_326r		3.5	s^{-1}	1 – 3, 31, 32 (*S1.1*) + [1,16]
**392**	ERBP_PKP_PIP2 -> ERBP_PKP + PIP3	c_a_327f	c_a_327f*ERBP_PKP_PIP2	2.5	s^{-1}	1 – 3, 31, 32 (*S1.1*) + [1,16]
**393**	ERBP_ShP_G_GABP_PKP <-> ERBP_ShP_G_GABP + PKP	c_a_328f	c_a_328f*ERBP_ShP_G_GABP_PKP - c_a_328r*ERBP_ShP_G_GABP*PKP	5	s^{-1}	1 – 3, 31, 32 (*S1.1*) + [1,16]
**394**	ERBP_ShP_G_GABP_PKP <-> ERBP_ShP_G_GABP + PKP	c_a_328r		0.05	$nM^{-1}\cdot s^{-1}$	1 – 3, 31, 32 (*S1.1*) + [1,16]
**395**	ERBP_G_GABP_PKP <-> ERBP_G_GABP + PKP	c_a_329f	c_a_329f*ERBP_G_GABP_PKP - c_a_329r*ERBP_G_GABP*PKP	5	s^{-1}	1 – 3, 31, 32 (*S1.1*) + [1,16]
**396**	ERBP_G_GABP_PKP <-> ERBP_G_GABP + PKP	c_a_329r		0.05	$nM^{-1}\cdot s^{-1}$	1 – 3, 31, 32 (*S1.1*) + [1,16]
**397**	ERBP_G_GAB_PIP3 -> ERBP_G_GABP + PIP3	c_a_330	c_a_330*ERBP_G_GAB_PIP3	0.01	s^{-1}	1 – 3, 31, 32 (*S1.1*) + [1,16]

**398**	ERBP_G_GAB + PIP3 <-> ERBP_G_GAB_PIP3	c_a_336f	c_a_336f*ERBP_G_GAB*PIP3 - c_a_336r*ERBP_G_GAB_PIP3	1	$nM^{-1}\cdot s^{-1}$	1 – 3, 31, 32 (*S1.1*) + [1,16]
**399**	ERBP_G_GAB + PIP3 <-> ERBP_G_GAB_PIP3	c_a_336r		2.5	s^{-1}	1 – 3, 31, 32 (*S1.1*) + [1,16]
**400**	ERB3P + PLCy <-> ERB3P_PL	c_b_185f	c_b_185f*ERB3P*PLCy - c_b_185r*ERB3P_PL	0.06	$nM^{-1}\cdot s^{-1}$	1 – 3 (*S1.1*) + [1,16]
**401**	ERB3P + PLCy <-> ERB3P_PL	c_b_185r		0.2	s^{-1}	1 – 3 (*S1.1*) + [1,16]
**402**	ERB3P_PL -> ERB3P_PLP	c_b_186f	c_b_186f*ERB3P_PL	1	s^{-1}	1 – 3 (*S1.1*) + [1,16]
**403**	ERB3P_PLP <-> ERB3P + PLCyP	c_b_187f	c_b_187f*ERB3P_PLP - c_b_187r*ERB3P*PLCyP	0.3	s^{-1}	1 – 3 (*S1.1*) + [1,16]
**404**	ERB3P_PLP <-> ERB3P + PLCyP	c_b_187r		0.006	$nM^{-1}\cdot s^{-1}$	1 – 3 (*S1.1*) + [1,16]
**405**	ERB3P + Grb <-> ERB3P_G	c_b_190f	c_b_190f*ERB3P*Grb - c_b_190r*ERB3P_G	0.0015	$nM^{-1}\cdot s^{-1}$	1 – 5 (*S1.1*) + [1,16]
**406**	ERB3P + Grb <-> ERB3P_G	c_b_190r		0.2	s^{-1}	1 – 5 (*S1.1*) + [1,16]
**407**	ERB3P_G + SOS <-> ERB3P_G_S	c_b_191f	c_b_191f*ERB3P_G*SOS - c_b_191r*ERB3P_G_S	0.01	$nM^{-1}\cdot s^{-1}$	1 – 5 (*S1.1*) + [1,16]
**408**	ERB3P_G + SOS <-> ERB3P_G_S	c_b_191r		0.06	s^{-1}	1 – 5 (*S1.1*) + [1,16]
**409**	ERB3P_G_S <-> ERB3P + G_S	c_b_192f	c_b_192f*ERB3P_G_S - c_b_192r*ERB3P*G_S	0.15	s^{-1}	1 – 5 (*S1.1*) + [1,16]
**410**	ERB3P_G_S <-> ERB3P + G_S	c_b_192r		0.0028	$nM^{-1}\cdot s^{-1}$	1 – 5 (*S1.1*) + [1,16]
**411**	ERB3P + Shc <-> ERB3P_Sh	c_b_194f	c_b_194f*ERB3P*Shc - c_b_194r*ERB3P_Sh	0.09	$nM^{-1}\cdot s^{-1}$	1 – 5 (*S1.1*) + [1,16]
**412**	ERB3P + Shc <-> ERB3P_Sh	c_b_194r		0.6	s^{-1}	1 – 5 (*S1.1*) + [1,16]
**413**	ERB3P_Sh -> ERB3P_ShP	c_b_195f	c_b_195f*ERB3P_Sh	6	s^{-1}	1 – 5 (*S1.1*) + [1,16]
**414**	ERB3P_ShP <-> ShP + ERB3P	c_b_196f	c_b_196f*ERB3P_ShP - c_b_196r*ShP*ERB3P	0.3	s^{-1}	1 – 5 (*S1.1*) + [1,16]
**415**	ERB3P_ShP <-> ShP + ERB3P	c_b_196r		0.0009	$nM^{-1}\cdot s^{-1}$	1 – 5 (*S1.1*) + [1,16]
**416**	ERB3P_ShP + Grb <-> ERB3P_ShP_G	c_b_197f	c_b_197f*ERB3P_ShP*Grb - c_b_197r*ERB3P_ShP_G	0.003	$nM^{-1}\cdot s^{-1}$	1 – 5 (*S1.1*) + [1,16]
**417**	ERB3P_ShP + Grb <-> ERB3P_ShP_G	c_b_197r		0.1	s^{-1}	1 – 5 (*S1.1*) + [1,16]
**418**	ERB3P_ShP_G <-> ERB3P + ShP_G	c_b_198f	c_b_198f*ERB3P_ShP_G - c_b_198r*ERB3P*ShP_G	0.3	s^{-1}	1 – 5 (*S1.1*) + [1,16]
**419**	ERB3P_ShP_G <-> ERB3P + ShP_G	c_b_198r		0.0009	$nM^{-1}\cdot s^{-1}$	1 – 5 (*S1.1*) + [1,16]
**420**	ERB3P_ShP_G + SOS <-> ERB3P_ShP_G_S	c_b_199f	c_b_199f*ERB3P_ShP_G*SOS - c_b_199r*ERB3P_ShP_G_S	0.01	$nM^{-1}\cdot s^{-1}$	1 – 6 (*S1.1*) + [1,16]
**421**	ERB3P_ShP_G + SOS <-> ERB3P_ShP_G_S	c_b_199r		0.0214	s^{-1}	1 – 6 (*S1.1*) + [1,16]
**422**	ERB3P_ShP_G_S <-> ShP_G_S + ERB3P	c_b_200f	c_b_200f*ERB3P_ShP_G_S - c_b_200r*ShP_G_S*ERB3P	0.12	s^{-1}	1 – 6 (*S1.1*) + [1,16]
**423**	ERB3P_ShP_G_S <-> ShP_G_S + ERB3P	c_b_200r		0.00024	$nM^{-1}\cdot s^{-1}$	1 – 6 (*S1.1*) + [1,16]
**424**	ERB3P_ShP + G_S <-> ERB3P_ShP_G_S	c_b_205f	c_b_205f*ERB3P_ShP*G_S - c_b_205r*ERB3P_ShP_G_S	0.009	$nM^{-1}\cdot s^{-1}$	1 – 6 (*S1.1*) + [1,16]

**425**	ERB3P_ShP + G_S <-> ERB3P_ShP_G_S	c_b_205r		0.0429	s^{-1}	1 – 6 (*S1.1*) + [1,16]
**426**	ERB3P_ShP_G_S + Ras_GDP <-> ERB3P_Sh_G_S_Ras_GDP	c_b_209f	c_b_209f*ERB3P_ShP_G_S*Ras_GDP - c_b_209r*ERB3P_Sh_G_S_Ras_GDP	0.00475	$nM^{-1}\cdot s^{-1}$	1 – 8 (*S1.1*) + [1,16]
**427**	ERB3P_ShP_G_S + Ras_GDP <-> ERB3P_Sh_G_S_Ras_GDP	c_b_209r		0.76	s^{-1}	1 – 8 (*S1.1*) + [1,16]
**428**	ERB3P_Sh_G_S_Ras_GDP <-> ERB3P_Sh_G_S_Ras + GDP	c_b_210f	c_b_210f*ERB3P_Sh_G_S_Ras_GDP - c_b_210r*ERB3P_Sh_G_S_Ras*GDP	46.5	s^{-1}	1 – 8 (*S1.1*) + [1,16]
**429**	ERB3P_Sh_G_S_Ras_GDP <-> ERB3P_Sh_G_S_Ras + GDP	c_b_210r		0.093	$nM^{-1}\cdot s^{-1}$	1 – 8 (*S1.1*) + [1,16]
**430**	ERB3P_Sh_G_S_Ras + GTP <-> ERB3P_Sh_G_S_Ras_GTP	c_b_211f	c_b_211f*ERB3P_Sh_G_S_Ras*GTP - c_b_211r*ERB3P_Sh_G_S_Ras_GTP	0.003	$nM^{-1}\cdot s^{-1}$	1 – 8 (*S1.1*) + [1,16]
**431**	ERB3P_Sh_G_S_Ras + GTP <-> ERB3P_Sh_G_S_Ras_GTP	c_b_211r		2.4	s^{-1}	1 – 8 (*S1.1*) + [1,16]
**432**	ERB3P_Sh_G_S_Ras_GTP <-> ERB3P_ShP_G_S + Ras_GTP	c_b_212f	c_b_212f*ERB3P_Sh_G_S_Ras_GTP - c_b_212r*ERB3P_ShP_G_S*Ras_GTP	806.4	s^{-1}	1 – 8 (*S1.1*) + [1,16]
**433**	ERB3P_Sh_G_S_Ras_GTP <-> ERB3P_ShP_G_S + Ras_GTP	c_b_212r		1.575	$nM^{-1}\cdot s^{-1}$	1 – 8 (*S1.1*) + [1,16]
**434**	ERB3P_ShP_G_S + Ras <-> ERB3P_Sh_G_S_Ras	c_b_213f	c_b_213f*ERB3P_ShP_G_S*Ras - c_b_213r*ERB3P_Sh_G_S_Ras	0.15625	$nM^{-1}\cdot s^{-1}$	1 – 8 (*S1.1*) + [1,16]
**435**	ERB3P_ShP_G_S + Ras <-> ERB3P_Sh_G_S_Ras	c_b_213r		0.001	s^{-1}	1 – 8 (*S1.1*) + [1,16]
**436**	ERB3P_G_S + Ras_GDP <-> ERB3P_G_S_Ras_GDP	c_b_214f	c_b_214f*ERB3P_G_S*Ras_GDP - c_b_214r*ERB3P_G_S_Ras_GDP	0.0075	$nM^{-1}\cdot s^{-1}$	1 – 8 (*S1.1*) + [1,16]
**437**	ERB3P_G_S + Ras_GDP <-> ERB3P_G_S_Ras_GDP	c_b_214r		1.2	s^{-1}	1 – 8 (*S1.1*) + [1,16]
**438**	ERB3P_G_S_Ras_GDP <-> ERB3P_G_S_Ras + GDP	c_b_215f	c_b_215f*ERB3P_G_S_Ras_GDP - c_b_215r*ERB3P_G_S_Ras*GDP	50	s^{-1}	1 – 8 (*S1.1*) + [1,16]
**439**	ERB3P_G_S_Ras_GDP <-> ERB3P_G_S_Ras + GDP	c_b_215r		0.1	$nM^{-1}\cdot s^{-1}$	1 – 8 (*S1.1*) + [1,16]
**440**	ERB3P_G_S_Ras + GTP <-> ERB3P_G_S_Ras_GTP	c_b_216f	c_b_216f*ERB3P_G_S_Ras*GTP - c_b_216r*ERB3P_G_S_Ras_GTP	0.1	$nM^{-1}\cdot s^{-1}$	1 – 8 (*S1.1*) + [1,16]
**441**	ERB3P_G_S_Ras + GTP <-> ERB3P_G_S_Ras_GTP	c_b_216r		80	s^{-1}	1 – 8 (*S1.1*) + [1,16]
**442**	ERB3P_G_S_Ras_GTP <-> ERB3P_G_S + Ras_GTP	c_b_217f	c_b_217f*ERB3P_G_S_Ras_GTP - c_b_217r*ERB3P_G_S*Ras_GTP	640	s^{-1}	1 – 8 (*S1.1*) + [1,16]
**443**	ERB3P_G_S_Ras_GTP <-> ERB3P_G_S + Ras_GTP	c_b_217r		1.25	$nM^{-1}\cdot s^{-1}$	1 – 8 (*S1.1*) + [1,16]
**444**	ERB3P_G_S + Ras <-> ERB3P_G_S_Ras	c_b_218f	c_b_218f*ERB3P_G_S*Ras - c_b_218r*ERB3P_G_S_Ras	0.25	$nM^{-1}\cdot s^{-1}$	1 – 8 (*S1.1*) + [1,16]
**445**	ERB3P_G_S + Ras <-> ERB3P_G_S_Ras	c_b_218r		0.0016	s^{-1}	1 – 8 (*S1.1*) + [1,16]

**446**	GAP + ERB3P <-> ERB3P_GAP	c_b_239f	c_b_239f*GAP*ERB3P - c_b_239r*ERB3P_GAP	0.083	$nM^{-1}\cdot s^{-1}$	1 – 8 (*S1.1*) + [1,16]
**447**	GAP + ERB3P <-> ERB3P_GAP	c_b_239r		0.15	s^{-1}	1 – 8 (*S1.1*) + [1,16]
**448**	Ras_GTP + ERB3P_GAP <-> ERB3P_GAP_Ras_GTP	c_b_240f	c_b_240f*Ras_GTP*ERB3P_GAP - c_b_240r*ERB3P_GAP_Ras_GTP	0.01	$nM^{-1}\cdot s^{-1}$	1 – 8 (*S1.1*) + [1,16]
**449**	Ras_GTP + ERB3P_GAP <-> ERB3P_GAP_Ras_GTP	c_b_240r		0.03	s^{-1}	1 – 8 (*S1.1*) + [1,16]
**450**	ERB3P_GAP_Ras_GTP -> ERB3P_GAP + Ras_GDP	c_b_241	c_b_241*ERB3P_GAP_Ras_GTP	1.494	s^{-1}	1 – 8 (*S1.1*) + [1,16]
**451**	ERB3P_ShP_G_S + ERKPP <-> ERB3P_Sh_G_S_ERKPP	c_b_250f	c_b_250f*ERB3P_ShP_G_S*ERKPP - c_b_250r*ERB3P_Sh_G_S_ERKPP	0.01	$nM^{-1}\cdot s^{-1}$	11, 24, 25 (*S1.1*) + [1,16]
**452**	ERB3P_ShP_G_S + ERKPP <-> ERB3P_Sh_G_S_ERKPP	c_b_250r		0.033	s^{-1}	11, 24, 25 (*S1.1*) + [1,16]
**453**	ERB3P_Sh_G_S_ERKPP -> ERB3P_ShP_G + ERKPP + SOSP	c_b_251	c_b_251*ERB3P_Sh_G_S_ERKPP	1	s^{-1}	11, 24, 25 (*S1.1*) + [1,16]
**454**	ERB3P_G_S + ERKPP <-> ERB3P_G_S_ERKPP	c_b_252f	c_b_252f*ERB3P_G_S*ERKPP - c_b_252r*ERB3P_G_S_ERKPP	0.01	$nM^{-1}\cdot s^{-1}$	11, 24, 25 (*S1.1*) + [1,16]
**455**	ERB3P_G_S + ERKPP <-> ERB3P_G_S_ERKPP	c_b_252r		0.033	s^{-1}	11, 24, 25 (*S1.1*) + [1,16]
**456**	ERB3P_G_S_ERKPP -> ERB3P_G + SOSP + ERKPP	c_b_253	c_b_253*ERB3P_G_S_ERKPP	1	s^{-1}	11, 24, 25 (*S1.1*) + [1,16]
**457**	ERB3P_G + GAB <-> ERB3P_G_GAB	c_b_256f	c_b_256f*ERB3P_G*GAB - c_b_256r*ERB3P_G_GAB	0.01	$nM^{-1}\cdot s^{-1}$	33 (*S1.1*) + [1,16]
**458**	ERB3P_G + GAB <-> ERB3P_G_GAB	c_b_256r		1	s^{-1}	33 (*S1.1*) + [1,16]
**459**	ERB3P_ShP_G + GAB <-> ERB3P_ShP_G_GAB	c_b_257f	c_b_257f*ERB3P_ShP_G*GAB - c_b_257r*ERB3P_ShP_G_GAB	0.01	$nM^{-1}\cdot s^{-1}$	33 (*S1.1*) + [1,16]
**460**	ERB3P_ShP_G + GAB <-> ERB3P_ShP_G_GAB	c_b_257r		1	s^{-1}	33 (*S1.1*) + [1,16]
**461**	ERB3P_G_GAB -> ERB3P_G_GABP	c_b_258	c_b_258*ERB3P_G_GAB	0.05	s^{-1}	33 (*S1.1*) + [1,16]
**462**	ERB3P_ShP_G_GAB -> ERB3P_ShP_G_GABP	c_b_259	c_b_259*ERB3P_ShP_G_GAB	0.05	s^{-1}	33 (*S1.1*) + [1,16]
**463**	ERB3P_G_GABP + PI3K <-> ERB3P_G_GABP_PK	c_b_260f	c_b_260f*ERB3P_G_GABP*PI3K - c_b_260r*ERB3P_G_GABP_PK	0.01	$nM^{-1}\cdot s^{-1}$	33 (*S1.1*) + [12-13,16]
**464**	ERB3P_G_GABP + PI3K <-> ERB3P_G_GABP_PK	c_b_260r		1	s^{-1}	33 (*S1.1*) + [12-13,16]
**465**	ERB3P_G_GABP_PK -> ERB3P_G_GABP_PKP	c_b_262	c_b_262*ERB3P_G_GABP_PK	1	s^{-1}	33 (*S1.1*) + [12-13,16]
**466**	ERB3P_ShP_G_GABP + PI3K <-> ERB3P_ShP_G_GABP_PK	c_b_261f	c_b_261f*ERB3P_ShP_G_GABP*PI3K - c_b_261r*ERB3P_ShP_G_GABP_PK	0.01	$nM^{-1}\cdot s^{-1}$	33 (*S1.1*) + [12-13,16]
**467**	ERB3P_ShP_G_GABP + PI3K <-> ERB3P_ShP_G_GABP_PK	c_b_261r		1	s^{-1}	33 (*S1.1*) + [12-13,16]

**468**	ERB3P_ShP_G_GABP_PK -> ERB3P_ShP_G_GABP_PKP	c_b_263	c_b_263*ERB3P_ShP_G_GABP_PK	1	s^{-1}	33 (*S1.1*) + [12-13,16]
**469**	ERB3P_ShP_G_GABP + GAP <-> ERB3P_ShP_G_GABP_GAP	c_b_264f	c_b_264f*ERB3P_ShP_G_GABP*GAP - c_b_264r*ERB3P_ShP_G_GABP_GAP	0.083	$nM^{-1}\cdot s^{-1}$	33 (*S1.1*) + [1,16]
**470**	ERB3P_ShP_G_GABP + GAP <-> ERB3P_ShP_G_GABP_GAP	c_b_264r		0.15	s^{-1}	33 (*S1.1*) + [12-13,16]
**471**	ERB3P_G_GABP + GAP <-> ERB3P_G_GABP_GAP	c_b_265f	c_b_265f*ERB3P_G_GABP*GAP - c_b_265r*ERB3P_G_GABP_GAP	0.083	$nM^{-1}\cdot s^{-1}$	33 (*S1.1*) + [1,16]
**472**	ERB3P_G_GABP + GAP <-> ERB3P_G_GABP_GAP	c_b_265r		0.15	s^{-1}	33 (*S1.1*) + [12-13,16]
**473**	ERB3P_G_GABP_GAP + Ras_GTP <-> ERB3P_G_GABP_GAP_Ras_GTP	c_b_266f	c_b_266f*ERB3P_G_GABP_GAP*Ras_GTP - c_b_266r*ERB3P_G_GABP_GAP_Ras_GTP	0.6225	$nM^{-1}\cdot s^{-1}$	7, 8, 33 (*S1.1*) + [12-13,16]
**474**	ERB3P_G_GABP_GAP + Ras_GTP <-> ERB3P_G_GABP_GAP_Ras_GTP	c_b_266r		0.3	s^{-1}	7, 8, 33 (*S1.1*) + [12-13,16]
**475**	ERB3P_G_GABP_GAP_Ras_GTP -> ERB3P_G_GABP_GAP + Ras_GDP	c_b_267f	c_b_267f*ERB3P_G_GABP_GAP_Ras_GTP	1.494	s^{-1}	7, 8, 33 (*S1.1*) + [12-13,16]
**476**	ERB3P_ShP_G_GABP_GAP + Ras_GTP <-> ERB3P_ShP_G_GABP_GAP_Ras_GTP	c_b_268f	c_b_268f*ERB3P_ShP_G_GABP_GAP*Ras_GTP - c_b_268r*ERB3P_ShP_G_GABP_GAP_Ras_GTP	0.6225	$nM^{-1}\cdot s^{-1}$	7, 8, 33 (*S1.1*) + [12-13,16]
**477**	ERB3P_ShP_G_GABP_GAP + Ras_GTP <-> ERB3P_ShP_G_GABP_GAP_Ras_GTP	c_b_268r		0.3	s^{-1}	7, 8, 33 (*S1.1*) + [12-13,16]
**478**	ERB3P_ShP_G_GABP_GAP_Ras_GTP -> ERB3P_ShP_G_GABP_GAP + Ras_GDP	c_b_269	c_b_269*ERB3P_ShP_G_GABP_GAP_Ras_GTP	1.494	s^{-1}	7, 8, 33 (*S1.1*) + [12-13,16]
**479**	ERB3P + PI3K <-> ERB3P_PK	c_b_270f	c_b_270f*ERB3P*PI3K - c_b_270r*ERB3P_PK	0.01	$nM^{-1}\cdot s^{-1}$	1 – 3, 31 - 32 (*S1.1*) + [1,16]
**480**	ERB3P + PI3K <-> ERB3P_PK	c_b_270r		1	s^{-1}	1 – 3, 31-32 (*S1.1*) + [12-13,16]
**481**	ERB3P_PK -> ERB3P_PKP	c_b_271f	c_b_271f*ERB3P_PK	1	s^{-1}	1 – 3, 31-32 (*S1.1*) + [12-13,16]
**482**	ERB3P + PKP <-> ERB3P_PKP	c_b_272r	c_b_272r*ERB3P*PKP - c_b_272f*ERB3P_PKP	0.01	$nM^{-1}\cdot s^{-1}$	31-32 (*S1.1*) + [12-13,16]
**483**	ERB3P + PKP <-> ERB3P_PKP	c_b_272f		0.1	s^{-1}	31-32 (*S1.1*) + [12-13,16]
**484**	ERB3P_ShP_G_GABP + PhoA <-> ERB3P_ShP_G_GABP_PhoA	c_b_278f	c_b_278f*ERB3P_ShP_G_GABP*PhoA - c_b_278r*ERB3P_ShP_G_GABP_PhoA	0.001	$nM^{-1}\cdot s^{-1}$	33 (*S1.1*) + [1,13,16]
**485**	ERB3P_ShP_G_GABP + PhoA <-> ERB3P_ShP_G_GABP_PhoA	c_b_278r		0.1	s^{-1}	33 (*S1.1*) + [11,13,16]
**486**	ERB3P_ShP_G_GABP_PhoA -> ERB3P_ShP_G_GAB + PhoA	c_b_280	c_b_280*ERB3P_ShP_G_GABP_PhoA	0.03	s^{-1}	33 (*S1.1*) + [11,13,16]
**487**	ERB3P_G_GABP + PhoA <-> ERB3P_G_GABP_PhoA	c_b_279f	c_b_279f*ERB3P_G_GABP*PhoA - c_b_279r*ERB3P_G_GABP_PhoA	0.001	$nM^{-1}\cdot s^{-1}$	33 (*S1.1*) + [11,13,16]
**488**	ERB3P_G_GABP + PhoA <-> ERB3P_G_GABP_PhoA	c_b_279r		0.1	s^{-1}	33 (*S1.1*) + [11,13,16]

**489**	ERB3P_G_GABP_PhoA -> ERB3P_G_GAB + PhoA	c_b_281	c_b_281*ERB3P_G_GABP_PhoA	0.03	s^{-1}	33 (*S1.1*) + [11,13,16]
**490**	ERB3P_G_GABP_PKP + PIP2 <-> ERB3P_G_GABP_PKP_PIP2	c_b_282f	c_b_282f*ERB3P_G_GABP_PKP*PIP2 - c_b_282r*ERB3P_G_GABP_PKP_PIP2	6.25	$nM^{-1}\cdot s^{-1}$	31 - 33 (*S1.1*) + [11,13,16]
**491**	ERB3P_G_GABP_PKP + PIP2 <-> ERB3P_G_GABP_PKP_PIP2	c_b_282r		3.5	s^{-1}	31 - 33 (*S1.1*) + [11,13,16]
**492**	ERB3P_ShP_G_GABP_PKP + PIP2 <-> ERB3P_ShP_G_GABP_PKP_PIP2	c_b_283f	c_b_283f*ERB3P_ShP_G_GABP_PKP*PIP2 - c_b_283r*ERB3P_ShP_G_GABP_PKP_PIP2	6.25	$nM^{-1}\cdot s^{-1}$	31 - 33 (*S1.1*) + [11,13,16]
**493**	ERB3P_ShP_G_GABP_PKP + PIP2 <-> ERB3P_ShP_G_GABP_PKP_PIP2	c_b_283r		3.5	s^{-1}	31 - 33 (*S1.1*) + [11,13,16]
**494**	ERB3P_G_GABP_PKP_PIP2 -> ERB3P_G_GABP_PKP + PIP3	c_b_284	c_b_284*ERB3P_G_GABP_PKP_PIP2	25	s^{-1}	31 - 33 (*S1.1*) + [11,13,16]
**495**	ERB3P_ShP_G_GABP_PKP_PIP2 -> ERB3P_ShP_G_GABP_PKP + PIP3	c_b_285	c_b_285*ERB3P_ShP_G_GABP_PKP_PIP2	25	s^{-1}	31 - 33 (*S1.1*) + [11,13,16]
**496**	ERB3P_ShP_G + GABP <-> ERB3P_ShP_G_GABP	c_b_290r	c_b_290r*ERB3P_ShP_G*GABP - c_b_290f*ERB3P_ShP_G_GABP	0.01	$nM^{-1}\cdot s^{-1}$	33 (*S1.1*) + [11,13,16]
**497**	ERB3P_ShP_G + GABP <-> ERB3P_ShP_G_GABP	c_b_290f		1	s^{-1}	33 (*S1.1*) + [11,13,16]
**498**	ERB3P_G + GABP <-> ERB3P_G_GABP	c_b_291r	c_b_291r*ERB3P_G*GABP - c_b_291f*ERB3P_G_GABP	0.01	$nM^{-1}\cdot s^{-1}$	33 (*S1.1*) + [11,13,16]
**499**	ERB3P_G + GABP <-> ERB3P_G_GABP	c_b_291f		1	s^{-1}	33 (*S1.1*) + [11,13,16]
**500**	ERB3P_G + GAB_PIP3 <-> ERB3P_G_GAB_PIP3	c_b_297f	c_b_297f*ERB3P_G*GAB_PIP3 - c_b_297r*ERB3P_G_GAB_PIP3	2.5	$nM^{-1}\cdot s^{-1}$	33 (*S1.1*) + [11,13,16]
**501**	ERB3P_G + GAB_PIP3 <-> ERB3P_G_GAB_PIP3	c_b_297r		1	s^{-1}	33 (*S1.1*) + [11,13,16]
**502**	ERB3P_ShP_G + GAB_PIP3 <-> ERB3P_ShP_G_GAB_PIP3	c_b_299r	c_b_299r*ERB3P_ShP_G*GAB_PIP3 - c_b_299f*ERB3P_ShP_G_GAB_PIP3	2.5	$nM^{-1}\cdot s^{-1}$	33 (*S1.1*) + [11,13,16]
**503**	ERB3P_ShP_G + GAB_PIP3 <-> ERB3P_ShP_G_GAB_PIP3	c_b_299f		1	s^{-1}	33 (*S1.1*) + [11,13,16]
**504**	ERB3P_ShP_G_GAB_PIP3 -> ERB3P_ShP_G_GABP + PIP3	c_b_300	c_b_300*ERB3P_ShP_G_GAB_PIP3	0.01	s^{-1}	33 (*S1.1*) + [11,13,16]
**505**	ERB3P_G_GABP_PKP + PhoB <-> ERB3P_G_GABP_PKP_PhoB	c_b_317f	c_b_317f*ERB3P_G_GABP_PKP*PhoB - c_b_317r*ERB3P_G_GABP_PKP_PhoB	0.001	$nM^{-1}\cdot s^{-1}$	31 - 33 (*S1.1*) + [11,13,16]
**506**	ERB3P_G_GABP_PKP + PhoB <-> ERB3P_G_GABP_PKP_PhoB	c_b_317r		0.038	s^{-1}	31 - 33 (*S1.1*) + [11,13,16]
**507**	ERB3P_G_GABP_PKP_PhoB -> ERB3P_G_GABP_PK + PhoB	c_b_318	c_b_318*ERB3P_G_GABP_PKP_PhoB	0.595	s^{-1}	31 - 33 (*S1.1*) + [11,13,16]
**508**	ERB3P_ShP_G_GABP_PKP + PhoB <-> ERB3P_ShP_G_GABP_PKP_PhoB	c_b_319f	c_b_319f*ERB3P_ShP_G_GABP_PKP*PhoB - c_b_319r*ERB3P_ShP_G_GABP_PKP_PhoB	0.001	$nM^{-1}\cdot s^{-1}$	31 - 33 (*S1.1*) + [11,13,16]
**509**	ERB3P_ShP_G_GABP_PKP + PhoB <-> ERB3P_ShP_G_GABP_PKP_PhoB	c_b_319r		0.038	s^{-1}	31 - 33 (*S1.1*) + [11,13,16]

**510**	ERB3P_ShP_G_GABP_PKP_PhoB -> ERB3P_ShP_G_GABP_PK + PhoB	c_b_320	c_b_320*ERB3P_ShP_G_GABP_PKP_PhoB	0.595	s^{-1}	31 - 33 (*S1.1*) + [11,13,16]
**511**	ERB3P_ShP_G_GAB + PIP3 <-> ERB3P_ShP_G_GAB_PIP3	c_b_323f	c_b_323f*ERB3P_ShP_G_GAB*PIP3 - c_b_323r*ERB3P_ShP_G_GAB_PIP3	2.5	$nM^{-1}\cdot s^{-1}$	31 - 33 (*S1.1*) + [11,13,16]
**512**	ERB3P_ShP_G_GAB + PIP3 <-> ERB3P_ShP_G_GAB_PIP3	c_b_323r		1	s^{-1}	31 - 33 (*S1.1*) + [11,13,16]
**513**	ERB3P_PKP + PIP2 <-> ERB3P_PKP_PIP2	c_b_326f	c_b_326f*ERB3P_PKP*PIP2 - c_b_326r*ERB3P_PKP_PIP2	6.25	$nM^{-1}\cdot s^{-1}$	31 - 32 (*S1.1*) + [11,13,16]
**514**	ERB3P_PKP + PIP2 <-> ERB3P_PKP_PIP2	c_b_326r		3.5	s^{-1}	31 - 32 (*S1.1*) + [11,13,16]
**515**	ERB3P_PKP_PIP2 -> ERB3P_PKP + PIP3	c_b_327f	c_b_327f*ERB3P_PKP_PIP2	2.5	s^{-1}	31 - 32 (*S1.1*) + [11,13,16]
**516**	ERB3P_ShP_G_GABP_PKP <-> ERB3P_ShP_G_GABP + PKP	c_b_328f	c_b_328f*ERB3P_ShP_G_GABP_PKP - c_b_328r*ERB3P_ShP_G_GABP*PKP	5	s^{-1}	31 - 33 (*S1.1*) + [11,13,16]
**517**	ERB3P_ShP_G_GABP_PKP <-> ERB3P_ShP_G_GABP + PKP	c_b_328r		0.05	$nM^{-1}\cdot s^{-1}$	31 - 33 (*S1.1*) + [11,13,16]
**518**	ERB3P_G_GABP_PKP <-> ERB3P_G_GABP + PKP	c_b_329f	c_b_329f*ERB3P_G_GABP_PKP - c_b_329r*ERB3P_G_GABP*PKP	5	s^{-1}	31 - 33 (*S1.1*) + [11,13,16]
**519**	ERB3P_G_GABP_PKP <-> ERB3P_G_GABP + PKP	c_b_329r		0.05	$nM^{-1}\cdot s^{-1}$	31 - 33 (*S1.1*) + [11,13,16]
**520**	ERB3P_G_GAB_PIP3 -> ERB3P_G_GABP + PIP3	c_b_330	c_b_330*ERB3P_G_GAB_PIP3	0.01	s^{-1}	31 - 33 (*S1.1*) + [11,13,16]
**521**	ERB3P_G_GAB + PIP3 <-> ERB3P_G_GAB_PIP3	c_b_336f	c_b_336f*ERB3P_G_GAB*PIP3 - c_b_336r*ERB3P_G_GAB_PIP3	1	$nM^{-1}\cdot s^{-1}$	31 - 33 (*S1.1*) + [11,13,16]
**522**	ERB3P_G_GAB + PIP3 <-> ERB3P_G_GAB_PIP3	c_b_336r		2.5	s^{-1}	31 - 33 (*S1.1*) + [11,13,16]
**523**	R + ERB3 <-> R_ERB3	c217f	c217f*R*ERB3 - c217r*R_ERB3	0.0001	$nM^{-1}\cdot s^{-1}$	1 - 3 (*S1.1*) + [1-9,13,16]
**524**	R + ERB3 <-> R_ERB3	c217r		0.1	s^{-1}	1 - 3 (*S1.1*) + [1-9,13,16]
**525**	EGF + R_ERB3 <-> Rl_ERB3	c218f	c218f*EGF*R_ERB3 - c218r*Rl_ERB3	0.0001	$nM^{-1}\cdot s^{-1}$	1 - 3 (*S1.1*) + [1-9,13,16]
**526**	EGF + R_ERB3 <-> Rl_ERB3	c218r		0.1	s^{-1}	1 - 3 (*S1.1*) + [1-9,13,16]
**527**	Rl_ERB3 -> RlP_ERB3P	c219f	c219f*Rl_ERB3	1	s^{-1}	1 - 3 (*S1.1*) + [1-9,13,16]
**528**	RlP_ERB3P <-> EGF + RP_ERB3P	c220f	c220f*RlP_ERB3P - c220r*EGF*RP_ERB3P	0.1	s^{-1}	1 - 3 (*S1.1*) + [1-9,13,16]
**529**	RlP_ERB3P <-> EGF + RP_ERB3P	c220r		0.01	$nM^{-1}\cdot s^{-1}$	1 - 3 (*S1.1*) + [1-9,13,16]
**530**	RP_ERB3P <-> RP + ERB3P	c221f	c221f*RP_ERB3P - c221r*RP*ERB3P	0.1	s^{-1}	1 - 3 (*S1.1*) + [1-9,13,16]
**531**	RP_ERB3P <-> RP + ERB3P	c221r		0.01	$nM^{-1}\cdot s^{-1}$	1 - 3 (*S1.1*) + [1-9,13,16]
**532**	Rl + ERB3 <-> Rl_ERB3	c223f	c223f*Rl*ERB3 - c223r*Rl_ERB3	0.001	$nM^{-1}\cdot s^{-1}$	1 - 3 (*S1.1*) + [1-9,13,16]
**533**	Rl + ERB3 <-> Rl_ERB3	c223r		0.1	s^{-1}	1 - 3 (*S1.1*) + [1-9,13,16]
**534**	ERB3P + R <-> R_ERB3P	c224f	c224f*ERB3P*R - c224r*R_ERB3P	0.001	$nM^{-1}\cdot s^{-1}$	1 - 3 (*S1.1*) + [1-9,13,16]

**535**	ERB3P + R <-> R_ERB3P	c224r		0.1	s^{-1}	1 - 3 (*S1.1*) + [1-9,13,16]
**536**	RlP_ERB3P <-> RlP + ERB3P	c226f	c226f*RlP_ERB3P - c227r_n*RlP*ERB3P	0.1	s^{-1}	1 - 3 (*S1.1*) + [1-9,13,16]
**537**	RlP_ERB3P <-> RlP + ERB3P	c227r_n		0.01	$nM^{-1}\cdot s^{-1}$	1 - 3 (*S1.1*) + [1-9,13,16]
**538**	RP + ERB3 <-> RP_ERB3	c227f	c227f*RP*ERB3 - c227r*RP_ERB3	0.001	$nM^{-1}\cdot s^{-1}$	1 - 3 (*S1.1*) + [1-9,13,16]
**539**	RP + ERB3 <-> RP_ERB3	c227r		0.1	s^{-1}	1 - 3 (*S1.1*) + [1-9,13,16]
**540**	RP_ERB3 -> RP_ERB3P	c228f	c228f*RP_ERB3	1	s^{-1}	1 - 3 (*S1.1*) + [1-9,13,16]
**541**	ERB3 + ERB3 <-> ERB3_ERB3	c229f	c229f*ERB3*ERB3 - c229r*ERB3_ERB3	0.0001	$nM^{-1}\cdot s^{-1}$	1 - 3 (*S1.1*) + [1-9,13,16]
**542**	ERB3 + ERB3 <-> ERB3_ERB3	c229r		0.1	s^{-1}	1 - 3 (*S1.1*) + [1-9,13,16]
**543**	ERB3P + ERB <-> ERB3P_ERB	c230f	c230f*ERB3P*ERB - c230r*ERB3P_ERB	0.01	$nM^{-1}\cdot s^{-1}$	1 - 3 (*S1.1*) + [1-9,13,16]
**544**	ERB3P + ERB <-> ERB3P_ERB	c230r		0.1	s^{-1}	1 - 3 (*S1.1*) + [1-9,13,16]
**545**	ERB3P_ERB3P <-> ERB3P + ERB3P	c232f	c232f*ERB3P_ERB3P - c232r*ERB3P*ERB3P	0.1	s^{-1}	1 - 3 (*S1.1*) + [1-9,13,16]
**546**	ERB3P_ERB3P <-> ERB3P + ERB3P	c232r		0.01	$nM^{-1}\cdot s^{-1}$	1 - 3 (*S1.1*) + [1-9,13,16]
**547**	ERB3 + ERB <-> ERB3_ERB	c233f	c233f*ERB3*ERB - c233r*ERB3_ERB	0.001	$nM^{-1}\cdot s^{-1}$	1 - 3 (*S1.1*) + [1-9,13,16]
**548**	ERB3 + ERB <-> ERB3_ERB	c233r		0.1	s^{-1}	1 - 3 (*S1.1*) + [1-9,13,16]
**549**	EGF + ERB3_ERB <-> EGF_ERB3_ERB	c234f	c234f*EGF*ERB3_ERB - c234r*EGF_ERB3_ERB	0.0001	$nM^{-1}\cdot s^{-1}$	1 - 3 (*S1.1*) + [1-9,13,16]
**550**	EGF + ERB3_ERB <-> EGF_ERB3_ERB	c234r		0.1	s^{-1}	1 - 3 (*S1.1*) + [1-9,13,16]
**551**	ERB3 + ERBP <-> ERB3_ERBP	c235f	c235f*ERB3*ERBP - c235r*ERB3_ERBP	0.001	$nM^{-1}\cdot s^{-1}$	1 - 3 (*S1.1*) + [1-9,13,16]
**552**	ERB3 + ERBP <-> ERB3_ERBP	c235r		0.1	s^{-1}	1 - 3 (*S1.1*) + [1-9,13,16]
**553**	ERB3_ERBP -> ERB3P_ERBP	c236f	c236f*ERB3_ERBP	1	s^{-1}	1 - 3 (*S1.1*) + [1-9,13,16]
**554**	ERB3P_ERBP <-> ERB3P + ERBP	c237f	c237f*ERB3P_ERBP - c237r*ERB3P*ERBP	0.1	s^{-1}	1 - 3 (*S1.1*) + [1-9,13,16]
**555**	ERB3P_ERBP <-> ERB3P + ERBP	c237r		0.001	$nM^{-1}\cdot s^{-1}$	1 - 3 (*S1.1*) + [1-9,13,16]
**556**	EGF + ERB3 <-> EGF_ERB3	c238f	c238f*EGF*ERB3 - c238r*EGF_ERB3	0.0001	$nM^{-1}\cdot s^{-1}$	1 - 3 (*S1.1*) + [1-9,13,16]
**557**	EGF + ERB3 <-> EGF_ERB3	c238r		0.1	s^{-1}	1 - 3 (*S1.1*) + [1-9,13,16]
**558**	EGF_ERB3 + R <-> EGF_ERB3_R	c239f	c239f*EGF_ERB3*R - c239r*EGF_ERB3_R	0.0001	$nM^{-1}\cdot s^{-1}$	1 - 3 (*S1.1*) + [1-9,13,16]
**559**	EGF_ERB3 + R <-> EGF_ERB3_R	c239r		0.1	s^{-1}	1 - 3 (*S1.1*) + [1-9,13,16]
**560**	EGF_ERB3 + RP <-> EGF_ERB3_RP	c240f	c240f*EGF_ERB3*RP - c240r*EGF_ERB3_RP	0.0001	$nM^{-1}\cdot s^{-1}$	1 - 3 (*S1.1*) + [1-9,13,16]
**561**	EGF_ERB3 + RP <-> EGF_ERB3_RP	c240r		0.1	s^{-1}	1 - 3 (*S1.1*) + [1-9,13,16]
**562**	EGF_ERB3_RP -> EGF_ERB3P_RP	c241f	c241f*EGF_ERB3_RP	1	s^{-1}	1 - 3 (*S1.1*) + [1-9,13,16]
**563**	EGF_ERB3P_RP <-> EGF + RP_ERB3P	c242f	c242f*EGF_ERB3P_RP - c242r*EGF*RP_ERB3P	0.1	s^{-1}	1 - 3 (*S1.1*) + [1-9,13,16]
**564**	EGF_ERB3P_RP <-> EGF + RP_ERB3P	c242r		0.0001	$nM^{-1}\cdot s^{-1}$	1 - 3 (*S1.1*) + [1-9,13,16]
**565**	EGF_ERB3P_RP <-> EGF_ERB3P + RP	c243f	c243f*EGF_ERB3P_RP - c243r*EGF_ERB3P*RP	0.1	s^{-1}	1 - 3 (*S1.1*) + [1-9,13,16]

**566**	EGF_ERB3P_RP <-> EGF_ERB3P + RP	c243r		0.0001	$nM^{-1}\cdot s^{-1}$	1 - 3 (*S1.1*) + [1-9,13,16]
**567**	EGF_ERB3 + ERBP <-> EGF_ERB3_ERBP	c244f	c244f*EGF_ERB3*ERBP - c244r*EGF_ERB3_ERBP	0.0001	$nM^{-1}\cdot s^{-1}$	1 - 3 (*S1.1*) + [1-9,13,16]
**568**	EGF_ERB3 + ERBP <-> EGF_ERB3_ERBP	c244r		0.1	s^{-1}	1 - 3 (*S1.1*) + [1-9,13,16]
**569**	EGF_ERB3_ERBP -> EGF_ERB3P_ERBP	c245f	c245f*EGF_ERB3_ERBP	1	s^{-1}	1 - 3 (*S1.1*) + [1-9,13,16]
**570**	EGF_ERB3P_ERBP <-> EGF + ERB3P_ERBP	c246f	c246f*EGF_ERB3P_ERBP - c246r*EGF*ERB3P_ERBP	0.1	s^{-1}	1 - 3 (*S1.1*) + [1-9,13,16]
**571**	EGF_ERB3P_ERBP <-> EGF + ERB3P_ERBP	c246r		0.0001	$nM^{-1}\cdot s^{-1}$	1 - 3 (*S1.1*) + [1-9,13,16]
**572**	EGF_ERB3 + ERB <-> EGF_ERB3_ERB	c247f	c247f*EGF_ERB3*ERB - c247r*EGF_ERB3_ERB	0.0001	$nM^{-1}\cdot s^{-1}$	1 - 3 (*S1.1*) + [1-9,13,16]
**573**	EGF_ERB3 + ERB <-> EGF_ERB3_ERB	c247r		0.1	s^{-1}	1 - 3 (*S1.1*) + [1-9,13,16]
**574**	EGF_ERB3P_ERBP <-> EGF_ERB3P + ERBP	c248f	c248f*EGF_ERB3P_ERBP - c248r*EGF_ERB3P*ERBP	0.1	s^{-1}	1 - 3 (*S1.1*) + [1-9,13,16]
**575**	EGF_ERB3P_ERBP <-> EGF_ERB3P + ERBP	c248r		0.0001	$nM^{-1}\cdot s^{-1}$	1 - 3 (*S1.1*) + [1-9,13,16]
**576**	EGF_ERB3 + ERB3 <-> EGF_ERB3_ERB3	c252f	c252f*EGF_ERB3*ERB3 - c252r*EGF_ERB3_ERB3	0.0001	$nM^{-1}\cdot s^{-1}$	1 - 3 (*S1.1*) + [1-9,13,16]
**577**	EGF_ERB3 + ERB3 <-> EGF_ERB3_ERB3	c252r		0.1	s^{-1}	1 - 3 (*S1.1*) + [1-9,13,16]
**578**	EGF + ERB3_ERB3 <-> EGF_ERB3_ERB3	c253f	c253f*EGF*ERB3_ERB3 - c253r*EGF_ERB3_ERB3	0.0001	$nM^{-1}\cdot s^{-1}$	1 - 3 (*S1.1*) + [1-9,13,16]
**579**	EGF + ERB3_ERB3 <-> EGF_ERB3_ERB3	c253r		0.1	s^{-1}	1 - 3 (*S1.1*) + [1-9,13,16]
**580**	CDC25CP + ERB3P <-> CDC25CP_ERB3P	c254f	c254f*CDC25CP*ERB3P - c254r*CDC25CP_ERB3P	0.01	$nM^{-1}\cdot s^{-1}$	26, 27 (*S1.1*) + [12–13,16]
**581**	CDC25CP + ERB3P <-> CDC25CP_ERB3P	c254r		0.1	s^{-1}	26, 27 (*S1.1*) + [12–13,16]
**582**	CDC25CP_ERB3P -> CDC25CP + ERB3	c255f	c255f*CDC25CP_ERB3P	0.5	s^{-1}	26, 27 (*S1.1*) + [12–13,16]
**583**	CDC25CP + ERB3P_ERB3P <-> CDC25CP_ERB3P_ERB3P	c256f	c256f*CDC25CP*ERB3P_ERB3P - c256r*CDC25CP_ERB3P_ERB3P	0.01	$nM^{-1}\cdot s^{-1}$	26, 27 (*S1.1*) + [12–13,16]
**584**	CDC25CP + ERB3P_ERB3P <-> CDC25CP_ERB3P_ERB3P	c256r		0.1	s^{-1}	26, 27 (*S1.1*) + [12–13,16]
**585**	CDC25CP_ERB3P_ERB3P -> CDC25CP + ERB3_ERB3	c257f	c257f*CDC25CP_ERB3P_ERB3P	0.5	s^{-1}	26, 27 (*S1.1*) + [12–13,16]
**586**	ERB3P + ERB3 <-> ERB3P_ERB3	c258f	c258f*ERB3P*ERB3 - c258r*ERB3P_ERB3	0.001	$nM^{-1}\cdot s^{-1}$	1 - 3 (*S1.1*) + [1-9,13,16]
**587**	ERB3P + ERB3 <-> ERB3P_ERB3	c258r		0.1	s^{-1}	1 - 3 (*S1.1*) + [1-9,13,16]
**588**	R_ERB3P + EGF <-> Rl_ERB3P	c259f	c259f*R_ERB3P*EGF - c259r*Rl_ERB3P	0.01	$nM^{-1}\cdot s^{-1}$	1 - 3 (*S1.1*) + [1-9,13,16]
**589**	R_ERB3P + EGF <-> Rl_ERB3P	c259r		0.1	s^{-1}	1 - 3 (*S1.1*) + [1-9,13,16]
**590**	Rl_ERB3P -> RlP_ERB3P	c260f	c260f*Rl_ERB3P	1	s^{-1}	1 - 3 (*S1.1*) + [1-9,13,16]
**591**	RP_ERB3P <-> ERB3P + RP	c261f	c261f*RP_ERB3P - c261r*ERB3P*RP	0.1	s^{-1}	1 - 3 (*S1.1*) + [1-9,13,16]

**592**	RP_ERB3P <-> ERB3P + RP	c261r		0.01	$nM^{-1}\cdot s^{-1}$	1 - 3 (*S1.1*) + [1-9,13,16]
**593**	ERBP_PKP + PhoB <-> ERBP_PKP_PhoB	c262f	c262f*ERBP_PKP*PhoB - c262r*ERBP_PKP_PhoB	0.001	$nM^{-1}\cdot s^{-1}$	1 – 3, 31, 32 (*S1.1*) + [11,13,16]
**594**	ERBP_PKP + PhoB <-> ERBP_PKP_PhoB	c262r	c263f*ERBP_PKP_PhoB	0.038	s^{-1}	1 – 3, 31, 32 (*S1.1*) + [11,13,16]
**595**	ERBP_PKP_PhoB -> ERBP_PK + PhoB	c263f		0.595	s^{-1}	1 – 3, 31, 32 (*S1.1*) + [1,16]
**596**	ERB3P_PKP + PhoB <-> ERB3P_PKP_PhoB	c264f	c264f*ERB3P_PKP*PhoB - c264r*ERB3P_PKP_PhoB	0.001	$nM^{-1}\cdot s^{-1}$	1 – 3, 31, 32 (*S1.1*) + [11,13,16]
**597**	ERB3P_PKP + PhoB <-> ERB3P_PKP_PhoB	c264r		0.038	s^{-1}	1 – 3, 31, 32 (*S1.1*) + [11,13,16]
**598**	ERB3P_PKP_PhoB -> ERB3P_PK + PhoB	c265f	c265f*ERB3P_PKP_PhoB	0.595	s^{-1}	1 – 3, 31, 32 (*S1.1*) + [11,13,16]
**599**	RP_PKP + PhoB <-> RP_PKP_PhoB	c266f	c266f*RP_PKP*PhoB - c266r*RP_PKP_PhoB	0.001	$nM^{-1}\cdot s^{-1}$	1 – 3, 31, 32 (*S1.1*) + [11,13,16]
**600**	RP_PKP + PhoB <-> RP_PKP_PhoB	c266r		0.038	s^{-1}	1 – 3, 31, 32 (*S1.1*) + [11,13,16]
**601**	RP_PKP_PhoB -> RP_PK + PhoB	c267f	c267f*RP_PKP_PhoB	0.595	s^{-1}	1 – 3, 31, 32 (*S1.1*) + [11,13,16]
**602**	ERB_ERB -> ERBP_ERBP	c268f	c268f*ERB_ERB	1	s^{-1}	1 – 3, 31, 32 (*S1.1*) + [11,13,16]
**603**	BetaCatenin + Cadh <-> BetaCatenin_Cadh	c269f	c269f*BetaCatenin*Cadh - c269r*BetaCatenin_Cadh	0.01	$nM^{-1}\cdot s^{-1}$	55, 56 (*S1.1*) + [1]
**604**	BetaCatenin + Cadh <-> BetaCatenin_Cadh	c269r		0.01	s^{-1}	55, 56 (*S1.1*) + [12-13]
**605**	BetaCatenin_Cadh + PTP <-> BetaCatenin_Cadh_PTP	c270f	c270f*BetaCatenin_Cadh*PTP - c270r*BetaCatenin_Cadh_PTP	0.01	$nM^{-1}\cdot s^{-1}$	55, 56 (*S1.1*) + [11,13]
**606**	BetaCatenin_Cadh + PTP <-> BetaCatenin_Cadh_PTP	c270r		1	s^{-1}	55, 56 (*S1.1*) + [11,13]
**607**	Cadh + PTP <-> Cadh_PTP	c271f	c271f*Cadh*PTP - c271r*Cadh_PTP	0.01	$nM^{-1}\cdot s^{-1}$	55, 56 (*S1.1*) + [11,13]
**608**	Cadh + PTP <-> Cadh_PTP	c271r		1	s^{-1}	55, 56 (*S1.1*) + [11,13]
**609**	BetaCatenin_Cadh + RP <-> BetaCatenin_Cadh_RP	c272f	c272f*BetaCatenin_Cadh*RP - c272r*BetaCatenin_Cadh_RP	2.5	$nM^{-1}\cdot s^{-1}$	55, 56 (*S1.1*) + [11,13,16]
**610**	BetaCatenin_Cadh + RP <-> BetaCatenin_Cadh_RP	c272r		1	s^{-1}	55, 56 (*S1.1*) + [11,13,16]
**611**	BetaCatenin_Cadh_RP -> BetaCateninY654 + Cadh + RP	c273f	c273f*BetaCatenin_Cadh_RP	1	s^{-1}	55, 56 (*S1.1*) + [11,13,16]
**612**	Cadh_PTP + BetaCateninY654 <-> BetaCateninY654_Cadh_PTP	c274f	c274f*Cadh_PTP*BetaCateninY654 - c274r*BetaCateninY654_Cadh_PTP	0.01	$nM^{-1}\cdot s^{-1}$	55, 56 (*S1.1*) + [11,13]
**613**	Cadh_PTP + BetaCateninY654 <-> BetaCateninY654_Cadh_PTP	c274r		0.1	s^{-1}	55, 56 (*S1.1*) + [11,13]

**614**	BetaCateninY654_Cadh_PTP -> BetaCatenin + Cadh_PTP	c275f	c275f*BetaCateninY654_Cadh_PTP	1	s^{-1}	55, 56 (*S1.1*) + [11,13]
**615**	BetaCatenin_Cadh_PTP <-> BetaCatenin + Cadh_PTP	c276f	c276f*BetaCatenin_Cadh_PTP - [276r)*BetaCatenin*Cadh_PTP	0.1	s^{-1}	55, 56 (*S1.1*) + [11,13]
**616**	BetaCatenin_Cadh_PTP <-> BetaCatenin + Cadh_PTP	276r		0.01	$nM^{-1}\cdot s^{-1}$	55, 56 (*S1.1*) + [11,13]
**617**	BetaCatenin_Cadh + ERBP <-> BetaCatenin_Cadh_ERBP	c277f	c277f*BetaCatenin_Cadh*ERBP - c277r*BetaCatenin_Cadh_ERBP	2.5	$nM^{-1}\cdot s^{-1}$	55, 56 (*S1.1*) + [11,13,16]
**618**	BetaCatenin_Cadh + ERBP <-> BetaCatenin_Cadh_ERBP	c277r		1	s^{-1}	55, 56 (*S1.1*) + [11,13,16]
**619**	BetaCatenin_Cadh_ERBP -> BetaCateninY654 + Cadh + ERBP	c278f	c278f*BetaCatenin_Cadh_ERBP	1	s^{-1}	55, 56 (*S1.1*) + [11,13,16]
**620**	TGF + TGF <-> DIMTGF	c480f	c480f*TGF*TGF - c480r*DIMTGF	0.01	$nM^{-1}\cdot s^{-1}$	77 - 80 (*S1.1*) + [12-13,17-19]
**621**	TGF + TGF <-> DIMTGF	c480r		0.01	s^{-1}	77 - 80 (*S1.1*) + [12-13,17-19]
**622**	DIMTGF + DIMTBRII <-> DIMTGF_DIMTBRII	C481F	C481F*DIMTGF*DIMTBRII - C481R*DIMTGF_DIMTBRII	0.01	$nM^{-1}\cdot s^{-1}$	77 - 80 (*S1.1*) + [12-13,17-19]
**623**	DIMTGF + DIMTBRII <-> DIMTGF_DIMTBRII	C481R		0.03	s^{-1}	77 - 80 (*S1.1*) + [12-13,17-19]
**624**	DIMTGF_DIMTBRII + DIMTBRI <-> DIMTGF_DIMTBRII_DIMTBRI	C482F	C482F*DIMTGF_DIMTBRII*DIMTBRI - C482R*DIMTGF_DIMTBRII_DIMTBRI	0.01	$nM^{-1}\cdot s^{-1}$	77 - 80 (*S1.1*) + [12-13,17-19]
**625**	DIMTGF_DIMTBRII + DIMTBRI <-> DIMTGF_DIMTBRII_DIMTBRI	C482R		0.01	s^{-1}	77 - 80 (*S1.1*) + [12-13,17-19]
**626**	DIMTGF_DIMTBRII_DIMTBRI -> DIMTGF_DIMTBRII_DIMTBRIP	C483	C483*DIMTGF_DIMTBRII_DIMTBRI	0.1	s^{-1}	77 - 80 (*S1.1*) + [12-13,17-19]
**627**	DIMTGF_DIMTBRII_DIMTBRIP + SMADII <-> DIMTGF_DIMTBRII_DIMTBRIP_SMADII	C486F	C486F*DIMTGF_DIMTBRII_DIMTBRIP*SMADII - C486R*DIMTGF_DIMTBRII_DIMTBRIP_SMADII	0.01	$nM^{-1}\cdot s^{-1}$	77 - 80 (*S1.1*) + [12-13,17-19]
**628**	DIMTGF_DIMTBRII_DIMTBRIP + SMADII <-> DIMTGF_DIMTBRII_DIMTBRIP_SMADII	C486R		10	s^{-1}	77 - 80 (*S1.1*) + [12-13,17-19]
**629**	TBRI + TBRI <-> DIMTBRI	C487F	C487F*TBRI*TBRI - C487R*DIMTBRI	0.01	$nM^{-1}\cdot s^{-1}$	77 - 80 (*S1.1*) + [12-13,17-19]
**630**	TBRI + TBRI <-> DIMTBRI	C487R		0.1	s^{-1}	77 - 80 (*S1.1*) + [12-13,17-19]
**631**	TBRII + TBRII <-> DIMTBRII	C488F	C488F*TBRII*TBRII - C488R*DIMTBRII	0.01	$nM^{-1}\cdot s^{-1}$	77 - 80 (*S1.1*) + [12-13,17-19]
**632**	TBRII + TBRII <-> DIMTBRII	C488R		0.1	s^{-1}	77 - 80 (*S1.1*) + [12-13,17-19]

**633**	DIMTGF_DIMTBRII_DIMTBRIP_SMADII -> DIMTGF_DIMTBRII_DIMTBRIP + SMADIIP	C489	C489*DIMTGF_DIMTBRII_DIMTBRIP_SMADII	1	s^{-1}	77 - 80 (*S1.1*) + [12-13,17-19]
**634**	DIMTGF_DIMTBRII_DIMTBRIP + SMADIII <-> DIMTGF_DIMTBRII_DIMTBRIP_SMADIII	C490F	C490F*DIMTGF_DIMTBRII_DIMTBRIP*SMADIII - C490R*DIMTGF_DIMTBRII_DIMTBRIP_SMADIII	0.01	$nM^{-1}\cdot s^{-1}$	77 - 80 (*S1.1*) + [12-13,17-19]
**635**	DIMTGF_DIMTBRII_DIMTBRIP + SMADIII <-> DIMTGF_DIMTBRII_DIMTBRIP_SMADIII	C490R		10	s^{-1}	77 - 80 (*S1.1*) + [12-13,17-19]
**636**	DIMTGF_DIMTBRII_DIMTBRIP_SMADIII -> DIMTGF_DIMTBRII_DIMTBRIP + SMADIIIP	C491	C491*DIMTGF_DIMTBRII_DIMTBRIP_SMADIII	1	s^{-1}	77 - 80 (*S1.1*) + [12-13], 17 - 19
**637**	DIMTGF_DIMTBRII_DIMTBRIP + TAK <-> DIMTGF_DIMTBRII_DIMTBRIP_TAK	C492F	C492F*DIMTGF_DIMTBRII_DIMTBRIP*TAK - C492R*DIMTGF_DIMTBRII_DIMTBRIP_TAK	0.01	$nM^{-1}\cdot s^{-1}$	82 – 84 (*S1.1*) + [12-13]
**638**	DIMTGF_DIMTBRII_DIMTBRIP + TAK <-> DIMTGF_DIMTBRII_DIMTBRIP_TAK	C492R		0.01	s^{-1}	82 – 84 (*S1.1*) + [12-13]
**639**	DIMTGF_DIMTBRII_DIMTBRIP_TAK -> DIMTGF_DIMTBRII_DIMTBRIP + TAKP	C493	C493*DIMTGF_DIMTBRII_DIMTBRIP_TAK	10	s^{-1}	82 – 84 (*S1.1*) + [12-13]
**640**	NLKP + TCFLEF <-> NLKP_TCFLEF	C496F	C496F*NLKP*TCFLEF - C496R*NLKP_TCFLEF	0.01	$nM^{-1}\cdot s^{-1}$	82 – 84 (*S1.1*) + [12-13]
**641**	NLKP + TCFLEF <-> NLKP_TCFLEF	C496R		1	s^{-1}	82 – 84 (*S1.1*) + [12-13]
**642**	NLKP_TCFLEF -> NLKP + TCFLEFP	C497	C497*NLKP_TCFLEF	1	s^{-1}	82 – 84 (*S1.1*) + [12-13]
**643**	TCFLEFP + Pase8 <-> TCFLEFP_Pase8	C498F	C498F*TCFLEFP*Pase8 - C498R*TCFLEFP_Pase8	0.01	$nM^{-1}\cdot s^{-1}$	82 – 84 (*S1.1*) + [12-13]
**644**	TCFLEFP + Pase8 <-> TCFLEFP_Pase8	C498R		1	s^{-1}	82 – 84 (*S1.1*) + [12-13]
**645**	TCFLEFP_Pase8 -> TCFLEF + Pase8	C499	C499*TCFLEFP_Pase8	0.1	s^{-1}	82 – 84 (*S1.1*) + [12-13]
**646**	TAKP + Pase9 <-> TAKP_Pase9	C500F	C500F*TAKP*Pase9 - C500R*TAKP_Pase9	0.01	$nM^{-1}\cdot s^{-1}$	82 – 84 (*S1.1*) + [12-13]
**647**	TAKP + Pase9 <-> TAKP_Pase9	C500R		1	s^{-1}	82 – 84 (*S1.1*) + [12-13]
**648**	TAKP_Pase9 -> TAK + Pase9	C501	C501*TAKP_Pase9	0.1	s^{-1}	82 – 84 (*S1.1*) + [12-13]
**649**	NLKP + Pase10 <-> NLKP_Pase10	C502F	C502F*NLKP*Pase10 - C502R*NLKP_Pase10	0.01	$nM^{-1}\cdot s^{-1}$	82 – 84 (*S1.1*) + [12-13]
**650**	NLKP + Pase10 <-> NLKP_Pase10	C502R		1	s^{-1}	82 – 84 (*S1.1*) + [12-13]
**651**	NLKP_Pase10 -> NLK + Pase10	C503	C503*NLKP_Pase10	0.1	s^{-1}	82 – 84 (*S1.1*) + [12-13]
**652**	SMADIIP + SMAD4 <-> SMADIIP_SMAD4	C504F	C504F*SMADIIP*SMAD4 - C504R*SMADIIP_SMAD4	0.01	$nM^{-1}\cdot s^{-1}$	77 - 80 (*S1.1*) + [12-13,17-19]
**653**	SMADIIP + SMAD4 <-> SMADIIP_SMAD4	C504R		0.01	s^{-1}	77 - 80 (*S1.1*) + [12-13,17-19]
**654**	SMADIIIP + SMAD4 <-> SMADIIIP_SMAD4	C505F	C505F*SMADIIIP*SMAD4 - C505R*SMADIIIP_SMAD4	0.01	$nM^{-1}\cdot s^{-1}$	77 - 80 (*S1.1*) + [12-13,17-19]
**655**	SMADIIIP + SMAD4 <-> SMADIIIP_SMAD4	C505R		0.01	s^{-1}	77 - 80 (*S1.1*) + [12-13,17-19]

**656**	TCFLEF + BetaCatenin <-> TCFLEF_BetaCatenin	C510F	C510F*TCFLEF*BetaCatenin - C510R*TCFLEF_BetaCatenin	0.01	$nM^{-1}\cdot s^{-1}$	60 (*S1.1*) + [12-13]
**657**	TCFLEF + BetaCatenin <-> TCFLEF_BetaCatenin	C510R		10	s^{-1}	60 (*S1.1*) + [12-13]
**658**	SMADIIIP + PP1A <-> SMADIIIP_PP1A	C511F	C511F*SMADIIIP*PP1A - C511R*SMADIIIP_PP1A	0.01	$nM^{-1}\cdot s^{-1}$	91 (*S1.1*) + [12-13,17-19]
**659**	SMADIIIP + PP1A <-> SMADIIIP_PP1A	C511R		10	s^{-1}	91 (*S1.1*) + [12-13,17-19]
**660**	SMADIIIP_PP1A -> SMADIII + PP1A	C512	C512*SMADIIIP_PP1A	0.1	s^{-1}	91 (*S1.1*) + [12-13,17-19]
**661**	SMADIIP + PP1A <-> SMADIIP_PP1A	C513F	C513F*SMADIIP*PP1A - C514R*SMADIIP_PP1A	0.01	$nM^{-1}\cdot s^{-1}$	91 (*S1.1*) + [12-13,17-19]
**662**	SMADIIP + PP1A <-> SMADIIP_PP1A	C514R	C514*SMADIIP_PP1A	10	s^{-1}	91 (*S1.1*) + [12-13,17-19]
**663**	SMADIIP_PP1A -> SMADII + PP1A	C514		0.1	s^{-1}	91 (*S1.1*) + [12-13,17-19]
**664**	TCFLEF + BetaCateninY654 <-> TCFLEF_BetaCateninY654	C515F	C515F*TCFLEF*BetaCateninY654 - C515R*TCFLEF_BetaCateninY654	0.01	$nM^{-1}\cdot s^{-1}$	55 - 65 (*S1.1*) + [12-13]
**665**	TCFLEF + BetaCateninY654 <-> TCFLEF_BetaCateninY654	C515R		10	s^{-1}	55 - 65 (*S1.1*) + [12-13]
**666**	TCFLEF_BetaCateninY654 + TFBS <-> TCFLEF_BetaCateninY654_TFBS	C516F	C516F*TCFLEF_BetaCateninY654*TFBS - C516R*TCFLEF_BetaCateninY654_TFBS	0.01	$nM^{-1}\cdot s^{-1}$	55 - 65 (*S1.1*) + [12-13]
**667**	TCFLEF_BetaCateninY654 + TFBS <-> TCFLEF_BetaCateninY654_TFBS	C516R		0.01	s^{-1}	55 - 65 (*S1.1*) + [12-13]
**668**	TCFLEF_BetaCatenin + TFBS <-> TCFLEF_BetaCatenin_TFBS	C517F	C517F*TCFLEF_BetaCatenin*TFBS - C517R*TCFLEF_BetaCatenin_TFBS	0.01	$nM^{-1}\cdot s^{-1}$	55 - 65 (*S1.1*) + [12-13]
**669**	TCFLEF_BetaCatenin + TFBS <-> TCFLEF_BetaCatenin_TFBS	C517R		0.02	s^{-1}	55 - 65 (*S1.1*) + [12-13]
**670**	DIMTGF_DIMTBRII + DIMTBRIP <-> DIMTGF_DIMTBRII_DIMTBRIP	C518F	C518F*DIMTGF_DIMTBRII*DIMTBRIP - C518R*DIMTGF_DIMTBRII_DIMTBRIP	0.01	$nM^{-1}\cdot s^{-1}$	77 - 80 (*S1.1*) + [12-13]
**671**	DIMTGF_DIMTBRII + DIMTBRIP <-> DIMTGF_DIMTBRII_DIMTBRIP	C518R		0.1	s^{-1}	77 - 80 (*S1.1*) + [12-13]
**672**	DIMTBRIP + PP1C <-> DIMTBRIP_PP1C	C519F	C519F*DIMTBRIP*PP1C - C519R*DIMTBRIP_PP1C	0.01	$nM^{-1}\cdot s^{-1}$	81 (*S1.1*) + [12-13]
**673**	DIMTBRIP + PP1C <-> DIMTBRIP_PP1C	C519R		1	s^{-1}	81 (*S1.1*) + [12-13]
**674**	DIMTBRIP_PP1C -> DIMTBRI + PP1C	C520	C520*DIMTBRIP_PP1C	0.5	s^{-1}	81 (*S1.1*) + [12-13]
**675**	SMADIIP_SMAD4 + TFBSI <-> SMADIIP_SMAD4_TFBSI	C521F	C521F*SMADIIP_SMAD4*TFBSI - C521R*SMADIIP_SMAD4_TFBSI	0.01	$nM^{-1}\cdot s^{-1}$	85 - 90 (*S1.1*) + [12-13]
**676**	SMADIIP_SMAD4 + TFBSI <-> SMADIIP_SMAD4_TFBSI	C521R		0.1	s^{-1}	85 - 90 (*S1.1*) + [12-13]
**677**	SMADIIIP_SMAD4 + TFBSI <-> SMADIIIP_SMAD4_TFBSI	C522F	C522F*SMADIIIP_SMAD4*TFBSI - C522R*SMADIIIP_SMAD4_TFBSI	0.01	$nM^{-1}\cdot s^{-1}$	85 - 90 (*S1.1*) + [12-13]
**678**	SMADIIIP_SMAD4 + TFBSI <-> SMADIIIP_SMAD4_TFBSI	C522R		0.1	s^{-1}	85 - 90 (*S1.1*) + [12-13]

**679**	Ras_GTP + PI3K <-> Ras_GTP_PK	C523F	C523F*Ras_GTP*PI3K - C523R*Ras_GTP_PK	0.001	$nM^{-1}\cdot s^{-1}$	34 (*S1.1*) + [12-13]
**680**	Ras_GTP + PI3K <-> Ras_GTP_PK	C523R		15	s^{-1}	34 (*S1.1*) + [12-13]
**681**	Ras_GTP_PK -> Ras_GTP_PKP	C524	C524*Ras_GTP_PK	0.1	s^{-1}	34 (*S1.1*) + [12-13]
**682**	Ras_GTP_PKP <-> Ras_GTP + PKP	C525R	C525R*Ras_GTP_PKP - C525F*Ras_GTP*PKP	15	s^{-1}	34 (*S1.1*) + [12-13]
**683**	Ras_GTP_PKP <-> Ras_GTP + PKP	C525F		0.001	$nM^{-1}\cdot s^{-1}$	34 (*S1.1*) + [12-13]
**684**	Ras_GTP_PKP + PIP2 <-> Ras_GTP_PKP_PIP2	C526F	C526F*Ras_GTP_PKP*PIP2 - C526R*Ras_GTP_PKP_PIP2	0.625	$nM^{-1}\cdot s^{-1}$	31, 32, 34 (*S1.1*) + [12-13]
**685**	Ras_GTP_PKP + PIP2 <-> Ras_GTP_PKP_PIP2	C526R		10	s^{-1}	31, 32, 34 (*S1.1*) + [12-13]
**686**	Ras_GTP_PKP + PhoB <-> Ras_GTP_PKP_PhoB	C528F	C528F*Ras_GTP_PKP*PhoB - C528R*Ras_GTP_PKP_PhoB	0.01	$nM^{-1}\cdot s^{-1}$	31, 32, 34 (*S1.1*) + [12-13]
**687**	Ras_GTP_PKP + PhoB <-> Ras_GTP_PKP_PhoB	C528R		1	s^{-1}	31, 32, 34 (*S1.1*) + [12-13]
**688**	Ras_GTP_PKP_PhoB -> Ras_GTP + PI3K + PhoB	C528	C528*Ras_GTP_PKP_PhoB	0.5	s^{-1}	31, 32, 34 (*S1.1*) + [12-13]
**689**	Ras_GTP_PKP_PIP2 -> Ras_GTP_PKP + PIP3	C527	C527*Ras_GTP_PKP_PIP2	2.5	s^{-1}	31, 32, 34 (*S1.1*) + [12-13]
**690**	ERKPP + AP1 <-> ERKPP_AP1	c528f	c528f*ERKPP*AP1 - c528r*ERKPP_AP1	0.01	$nM^{-1}\cdot s^{-1}$	15 - 23 (*S1.1*) + [12-13]
**691**	ERKPP + AP1 <-> ERKPP_AP1	c528r		10	s^{-1}	15 - 23 (*S1.1*) + [12-13]
**692**	ERKPP_AP1 -> ERKPP + AP1P	c529	c529*ERKPP_AP1	1	s^{-1}	15 - 23 (*S1.1*) + [12-13]
**693**	AP1P + Pase12 <-> AP1P_Pase12	c530f	c530f*AP1P*Pase12 - c530r*AP1P_Pase12	0.01	$nM^{-1}\cdot s^{-1}$	15 - 23 (*S1.1*) + [12-13]
**694**	AP1P + Pase12 <-> AP1P_Pase12	c530r		0.1	s^{-1}	15 - 23 (*S1.1*) + [12-13]
**695**	AP1P_Pase12 -> AP1 + Pase12	c531	c531*AP1P_Pase12	1	s^{-1}	15 - 23 (*S1.1*) + [12-13]
**696**	AP1 + TFBSII <-> AP1_TFBSII	c532f	c532f*AP1*TFBSII - c532r*AP1_TFBSII	0.01	$nM^{-1}\cdot s^{-1}$	15 - 23 (*S1.1*) + [12-13]
**697**	AP1 + TFBSII <-> AP1_TFBSII	c532r		7	s^{-1}	15 - 23 (*S1.1*) + [12-13]
**698**	AP1P + TFBSII <-> AP1P_TFBSII	c533f	c533f*AP1P*TFBSII - c533r*AP1P_TFBSII	0.01	$nM^{-1}\cdot s^{-1}$	15 - 23 (*S1.1*) + [12-13]
**699**	AP1P + TFBSII <-> AP1P_TFBSII	c533r		0.35	s^{-1}	15 - 23 (*S1.1*) + [12-13]
**700**	WNT + FRZ <-> WNT_FRZ	c534f	c534f*WNT*FRZ - c534r*WNT_FRZ	0.01	$nM^{-1}\cdot s^{-1}$	49, 70 - 75 (*S1.1*) + [12-13]
**701**	WNT + FRZ <-> WNT_FRZ	c534r		0.04	s^{-1}	49, 70 - 75 (*S1.1*) + [12-13]
**702**	WNT_FRZ + LRP6 <-> WNT_FRZ_LRP6	c535f	c535f*WNT_FRZ*LRP6 - c535r*WNT_FRZ_LRP6	0.01	$nM^{-1}\cdot s^{-1}$	49, 70 - 75 (*S1.1*) + [12-13]
**703**	WNT_FRZ + LRP6 <-> WNT_FRZ_LRP6	c535r		0.04	s^{-1}	49, 70 - 75 (*S1.1*) + [12-13]
**704**	WNT_FRZ_LRP6 + DVL <-> WNT_FRZ_LRP6_DVL	c536f	c536f*WNT_FRZ_LRP6*DVL - c536r*WNT_FRZ_LRP6_DVL	0.01	$nM^{-1}\cdot s^{-1}$	49, 70 - 75 (*S1.1*) + [12-13]

**705**	WNT_FRZ_LRP6 + DVL <-> WNT_FRZ_LRP6_DVL	c536r		0.04	s^{-1}	49, 70 - 75 (*S1.1*) + [12-13]
**706**	AXN + GSK <-> AXN_GSK	c537f	c537f*AXN*GSK - c537r*AXN_GSK	0.01	$nM^{-1}\cdot s^{-1}$	49, 70 - 75 (*S1.1*) + [12-13,20]
**707**	AXN + GSK <-> AXN_GSK	c537r		0.1	s^{-1}	48 - 55 (*S1.1*) + [12-13,20]
**708**	AXN_GSK -> AXNP_GSK	c538	c538*AXN_GSK	1	s^{-1}	48 - 55 (*S1.1*) + [12-13,20]
**709**	AXNP_GSK + APC <-> AXNP_GSK_APC	c539f	c539f*AXNP_GSK*APC - c539r*AXNP_GSK_APC	0.01	$nM^{-1}\cdot s^{-1}$	48 - 55 (*S1.1*) + [12-13,20]
**710**	AXNP_GSK + APC <-> AXNP_GSK_APC	c539r		0.1	s^{-1}	48 - 55 (*S1.1*) + [12-13,20]
**711**	AXNP_GSK_APC -> AXNP_GSK_APCP	c540	c540*AXNP_GSK_APC	1	s^{-1}	48 - 55 (*S1.1*) + [12-13,20]
**712**	WNT_FRZ_LRP6_DVL + GSK <-> WNT_FRZ_LRP6_DVL_GSK	c541f		0.01	$nM^{-1}\cdot s^{-1}$	49, 70 - 75 (*S1.1*) + [12-13]
**713**	WNT_FRZ_LRP6_DVL + GSK <-> WNT_FRZ_LRP6_DVL_GSK	c541r	c541f*WNT_FRZ_LRP6_DVL*GSK - c541r*WNT_FRZ_LRP6_DVL_GSK	0.04	s^{-1}	49, 70 - 75 (*S1.1*) + [12-13]
**714**	WNT_FRZ_LRP6_DVL_GSK -> WNT_FRZ_LRP6P_DVL_GSK	c542	c542*WNT_FRZ_LRP6_DVL_GSK	1	s^{-1}	49, 70 - 75 (*S1.1*) + [12-13]
**715**	WNT_FRZ_LRP6P_DVL_GSK + AXN <-> WNT_FRZ_LRP6P_DVL_GSK_AXN	c543f	c543f*WNT_FRZ_LRP6P_DVL_GSK*AXN - c543r*WNT_FRZ_LRP6P_DVL_GSK_AXN	0.01	v	49, 70 - 75 (*S1.1*) + [12-13]
**716**	WNT_FRZ_LRP6P_DVL_GSK + AXN <-> WNT_FRZ_LRP6P_DVL_GSK_AXN	c543r		0.04	s^{-1}	49, 70 - 75 (*S1.1*) + [12-13]
**717**	AXNP_GSK_APCP + BetaCatenin <-> AXNP_GSK_APCP_BetaCatenin	c544f	c544f*AXNP_GSK_APCP*BetaCatenin - c544r*AXNP_GSK_APCP_BetaCatenin	0.01	$nM^{-1}\cdot s^{-1}$	48 - 55 (*S1.1*) + [12-13,20]
**718**	AXNP_GSK_APCP + BetaCatenin <-> AXNP_GSK_APCP_BetaCatenin	c544r		0.1	s^{-1}	48 - 55 (*S1.1*) + [12-13,20]
**719**	AXNP_GSK_APCP_BetaCatenin -> AXNP_GSK_APCP + BetaCateninP	c545	c545*AXNP_GSK_APCP_BetaCatenin	1	s^{-1}	48 - 55 (*S1.1*) + [12-13,20]
**720**	BetaCateninP -> BetaCateninU	c546	c546*BetaCateninP	1	s^{-1}	49 , 53 (*S1.1*) + [20,21]
**721**	BetaCatenin_generator -> BetaCatenin	c547	c547*BetaCatenin_generator	0.05	s^{-1}	49 , 53 (*S1.1*) + [20,21]
**722**	WNT_FRZ_LRP6P_DVL_GSK + PhoE <-> WNT_FRZ_LRP6P_DVL_GSK_PhoE	c548f	c548f*WNT_FRZ_LRP6P_DVL_GSK*PhoE - c548r*WNT_FRZ_LRP6P_DVL_GSK_PhoE	0.01	$nM^{-1}\cdot s^{-1}$	49, 70 - 75 (*S1.1*) + [12-13]
**723**	WNT_FRZ_LRP6P_DVL_GSK + PhoE <-> WNT_FRZ_LRP6P_DVL_GSK_PhoE	c548r		0.1	s^{-1}	49, 70 - 75 (*S1.1*) + [12-13]
**724**	WNT_FRZ_LRP6P_DVL_GSK_PhoE -> WNT_FRZ_LRP6_DVL + GSK + PhoE	c549	c549*WNT_FRZ_LRP6P_DVL_GSK_PhoE	0.5	s^{-1}	49, 70 - 75 (*S1.1*) + [12-13]
**725**	AXNP_GSK + PhoE <-> AXNP_GSK_PhoE	c550f	c550f*AXNP_GSK*PhoE - c550r*AXNP_GSK_PhoE	0.01	$nM^{-1}\cdot s^{-1}$	48 - 55 (*S1.1*) + [12-13,20]
**726**	AXNP_GSK + PhoE <-> AXNP_GSK_PhoE	c550r		0.1	s^{-1}	48 - 55 (*S1.1*) + [12-13,20]
**727**	AXNP_GSK_PhoE -> AXN + GSK + PhoE	c551	c551*AXNP_GSK_PhoE	0.5	s^{-1}	48 - 55 (*S1.1*) + [12-13,20]

**728**	BetaCateninP + PhoE <-> BetaCateninP_PhoE	c552f	c552f*BetaCateninP*PhoE - c552r*BetaCateninP_PhoE	0.01	$nM^{-1}\cdot s^{-1}$	48 - 55 (*S1.1*) + [12-13,20]
**729**	BetaCateninP + PhoE <-> BetaCateninP_PhoE	c552r		0.1	s^{-1}	48 - 55 (*S1.1*) + [12-13,20]
**730**	BetaCateninP_PhoE -> BetaCatenin + PhoE	c553	c553*BetaCateninP_PhoE	1	s^{-1}	48 - 55 (*S1.1*) + [12-13,20]
**731**	AXNP_GSK <-> AXNP + GSK	c554r	c554r*AXNP_GSK - c554f*AXNP*GSK	0.1	s^{-1}	48 - 55 (*S1.1*) + [12-13,20]
**732**	AXNP_GSK <-> AXNP + GSK	c554f		0.01	$nM^{-1}\cdot s^{-1}$	48 - 55 (*S1.1*) + [12-13,20]
**733**	AXNP + PhoE <-> AXNP_PhoE	c555f	c555f*AXNP*PhoE - c555r*AXNP_PhoE	0.01	$nM^{-1}\cdot s^{-1}$	48 - 55 (*S1.1*) + [12-13,20]
**734**	AXNP + PhoE <-> AXNP_PhoE	c555r		0.1	s^{-1}	48 - 55 (*S1.1*) + [12-13,20]
**735**	AXNP_PhoE -> AXN + PhoE	c556	c556*AXNP_PhoE	1	s^{-1}	48 - 55 (*S1.1*) + [12-13,20]
**736**	AXNP_GSK_APCP <-> AXNP_GSK + APCP	c557r	c557r*AXNP_GSK_APCP - c557f*AXNP_GSK*APCP	0.1	s^{-1}	48 - 55 (*S1.1*) + [12-13,20]
**737**	AXNP_GSK_APCP <-> AXNP_GSK + APCP	c557f		0.01	$nM^{-1}\cdot s^{-1}$	48 - 55 (*S1.1*) + [12-13,20]
**738**	APCP + PhoE <-> APCP_PhoE	c558f	c558f*APCP*PhoE - c558r*APCP_PhoE	0.01	$nM^{-1}\cdot s^{-1}$	48 - 55 (*S1.1*) + [12-13,20]
**739**	APCP + PhoE <-> APCP_PhoE	c558r		0.1	s^{-1}	48 - 55 (*S1.1*) + [12-13,20]
**740**	APCP_PhoE -> APC + PhoE	c559	c559*APCP_PhoE	1	s^{-1}	48 - 55 (*S1.1*) + [12-13,20]
**741**	PIP2_Gen -> PIP2	c560	c560*PIP2_Gen	10	s^{-1}	32, 37 (*S1.1*) + [12-13,21]
**742**	PIP2 + PLCyP <-> PIP2_PLCyP	c561f	c561f*PIP2*PLCyP - c561r*PIP2_PLCyP	0.01	$nM^{-1}\cdot s^{-1}$	28, 29 (*S1.1*) + [12-13]
**743**	PIP2 + PLCyP <-> PIP2_PLCyP	c561r		10	s^{-1}	28, 29 (*S1.1*) + [12-13]
**744**	PIP2_PLCyP -> IP3 + DAG + PLCyP	c562	c562*PIP2_PLCyP	0.001	s^{-1}	28, 29 (*S1.1*) + [12-13]
**745**	DAG + PKC <-> DAG_PKC	c563f	c563f*DAG*PKC - c563r*DAG_PKC	0.01	$nM^{-1}\cdot s^{-1}$	28, 29 (*S1.1*) + [12-13],
**746**	DAG + PKC <-> DAG_PKC	c563r		0.1	s^{-1}	28, 29 (*S1.1*) + [12-13],
**747**	IP3 -> IP3D	c565	c565*IP3	0.00001	s^{-1}	28, 29 (*S1.1*) + [12-13,21]
**748**	DAG -> DAGD	c566	c566*DAG	0.00001	s^{-1}	28, 29 (*S1.1*) + [12-13,21]
**749**	DAG_PKC + Raf <-> DAG_PKC_Raf	c567f	c567f*DAG_PKC*Raf - c567r*DAG_PKC_Raf	0.01	$nM^{-1}\cdot s^{-1}$	30 (*S1.1*) + [12-13]
**750**	DAG_PKC + Raf <-> DAG_PKC_Raf	c567r		0.1	s^{-1}	30 (*S1.1*) + [12-13]
**751**	DAG_PKC_Raf -> DAG + PKC + [Raf)	c568	c568*DAG_PKC_Raf	10	s^{-1}	30 (*S1.1*) + [12-13]
**752**	WNT_FRZ_LRP6 + TAK <-> WNT_FRZ_LRP6_TAK	c569f	c569f*WNT_FRZ_LRP6*TAK - c569r*WNT_FRZ_LRP6_TAK	0.01	$nM^{-1}\cdot s^{-1}$	76, 83, 84 (*S1.1*) + [12-13]
**753**	WNT_FRZ_LRP6 + TAK <-> WNT_FRZ_LRP6_TAK	c569r		10	s^{-1}	76, 83, 84 (*S1.1*) + [12-13]
**754**	WNT_FRZ_LRP6_TAK -> WNT_FRZ_LRP6 + TAKP	c570	c570*WNT_FRZ_LRP6_TAK	0.1	s^{-1}	76, 83, 84 (*S1.1*) + [12-13]
**755**	TAKP + TAB <-> TAKP_TAB	c571f	c571f*TAKP*TAB - c571r*TAKP_TAB	0.01	$nM^{-1}\cdot s^{-1}$	76, 83, 84 (*S1.1*) + [12-13]

**756**	TAKP + TAB <-> TAKP_TAB	c571r		10	s^{-1}	76, 83, 84 (*S1.1*) + [12-13]
**757**	TAKP_TAB + NLK <-> TAKP_TAB_NLK	c572f	c572f*TAKP_TAB*NLK - c572r*TAKP_TAB_NLK	0.01	$nM^{-1}\cdot s^{-1}$	76, 83, 84 (*S1.1*) + [12-13]
**758**	TAKP_TAB + NLK <-> TAKP_TAB_NLK	c572r		10	s^{-1}	76, 83, 84 (*S1.1*) + [12-13]
**759**	TAKP_TAB_NLK -> TAKP_TAB + NLKP	c573	c573*TAKP_TAB_NLK	1	s^{-1}	76, 83, 84 (*S1.1*) + [12-13]
**760**	GROUCHO + TCFLEF <-> GROUCHO_TCFLEF	c578f	c578f*GROUCHO*TCFLEF - c578r*GROUCHO_TCFLEF	0.01	$nM^{-1}\cdot s^{-1}$	68, 69 (*S1.1*) + [12-13]
**761**	GROUCHO + TCFLEF <-> GROUCHO_TCFLEF	c578r		10	s^{-1}	68, 69 (*S1.1*) + [12-13]
**762**	GROUCHO_TCFLEF + TFBS <-> GROUCHO_TCFLEF_TFBS	c579f	c579f*GROUCHO_TCFLEF*TFBS - c579r*GROUCHO_TCFLEF_TFBS	0.01	$nM^{-1}\cdot s^{-1}$	68, 69 (*S1.1*) + [12-13]
**763**	GROUCHO_TCFLEF + TFBS <-> GROUCHO_TCFLEF_TFBS	c579r		1	s^{-1}	68, 69 (*S1.1*) + [12-13]
**764**	APC + BetaCatenin <-> APC_BetaCatenin	c581f	c581f*APC*BetaCatenin - c581r*APC_BetaCatenin	0.01	$nM^{-1}\cdot s^{-1}$	48 - 55 (*S1.1*) + [12-13,20]
**765**	APC + BetaCatenin <-> APC_BetaCatenin	c581r		12	s^{-1}	48 - 55 (*S1.1*) + [12-13,20]
**766**	SMAD4 + TCFLEF <-> SMAD4_TCFLEF	c582f	c582f*SMAD4*TCFLEF - c582r*SMAD4_TCFLEF	0.01	$nM^{-1}\cdot s^{-1}$	69 (*S1.1*) + [13,14]
**767**	SMAD4 + TCFLEF <-> SMAD4_TCFLEF	c582r		1	s^{-1}	69(*S1.1*) + [13,14]
**768**	SMAD4_TCFLEF + TFBS <-> SMAD4_TCFLEF_TFBS	c583f	c583f*SMAD4_TCFLEF*TFBS - c583r*SMAD4_TCFLEF_TFBS	0.01	$nM^{-1}\cdot s^{-1}$	69 (*S1.1*) + [13,14]
**769**	SMAD4_TCFLEF + TFBS <-> SMAD4_TCFLEF_TFBS	c583r		1	s^{-1}	69 (*S1.1*) + [13,14]
**770**	PIP2 -> PIP2deg	c584	c584*PIP2	0.0004	s^{-1}	32, 37 (*S1.1*) + [12-13,21]
**771**	[Raf) + Raf_inhibitor <-> [Raf_Raf_inhibitor)	c585f	c585f*[Raf*)*Raf_inhibitor - c585r*[Raf*_Raf_inhibitor)	0.01	$nM^{-1}\cdot s^{-1}$	[23]
**772**	[Raf) + Raf_inhibitor <-> [Raf_Raf_inhibitor)	c585r		0.001	s^{-1}	[23]
**773**	PKP + PI3K_inhibitor <-> PKP_PI3K_inhibitor	c586f	c586f*PKP*PI3K_inhibitor - c586r*PKP_PI3K_inhibitor	0.01	$nM^{-1}\cdot s^{-1}$	[23]
**774**	PKP + PI3K_inhibitor <-> PKP_PI3K_inhibitor	c586r		1.5	s^{-1}	[23]
**775**	BetaCatenin -> BetaCateninDeg	c587	c587*BetaCatenin	0.0000001	s^{-1}	49, 53 (*S1.1*) + [20,21]
**776**	RP + PanERB <-> RP_PanERB	c588f	c588f*RP*PanERB - c588r*RP_PanERB	0.01	$nM^{-1}\cdot s^{-1}$	[23]
**777**	RP + PanERB <-> RP_PanERB	c588r		0.00001	s^{-1}	[23]
**778**	ERBP + PanERB <-> ERBP_PanERB	c589f	c589f*ERBP*PanERB - c589r*ERBP_PanERB	0.01	$nM^{-1}\cdot s^{-1}$	[23]
**779**	ERBP + PanERB <-> ERBP_PanERB	c589r		0.00001	s^{-1}	[23]
**780**	ERB3P + PanERB <-> ERB3P_PanERB	c590f	c590f*ERB3P*PanERB - c590r*ERB3P_PanERB	0.01	$nM^{-1}\cdot s^{-}$	[23]
**781**	ERB3P + PanERB <-> ERB3P_PanERB	c590r		0.00001	s^{-1}	[23]

**782**	PIP3 -> PIP3Deg	c591	c591*PIP3	0.0001	s^{-1}	[23]
**783**	AKT + AKT_inhibi <-> AKT_AKT_inhibi	c592f	c592f*AKT*AKT_inhibi - c592r*AKT_AKT_inhibi	0.01	$nM^{-1}\cdot s^{-1}$	[23]
**784**	AKT + AKT_inhibi <-> AKT_AKT_inhibi	c592r		48	s^{-1}	[23]
**785**	AXNP_GSK + BetaCatenin <-> AXNP_GSK_BetaCatenin	c593f	c593f*AXNP_GSK*BetaCatenin - c593r*AXNP_GSK_BetaCatenin	0.01	$nM^{-1}\cdot s^{-1}$	48 - 55 (*S1.1*) + [12-13,20]
**786**	AXNP_GSK + BetaCatenin <-> AXNP_GSK_BetaCatenin	c593r		10000	s^{-1}	48 - 55 (*S1.1*) + [12-13,20]
**787**	AXNP_GSK_BetaCatenin -> AXNP_GSK + BetaCateninP	c594	c594*AXNP_GSK_BetaCatenin	1	s^{-1}	48 - 55 (*S1.1*) + [12-13,20]
**788**	MEKPP + MEK_inhi <-> MEKPP_MEK_inhi	c595f	c595f*MEKPP*MEK_inhi - c595r*MEKPP_MEK_inhi	0.01	$nM^{-1}\cdot s^{-1}$	[23]
**789**	MEKPP + MEK_inhi <-> MEKPP_MEK_inhi	c595r		3	s^{-1}	[23]
**790**	G_S + ERKPP <-> G_S_ERKPP	c596f	c596f*G_S*ERKPP - c596r*G_S_ERKPP	0.01	$nM^{-1}\cdot s^{-1}$	11, 24, 25 (*S1.1*) + [12-13]
**791**	G_S + ERKPP <-> G_S_ERKPP	c596r		0.033	s^{-1}	11, 24, 25 (*S1.1*) + [12-13]
**792**	G_S_ERKPP -> Grb + SOSP + ERKPP	c597	c597*G_S_ERKPP	1	s^{-1}	11, 24, 25 (*S1.1*) + [12-13]
**793**	AKTP + MDM2 <-> AKTP_MDM2	c598f	c598f*AKTP*MDM2 - c598r*AKTP_MDM2	0.01	$nM^{-1}\cdot s^{-1}$	40 (*S1.1*) + [12-13]
**794**	AKTP + MDM2 <-> AKTP_MDM2	c598r		0.1	s^{-1}	40 (*S1.1*) + [12-13]
**795**	AKTP_MDM2 -> AKTP + MDM2P	c599	c599*AKTP_MDM2	0.1	s^{-1}	40 (*S1.1*) + [12-13]
**796**	MDM2P + TP53 <-> MDM2P_TP53	c600f	c600f*MDM2P*TP53 - c600r*MDM2P_TP53	0.01	$nM^{-1}\cdot s^{-1}$	40, 41 (*S1.1*) + [12-13]
**797**	MDM2P + TP53 <-> MDM2P_TP53	c600r		0.1	s^{-1}	40, 41 (*S1.1*) + [12-13]
**798**	MDM2P_TP53 -> MDM2P + TP53U	c601	c601*MDM2P_TP53	1	s^{-1}	40, 41 (*S1.1*) + [12-13]
**799**	TP53gen -> TP53	c602	c602*TP53gen	0.05	s^{-1}	(12,13,21)
**800**	MDM2P + Pho13 <-> MDM2P_Pho13	c603f	c603f*MDM2P*Pho13 - c603r*MDM2P_Pho13	0.01	$nM^{-1}\cdot s^{-1}$	40 (*S1.1*) + [12-13]
**801**	MDM2P + Pho13 <-> MDM2P_Pho13	c603r		0.1	s^{-1}	40 (*S1.1*) + [12-13]
**802**	MDM2P_Pho13 -> MDM2 + Pho13	c604	c604*MDM2P_Pho13	1	s^{-1}	40 (*S1.1*) + [12-13]
**803**	TP53 + TFBSIV <-> TP53_TFBSIV	c605f	c605f*TP53*TFBSIV - c605r*TP53_TFBSIV	0.01	$nM^{-1}\cdot s^{-}$	40 -4 3 (*S1.1*) + [12-13]
**804**	TP53 + TFBSIV <-> TP53_TFBSIV	c605r		0.1	s^{-1}	40 - 43 (*S1.1*) + [12-13]
**805**	RP_ShP_G_GABP + ERKPP <-> RP_ShP_G_GABP_ERKPP	c606f	c606f*RP_ShP_G_GABP*ERKPP - c606r*RP_ShP_G_GABP_ERKPP	0.01	$nM^{-1}\cdot s^{-1}$	33, 35, 36 (*S1.1*) + [4,12-13,16]
**806**	RP_ShP_G_GABP + ERKPP <-> RP_ShP_G_GABP_ERKPP	c606r		0.1	s^{-1}	33, 35, 36 (*S1.1*) + [4,12-13,16]
**807**	RP_ShP_G_GABP_ERKPP -> RP_ShP_G + GABPP + ERKPP	c607	c607*RP_ShP_G_GABP_ERKPP	1	s^{-1}	33, 35, 36 (*S1.1*) + [4,12-13,16]

**808**	RP_G_GABP + ERKPP <-> RP_G_GABP_ERKPP	c608f	c608f*RP_G_GABP*ERKPP - c608r*RP_G_GABP_ERKPP	0.01	$nM^{-1}\cdot s^{-1}$	33, 35, 36 (*S1.1*) + [4,12-13,16]
**809**	RP_G_GABP + ERKPP <-> RP_G_GABP_ERKPP	c608r		0.1	s^{-1}	33, 35, 36 (*S1.1*) + [4,12-13,16]
**810**	RP_G_GABP_ERKPP -> RP_G + GABPP + ERKPP	c609	c609*RP_G_GABP_ERKPP	1	s^{-1}	33, 35, 36 (*S1.1*) + [4,12-13,16]
**811**	ERBP_ShP_G_GABP + ERKPP <-> ERBP_ShP_G_GABP_ERKPP	c610f	c610f*ERBP_ShP_G_GABP*ERKPP - c610r*ERBP_ShP_G_GABP_ERKPP	0.01	$nM^{-1}\cdot s^{-1}$	33, 35, 36 (*S1.1*) + [4,12-13,16]
**812**	ERBP_ShP_G_GABP + ERKPP <-> ERBP_ShP_G_GABP_ERKPP	c610r		0.1	s^{-1}	33, 35, 36 (*S1.1*) + [4,12-13,16]
**813**	ERBP_ShP_G_GABP_ERKPP -> ERBP_ShP_G + GABPP + ERKPP	c611	c611*ERBP_ShP_G_GABP_ERKPP	1	s^{-1}	33, 35, 36 (*S1.1*) + [4,12-13,16]
**814**	ERBP_G_GABP + ERKPP <-> ERBP_G_GABP_ERKPP	c612f	c612f*ERBP_G_GABP*ERKPP - c612r*ERBP_G_GABP_ERKPP	0.01	$nM^{-1}\cdot s^{-1}$	33, 35, 36 (*S1.1*) + [4,12-13,16]
**815**	ERBP_G_GABP + ERKPP <-> ERBP_G_GABP_ERKPP	c612r		0.1	s^{-1}	33, 35, 36 (*S1.1*) + [4,12-13,16]
**816**	ERBP_G_GABP_ERKPP -> ERBP_G + GABPP + ERKPP	c613	c613*ERBP_G_GABP_ERKPP	1	s^{-1}	33, 35, 36 (*S1.1*) + [4,12-13,16]
**817**	ERB3P_G_GABP + ERKPP <-> ERB3P_G_GABP_ERKPP	c614f	c614f*ERB3P_G_GABP*ERKPP - c614r*ERB3P_G_GABP_ERKPP	0.01	$nM^{-1}\cdot s^{-1}$	33, 35, 36 (*S1.1*) + [4,12-13,16]
**818**	ERB3P_G_GABP + ERKPP <-> ERB3P_G_GABP_ERKPP	c614r		0.1	s^{-1}	33, 35, 36 (*S1.1*) + [4,12-13,16]
**819**	ERB3P_G_GABP_ERKPP -> ERB3P_G + GABPP + ERKPP	c615	c615*ERB3P_G_GABP_ERKPP	1	s^{-1}	33, 35, 36 (*S1.1*) + [4,12-13,16]
**820**	ERB3P_ShP_G_GABP + ERKPP <-> ERB3P_ShP_G_GABP_ERKPP	c616f	c616f*ERB3P_ShP_G_GABP*ERKPP - c616r*ERB3P_ShP_G_GABP_ERKPP	0.01	$nM^{-1}\cdot s^{-1}$	33, 35, 36 (*S1.1*) + [4,12-13,16]
**821**	ERB3P_ShP_G_GABP + ERKPP <-> ERB3P_ShP_G_GABP_ERKPP	c616r		0.1	s^{-1}	33, 35, 36 (*S1.1*) + [4,12-13,16]
**822**	ERB3P_ShP_G_GABP_ERKPP -> ERB3P_ShP_G + GABPP + ERKPP	c617	c617*ERB3P_ShP_G_GABP_ERKPP	1	s^{-1}	33, 35, 36 (*S1.1*) + [4,12-13,16]
**823**	GABP + ERKPP <-> GABP_ERKPP	c618f	c618f*GABP*ERKPP - c618r*GABP_ERKPP	0.01	$nM^{-1}\cdot s^{-1}$	33, 35, 36 (*S1.1*) + [4,12-13]
**824**	GABP + ERKPP <-> GABP_ERKPP	c618r		0.1	s^{-1}	33, 35, 36 (*S1.1*) + [4,12-13]
**825**	GABPP + PhoA <-> GABPP_PhoA	c619f	c619f*GABPP*PhoA - c619r*GABPP_PhoA	0.01	$nM^{-1}\cdot s^{-1}$	33, 35, 36 (*S1.1*) + [4,12-13]
**826**	GABPP + PhoA <-> GABPP_PhoA	c619r		0.1	s^{-1}	33, 35, 36 (*S1.1*) + [4,12-13]
**827**	GABPP_PhoA -> GABP + PhoA	c620	c620*GABPP_PhoA	1	s^{-1}	33, 35, 36 (*S1.1*) + [4,12-13]

**828**	GABP_ERKPP -> GABPP + ERKPP	c621	c621*GABP_ERKPP	1	s^{-1}	33, 35, 36 (*S1.1*) + [4,12-13]
**829**	ARF + MDM2 <-> [ARF_ MDM2)	c622f	c622f*ARF*MDM2 - c622r*[ARF_ MDM2)	0.01	$nM^{-1}\cdot s^{-1}$	40, 41 (*S1.1*) + [12-13]
**830**	ARF + MDM2 <-> [ARF_ MDM2)	c622r		0.001	s^{-1}	40, 41 (*S1.1*) + [12-13]
**831**	CYCLIND1 + CDK4 <-> CYCLIND1_CDK4	c623f	c623f*CYCLIND1*CDK4 - c623r*CYCLIND1_CDK4	0.01	$nM^{-1}\cdot s^{-1}$	92 (*S1.1*) + [12-13]
**832**	CYCLIND1 + CDK4 <-> CYCLIND1_CDK4	c623r		0.1	s^{-1}	92 (*S1.1*) + [12-13]
**833**	CYCLINE + CDK2 <-> CYCLINE_CDK2	c624f	c624f*CYCLINE*CDK2 - c624r*CYCLINE_CDK2	0.01	$nM^{-1}\cdot s^{-1}$	92 (*S1.1*) + [12-13]
**834**	CYCLINE + CDK2 <-> CYCLINE_CDK2	c624r		0.1	s^{-1}	92 (*S1.1*) + [12-13]
**835**	E2F + DP1 <-> E2F_DP1	c625f	c625f*E2F*DP1 - c625r*E2F_DP1	0.01	$nM^{-1}\cdot s^{-1}$	93, 94 (*S1.1*) + [12-13]
**836**	E2F + DP1 <-> E2F_DP1	c625r		0.1	s^{-1}	93, 94 (*S1.1*) + [12-13]
**837**	E2F_DP1 + RB <-> E2F_DP1_RB	c626f	c626f*E2F_DP1*RB - c626r*E2F_DP1_RB	0.01	$nM^{-1}\cdot s^{-1}$	93, 94 (*S1.1*) + [12-13]
**838**	E2F_DP1 + RB <-> E2F_DP1_RB	c626r		0.1	s^{-1}	93, 94 (*S1.1*) + [12-13]
**839**	CYCLIND1_CDK4 + RB <-> CYCLIND1_CDK4_RB	c627f	c627f*CYCLIND1_CDK4*RB - c627r*CYCLIND1_CDK4_RB	0.01	$nM^{-1}\cdot s^{-1}$	92 (*S1.1*) + [12-13]
**840**	CYCLIND1_CDK4 + RB <-> CYCLIND1_CDK4_RB	c627r		0.1	s^{-1}	92 (*S1.1*) + [12-13]
**841**	CYCLIND1_CDK4_RB -> CYCLIND1_CDK4 + RBP	c628	c628*CYCLIND1_CDK4_RB	1	s^{-1}	92 (*S1.1*) + [12-13]
**842**	CYCLINE_CDK2 + RBP <-> CYCLINE_CDK2_RBP	c629f	c629f*CYCLINE_CDK2*RBP - c629r*CYCLINE_CDK2_RBP	0.01	$nM^{-1}\cdot s^{-1}$	92 (*S1.1*) + [12-13]
**843**	CYCLINE_CDK2 + RBP <-> CYCLINE_CDK2_RBP	c629r		0.1	s^{-1}	92 (*S1.1*) + [12-13]
**844**	CYCLINE_CDK2_RBP -> CYCLINE_CDK2 + RBPP	c630	c630*CYCLINE_CDK2_RBP	1	s^{-1}	92 (*S1.1*) + [12-13]
**845**	RBPP + Pho14 <-> RBPP_Pho14	c631f	c631f*RBPP*Pho14 - c631r*RBPP_Pho14	0.01	$nM^{-1}\cdot s^{-1}$	92 (*S1.1*) + [12-13]
**846**	RBPP + Pho14 <-> RBPP_Pho14	c631r		0.1	s^{-1}	92 (*S1.1*) + [12-13]
**847**	RBPP_Pho14 -> RBP + Pho14	c632	c632*RBPP_Pho14	1	s^{-1}	92 (*S1.1*) + [12-13]
**848**	RBP + Pho14 <-> RBP_Pho14	c633f	c633f*RBP*Pho14 - c633r*RBP_Pho14	0.01	$nM^{-1}\cdot s^{-1}$	92 (*S1.1*) + [12-13]
**849**	RBP + Pho14 <-> RBP_Pho14	c633r		0.1	s^{-1}	92 (*S1.1*) + [12-13]
**850**	RBP_Pho14 -> RB + Pho14	c634	c634*RBP_Pho14	1	s^{-1}	92 (*S1.1*) + [12-13]
**851**	E2F_DP1_RB + TFBSV <-> E2F_DP1_RB_TFBSV	c635f	c635f*E2F_DP1_RB*TFBSV - c635r*E2F_DP1_RB_TFBSV	0.01	$nM^{-1}\cdot s^{-1}$	92, 93 (*S1.1*) + [12-13]
**852**	E2F_DP1_RB + TFBSV <-> E2F_DP1_RB_TFBSV	c635r		0.1	s^{-1}	92, 93 (*S1.1*) + [12-13]

**853**	E2F_DP1 + TFBSV <-> E2F_DP1_TFBSV	c636f	c636f*E2F_DP1*TFBSV - c636r*E2F_DP1_TFBSV	0.01	$nM^{-1}\cdot s^{-1}$	92, 93 (*S1.1*) + [12-13]
**854**	E2F_DP1 + TFBSV <-> E2F_DP1_TFBSV	c636r		0.1	s^{-1}	92, 93 (*S1.1*) + [12-13]
**855**	CYCLIND1_CDK4 + P16 <-> CYCLIND1_CDK4_P16	c637f	c637f*CYCLIND1_CDK4*P16 - c637r*CYCLIND1_CDK4_P16	0.01	$nM^{-1}\cdot s^{-1}$	92, 93 (*S1.1*) + [12-13]
**856**	CYCLIND1_CDK4 + P16 <-> CYCLIND1_CDK4_P16	c637r		0.1	s^{-1}	92, 93 (*S1.1*) + [12-13]
**857**	CDC25C + ERKPP <-> CDC25C_ERKPP	c642f	c642f*CDC25C*ERKPP - c642r*CDC25C_ERKPP	0.01	$nM^{-1}\cdot s^{-1}$	26, 27 (*S1.1*) + [12-13]
**858**	CDC25C + ERKPP <-> CDC25C_ERKPP	c642r		0.01	s^{-1}	26, 27 (*S1.1*) + [12-13]
**859**	CDC25C_ERKPP -> CDC25CP + ERKPP	c643	c643*CDC25C_ERKPP	10	s^{-1}	26, 27 (*S1.1*) + [12-13]
**860**	CDC25CP + Pho16 <-> CDC25CP_Pho16	c644f	c644f*CDC25CP*Pho16 - c644r*CDC25CP_Pho16	0.01	$nM^{-1}\cdot s^{-1}$	26, 27 (*S1.1*) + [12-13]
**861**	CDC25CP + Pho16 <-> CDC25CP_Pho16	c644r		10	s^{-1}	26, 27 (*S1.1*) + [12-13]
**862**	CDC25CP_Pho16 -> CDC25C + Pho16	c645	c645*CDC25CP_Pho16	0.1	s^{-1}	26, 27 (*S1.1*) + [12-13]
**863**	Pb_MYC -> MYC_mRNA	c646	c646*MYC_mRNA_Gen	0.00005	s^{-1}	[21]
**864**	MYC_mRNA -> null	c647	c647*MYC_mRNA	0.00027	s^{-1}	95 (*S1.1*) + [22]
**865**	PKP_PIP2 + PI3K_inhibitor <-> PKP_PIP2_PI3K_inhibitor	c648f	c648f*PKP_PIP2*PI3K_inhibitor - c648r*PKP_PIP2_PI3K_inhibitor	0.01	$nM^{-1}\cdot s^{-1}$	[23]
**866**	PKP_PIP2 + PI3K_inhibitor <-> PKP_PIP2_PI3K_inhibitor	c648r		1.5	s^{-1}	[23]
**867**	Pb_CCND1 -> CCND1_mRNA	c649	c649*CCND1_mRNA_Gen	0.00005	s^{-1}	[21]
**868**	CCND1_mRNA -> null	c650	c650*(ERKPPcont / ERKPP) * CCND1_mRNA	0.0000057	s^{-1}	96 (*S1.1*) + [22]
**869**	GSKP + AZAKEN <-> GSKP_AZAKEN	c651f	c651f*GSKP*AZAKEN - c651r*GSKP_AZAKEN	0.01	$nM^{-1}\cdot s^{-1}$	[23]
**870**	GSKP + AZAKEN <-> GSKP_AZAKEN	c651r		0.18	s^{-1}	[23]

Supplementary Table 2.2 - Species Initial Concentration

Supplementary Table 2.2 reports the total concentration of 81 basic species (the total concentration of each species involved in biochemical reactions, adding up all the modified forms and complexes in which a given basic species is involved).

We considered growth factors (EGF, TGFβ, WNT) non-consumable. We considered the GDP and GTP species to be in large excess (non-consumable).

	Molecular species as written in the rates equations (simplified notation)	Concentrations (nM)	Molecular species as written in MIM's cartouches	Extrapolations from Annotation List References (ST 1.1) + Notes / Ref. listed below
**1**	R	100	EGFR (ErbB family)	[1-10]
**2**	EGF	0.1		[7]
**3**	PLCy	105		[1-10]
**4**	Grb	85	Grb2	[1-10]
**5**	SOS	10		[1-10]
**6**	Shc	100		[1-10]
**7**	Ras	85		[1-10]
**8**	GDP	500		[1-10]
**9**	GTP	10000		[1-10]
**10**	Raf	50	BRAF	[1-10]
**11**	Pase1 (BRAF phosphatase)	50	Pase1	[1-10]
**12**	MEK	200		[1-10]
**13**	Pase2 (MEK phosphatase)	50	Pase7	[1-10]
**14**	ERK	200		[1-10]
**15**	Pase3 (ERK phosphatase)	100	MKP3	[1-10]
**16**	GAP	12		[1-10]
**17**	Pase5 (SOS phosphatase)	50		[1-10]
**18**	GAB	50		[1-10]
**19**	PTEN	50		[1-10]
**20**	PhoA (GAB1 phosphatase)	100	Pase3	[1-10]
**21**	AKT	100		[1-10]

**22**	PDK1	100		[1-10]
**23**	TAKT (AKT phosphatase)	50	PHLPP	[1-10]
**24**	GSK	50	GSK3β	[1-10]
**25**	PhoB (PI3K phosphatase)	50	PI3K inactivator	[1-10]
**26**	PhoC (GSK3β)	20	Pase4	[1-10]
**27**	PI3K	200		[1-10]
**28**	APC	100		[22,29]
**29**	PhoE (AXN, APC, BetaCatenin, LRP5/6 phosphatase)	10	Pase6	49, 54, 55 (*S1.1*) + [29]
**30**	PTP (BetaCateninY654)	100	PTP1B	[1-10]
**31**	ERB	1	ErbB2 (ErbB family)	[1-10]
**32**	PTP1E (Shc phosphatase)	50		[1-10]
**33**	PaseX (PLCy phosphatase)	50	Pase2	[1-10]
**34**	ERB3	1	ErbB3 (ErbB family)	[1-10]
**35**	Cadh	50	E-Cadherin	[1-10]
**36**	TGF	0.01	TGFβ	[30]
**37**	PP1C	10		77 – 81 (*S1.1*) + [29]
**38**	SMADII	25		77 – 81 (*S1.1*) + [29]
**39**	TBRI	100	TGFβ receptor I	77 – 81 (*S1.1*) + [29]
**40**	TBRII	100	TGFβ receptor II	77 – 81 (*S1.1*) + [29]
**41**	SMADIII	5		77 – 81 (*S1.1*) + [29]
**42**	TAK	25	TAK1	82 – 84 (*S1.1*) + [29]
**43**	NLK	50		82 – 84 (*S1.1*) + [29]
**44**	TCFLEF	15	TCF7L2	[22,29]

**45**	Pase8 (TCF7L2 phosphatase)	10		[29]
**46**	Pase9 (TAK phosphatase)	10		82 – 84 (*S1.1*) + [29]
**47**	Pase10 (NLK phosphatase)	10		82 – 84 (*S1.1*) + [29]
**48**	SMAD4	25		77 – 81 (*S1.1*) + [29]
**49**	PP1A (SMADII, SMADIII phosphatase)	10	PPM1A	77 – 81 (*S1.1*) + [29]
**50**	TFBS	0.00166	$TFBS_{TCF7L2}$ (DNA BINDING SITE)	[29,31]
**51**	AP1	10		16, 17 (*S1.1*) + [29]
**52**	Pase12 (AP1 phosphatase)	20		[29]
**53**	TFBSII	0.00083	$TFBS_{AP1}$ (DNA BINDING SITE)	[29,31]
**54**	WNT	0.01		[29,32]
**55**	FRZ	100		70 – 75 (*S1.1*) + [29]
**56**	LRP6	100		70 – 75 (*S1.1*) + [29]
**57**	DVL	100		70 – 75 (*S1.1*) + [22,29]
**58**	AXN	0.02	Axin	70 – 75 (*S1.1*) + [22,29]
**59**	BetaCatenin_generator	0.01		[29]
**60**	PIP2_Gen	0.01		[29]
**61**	PKC	50		28 – 29 (*S1.1*) + [29]
**62**	TAB	25	TAB2	82 – 84 (*S1.1*) + [29]
**63**	GROUCHO	25		[29]
**64**	TFBSI	0.00083	$TFBS_{SMAD}$ (DNA BINDING SITE)	[29,31]
**65**	MDM2	10		[29]
**66**	TP53gen	0.1		[29]
**67**	Pho13 (MDM2 phosphatase)	50	Pase13	92 -94 (*S1.1*) + [29]

**68**	TFBSIV	0.00083	$TFBS_{TP53}$ (DNA BINDING SITE)	[29,31]
**69**	ARF	10		92 (*S1.1*) + [29]
**70**	CYCLIND1	10		92 (*S1.1*) + [29]
**71**	CDK4	10		92 (*S1.1*) + [29]
**72**	CYCLINE	10		92 (*S1.1*) + [29]
**73**	CDK2	10		92 (*S1.1*) + [29]
**74**	RB	10		92 -94 (*S1.1*) + [29]
**75**	E2F	10		92 -94 (*S1.1*) + [29]
**76**	DP1	10		92 -94 (*S1.1*) + [29]
**77**	Pho14 (RB phosphatase)	10	Pase14	[29]
**78**	TFBSV	0.00083	$TFBS_{E2F-DP1}$ (DNA BINDING SITE)	[29,31]
**79**	P16	10		92 -94 (*S1.1*) + [29]
**80**	Pho16 (CDC25C phosphatase)	5	Pase11	[29]
**81**	CDC25C (ErbB family phosphatase)	50		[29]

Supplementary references and notes to Table 2.1 – Reaction List and to Supplementary Table 2.2 - Species Initial Concentration:

1. Castagnino N, Tortolina L, Balbi A, Pesenti R, Montagna R, Ballestrero A, Soncini D, Moran E, Nencioni A and Parodi S. Dynamic simulations of pathways downstream of ERBB-family, including mutations and treatments: concordance with experimental results. Curr Cancer Drug Targets. 2010; 10(7):737-757.
2. Kholodenko BN, Demin OV, Moehren G and Hoek JB. Quantification of short term signaling by the epidermal growth factor receptor. The Journal of biological chemistry. 1999; 274(42):30169-30181.
3. Blinov ML, Faeder JR, Goldstein B and Hlavacek WS. A network model of early events in epidermal growth factor receptor signaling that accounts for combinatorial complexity. Bio Systems. 2006; 83(2-3):136-151.

4. Birtwistle MR, Hatakeyama M, Yumoto N, Ogunnaike BA, Hoek JB and Kholodenko BN. Ligand-dependent responses of the ErbB signaling network: experimental and modeling analyses. Molecular systems biology. 2007; 3:144.

5. Wolf J, Dronov S, Tobin F and Goryanin I. The impact of the regulatory design on the response of epidermal growth factor receptor-mediated signal transduction towards oncogenic mutations. FEBS J. 2007; 274(21):5505-5517.

6. Chen WW, Schoeberl B, Jasper PJ, Niepel M, Nielsen UB, Lauffenburger DA and Sorger PK. Input-output behavior of ErbB signaling pathways as revealed by a mass action model trained against dynamic data. Molecular systems biology. 2009; 5:239.

7. Borisov N, Aksamitiene E, Kiyatkin A, Legewie S, Berkhout J, Maiwald T, Kaimachnikov NP, Timmer J, Hoek JB and Kholodenko BN. Systems-level interactions between insulin-EGF networks amplify mitogenic signaling. Molecular systems biology. 2009; 5:256.

8. Schoeberl B, Eichler-Jonsson C, Gilles ED and Muller G. Computational modeling of the dynamics of the MAP kinase cascade activated by surface and internalized EGF receptors. Nature biotechnology. 2002; 20(4):370-375.

9. Kiyatkin A, Aksamitiene E, Markevich NI, Borisov NM, Hoek JB and Kholodenko BN. Scaffolding protein Grb2-associated binder 1 sustains epidermal growth factor-induced mitogenic and survival signaling by multiple positive feedback loops. The Journal of biological chemistry. 2006; 281(29):19925-19938.

10. Kholodenko BN, Hoek JB and Westerhoff HV. Why cytoplasmic signalling proteins should be recruited to cell membranes. Trends in cell biology. 2000; 10(5):173-178.

11. Stites EC, Trampont PC, Ma Z and Ravichandran KS. Network analysis of oncogenic Ras activation in cancer. Science. 2007; 318(5849):463-467.

12. The references associated with the Annotation List give information about all the interactions described in our MIM. If a good quality experimental paper says, for instance, that protein subspecies A (after a given posttranslational modification) interacts well with the molecule B, we can assume that we will have a 95% of [A:B].

13. There will be a free 5% of the less abundant partner. This could be a general default assumption, based on semi-quantitative information. The diffusion limit is 7 × 109•M-1•s-1. However, considering the very crowded cellular environment, the very large size of many multi–protein complexes, the fact that only a

fraction of protein–protein encounters will be productive, the contribution of the association rate to the equilibrium dissociation constant was fixed at 107·M-1·s-1, except for older values obtained by previous papers.

14. Similarly, a reasonable first order default dissociation rate constant could be in the order of 10-2-10-3•s-1. This ranges will insure a good affinity between the two interacting species. We have utilized the semi-quantitative information implicitly present in the papers quoted in the References accompanying the Annotation List according to the above considerations, to fill up gaps concerning rates and concentrations.
15. Numerical values have been interpolated by taking into account the constraints imposed by: a) existing values; b) molecular anatomy of the network; and c) indirect evidences at the molecular, cellular and clinical level, that we tried to satisfy. In general, the network system imposed relatively narrow ranges (3-5 times intervals) of the interpolated values. .
16. Leahy DJ. A molecular view of anti-ErbB monoclonal antibody therapy. Cancer cell. 2008; 13(4):291-293.
17. Verveer PJ, Wouters FS, Reynolds AR and Bastiaens PI. Quantitative imaging of lateral ErbB1 receptor signal propagation in the plasma membrane. Science. 2000; 290(5496):1567-1570.
18. The protein-protein interaction reactions of the receptors family ErbB was described in a simplified way, attributing the same rates to all the members of the family.
19. Zi Z and Klipp E. Constraint-based modeling and kinetic analysis of the Smad dependent TGF-beta signaling pathway. PloS one. 2007; 2(9):e936.
20. Clarke DC, Betterton MD and Liu X. Systems theory of Smad signalling. Systems biology. 2006; 153(6):412-424.
21. Vilar JM, Jansen R and Sander C. Signal processing in the TGF-beta superfamily ligand-receptor network. PLoS computational biology. 2006; 2(1):e3.
22. Lee E, Salic A, Kruger R, Heinrich R and Kirschner MW. The roles of APC and Axin derived from experimental and theoretical analysis of the Wnt pathway. PLoS biology. 2003; 1(1):E10.
23. see in the main text "Derivation of a Transcription Rate Function for MYC and CCND1" and S4.
24. β-catenin, PIP2 and TP53 were endowed with a slow production and degradation rate. In this way, the model would tend to a steady state rather than to a continuous accumulation or complete destruction of the two molecules. .

25. β-catenin protein will be rapidly degraded, and therefore set on a lower concentration level, in the presence of an intact destruction complex (Axin:APC:GSK3β). The model simulates a very slow degradation of a mutated β-catenin in HCT116 cells, or an APC inactivation in HT29 cells, and therefore a very slow β-catenin degradation also in these cells. .

26. PIP3, IP3, DAG were endowed with a slow degradation rate. In this way, the model would tend to a steady state rather than to a continuous accumulation of these molecules.

27. mRNA of MYC and CCND1 were endowed with a slow production and degradation rate. In this way, the model would tend to a steady state rather than to a continuous accumulation or complete destruction of the two messengers. See main text "Derivation of a Transcription Rate Function for MYC and CCND1" and S4 for the transcription rates of MYC (MYC_mRNA_Gen) and CCND1 (CCND1_mRNA_Gen) .

28. Inhibitors were implemented as follows: according to the information given by the company selling the inhibitor (see S3.1: Details about cell cultures and reagents) and according to the concentration of inhibitor used, we calculated different inhibition levels for each inhibitor.

29. Numerical values have been interpolated by taking into account the constraints imposed by: a) existing values; b) molecular anatomy of the network.

30. Wakefield LM, Smith DM, Masui T, Harris CC and Sporn MB. Distribution and modulation of the cellular receptor for transforming growth factor-beta. The Journal of cell biology. 1987; 105(2):965-975.

31. We assume that activators and repressor complexes can bind DNA in different sites. In the case of concentration value of TFBS, we considered two binding site: one binding site for TCF4 and one binding site for SMAD4. .

32. Carlson ME, Conboy MJ, Hsu M, Barchas L, Jeong J, Agrawal A, Mikels AJ, Agrawal S, Schaffer DV and Conboy IM. Relative roles of TGF-beta1 and Wnt in the systemic regulation and aging of satellite cell responses. Aging cell. 2009; 8(6):676-689.

Supplementary Material 3.1 - Cell cultures and reagents

Cells were cultured in DMEM (Gibco), supplemented with 10% heat–inactivated fetal bovine serum (FBS) (Hyclone), 2mM L-glutamine (Gibco) and 1% Penicillin-Streptomycin solution (Gibco). No EGF was added to our medium. Considering that we gave 10% serum, the corresponding simulations were performed at 1/10 the physiologic EGF concentration, therefore at 0.01 nM. The following inhibitors were used: MEK inhibitor CI-1040 (also known as PD184352) (Sigma-Aldrich) (kindly provided by Dr. Alex von Kriegsheim), PI3K inhibitor PI-103 (Sigma-Aldrich/Tocris) (kindly provided by Dr. Natalia Volinsky), AKT inhibitor Perifosine (Selleck), GSK3β inhibitor 1-Azakenpaullone (Sigma-Aldrich), and Tankyrase inhibitor XAV939 (Sigma-Aldrich). Perifosine was dissolved in ethanol (Et-OH), while all others inhibitors were dissolved in DMSO. At the level of final dilution we had [(1 ul Et-OH + 3ul DMSO)/(1 ml culture medium)]. We used CI-1040 at a final concentration of 2μM (IC50 ≈ 300nM. Inhibition of the target in our simulations: around 80% for both cell lines). We used PI-103 at 500nM (IC50 ≈ 150nM. Inhibition of the specific target in our simulations: around 85% for both cell lines). We used Perifosine at 20 and 40 μM (IC50 ≈ 4.8μM, inhibition of the specific target in our simulations ≈ 50% (20 μM) or ≈ 70% (40 μM) for both cell lines. We used Azakenpaullone at 1μM (IC50 ≈ 18nM, inhibition of the specific target in our simulations ≈ 98% for HT29). We used XAV939 at 1μM (IC50 7.5nM, inhibition of the specific target in our simulations ≈ 99% for both cell lines). These inhibition levels are derived from the IC50s given in the product data-sheets and our subsequent computations performed using our reaction list (Supplementary Table 2.1).

Supplementary material 3.2 - Western Blots: Methods and raw results

Preliminary experiments: We performed preliminary experiments to determine the optimal time to assay the proteins after addition of inhibitors, and to examine the effect of complete media change either on the day before addition of inhibitors or at the same time as inhibitors were added (data not shown).

We performed preliminary experiments to determine the optimal time to assay the proteins after addition of inhibitors (5', 10', 20', 30', 40' and 60' min), and to examine the effect of complete media change either on the day before addition of inhibitors or at the same time as inhibitors were added (data not shown). From these experiments we selected 30 min as a representative time to measure changes for protein and phospho-protein levels. A quasi plateau effect had been reached in the time interval 10' - 60' min. The timing of complete fresh media change did not alter the trend of experimental results, as long as controls were treated in the same manner. We also considered the practically negligible role of the vehicles used for

our inhibitors [(EtOH 1ul + DMSO 3 ul)/(1 ml culture medium)] (data not shown). After these preliminary experiments, all the combinations of different inhibitors were examined at 30' min incubation, after a change with complete fresh medium.

We seeded cells in 6 wells plates, to obtain an 80-90% confluence at the time of pharmacological treatment. After incubation with inhibitors, the cells were washed twice with cold PBS and lysed with lysis buffer (25mM Tris-phosphate (pH 7.8), 2mM DTT, 2mM EDTA, 2mM EGTA 10% glycerol, 1% Triton X-100) in the presence of two phosphatase inhibitors (50 mM NaF, 5 mM Na_3VO_4) and a protease inhibitor cocktail (Sigma-Aldrich). We sonicated all lysates for 10 seconds at power 2 with a MISONIX XL-2000 series sonicator. Thirty micrograms of whole lysate were resolved, for all samples, using an 8% acrylamide gel electrophoresis under reducing conditions. We transferred it to a PVDF membrane. The resulting membranes were blocked with 5% BSA (Sigma-Aldrich) diluted in a TBS solution with 0.1% Tween 20 (TBST), for 1 hour. Membranes were then incubated with primary antibodies: [HER2/ErbB2 (29D8) (#2165)]; [EGF receptor (D38B1) (#4267)]; [α/β-Tubulin (#2148)]; [β-Actin (#4970)]; [PTEN (#9552)]; [AKT (pan) (#4691)]; [phospho-AKT (Ser473) (#4060)]; [p44/42 MAPK (Erk1/2) (#9102)]; [phospho-44/42 MAPK (Erk1/2) (Thr202/Tyr204) (#9101)]. We used dilutions and protocols suggested by the supplier (Cell Signaling Technology). For the evaluation of phospho/dephospho ratios of AKT and ERK1/2, first we performed the detection using antibodies for phosphorylated states and, after stripping, the membranes were re-probed using the anti-total proteins antibodies. The stripping procedure adopted is according to Yee-Guide Yeung and E. Richard Stanley [1], based on the use of Guanidine hydrochloride. The stripped membranes were saturated with a solution of TBST- MILK 5%. We performed the detection using a secondary horseradish peroxidase-linked goat anti-rabbit antibody (Santa-Cruz) at a dilution of 1:5000 and an ECL chemi-luminescence system (Thermo Scientific). After Western blotting, we estimated the intensity of the bands obtained by a ChemidocXSR station using the software QuantityOne (Bio-Rad).

We used the reads from total protein amount of the same stripped membrane to standardize all the values obtained for the phosphorylated state. To obtain a comprehensive assessment of the phosphorylated versus non phosphorylated ratio change, for each experimental condition, the data were further expressed as values related to the "not treated " condition of the same membrane, were the ratio were arbitrary fixed to be equal to 1. For both cell lines the treatments were replicated by three independent experiments and the statistical analysis of the data were approached by GraphPad Prism 5.

PTEN, ErbB2, EGFR expression analysis in HCT116 and HT29 cancer lines

HCT116 and HT29 mutations / alterations present in our MIM are according to Cancer Cell Line Encyclopedia [2, 3].

To determine the concentrations to be used in the modeling of our MIM, for the cell lines used for our experimental verifications, we performed a semi-quantitative determination by western blot analysis, for protein PTEN, ErbB2 and EGFR. Two total cell lysates were used for each cell line, corresponding to untreated samples, obtained from independent experiments. We transferred and evaluated all samples on the same PVDF membrane and we normalized the results using the anti-tubulin antibody.

HCT116 had not completely lost PTEN, despite the homozygous mutation in the 3'-UTR of its messenger, but showed only a reduction of expression of about 35%, compared to the HT29 cancer line. Concerning the expression of EGF's receptors, HT29 showed a double amount of ErbB2 compared to HCT116, while had a reduction of expression of about 20% with regard to the presence of EGFR.

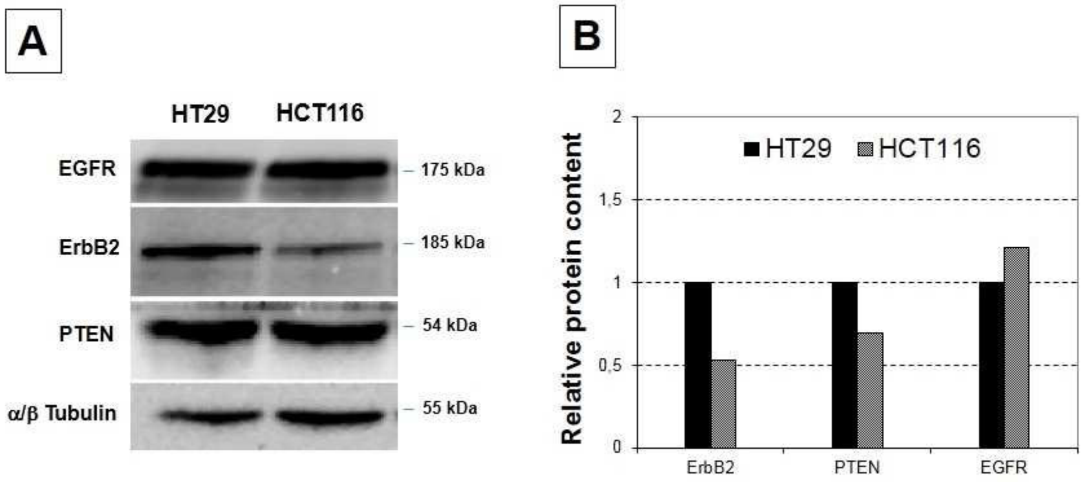

Supplementary Figure 3.2.1: PTEN, ErbB2, EGFR expression analysis. Panel A: evaluation of the relative content of ErbB2, PTEN, and EGFR, in HT29 and HCT116 cell lines. We used tubulin to normalize the values. Here we show a representative panel for the semi-quantitative comparison.

Panel B: The histogram shows the relative protein content for ErbB2, PTEN and EGFR in HT29 and HCT116 cells respectively, obtained by averaging the values from two independent experiments. To better compare the relative protein content in the two cell lines, for each protein the values obtained in the HT29 cell line, were made = 1.

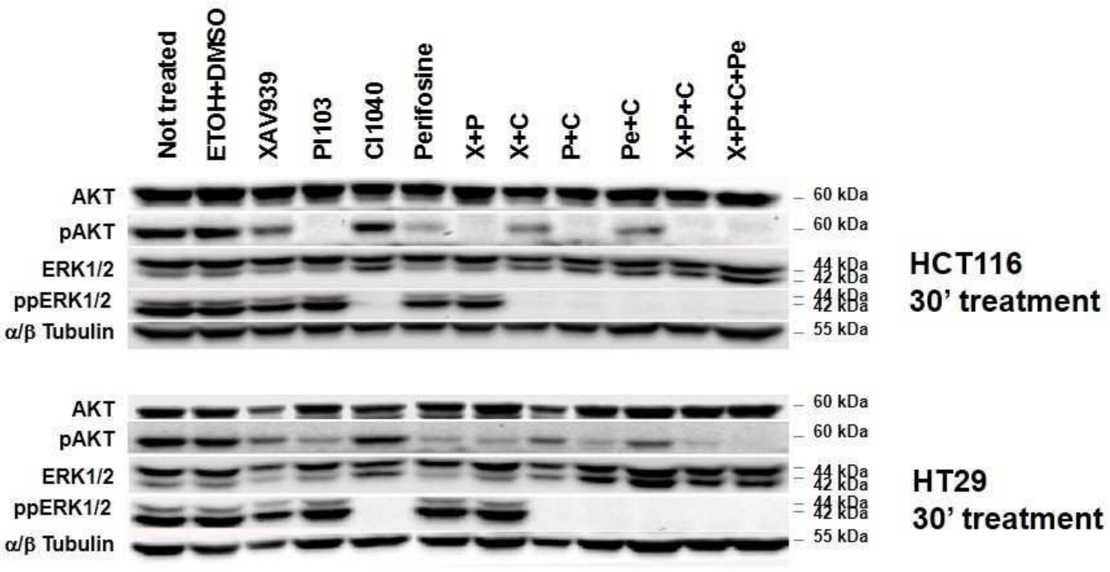

Supplementary Figure 3.2.2: **pAKT/AKT and ppERK1/2/ERK ratio analyses in HT26 and HCT116 cell lines.** Representative panel of a semi-quantitative analysis for pAKT/AKT and ppERK1/2/ERK ratio change during a pharmacological treatment of 30 minutes in HCT116 and HT29 cell lines, respectively.

We resolved 30 ug of total lysate for each experimental condition by 8% acrylamide gel electrophoresis under reducing conditions. The values referred to phosphorylated proteins (pAKT and ppERK1/2) were normalized using the real total amount of the same protein present on the PVDF membrane, detected after stripping and re-probing (AKT and ERK1/2). We introduced a further loading control using an α/β Tubulin antibody.

Inhibitors' abbreviations: XAV939 (X), PI-103 (P), CI-1040 (C), Perifosine (Pe).

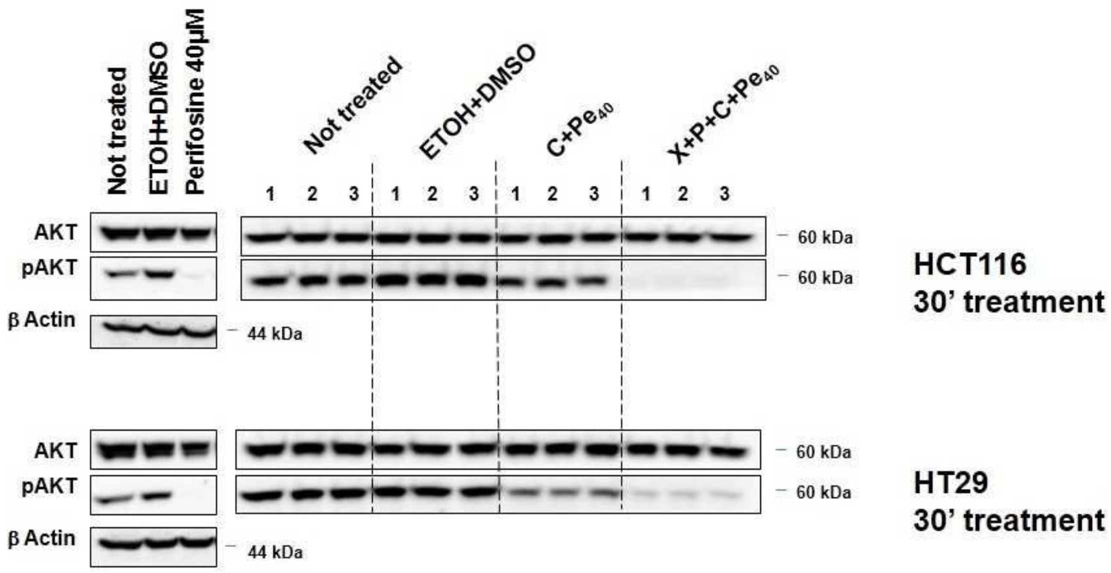

Supplementary Figure 3.2.3: **pAKT/AKT ratio analysis in HT26 and HCT116 cell lines, using Perifosine 40uM.** Representative panels of a semi-quantitative analyses for pAKT/AKT ratio change during pharmacological treatments of 30 minutes in HCT116 and HT29 cell lines by using Perifosine 40uM alone (panels on the left) or in combination with others inhibitors (panels on the right). The panels on the left show only one representative experiment of a total of three evaluated in the main text for the treatments with Perifosine 40uM alone, while the panels on the right show all the biological triplicate experiments on the same membrane of the treatments with inhibitors' combinations. We resolved 30 ug of total lysate for each experimental condition by 8% acrylamide gel electrophoresis under reducing conditions. The values referred to phosphorylated protein pAKT was normalized using the real total amount of the same protein present on the PVDF membrane. We introduced a further loading control using an β-Actin antibody. Inhibitors' abbreviations: XAV939 (X), PI-103 (P), CI-1040 (C), Perifosine (Pe40).

References to Supplementary Material 3.2 - Western Blots

1. Yeung YG and Stanley ER. A solution for stripping antibodies from polyvinylidene fluoride immunoblots for multiple reprobing. Analytical biochemistry. 2009; 389(1):89-91.
2. The Cancer Cell Line Encyclopedia.
3. Barretina J, Caponigro G, Stransky N, Venkatesan K, Margolin AA, Kim S, Wilson CJ, Lehar J, Kryukov GV, Sonkin D, Reddy A, Liu M, Murray L, Berger MF, Monahan JE, Morais P, et al. The Cancer Cell Line Encyclopedia enables predictive modelling of anticancer drug sensitivity. Nature. 2012; 483(7391):603-607.

Supplementary Figure 4.1 - Illustration of the transcription factors and transcription factor binding sites considered in the *Promoter/TF/RNAP* and *TFBS/TF/RNAP* systems

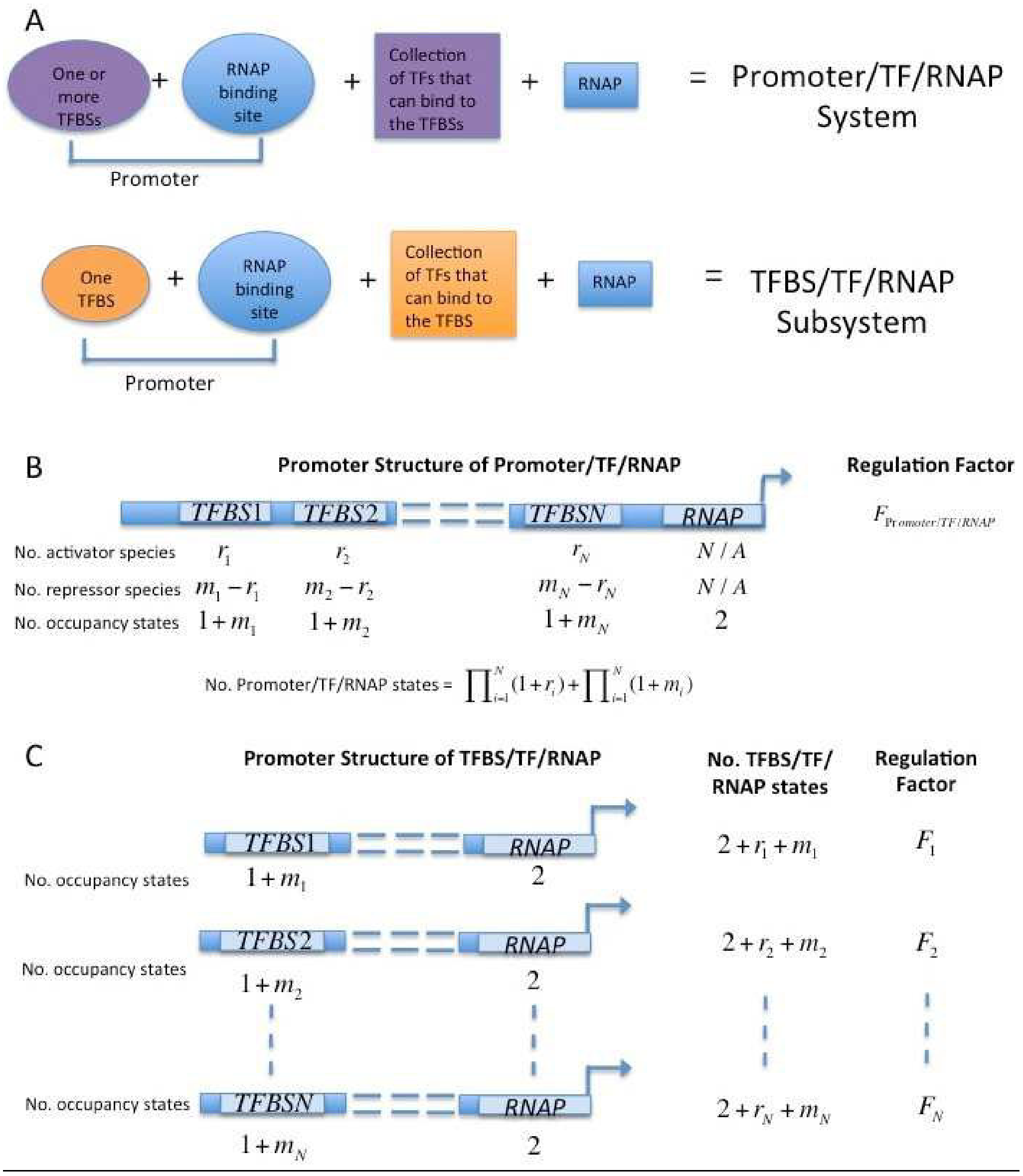

Supplementary Figure 4.1. Illustration of the transcription factors and transcription factor binding sites considered in the *Promoter/TF/RNAP* and *TFBS/TF/RNAP* systems. A. *Promoter/TF/RNAP* and *TFBS/TF/RNAP* are collections of promoter binding sites (ovals) and the proteins that bind to them (rectangles). Binding sites and their binding proteins can be associated with RNAP (blue) or TFs (purple or

orange). *Promoter/TF/RNAP* consists of one or more TFBSs, an RNAP binding site, all TFs that can bind to the TFBSs and RNAP. *TFBS/TF/RNAP* consists of one TFBS, an RNAP binding site and all TFs that can bind to the TFBS. **B.** *Promoter/TF/RNAP* is illustrated for a promoter with N TFBSs. The nth TFBS can be unoccupied or occupied by any one of the m_n TFs assumed to bind to that site, for $n = 1, \dots, N$. Activators constitute r_n of the m_n TFs. The regulation factor of the gene represented by *Promoter/TF/RNAP* is denoted by $F_{Promoter/TF/RNAP}$. The number of terms constituting $F_{Promoter/TF/RNAP}$ equals the number of possible occupancy states of *Promoter/TF/RNAP*.

Supplementary Material 4.2 - Literature background describing the involvement of key transcription factors and transcription factor binding sites in regulation of MYC and CCND1 transcription

The first step in building the thermo-statistical model involves identifying key transcription factor binding sites (TFBSs) responsible for MYC and CCND1 activation and repression, as well as the main TFs that bind to them. We have considered only some of the most important and best studied TFs (see reviews on MYC transcription [1, 2] and CCND1 transcription [3, 4]).

We start with TCF7L2, also known as TCF4, which is the most prominently expressed member of the TCF family of TFs in the intestinal epithelium, and a crucial transcriptional regulator in intestinal cells in particular [1, 5, 6]. TCF7L2 binding sites (termed $TFBS_{TCF7L2}$ in our model) have been found in both the MYC and CCND1 promoters, and have been shown to bind a number of complexes responsible for activation or repression of transcription. A complex of TCF7L2 and β-catenin can activate transcription of either gene through binding to $TFBS_{TCF7L2}$ [7-10]. Phosphorylation of β-catenin at Y654 in the cytoplasm results in its release from cadherins (especially type E) and facilitates its migration to the nucleus, with a consequent increase in TCF-mediated transcriptional activity [8, 11]. TCF7L2 binding sites can also bind the transcription activator complex TCF7L2-SMAD4, or, in contrast, mediate repression of transcription by binding a complex of TCF7L2 and GROUCHO [12-14].

SMAD4 can alternatively heterodimerise with TGF-β receptor regulated SMADs (SMAD2 or SMAD3), whose affinity for nuclear factors increases upon their phosphorylation (15, 16). These SMAD complexes can then bind to a SMAD binding site ($TFBS_{SMAD}$) on MYC, repressing its transcription [15-17]. A similar inhibitory role of the TGF-β pathway on CCDN1 transcription has been described [18, 19]. SMAD4 (most likely complexed with phosphorylated SMAD2 or SMAD3) binds to the promoter region of CCND1 upon TGF-β treatment, repressing transcription [20].

AP1 (activating protein-1) is the collective term applied to dimeric transcription factors with subunits consisting of Jun, Fos or ATF (activating transcription factor) [21]. AP1, depending on its composition, has

been shown to activate or repress CCND1, and its activity can be modulated by phosphorylation [3, 22]. In the model, we assume a net-positive effect of AP1 on CCND1 transcription [23-25], where CCND1 has a consensus AP1 ($TFBS_{AP1}$) site in its promoter [3, 25]. A canonical AP1 responsive element has also been identified in the MYC promoter [26] and MYC transcription is known to be activated by the MAPK pathway, which mediates AP1 phosphorylation [27] .

The tumor suppressor protein TP53 binds to the MYC promoter on a $TFBS_{TP53}$ site and represses MYC transcription through a mechanism that involves histone deacetylation [28]. TP53 also represses CCND1 transcription indirectly, by down regulating the transcriptional co-activator Bcl-3 [4, 29]. Additionally, the CCND1 promoter contains a functional (ChIP confirmed) human NF-κB binding site at -39bp, which binds TP53 [4].

MYC and CCND1 are target genes of E2F, a family of proteins that require a dimerization partner (DP) protein in order to bind DNA [30, 31]. RB protein transrepresses E2F target genes through binding to E2F-DP complexes on the gene promoter at a $TFBS_{E2F-DP1}$ site [17, 30]. Multi-phosphorylation of RB results in dissociation of RB from E2F-DP1 and subsequent activation of the E2F-DP1 complex, allowing for activation of transcription [17, 30, 31].

For convenience, we introduce shorthand designations for the TF activator and repressor complexes that bind to the promoter regions of MYC and CCND1 in our model. We assume that activators β-Catenin-TCF7L2 (x_1), pβ-Catenin(Y654)-TCF7L2 (x_1p), SMAD4-TCF7L2 (x_2) and repressor GROUCHO-TCF7L2 (y_1) compete for binding to the same site, $TFBS_{TCF7L2}$. Likewise, pSMAD2-SMAD4 (y_2) and pSMAD3-SMAD4 (y_3) repressor complexes compete for $TFBS_{SMAD4}$. AP1 (x_3) and pAP1 (x_3p) bind mutually exclusively to $TFBS_{AP1}$. Finally, we assume that TP53 (y_4) binds to $TFBS_{TP53}$, and that E2F-DP1 (x_4) competes with its repressor counterpart E2F-DP1-RB (y_5) for $TFBS_{E2F-DP1}$.

Supplementary Material 4.3 - Derivation of the transcription rate function for MYC and CCND1

Regulation of transcription is typically multi-factorial, involving a series of transcriptional activators, repressors and co-factors that control the recruitment of the transcriptional machinery and RNA Polymerase (RNAP) to the transcription start site [32, 33]. With reference to eukaryotic / mammalian cells mRNAs, we have in mind RNAP II. Several useful tools have been employed to investigate the relationship between active TF concentrations and the mRNA levels of genes under their control. One approach is that of a statistical thermodynamic framework [34-36], which we have applied to relate MYC and CCND1 transcription rates to the concentrations of their upstream transcriptional activator and repressor complexes.

The first step in building the thermo-statistical model was to identify key transcription factor binding sites (TFBSs) responsible for activation or repression of MYC and CCND1 transcription, as well as the main TFs that bind to them (see Fig. 2 and Supplementary Material 4.2 for details). As these genes have similar key transcriptional regulators, for the purposes of the model we assume that they have equivalent promoter regions and the same transcription rates. The entire system consisting of all five TFBSs, all TFs considered in the MIM and RNAP (RNA-Polymerase), is denoted as a *Promoter/TF/RNAP* system, whereas the designation *TFBS/TF/RNAP* refers to a subsystem of *Promoter/TF/RNAP* where only one TFBS, the corresponding TFs and RNAP are included (Supplementary Fig. 4.1). For instance, $TFBS_{TCF7L2}$ together with the competing activator complexes β-Catenin- TCF7L2, pβ-CateninY654-TCF7L2, SMAD4-TCF7L2 and repressor complex GROUCHO-TCF7L2, and the RNAP binding site is denoted as $TFBS_{TCF7L2}/TF/RNAP$.

Transcription occurs when RNAP binds to the promoter, and the transcription rate is assumed to be proportional to the probability that RNAP is bound [34, 37]. Promoter-TF interactions change the probability of RNAP binding to the promoter and, therefore, the transcription rate. There are many possible states in which the individual *TFBS/TF/RNAP* subsystems and the entire *Promoter/TF/RNAP* system can reside. These states tell us, which (if any) TFs are bound to TFBSs, and whether RNAP is bound. Two rules impact on the number of possible states of *TFBS/TF/RNAP* (or *Promoter/TF/RNAP*). First, we assume that RNAP and a repressor TF cannot be simultaneously bound to the promoter region, and secondly, we assume that

two or more TFs competing for a TFBS cannot be simultaneously bound to it. According to Boltzmann statistics, each state can be assigned a statistical weight, and the probability of occurrence of each state is proportional to its statistical weight. The proportionality constant is the sum of statistical weights over all potential states. Expressions for the statistical weights for different *TFBS/TF/RNAP* states are given elsewhere (Supplementary Material 4.5, Supplementary Tables 4.7- 4.12), while here we describe the probability of RNAP being bound to or unbound from the promoter, $\mathrm{P(RNAP_{bound})}$, in terms of these weights. The probability $\mathrm{P(RNAP_{bound})}$ is proportional to the sum of the statistical weights for all DNA states where RNAP is bound, denoted as $\mathrm{S(RNAP_{bound})}$. Similarly, the probability of RNAP not being bound to the DNA is proportional to the sum of the statistical weights of all states where RNAP is not bound to the DNA, denoted as $\mathrm{S(RNAP_{unbound})}$.

We describe these relationships mathematically as,

$$\mathrm{P(RNAP_{bound})} = \frac{\mathrm{S(RNAP_{bound})}}{\mathrm{Z}}, \quad \mathrm{P(RNAP_{unbound})} = \frac{\mathrm{S(RNAP_{unbound})}}{\mathrm{Z}} \tag{1}$$

where the proportionality coefficient Z is the partition function equal to the sum of $\mathrm{S(RNAP_{bound})}$ and $\mathrm{S(RNAP_{unbound})}$.

Consequently,

$$\mathrm{P(RNAP_{bound})} = \frac{\mathrm{S(RNAP_{bound})}}{\mathrm{S(RNAP_{bound})} + \mathrm{S(RNAP_{unbound})}} \tag{2}$$

To simplify the algebraic expressions, it is convenient to introduce the regulation factor of a gene, $\mathrm{F_{reg}}$, which is directly expressed in terms of $\mathrm{P(RNAP_{bound})}$, as follows [35, 36]. We take the sum of the statistical weights of all DNA states where RNAP is bound to the promoter and divide it by the sum of the statistical weights where RNAP is not bound, and normalize it by the weight of the reference state of RNAP binding (in the absence of all TFs). This weight is expressed as $\frac{\mathrm{[RNAP]}}{\mathrm{K_{RNAP}}}$, where [RNAP] denotes the cellular concentration of RNAP and $\mathrm{K_{RNAP}}$ is the equilibrium dissociation constant of the RNAP-DNA complex [38].

For a consideration about possible values in mammalian cells (in the absence of all TFs) of the ratio $\frac{\mathrm{[RNAP]}}{\mathrm{K_{RNAP}}}$, look at Supplementary Material 4.6.

F_{reg} is defined as,

$$F_{reg} = \left(\frac{[RNAP]}{K_{RNAP}}\right)^{-1} \frac{S(RNAP_{bound})}{S(RNAP_{unbound})} = \left(\frac{[RNAP]}{K_{RNAP}}\right)^{-1} \frac{P(RNAP_{bound})}{P(RNAP_{unbound})} =$$

$$= \left(\frac{[RNAP]}{K_{RNAP}}\right)^{-1} \frac{P(RNAP_{bound})}{1 - P(RNAP_{bound})} \qquad (3)$$

From Eqn. 3, it follows that the probability of RNAP to be bound to the promoter can be expressed in terms of the regulation factor, as follows,

$$P(RNAP_{bound}) = \frac{1}{1+\left(\frac{[RNAP]}{K_{RNAP}}F_{reg}\right)^{-1}} \qquad (4)$$

We now need to decompose the probability $P(RNAP_{bound})$ into the relative contributions of each TFBS in recruiting RNAP (or inhibiting the recruitment of RNAP) to its binding site, assuming independence of TFBSs. Importantly, the regulation factor of a gene with multiple independent TFBSs is expressed as the product of the regulation factors for each *TFBS/TF/RNAP* subsystem (see a mathematical derivation in Supplementary Material 4.4). Therefore, the regulation factor for our genes of interest is given by,

$$F_{Promoter/TF/RNAP} = F_{TCF7L2} * F_{SMAD4} * F_{AP1} * F_{TP53} * F_{E2F-DP1} \qquad (5)$$

where $F_{Promoter/TF/RNAP}$ is the regulation factor of the entire *Promoter/TF/RNAP* system and $F_{TCF7L2}, F_{SMAD}, F_{AP1}, F_{TP53}$ and $F_{E2F-DP1}$ are the regulation factors of the *TFBS/TF/RNAP* subsystems, corresponding to the independent single sites, $TFBS_{TCF7L2}$, $TFBS_{SMAD4}$, $TFBS_{AP1}$, $TFBS_{TP53}$ and $TFBS_{E2F\text{-}DP1}$, respectively. The transcription rates of MYC and CCND1 are obtained from Eqn. 5, as described in Supplementary Material 4.6.

Supplementary Material 4.4 - Derivation of the regulation factor $F_{Promoter/TF/RNAP}$ in terms of the regulation factors of independent $TFBS/TF/RNAP$ subsystems

Consider a gene promoter with *N* distinct TFBSs, where at each site there are a number of different types of activator and repressor TF complexes competing for binding to the DNA. To find an expression for the probability that RNAP is bound to the promoter we need to consider all possible promoter-binding patterns that can occur, exhaustively allocating statistical weights to all combinatorial possibilities. We assume that all TFBSs are non-overlapping and each promoter-bound activator TF can interact with RNAP, decreasing the free energy of RNAP binding to the promoter. Rather than listing all possible states of the promoter, we show here that we can simplify derivation of the regulation factor of the full promoter, $F_{Promoter/TF/RNAP}$, by writing it as a product of the regulation factors of the individual TFBSs on the promoter as follows (Eqn. 5 of Supplementary Document 4.3):

$$F_{Promoter/TF/RNAP} = F_{TCF7L2} * F_{SMAD} * F_{AP1} * F_{TP53} * F_{E2F-DP1} \quad (1)$$

In the general case, where there are *N* independent TFBSs, and F_n is the regulation factor for the *TFBS/TF/RNAP* subsystem number *n*, we claim that the regulation factor of the full system can be expressed as the product of all F_n:

$$F_{Promoter/TF/RNAP} = \prod_{n=1}^{N} F_n \quad (2)$$

The remainder of this section is dedicated to providing a proof for Eqn. 2, a convenient shortcut for computing the regulation factor of a gene with many TFBSs, as previously proposed in the literature without a formal proof (1). We first rewrite Eqn. 2 in terms of probabilities of $RNAP$ binding to the N-site promoter (also known as the *Promoter/TF/RNAP* system), as follows:

$$\left(\frac{[RNAP]}{K_{RNAP}}\right)^{-1}\frac{P(RNAP_{bound})}{P(RNAP_{unbound})}$$

$$= \prod_{n=1}^{N}\left(\frac{[RNAP]}{K_{RNAP}}\right)^{-1}\frac{P(RNAP_{bound}|only\ TFBS\ n\ occupied)}{P(RNAP_{unbound}|only\ TFBS\ n\ occupied)} \tag{3}$$

We aim now to prove that Eqn. 3 (which is equivalent to Eqn. 2) holds for an N-site promoter by splitting the probabilities into their statistical weight components (see Supplementary Document *4.5* for an example of how to calculate statistical weights). Suppose that at each site $n = 1, \dots, N$ there are m_n types of TF complexes (of which r_n are activators, $0 \le r_n \le m_n$) competing for binding to the DNA. For each site n we order the numbering of the m_n TF species at each site such that the activators have the lower valued subscripts and define the functions, h_n and g_n, as follows

$$h_n = \sum_{i=1}^{m_n}\frac{[TF_{n,i}]}{K_{TF_{n,i}}},\ g_n = \sum_{i=1}^{r_n}\frac{[TF_{n,i}]}{K_{TF_{n,i}}}e^{-\Delta\varepsilon_{RNAP-TF_{n,i}}}, \tag{4}$$

where $TF_{n,i}$ is the ith TF species competing for TFBS n, $K_{TF_{n,i}}$ is the equilibrium dissociation constant of $TF_{n,i}$ and TFBS *n,* and $\Delta\varepsilon_{RNAP-TF_{n,i}}$ (in units of RT) is the negative-valued change in the free energy of RNAP binding to the promoter induced by $TF_{n,i}$ bound to TFBS *n.* The function h_n equals the total statistical weight of occupancy of TFBS *n* by activator or repressor TFs (within the *Promoter/TF/RNAP* system) when all other TFBSs are unoccupied and RNAP is not bound to the promoter h_n. The function g_n equals the total statistical weight of occupancy of TFBS *n*, when all other TFBSs are free but RNAP is bound to the DNA.

For independent TFBSs, the statistical weights representing the occupancy of multiple sites with RNAP being bound or not bound to the promoter can be written in terms of sums and products of the elementary h_n and g_n terms, as presented below [36]. The statistical weight of all possible cases of occupancy of one or more than one TFBS when RNAP is not bound is given by the sums and products of the corresponding h_n terms, leading directly to the following expression for the probability $P(RNAP_{unbound})$ of RNAP being not bound to the promoter,

$$P(RNAP_{unbound})$$
$$= (1 + \Sigma_{i=1}^{N} h_i + \Sigma_{i=1}^{N} h_i \Sigma_{j=i+1}^{N} h_j + \Sigma_{i=1}^{N} h_i \Sigma_{j=i+1}^{N} h_j \Sigma_{k=j+1}^{N} h_k + \cdots + \Sigma_{i_1=1}^{N} h_{i_1} \Sigma_{i_2=i_1+1}^{N} h_{i_2} * \dots \quad (5)$$
$$* \Sigma_{i_{n-1}=N-1}^{N} h_{i_{n-1}} + \Sigma_{i_1=1}^{N} h_{i_1} \Sigma_{i_2=i_1+1}^{N} h_{i_2} * \dots * \Sigma_{i_{n-1}=N-1}^{N} h_{i_{n-1}} * \Sigma_{i_n=N}^{N} h_{i_n})/Z$$

where the successive terms correspond to the promoter states when RNAP is unbound and no TF is bound to DNA, only one TFBS is occupied, two TFBSs are occupied, …, and all *N* TFBSs are occupied. *Z* is the standard partition function that is the sum of statistical weights of all possible promoter-binding scenarios. The probability $P(RNAP_{unbound})$ is a polynomial expression of *N* arguments $h_1, h_2, \dots, h_N$ and can be factorized by extracting h_1 from the expression Eqn. 5 as follows:

$$P(RNAP_{unbound})$$
$$= (1 + (h_1 + \Sigma_{i=2}^{N} h_i) + (h_1 \Sigma_{j=2}^{N} h_j + \Sigma_{i=2}^{N} h_i \Sigma_{j=i+1}^{N} h_j)$$
$$+ (h_1 \Sigma_{j=2}^{N} h_j \Sigma_{k=j+1}^{N} h_k + \Sigma_{i=2}^{N} h_i \Sigma_{j=i+1}^{N} h_j \Sigma_{k=j+1}^{N} h_k) + \cdots + (h_1 \Sigma_{i_2=2}^{N} h_{i_2} * \dots * \Sigma_{i_{n-1}=N-1}^{N} h_{i_{n-1}}$$
$$+ \Sigma_{i_1=2}^{N} h_{i_1} \Sigma_{i_2=i_1+1}^{N} h_{i_2} * \dots * \Sigma_{i_{n-1}=N-1}^{N} h_{i_{n-1}}) + (h_1 \Sigma_{i_2=2}^{N} h_{i_2} * \dots * \Sigma_{i_{n-1}=N-1}^{N} h_{i_{n-1}} * \Sigma_{i_n=N}^{N} h_{i_n}$$
$$+ \Sigma_{i_1=2}^{N} h_{i_1} \Sigma_{i_2=i_1+1}^{N} h_{i_2} * \dots * \Sigma_{i_{n-1}=N-1}^{N} h_{i_{n-1}} * \Sigma_{i_n=N}^{N} h_{i_n}))/Z$$
$$= ((1 + h_1) + (1 + h_1)\Sigma_{i=2}^{N} h_i + (1 + h_1)\Sigma_{i=2}^{N} h_i \Sigma_{j=i+1}^{N} h_j + \cdots$$
$$+ (1 + h_1)\Sigma_{i_1=2}^{N} h_{i_1} \Sigma_{i_2=i_1+1}^{N} h_{i_2} * \dots * \Sigma_{i_{n-1}=N-1}^{N} h_{i_{n-1}} + (1 + h_1)\Sigma_{i_1=2}^{N} h_{i_1} \Sigma_{i_2=i_1+1}^{N} h_{i_2} * \dots \quad (6)$$
$$* \Sigma_{i_{n-1}=N-1}^{N} h_{i_{n-1}} * \Sigma_{i_n=N}^{N} h_{i_n})/Z$$
$$= (1 + h_1)(1 + \Sigma_{i=2}^{N} h_i + \Sigma_{i=2}^{N} h_i \Sigma_{j=i+1}^{N} h_j + \Sigma_{i_1=2}^{N} h_{i_1} \Sigma_{i_2=i_1+1}^{N} h_{i_2} * \dots * \Sigma_{i_{n-1}=N-1}^{N} h_{i_{n-1}}$$
$$+ \Sigma_{i_1=2}^{N} h_{i_1} \Sigma_{i_2=i_1+1}^{N} h_{i_2} * \dots * \Sigma_{i_{n-1}=N-1}^{N} h_{i_{n-1}} * \Sigma_{i_n=N}^{N} h_{i_n})/Z$$

We can now extract the h_2 terms in the same way:

$$P(RNAP_{unbound})$$
$$= (1 + h_1)(1 + (h_2 + \Sigma_{i=3}^{N} h_i) + \left(h_2 \Sigma_{j=3}^{N} h_j + \Sigma_{i=3}^{N} h_i \Sigma_{j=i+1}^{N} h_j\right)$$
$$+ \left(h_2 \Sigma_{i_2=3}^{N} h_{i_2} * \dots * \Sigma_{i_{n-1}=N-1}^{N} h_{i_{n-1}} + \Sigma_{i_1=3}^{N} h_{i_1} \Sigma_{i_2=i_1+1}^{N} h_{i_2} * \dots * \Sigma_{i_{n-1}=N-1}^{N} h_{i_{n-1}}\right)$$
$$+ (h_2 \Sigma_{i_2=3}^{N} h_{i_2} * \dots * \Sigma_{i_{n-1}=N-1}^{N} h_{i_{n-1}} * \Sigma_{i_n=N}^{N} h_{i_n} + \Sigma_{i_1=3}^{N} h_{i_1} \Sigma_{i_2=i_1+1}^{N} h_{i_2} * \dots * \Sigma_{i_{n-1}=N-1}^{N} h_{i_{n-1}} \quad (7)$$
$$* \Sigma_{i_n=N}^{N} h_{i_n}))/Z$$
$$= (1 + h_1)(1 + h_2)(1 + \Sigma_{i=3}^{N} h_i + \Sigma_{i=3}^{N} h_i \Sigma_{j=i+1}^{N} h_j + \Sigma_{i_1=3}^{N} h_{i_1} \Sigma_{i_2=i_1+1}^{N} h_{i_2} * \dots$$
$$* \Sigma_{i_{n-1}=N-1}^{N} h_{i_{n-1}} + \Sigma_{i_1=3}^{N} h_{i_1} \Sigma_{i_2=i_1+1}^{N} h_{i_2} * \dots * \Sigma_{i_{n-1}=N-1}^{N} h_{i_{n-1}} * \Sigma_{i_n=N}^{N} h_{i_n})/Z$$

Continuing in this manner for $h_3, h_4, \dots, h_N$ leads to the simplified expression

$$P(RNAP_{unbound}) = \prod_{i=1}^{N} (1 + h_i)/Z \quad (8)$$

In order to derive the probability $P(RNAP_{bound})$ of RNAP being bound to the promoter, we reuse the assumption that each TFBS acts independently facilitating or repressing RNAP binding. This means that TFs bound to different TFBSs do not directly interact with each other and independently contribute or repress the RNAP binding to the promoter. These assumptions allow us to generate a simplified expression for $P(RNAP_{bound})$ in terms of $g_1, g_2, \dots, g_N$ similar to that derived for $P(RNAP_{unbound})$ as follows:

$$P(RNAP_{bound})$$
$$= \frac{[RNAP]}{K_{RNAP}}(1 + \Sigma_{i=1}^{N} g_i + \Sigma_{i=1}^{N} g_i \Sigma_{j=i+1}^{N} g_j + \Sigma_{i=1}^{N} g_i \Sigma_{j=i+1}^{N} g_j \Sigma_{k=j+1}^{N} g_k + \cdots$$
$$+ \Sigma_{i_1=1}^{N} g_{i_1} \Sigma_{i_2=i_1+1}^{N} g_{i_2} * \dots * \Sigma_{i_{n-1}=N-1}^{N} g_{i_{n-1}} + \Sigma_{i_1=1}^{N} g_{i_1} \Sigma_{i_2=i_1+1}^{N} g_{i_2} * \dots * \Sigma_{i_{n-1}=N-1}^{N} g_{i_{n-1}} \quad (9)$$
$$* \Sigma_{i_n=N}^{N} g_{i_n})/Z$$
$$= \frac{[RNAP]}{K_{RNAP}} \prod_{i=1}^{N} (1 + g_i)/Z = \left(\frac{[RNAP]}{K_{RNAP}}\right)^{-N+1} \prod_{i=1}^{N} \frac{[RNAP]}{K_{RNAP}} \frac{(1 + g_i)}{Z}$$

The second equality follows by analogy with the expression for $P(RNAP_{unbound})$.

Thus, by Eqns. (3 (Supplementary Material 4.3), 8 and 9) we can write the following:

$$F_{Promoter/TF/RNAP} = \left(\frac{[RNAP]}{K_{RNAP}}\right)^{-1} \frac{P(RNAP_{bound})}{P(RNAP_{unbound})} = \left(\frac{[RNAP]}{K_{RNAP}}\right)^{-N} \frac{\prod_{i=1}^{N} \frac{[RNAP]}{K_{RNAP}}(1+g_i)/Z}{\prod_{i=1}^{N}(1+h_i)/Z} \tag{10}$$

The usefulness of Eqn. 10 is shown in the following expression, where $Z_1, Z_2, \ldots, Z_N$ are the partition functions for the *TFBS/TF/RNAP* subsystems where only TFBS1, TFBS2, …, or TFBSN can be occupied by TFs, respectively:

$$\left(\frac{[RNAP]}{K_{RNAP}}\right)^{-1} \frac{P(RNAP_{bound})}{P(RNAP_{unbound})} = \left(\frac{[RNAP]}{K_{RNAP}}\right)^{-N} \frac{\prod_{i=1}^{N} \frac{[RNAP]}{K_{RNAP}}(1+g_i)/Z_i}{\prod_{i=1}^{N} \frac{1+h_i}{Z_i}}$$
$$= \left(\frac{[RNAP]}{K_{RNAP}}\right)^{-N} \prod_{i=1}^{N} \frac{P(RNAP_{bound} | only\ TFBS\ n\ occupied)}{P(RNAP_{unbound} | only\ TFBS\ n\ occupied\ )} \tag{11}$$

Thus, we have proved that Eqn. 2 holds. Therefore, we can compute the regulation factor for a complex *Promoter/TF/RNAP* system by calculating the individual regulation factors for each *TFBS/TF/RNAP* subsystem and using the above equation.

It follows that for MYC and CCND1 transcription Eqn. 1 holds (which is Eqn. 5 of Supplementary Material 4.3).

Supplementary Material 4.5 - Example: Detailed derivation of the regulation factor $F_{E2F-DP1}$ for the *TFBS/TF/RNAP* subsystem associated with the E2F-DP1 transcription factor binding site

As an illustrative example, we present here a derivation of the regulation factor $F_{E2F-DP1}$ for the E2F-DP1 binding-site ($TFBS_{E2F-DP1}$) in terms of the activator (E2F-DP1, x_4) and repressor (E2F-DP1-RB, y_5) concentrations. The shorthand notation for the activators and repressors is as defined in Supplementary

Material 4.2 and Supplementary Fig. 4.1. The regulation factors F_{TFBS} for the remaining TFBSs are expressed in Supplementary Tables 4.7 - 4.12. There are five possible states of the TFBS$_{E2F\text{-}DP1}$/TF/RNAP subsystem (Table *4.7*): state 1 is the unbound promoter reference state, state 2 where only $RNAP$ is bound, states 3 and 4 where the activator x_4 is bound in the absence or presence of $RNAP$, respectively, and state 5 where only the repressor y_5 is bound to the promoter (y_5 and $RNAP$ cannot bind to the DNA simultaneously, as we assume that transcription cannot occur in the presence of the repressor). The statistical weight of each of these states is determined by the product of a cellular concentration term and a Boltzmann factor. The concentration determines the number of ways this state (*i*) can occur, and the Boltzmann factor $\exp(-\varepsilon_i/RT)$ of state i is the exponential of minus the free energy change of the state relative to the reference state, where free energy changes arise from association and dissociation of complexes of TFs, RNAP and DNA. The free energy change ε_i is measured in units of RT (*R* is the gas constant, *T* is absolute temperature). Since we deal with free energy changes rather than absolute values we assume zero free energy of the reference state of the unbound promoter, and consequently a statistical weight equal to 1. Two of the five possible states of $TFBS_{E2F-DP1}/TF/RNAP$ subsystem have RNAP bound to the promoter (states 2 and 4, Supplementary Table 4.7), where the activator x_4 is either unbound or promoter-bound. Their statistical weights (w_2 and w_4, respectively) are expressed as,

$$w_2 = \frac{[RNAP]}{K_{RNAP}}, \qquad w_4 = \frac{[x_4][RNAP]}{K_{x_4}K_{RNAP}} e^{\frac{-\varepsilon_{x_4R}}{RT}} \tag{1}$$

where K_{x_4} is the dissociation constant of the activator x_4 and *TFBS*$_{E2F\text{-}DP1}$, ε_{x_4R} is the free energy change of RNAP binding induced by the interaction of E2F-DP1 and the transcriptional machinery, and concentrations are denoted by square brackets. For the three remaining states (1, 3 and 5), RNAP is unbound, and their statistical weights are given by,

$$w_1 = 1, \;\; w_3 = [x_4]/K_{x_4}, \;\; w_5 = [y_5]/K_{y_5} \tag{2}$$

Using Eqn. 3 (Supplementary Material 4.3), we obtain the regulation factor $F_{E2F-DP1}$ as the ratio of probabilities of $RNAP$ being bound or not bound to the gene promoter as follows:

$$F_{\mathrm{E2F-DP1}} = \left(\frac{[RNAP]}{K_{RNAP}}\right)^{-1} \frac{w_2 + w_4}{w_1 + w_3 + w_5} = \frac{1 + \frac{[x_4]}{K_{x_4}} e^{-\frac{\varepsilon_{x_4 R}}{RT}}}{1 + \frac{[x_4]}{K_{x_4}} + \frac{[y_5]}{K_{y_5}}} = \frac{1 + A_6 \frac{[x_4]}{K_{x_4}}}{1 + \frac{[x_4]}{K_{x_4}} + \frac{[y_5]}{K_{y_5}}} \quad (3)$$

The Boltzmann factor $A_6 = e^{-\varepsilon_{x_4 R}/RT}$ is larger than 1, since the free energy change $\varepsilon_{x_4 R}$ is negative, because the activator complex E2F-DP1 lowers the energy of RNAP binding to DNA.

The regulation factors for the other *TFBS/TF/RNAP* subsystems in the model are derived in the same manner as presented here. The regulation factors and statistical weights of associated promoter states are shown in Supplementary Tables 4.7 – 4.11, and summarized in Supplementary Table 4.12.

Supplementary Material 4.6 - Final expression for the transcription rate of MYC and CCND1.

Introductory note: Regulation of RNA Polymerase II (RNAP for short) transcription is a very complex event in mammals (and eukaryotes) [39]. RNAP itself is made of 12 subunits in humans. Sequence-specific DNA binding factors establish a bridge / communication with general/basal factors through an assortment of co-regulators (mediator complex). Recognition sites for sequence-specific factors tend to be arranged in clusters. In addition, there is associated involvement of chromatin-remodeling factors which catalyze covalent modifications of histones and other proteins. Complexity is further increased by cis-acting at long distance sequences (> 1kbp), either upstream or downstream of RNA transcription start, controlled by specific enhancer/regulator proteins. Post-translational modifications can modulate the DNA affinity of sequence-specific factors. Specific phosphorylations can for instance increase this affinity [39].

A chain of events starting with the cooperative presence of sequence specific factors co-opts mediator-complex-proteins and finally the core-transcription-complex containing RNAP [40]. In the absence of this chain of events, RNA polymerase II is generally thought to recognize, at low affinity, DNA non-specifically, with a weak intrinsic preference for Inr (initiator) consensus sequences (Py-Py-A-N-T/A-Py-Py in mammals) [40].

In calculation of the transcription rate in the thermostatistical model, the number of RNAP molecules in the nucleus of mammalian cells must be considered. Hieda *et al*. [41], in Table 1 of their work, report the number of the different forms of the largest (catalytic) subunit of RNA polymerase II, for HeLa cells. They report the total number of RNAPII nuclear molecules to be approximately 320,000. Maul and Deaven [42], in Table 1 of their work, give an estimation of the nuclear volume of different cell types. For HeLa cells, the nuclear volume was found to be approximately 374 µm^3 (3.74·10^-13 liters). Dividing the number of nuclear molecules by the ratio of Avogadro's number to nuclear volume, RNAPII nuclear molarity can be estimated to be around 1·10^-4 M.

K_{RNAP} is the equilibrium dissociation constant for a non-specific binding of RNAP II to chromatin-DNA, in the absence of all TFs. Because of a non-specific type of binding, K_{RNAP} will tend to be high (rapid

dissociation of RNAP from chromatin-DNA). For this base-line non-specific chromatin-DNA binding, we estimated the K_{RNAP} equilibrium dissociation constant to be roughly around 10^-2 - 10^-3 M. Accordingly, we have tested the predictions of our simulations arranging the value of $[RNAP]/K_{RNAP}$ between .01 and .1. Concordance with experimental results remained quite good, substantially invariant for the two values.

Computation of transcription rates: By the results of Supplementary Material 4.4 and the expressions of the regulation factors for the other TFBSs (Supplementary Tables 4.7 - 4.11), we obtain the regulation factor for the *Promoter/TF/RNAP* system of our genes of interest, as follows,

$$F_{Promoter/TF/RNAP} = \frac{1 + A_1 \frac{[x_1]}{K_{x_1}} + A_2 \frac{[x_1 p]}{K_{x_1 p}} + A_3 \frac{[x_2]}{K_{x_2}}}{1 + \frac{[x_1]}{K_{x_1}} + \frac{[x_1 p]}{K_{x_1 p}} + \frac{[x_2]}{K_{x_2}} + \frac{[y_1]}{K_{y_1}}} * \frac{1 + A_4 \frac{[x_3]}{K_{x_3}} + A_5 \frac{[x_3 p]}{K_{x_3 p}}}{1 + \frac{[x_3]}{K_{x_3}} + \frac{[x_3 p]}{K_{x_3 p}}} * \frac{1 + A_6 \frac{[x_4]}{K_{x_4}}}{1 + \frac{[x_4]}{K_{x_4}} + \frac{[y_5]}{K_{y_5}}} * \frac{1}{1 + \frac{[y_2]}{K_{y_2}} + \frac{[y_3]}{K_{y_3}}} * \frac{1}{1 + \frac{[y_4]}{K_{y_4}}} \quad (1)$$

Here the K values denote the equilibrium dissociation constants of the TF-TFBS complexes, and the A_i terms are dimensionless multipliers (the Boltzmann factors, whose expressions are given in Supplementary Tables 4.7 - 4.11). The multipliers A_i are larger than 1, because RNAP binding to DNA is energetically favorable, and therefore has a negative free energy.

Finally, the probability that RNAP is bound to the promoter is obtained from Eqns. 1 and 4 of Supplementary Material 4.4) .

$$P(RNAP_{bound})$$
$$= \frac{[RNAP]}{K_{RNAP}}\left(\left(1 + A_1\frac{[x_1]}{K_{x_1}} + A_2\frac{[x_1p]}{K_{x_1p}} + A_3\frac{[x_2]}{K_{x_2}}\right) * \left(1 + A_4\frac{[x_3]}{K_{x_3}} + A_5\frac{[x_3p]}{K_{x_3p}}\right) * \left(1 + \ A_6\frac{[x_4]}{K_{x_4}}\right)\right)$$
$$/\left(\frac{[RNAP]}{K_{RNAP}}\left(\left(1 + A_1\frac{[x_1]}{K_{x_1}} + A_2\frac{[x_1p]}{K_{x_1p}} + A_3\frac{[x_2]}{K_{x_2}}\right) * \left(1 + A_4\frac{[x_3]}{K_{x_3}} + A_5\frac{[x_3p]}{K_{x_3p}}\right) * \left(1 + \ A_6\frac{[x_4]}{K_{x_4}}\right)\right)\right. \quad (2)$$
$$+ \left(1 + \frac{[x_1]}{K_{x_1}} + \frac{[x_1p]}{K_{x_1p}} + \frac{[x_2]}{K_{x_2}} + \frac{[y_1]}{K_{y_1}}\right) * \left(1 + \frac{[y_2]}{K_{y_2}} + \frac{[y_3]}{K_{y_3}}\right) * \left(1 + \frac{[x_3]}{K_{x_3}} + \frac{[x_3p]}{K_{x_3p}}\right) * \left(1 + \frac{[y_4]}{K_{y_4}}\right)$$
$$\left. * \left(1 + \frac{[x_4]}{K_{x_4}} + \frac{[y_5]}{K_{y_5}}\right)\right)$$

The K and A terms are dissociation constants and exponential functions of free energy changes, respectively, as previously described.

We assume in our model that no one TFBS or TF dominates the control of the promoter over the transcription rate, but that phosphorylated TFs have a greater affinity for the transcriptional machinery than their unphosphorylated counterparts [43, 44]. Therefore, we choose $A_1, A_3, A_4 = 2$ and $A_2, A_5 = 10$. Similar values with similar ratios also provided a satisfactory fit between the qPCR data and the output of the simulated MIM.

The transcription rate is proportional to the probability $P(RNAP_{bound})$ of RNAP being bound to the gene of interest [38], and taking into account the mRNA decay rate k_{deg}, we finally obtain the equation for the time evolution of mRNA concentrations, which we use in our model,

$$\frac{d}{dt}mRNA = k * P(RNAP_{bound}) - k_{deg}mRNA \quad (3)$$

Supplementary Table 4.7 - Computation of regulation factor $F_{E2F-DP1}$

Promoter-Bound (0 or 1)			*Statistical Weight of the Promoter State*
x_4	y_5	$RNAP$	
0	0	0	$w_1 = 1$
0	0	1	$w_2 = \frac{[RNAP]}{K_{RNAP}}$
1	0	0	$w_3 = \frac{[x_4]}{K_{x_4}}$
1	0	1	$w_4 = A_6 \frac{[x_4][RNAP]}{K_{x_4} K_{RNAP}}, A_6 = e^{\frac{-\varepsilon_{x_4 R}}{RT}}$
0	1	0	$w_5 = \frac{[y_5]}{K_{y_5}}$
$F_{E2F-DP1} = \left(\frac{[RNAP]}{K_{RNAP}}\right)^{-1} \frac{w_2 + w_4}{w_1 + w_3 + w_5} = \frac{1 + A_6 \frac{[x_4]}{K_{x_4}}}{1 + \frac{[x_4]}{K_{x_4}} + \frac{[y_5]}{K_{y_5}}}$			

Supplementary Table 4.7 - Computation of regulation factor $F_{E2F-DP1}$. Enumeration and statistical weight evaluation of each possible state for a promoter subsystem (*TFBS*$TFBS_{E2F-DP1}$*/TF/RNAP*) with an $RNAP$ site and a single $TFBS_{E2F-DP1}$ that binds either activator complex E2F-DP1 (x_4) or transcriptional repressor complex E2F-DP1-RB (y_5). The parameter $\varepsilon_{x_4 R}$ is the free energy change of RNAP binding, induced by the interaction of x_4 and the transcriptional machinery. This free energy change is negative for an activator, thus the Boltzmann factor A_6 is positive. The function $F_{E2F-DP1}$ is the regulation factor associated with $TFBS_{E2F-DP1}$*/TF/RNAP*.

Supplementary Table 4.8 - Computation of regulation factor F_{TCF7L2}

Promoter-Bound(0 or 1)					*Statistical Weight of the Promoter State*
x_1	x_1p	x_2	y_1	$RNAP$	
0	0	0	0	0	$w_1 = 1$
0	0	0	0	1	$w_2 = \frac{[RNAP]}{K_{RNAP}}$
1	0	0	0	0	$w_3 = \frac{[x_1]}{K_{x_1}}$
1	0	0	0	1	$w_4 = A_1 \frac{[x_1][RNAP]}{K_{x_1} K_{RNAP}}, A_1 = e^{-\frac{\varepsilon_{x_1 R}}{RT}}$
0	1	0	0	0	$w_5 = \frac{[x_1p]}{K_{x_1p}}$
0	1	0	0	1	$w_6 = A_2 \frac{[x_1p][RNAP]}{K_{x_1p} K_{RNAP}}, A_2 = e^{-\frac{\varepsilon_{x_1pR}}{RT}}$
0	0	1	0	0	$w_7 = \frac{[x_2]}{K_{x_2}}$
0	0	1	0	1	$w_8 = A_3 \frac{[x_2][RNAP]}{K_{x_2} K_{RNAP}}, A_3 = e^{-\frac{\varepsilon_{x_2 R}}{RT}}$
0	0	0	1	0	$w_9 = \frac{[y_1]}{K_{y_1}}$

$$F_{TCF7L2} = \left(\frac{[RNAP]}{K_{RNAP}}\right)^{-1} \left(\frac{w_2 + w_4 + w_6 + w_8}{w_1 + w_3 + w_5 + w_7 + w_9}\right)$$

$$= \frac{1 + A_1 \frac{[x_1]}{K_{x_1}} + A_2 \frac{[x_1p]}{K_{x_1p}} + A_3 \frac{[x_2]}{K_{x_2}}}{1 + \frac{[x_1]}{K_{x_1}} + \frac{[x_1p]}{K_{x_1p}} + \frac{[x_2]}{K_{x_2}} + \frac{[y_1]}{K_{y_1}}}$$

Supplementary Table 4.8 - Computation of regulation factor F_{TCF7L2}. The F_{TCF7L2} regulation factor for the *TFBS$_{TCF7L2}$/TF/RNAP* subsystem is computed via enumeration and statistical weight evaluation of each possible state of the system. This site has three activator TF complexes and one repressor TF complex

competing for binding to it: β-Catenin-TCF7L2 (x_1), pβ-Catenin(Y654)-TCF7L2 (x_1p), SMAD4-TCF7L2 (x_2) and repressor GROUCHO-TCF7L2 (y_1). There are nine possible states of *TFBS$_{TCF7L2}$/TF/RNAP*: each of the activator complexes singly binding to the DNA where the $RNAP$ is bound or not bound to its site, the unbound (reference) state, the inhibitor y_1-bound state and the state where $RNAP$ is bound without the presence of an activator. Note that where we consider a transcriptional repressor we assume that $RNAP$ cannot bind to the promoter to activate transcription. The parameters ε_{x_1R}, ε_{x_1pR} and ε_{x_2R} (measured in units of RT) are the free energy changes of RNAP binding induced by the interaction of bound x_1, x_1p or x_2 with the transcriptional machinery. These free energy changes are negative for activators, thus the multipliers A_1, A_2 and A_3 are positive.

Supplementary Table 4.9 - Computation of regulation factor F_{SMAD4}

Promoter-Bound (0 or 1)			*Statistical Weight of the Promoter State*
y_2	y_3	$RNAP$	
0	0	0	$w_1 = 1$
0	0	1	$w_2 = \frac{[RNAP]}{K_{RNAP}}$
1	0	0	$w_3 = \frac{[y_2]}{K_{y_2}}$
0	1	0	$w_4 = \frac{[y_3]}{K_{y_3}}$
$F_{SMAD4} = \left(\frac{[RNAP]}{K_{RNAP}}\right)^{-1} \frac{w_2}{w_1 + w_3 + w_4} = \frac{1}{1 + \frac{[y_2]}{K_{y_2}} + \frac{[y_3]}{K_{y_3}}}$			

Supplementary Table 4.9 - Computation of regulation factor F_{SMAD4}. The F_{SMAD4} regulation factor for the *$TFBS_{SMAD4}$/TF/RNAP* subsystem is computed via enumeration and statistical weight evaluation of each possible state of the system. There are four states of this promoter: each of the repressor complexes, pSMAD2(S467)-SMAD4 (y_2) and pSMAD3(S425)-SMAD4 (y_3), singly binding to the DNA, the unbound (reference) state and the state where only $RNAP$ is bound. Note that where we consider a transcriptional repressor we assume that $RNAP$ cannot bind to the promoter to activate transcription.

Supplementary Table 4.10 - Computation of regulation factor F_{AP1}

Promoter-Bound (0 or 1)			*Statistical Weight of the Promoter State*
x_3	x_3p	$RNAP$	
0	0	0	$w_1 = 1$
0	0	1	$w_2 = \frac{[RNAP]}{K_{RNAP}}$
1	0	0	$w_3 = \frac{[x_3]}{K_{x_3}}$
1	0	1	$w_4 = A_4 \frac{[RNAP][x_3]}{K_{RNAP}K_{x_3}}, A_4 = e^{-\frac{\varepsilon_{x_3R}}{RT}}$
0	1	0	$w_5 = \frac{[x_3p]}{K_{x_3p}}$
0	1	1	$w_6 = A_5 \frac{[RNAP][x_3p]}{K_{RNAP}K_{x_3p}}, A_5 = e^{-\frac{\varepsilon_{x_3pR}}{RT}}$
$F_{AP1} = \left(\frac{[RNAP]}{K_{RNAP}}\right)^{-1} \left(\frac{w_2 + w_4 + w_6}{w_1 + w_3 + w_5}\right) = \frac{1 + A_4 \frac{[x_3]}{K_{x_3}} + A_5 \frac{[x_3p]}{K_{x_3p}}}{1 + \frac{[x_3]}{K_{x_3}} + \frac{[x_3p]}{K_{x_3p}}}$			

Supplementary Table 4.10 - Computation of regulation factor F_{AP1}. The F_{AP1} regulation factor for the *TFBS$_{AP1}$/TF/RNAP* subsystem is computed via enumeration and statistical weight evaluation of each possible state of the system. There are six possible states of this promoter: each of the activator complexes, AP1 (x_3) and pAP1 (x_3p) singly binding to the DNA with or without the presence of $RNAP$, the unbound (reference) state and the state where only $RNAP$ is bound. The parameters ε_{x_3R} and ε_{x_3pR} (measured in units of RT) are the free energy changes of RNAP binding induced by the binding of x_3 or x_3p to the transcriptional machinery. These free energy changes are negative for activators, thus the multipliers A_4 and A_5 are positive.

Supplementary Table 4.11 - Computation of regulation factor $\boldsymbol{F_{TP53}}$

Promoter-Bound (0 or 1)		*Statistical Weight of the Promoter State*
y_4	$RNAP$	
0	0	$w_1 = 1$
0	1	$w_2 = \frac{[RNAP]}{K_{RNAP}}$
1	0	$w_3 = \frac{[y_4]}{K_{y_4}}$
$F_{TP53} = \left(\frac{[RNAP]}{K_{RNAP}}\right)^{-1} \frac{w_2}{w_1 + w_3} = \frac{1}{1 + \frac{[y_4]}{K_{y_4}}}$		

Supplementary Table 4.11 - Computation of regulation factor $\boldsymbol{F_{TP53}}$. The F_{TP53} regulation factor for the $TFBS_{TP53}$/*TF/RNAP* subsystem is computed via enumeration and statistical weight evaluation of each possible state of the system. There are three states of this promoter: the repressor TP53 (y_4) bound to the promoter, the unbound (reference) state and the state where only $RNAP$ is bound. Note that where we consider a transcriptional repressor we assume that $RNAP$ cannot bind to the promoter to activate transcription.

Supplementary Table 4.12 - Summary of regulation factor expressions

TF Binding Site	Regulation Factor Name	Regulation Factor Expression
$TFBS_{TCF7L2}$	F_{TCF7L2}	$\frac{1+A_1\frac{[x_1]}{K_{x_1}}+A_2\frac{[x_1p]}{K_{x_1p}}+A_3\frac{[x_2]}{K_{x_2}}}{1+\frac{[x_1]}{K_{x_1}}+\frac{[x_1p]}{K_{x_1p}}+\frac{[x_2]}{K_{x_2}}+\frac{[y_1]}{K_{y_1}}}$
$TFBS_{SMAD4}$	F_{SMAD4}	$\frac{1}{1+\frac{[y_2]}{K_{y_2}}+\frac{[y_3]}{K_{y_3}}}$
$TFBS_{AP1}$	F_{AP1}	$\frac{1+A_4\frac{[x_3]}{K_{x_3}}+A_5\frac{[x_3p]}{K_{x_3p}}}{1+\frac{[x_3]}{K_{x_3}}+\frac{[x_3p]}{K_{x_3p}}}$
$TFBS_{TP53}$	F_{TP53}	$\frac{1}{1+\frac{[y_4]}{K_{y_4}}}$
$TFBS_{\mathrm{E2F-DP1}}$	$F_{E2F-DP1}$	$\frac{1+A_6\frac{[x_4]}{K_{x_4}}}{1+\frac{[x_4]}{K_{x_4}}+\frac{[y_5]}{K_{y_5}}}$

Supplementary Table 4.12 - Summary of regulation factor expressions. The regulation factor expressions for each of the TCF7L2, SMAD4, AP1, TP53 and E2F-DP1 binding sites, which bind TF complexes of TCF7L2, SMAD4, AP1, TP53 and E2F-DP1, respectively.

References

1. Wierstra I and Alves J. The c-myc promoter: still MysterY and challenge. Adv Cancer Res. 2008; 99:113-333.
2. Levens D. How the c-myc promoter works and why it sometimes does not. J Natl Cancer Inst Monogr. 2008; (39):41-43.
3. Klein EA and Assoian RK. Transcriptional regulation of the cyclin D1 gene at a glance. J Cell Sci. 2008; 121(Pt 23):3853-3857.
4. Guo ZY, Hao XH, Tan FF, Pei X, Shang LM, Jiang XL and Yang F. The elements of human cyclin D1 promoter and regulation involved. Clin Epigenetics. 2011; 2(2):63-76.
5. Korinek V, Barker N, Morin PJ, vanWichen D, deWeger R, Kinzler KW, Vogelstein B and Clevers H. Constitutive transcriptional activation by a beta-catenin-Tcf complex in APC(-/-) colon carcinoma. Science. 1997; 275(5307):1784-1787.
6. van de Wetering M, Sancho E, Verweij C, de Lau W, Oving I, Hurlstone A, van der Horn K, Batlle E, Coudreuse D, Haramis AP, Tion-Pon-Fong M, Moerer P, van den Born M, Soete G, Pals S, Eilers M, et al. The beta-catenin/TCF-4 complex imposes a crypt progenitor phenotype on colorectal cancer cells. Cell. 2002; 111(2):241-250.
7. He TC, Sparks AB, Rago C, Hermeking H, Zawel L, da Costa LT, Morin PJ, Vogelstein B and Kinzler KW. Identification of c-MYC as a target of the APC pathway. Science. 1998; 281(5382):1509-1512.
8. Piedra J, Martinez D, Castano J, Miravet S, Dunach M and de Herreros AG. Regulation of beta-catenin structure and activity by tyrosine phosphorylation. Journal of Biological Chemistry. 2001; 276(23):20436-20443.
9. Shtutman M, Zhurinsky J, Simcha I, Albanese C, D'Amico M, Pestell R and Ben-Ze'ev A. The cyclin D1 gene is a target of the beta-catenin/LEF-1 pathway. Proceedings of the National Academy of Sciences of the United States of America. 1999; 96(10):5522-5527.
10. Tetsu O and McCormick F. beta-catenin regulates expression of cyclin D1 in colon carcinoma cells. Nature. 1999; 398(6726):422-426.

11. van Veelen W, Le NH, Helvensteijn W, Blonden L, Theeuwes M, Bakker ERM, Franken PF, van Gurp L, Meijlink F, van der Valk MA, Kuipers EJ, Fodde R and Smits R. beta-catenin tyrosine 654 phosphorylation increases Wnt signalling and intestinal tumorigenesis. Gut. 2011; 60(9):1204-1212.

12. Arce L, Pate KT and Waterman ML. Groucho binds two conserved regions of LEF-1 for HDAC-dependent repression. Bmc Cancer. 2009; 9.

13. Daniels DL and Weis WI. beta-catenin directly displaces Groucho/TLE repressors from Tcf/Lef in Wnt-mediated transcription activation. Nature Structural & Molecular Biology. 2005; 12(4):364-371.

14. Lim SK and Hoffmann FM. Smad4 cooperates with lymphoid enhancer-binding factor 1/T cell-specific factor to increase c-myc expression in the absence of TGF-beta signaling. Proceedings of the National Academy of Sciences of the United States of America. 2006; 103(49):18580-18585.

15. Chen CR, Kang YB, Siegel PM and Massague J. E2F4/5 and p107 as Smad cofactors linking the TGF beta receptor to c-myc repression. Cell. 2002; 110(1):19-32.

16. Massague J, Seoane J and Wotton D. Smad transcription factors. Genes & Development. 2005; 19(23):2783-2810.

17. Yagi K, Furuhashi M, Aoki H, Goto D, Kuwano H, Sugamura K, Miyazono K and Kato M. c-myc is a downstream target of the Smad pathway. Journal of Biological Chemistry. 2002; 277(1):854-861.

18. Ko TC, Sheng HM, Reisman D, Thompson EA and Beauchamp RD. Transforming Growth-Factor-Beta-1 Inhibits Cyclin D1 Expression in Intestinal Epithelial-Cells. Oncogene. 1995; 10(1):177-184.

19. Mithani SK, Balch GC, Shiou SR, Whitehead RH, Datta PK and Beauchamp RD. Smad3 has a critical role in TGF-beta-mediated growth inhibition and apoptosis in colonic epithelial cells. Journal of Surgical Research. 2004; 117(2):296-305.

20. Ding ZH, Wu CJ, Chu GC, Xiao YH, Ho D, Zhang JF, Perry SR, Labrot ES, Wu XQ, Lis R, Hoshida Y, Hiller D, Hu BL, Jiang S, Zheng HW, Stegh AH, et al. SMAD4-dependent barrier constrains prostate cancer growth and metastatic progression. Nature. 2011; 470(7333):269-+.

21. Karin M, Liu Z and Zandi E. AP-1 function and regulation. Curr Opin Cell Biol. 1997; 9(2):240-246.

22. Shaulian E and Karin M. AP-1 in cell proliferation and survival. Oncogene. 2001; 20(19):2390-2400.

23. Albanese C, Johnson J, Watanabe G, Eklund N, Vu D, Arnold A and Pestell RG. Transforming p21ras mutants and c-Ets-2 activate the cyclin D1 promoter through distinguishable regions. The Journal of biological chemistry. 1995; 270(40):23589-23597.

24. Bakiri L, Lallemand D, Bossy-Wetzel E and Yaniv M. Cell cycle-dependent variations in c-Jun and JunB phosphorylation: a role in the control of cyclin D1 expression. EMBO J. 2000; 19(9):2056-2068.

25. Zhang HS, Yan B, Li XB, Fan L, Zhang YF, Wu GH, Li M and Fang J. PAX2 protein induces expression of cyclin D1 through activating AP-1 protein and promotes proliferation of colon cancer cells. The Journal of biological chemistry. 2012; 287(53):44164-44172.

26. Iavarone C, Catania A, Marinissen MJ, Visconti R, Acunzo M, Tarantino C, Carlomagno MS, Bruni CB, Gutkind JS and Chiariello M. The platelet-derived growth factor controls c-myc expression through a JNK- and AP-1-dependent signaling pathway. The Journal of biological chemistry. 2003; 278(50):50024-50030.

27. Kerkhoff E, Houben R, Loffler S, Troppmair J, Lee JE and Rapp UR. Regulation of c-myc expression by Ras/Raf signalling. Oncogene. 1998; 16(2):211-216.

28. Ho JSL, Ma WL, Mao DYL and Benchimol S. p53-dependent transcriptional repression of c-myc is required for G(1) cell cycle arrest. Molecular and Cellular Biology. 2005; 25(17):7423-7431.

29. Rocha S, Martin AM, Meek DW and Perkins ND. p53 Represses cyclin D1 transcription through down regulation of Bcl-3 and inducing increased association of the p52 NF-kappa B subunit with histone deacetylase 1. Molecular and Cellular Biology. 2003; 23(13):4713-4727.

30. Bracken AP, Ciro M, Cocito A and Helin K. E2F target genes: unraveling the biology. Trends in Biochemical Sciences. 2004; 29(8):409-417.

31. Chen HZ, Tsai SY and Leone G. Emerging roles of E2Fs in cancer: an exit from cell cycle control. Nature Reviews Cancer. 2009; 9(11):785-797.

32. Carey MF. Transcriptional activation. A holistic view of the complex. Current biology : CB. 1995; 5(9):1003-1005.

33. Posern G and Treisman R. Actin' together: serum response factor, its cofactors and the link to signal transduction. Trends Cell Biol. 2006; 16(11):588-596.

34. Frank TD, Carmody AM and Kholodenko BN. Versatility of cooperative transcriptional activation: a thermodynamical modeling analysis for greater-than-additive and less-than-additive effects. PloS one. 2012; 7(4):e34439.

35. Garcia HG, Sanchez A, Kuhlman T, Kondev J and Phillips R. Transcription by the numbers redux: experiments and calculations that surprise. Trends Cell Biol. 2010; 20(12):723-733.

36. Bintu L, Buchler NE, Garcia HG, Gerland U, Hwa T, Kondev J and Phillips R. Transcriptional regulation by the numbers: models. Current opinion in genetics & development. 2005; 15(2):116-124.

37. Brewster RC, Jones DL and Phillips R. Tuning promoter strength through RNA polymerase binding site design in Escherichia coli. PLoS computational biology. 2012; 8(12):e1002811.

38. Frank TD, Cheong A, Okada-Hatakeyama M and Kholodenko BN. Catching transcriptional regulation by thermostatistical modeling. Physical biology. 2012; 9(4):045007.

39. Kadonaga JT. Regulation of RNA polymerase II transcription by sequence-specific DNA binding factors. Cell. 2004; 116(2):247-257.

40. Smale ST and Kadonaga JT. The RNA polymerase II core promoter. Annual review of biochemistry. 2003; 72:449-479.

41. Hieda M, Winstanley H, Maini P, Iborra FJ and Cook PR. Different populations of RNA polymerase II in living mammalian cells. Chromosome research : an international journal on the molecular, supramolecular and evolutionary aspects of chromosome biology. 2005; 13(2):135-144.

42. Maul GG and Deaven L. Quantitative determination of nuclear pore complexes in cycling cells with differing DNA content. J Cell Biol. 1977; 73(3):748-760.

43. Carey M. The enhanceosome and transcriptional synergy. Cell. 1998; 92(1):5-8.

44. Pyrzynska B, Mosieniak G and Kaminska B. Changes of the trans-activating potential of AP-1 transcription factor during cyclosporin A-induced apoptosis of glioma cells are mediated by phosphorylation and alterations of AP-1 composition. Journal of neurochemistry. 2000; 74(1):42-51.

Supplementary Material 5.1 Examples of behaviors of the model

Our rational modeling approach makes it possible to calculate the effect of all altered proteins (present in the MIM) or a given compound (at any dose) from the point of inhibition down to transcriptional effects. As an example to provide insight into the workings of the MIM we will describe the effect of the MEK inhibitor CI-1040 in HCT116 cells. Considering the "physiological model" (no mutations/alterations) and a concentration of EGF = 0.01 nM (expected in the presence of 10% calf serum), the physiologic concentration of MEKKPP is 2.18 nM, however given the KRAS mutation present in HCT116 cells, this goes up to 80.79 nM (all MEKPP complexes included). Due to the elevated active MEK, activated ERKPP also goes up from .046 nM, in the physiologic model, to 127.51 nM, in the HCT116 model.

In the presence of the MEK inhibitor, CI-1040 (2μM), a new complex was formed [MEKPP:CI-1040]. The residual MEKPP complexes have now a concentration of 24.15 nM, while the concentrations of ERKPP complexes is now down to 17.51 nM. As a result, the downstream components of the pathway are also suppressed reducing the transcription of CCND1 and MYC. The original HCT116 transcription level of c-MYC is = 0.0396 nM. Adding the MEK inhibitor, the c-MYC transcription level goes down to 0.0188 nM. The parallel values for CCND1 are the following: original HCT116 transcription level of CCND1 = 1.874 nM. In the presence of MEK inhibitor we go down to 0.516 nM.

Our model makes it easier to examine both positive and negative feedbacks in the signaling networks and how these are affected by mutations (sometimes competing mutations) or inhibitor treatments.

An example of how the model can be used to elucidate and quantify complicated competing mutational effects is AKT phosphorylation. A KRAS mutation introduced in the physiologic model produces a negative feedback to AKT-P, while an additional PI3K mutation increases AKT-P. Again starting from a physiologic model with EGF = 0.01 nM, a mutated KRAS increases ERKPP (from 0.046 nM to 134.17 nM). This in turn increases phosphorylation of CDC25C (from 12.95 nM to 49.73 nM) which reduces phosphorylated ErbB family proteins (from 6.95 nM to 1.61 nM), through CDC25C phosphatase activity. Reduced ErbBs decrease AKT-P (from 13.52 to 4.93 nM). Conversely, due to the PI3K mutation, activated PI3K increases (from 6.38 nM to 199.6 nM); this in turn increases activated AKT-P (from 13.52 nM to 63.8 nM). Taken together the effects of these two competing (with respect to AKT-P) mutations result in AKT-P becoming 62.02 nM, compared to 13.52 nM in the physiologic model. Considering the effects of the additional four mutations (TGFβR-II, E-Cadherin, PTEN, β-catenin) also present in HCT116 cells, AKT-P becomes 67.01 nM.

This is just one example, but our model can be used to quickly examine the combined effect of any number of mutations and inhibitors, on the level of any of the proteins present in the MIM, accounting not only for linear relationships but also the numerous intricate feedbacks which occur in this signaling network.

Supplementary Material 5.2 Starting from physiologic model: no mutations, KRAS mutation. Inhibitors: no inhibitors, panErb inhibitor, MEK inhibitor, both inhibitors

	EGFRP Family	ERKPP	AKTP	c-MYC	CCND1
Physiologic	1 (5.89)	1 (0.046)	1 (13.52)	1 (0.0039)	1 (0.1853)
Physiologic + PanErb Inhibitor	.18 (1.06)	0.037 (0.0018)	0.18 (2.49)	.37 (0.0014)	.014 (0.0026)
Physiologic + CI-1040	1.03 (6.07)	0.98 (0.045)	1.02 (13.79)	1.01 (0.0039)	0.99 (0.1828)
Physiologic + PanErb Inhibitor + CI-1040	0.18 (1.06)	0.037 (0.017)	0.18 (2.49)	.37 (0.0014)	.014 (0.0025)
KRAS mutated	0.129 (0.76)	2919 (134.17)	0.36 (4.93)	2.75 (0.0108)	2.75 (0.51)
KRAS mutated + PanErb Inhib.	0.055 (0.324)	2893 (133.10)	0.17 (2.30)	1.66 (0.00647)	1.64 (0.304)
KRAS mutated + CI-1040	0.130 (0.76)	450 (20.71)	0.37 (4.96)	1.26 (0.00491)	0.16 (0.030)
KRAS mutated + PanErb Inhib. + CI-1040	0.055 (0.324)	430 (19.80)	0.17 (2.30)	0.74 (0.00298)	0.09 (0.0904)

Comment: EGFR-P, ERKPP and AKT-P (all P-complexes were added together in the computation, for each signaling-protein), modeling at 30 min – 1 h, show a reasonable relative trend, see also Fig. 2 and 4 of (56, reference in main text). C-MYC and CCND1 mRNAs, at 4 – 8 h, show a more integrated behavior, closer to cell growth inhibition in the paper of Misale et al. (56, reference in main text). Consider the last four lines of Table *Supplementary Material 5.2* above, versus Fig. 5 of (56, reference in main text).

We give relative values normalized to 1 for the physiologic condition, and nM values in parenthesis.

Supplementary Material 6 - Simulated and Experimental Data

In Tables Supplementary Material 6.1 (HCT116) and Supplementary Material 6.2 (HT29) we report simulated and experimental values for ERKPP and AKTP proteins (controls normalized to 1) in the presence of different inhibitors, both alone and in association, in HCT116 and HT29 CRC lines, respectively. Experiments performed at 30 min.

In Tables Supplementary Material 6.3 and 6.4 we report simulated and experimental values for MYC and CCND1 mRNA levels (controls normalized to 1) in the presence of different inhibitors, in HCT116 and HT29 CRC lines, respectively. In HCT116 CRC line associations of inhibitors were also explored. Experiments performed both at 4h and 8h are reported and utilized for statistical analyses.

P-PROTEINS EXPERIMENTS PERFORMED WITH HCT116 CANCER LINE

**Table Supplementary Material 6.1**	**ERKPP and AKTP levels in HCT116 cell line**			
**ERKPP**				
	Experimental Data			Simulated Data
Controls	1.000	1.000	1.000	1.000
XAV939 (X)	0.747	1.861	0.568	1.000
PI103 (P)	0.956	1.806	0.494	1.015
CI1040 (C)	0.065	0.339	0.101	0.113
Perifosine 20 (Pe_{20})	0.676	1.228	0.759	1.016
Perifosine 40 (Pe_{40})	0.958	1.107	0.838	1.021
X + P	0.618	0.998	1.224	1.015
X + C	0.046	0.185	0.175	0.113
P + C	0.039	0.248	0.137	0.120
C + Pe_{20}	0.031	0.102	0.103	0.120
C + Pe_{40}	0.045	0.026	0.022	0.122
X + P + C	0.042	0.108	0.097	0.120
Pe20 + X + P + C	0.026	0.077	0.073	0.126
Pe40 + X + P + C	0.022	0.019	0.013	0.127
**AKTP**				
	Experimental Data			Simulated Data
Controls	1.000	1.000	1.000	1.000
XAV939 (X)	0.728	0.634	0.993	1.000
PI103 (P)	0.259	0.396	0.566	0.800
CI1040 (C)	0.935	1.137	1.328	1.000
Perifosine 20 (Pe_{20})	0.494	0.703	0.950	0.571
Perifosine 40 (Pe_{40})	0.171	0.243	0.132	0.395
X + P	0.312	0.343	0.480	0.800
X + C	0.771	0.649	1.349	1.000
P + C	0.418	0.456	0.461	0.801
C + Pe_{20}	0.731	0.446	0.649	0.572
C + Pe_{40}	0.908	0.844	0.812	0.395
X + P + C	0.439	0.370	0.382	0.801
Pe20 + X + P + C	0.417	0.282	0.456	0.319
Pe40 + X + P + C	0.135	0.135	0.138	0.198

P-PROTEINS EXPERIMENTS PERFORMED WITH HT29 CANCER LINE

**Table Supplementary Material 6.2** **ERKPP and AKTP levels in HT29 cell line**				
**ERKPP**				
	Experimental Data			Simulated Data
Controls	1.000	1.000	1.000	1.000
XAV939 (X)	1.002	0.951	0.787	1.000
PI103 (P)	1.207	0.517	0.688	1.021
CI1040 (C)	0.094	0.357	0.041	0.157
Perifosine 20 (Pe_{20})	1.212	0.461	0.630	1.033
Perifosine 40 (Pe_{40})	1.959	1.892	1.282	1.043
X + P	0.942	0.353	0.796	1.021
X + C	0.110	0.146	0.057	0.157
P + C	0.081	0.144	0.037	0.176
C + Pe_{20}	0.066	0.145	0.033	0.187
C + Pe_{40}	0.423	0.108	0.063	0.197
X + P + C	0.063	0.136	0.026	0.176
Pe20 + X + P + C	0.056	0.155	0.025	0.203
Pe40 + X + P + C	0.058	0.030	0.017	0.208
**AKTP**				
	Experimental Data			Simulated Data
Controls	1.000	1.000	1.000	1.000
XAV939 (X)	0.567	0.641	0.485	1.000
PI103 (P)	0.298	0.246	0.353	0.672
CI1040 (C)	0.943	0.321	0.468	1.000
Perifosine 20 (Pe_{20})	0.285	0.215	0.220	0.498
Perifosine 40 (Pe_{40})	0.080	0.239	0.153	0.328
X + P	0.235	0.225	0.276	0.672
X + C	0.637	0.762	0.577	1.000
P + C	0.253	0.231	0.211	0.673
C + Pe_{20}	0.336	0.385	0.236	0.498
C + Pe_{40}	0.471	0.401	0.384	0.328
X + P + C	0.142	0.148	0.214	0.673
Pe20 + X + P + C	0.120	0.116	0.090	0.215
Pe40 + X + P + C	0.180	0.163	0.181	0.127

Tables Supplementary Material 6.1 and 6.2: Protein levels indicated as ratio ppERK/ERK and pAKT/AKT, 30 min treatments. Tables Supplementary Material 6.1 referred to HCT116 cells, Table Supplementary Material 6.2 referred to HT29

cells. Not treated samples normalized to 1. For combination treatments inhibitor names were abbreviated as follows; XAV939 (X), PI-103 (P), CI-1040 (C), Perifosine (Pe)

mRNA EXPERIMENTAL RESULTS PERFORMED WITH HCT116 CANCER LINE

**Table Supplementary Material 6.3** **MYC mRNA levels HCT116 cell line**					
	Experimental Data				Simulated Data
4h Control	1.000	1.000	1.000	1.000	1.000
8h Control	1.000	1.000	1.000	1.000	1.000
4h CI1040 (C)	0.366	0.352	0.264	0.310	0.484
8h CI1040 (C)	0.465	0.415	0.593	0.406	0.473
4h PI103 (P)	1.399	1.274	1.144	1.154	0.972
8h PI103 (P)	1.291	1.392	1.798	1.555	0.969
4h XAV939 (X)	0.978	0.918	0.803	0.793	1.000
4h P + C	0.391	0.449	0.420	0.382	0.477
4h X + C	0.262	0.231	0.198	0.202	0.484
4h X + P	0.987	0.815	0.856	0.781	0.972
4h X + P + C	0.269	0.279	0.279	0.241	0.477
**CCND1 mRNA levels HCT116 cell line**					
	Experimental Data				Simulated Data
4h Control	1.000	1.000	1.000	1.000	1.000
8h Control	1.000	1.000	1.000	1.000	1.000
4h CI1040 (C)	0.571	0.548	0.349	0.530	0.512
8h CI1040 (C)	0.250	0.225	0.294	0.218	0.275
4h PI103 (P)	0.885	0.882	0.736	0.776	0.999
8h PI103 (P)	1.064	0.961	1.248	1.088	0.998
4h XAV939 (X)	0.990	0.947	0.866	0.853	1.000
4h P + C	0.345	0.418	0.370	0.412	0.533
4h X + C	0.636	0.589	0.496	0.521	0.512
4h X + P	0.706	0.711	0.677	0.727	0.999
4h X + P + C	0.448	0.446	0.439	0.435	0.533

Tables Supplementary Material 6.3: MYC and CCND1 mRNA levels referred to HCT116 cells

Not treated samples normalized to 1. For combination treatments inhibitor names were abbreviated as follows; XAV939 (X), PI-103 (P), CI-1040 (C).

mRNA EXPERIMENTAL RESULTS PERFORMED WITH HT29 CANCER LINE

Table Supplementary Material 6.4	MYC mRNA levels HT29 cell line				
	Experimental Data				Simulated Data
4h Control	1.000	1.000	1.000	1.000	1.000
8h Control	0.780	0.999	0.820	1.041	1.000
4h CI1040 (C)	0.102	0.115	0.106	0.109	0.556
8h CI1040 (C)	0.067	0.052	0.060	0.055	0.547
4h PI103 (P)	0.942	1.105	0.956	1.257	1.049
8h PI103 (P)	1.047	1.019	0.909	0.956	1.050
4h Azakenpaullone	1.164	1.229	1.016	1.277	1.000
8h Azakenpaullone	1.409	1.334	1.168	1.316	1.000
CCND1 mRNA levels HT29 cell line					
	Experimental Data				Simulated Data
4h Control	1.000	1.000	1.000	1.000	1.000
8h Control	0.936	0.811	0.960	0.916	1.000
4h CI1040 (C)	0.522	0.398	0.487	0.467	0.655
8h CI1040 (C)	0.162	0.103	0.130	0.133	0.440
4h PI103 (P)	0.929	0.948	0.773	1.118	1.005
8h PI103 (P)	0.952	0.909	0.753	0.987	1.010
4h Azakenpaullone	1.212	0.985	1.012	1.089	1.000
8h Azakenpaullone	1.350	1.190	0.995	1.383	1.000

Tables Supplementary Material 6.4: CCND1 mRNA an MYC levels referred to HT29 cells.

Not treated samples normalized to 1. For combination treatments inhibitor names were abbreviated as follows; XAV939 (X), PI-103 (P), CI-1040 (C).
